# Antistaphylococcal Triazole-Based Molecular Hybrids: Design, Synthesis and Activity

**DOI:** 10.3390/ph18010083

**Published:** 2025-01-11

**Authors:** Kostiantyn Shabelnyk, Alina Fominichenko, Oleksii Antypenko, Olexandr Gaponov, Svitlana Koptieva, Svitlana Shyshkina, Oleksii Voskoboinik, Sergiy Okovytyy, Serhii Kovalenko, Valentyn Oksenych, Oleksandr Kamyshnyi

**Affiliations:** 1 Department of Pharmaceutical, Organic and Bioorganic chemistry, Zaporizhzhia State Medical and Pharmaceutical University, 69000 Zaporizhzhia, Ukraine; 2 Bacteriological Laboratory, Zaporizhzhia Regional Hospital, 69600 Zaporizhzhia, Ukraine; 3 Institute of Chemistry and Geology, Oles Honchar Dnipro National University, 49000 Dnipro, Ukraine; 4 SSI “Institute for Single Crystals” of the National Academy of Sciences of Ukraine, 61072 Kharkiv, Ukraine; 5 Department of Composite Materials, Chemistry and Technologies, National University «Zaporizhzhia Polytechnic», 69063 Zaporizhzhia, Ukraine; a.yu.voskoboynik@gmail.com; 6 Faculty of Medicine, University of Bergen, 5020 Bergen, Norway; 7 Department of Microbiology, Virology and Immunology, I. Horbachevsky Ternopil State Medical University, 46001 Ternopil, Ukraine; kamyshnyi_om@tdmu.edu.ua

**Keywords:** triazole, «one-pot» synthesis, molecular docking, antistaphylococcal activity

## Abstract

Background: In the era of resistance, the design and search for new “small” molecules with a narrow spectrum of activity that target a protein or enzyme specific to a certain bacterium with high selectivity and minimal side effects remains an urgent problem of medicinal chemistry. In this regard, we developed and successfully implemented a strategy for the search for new hybrid molecules, namely, the not broadly known [2-(3-R-1*H*-[1,2,4]-triazol-5-yl)phenyl]amines. They can act as “building blocks” and allow for the introduction of certain structural motifs into the desired final products in order to enhance the antistaphylococcal effect. Methods: The “one-pot” synthesis of the latter is based on the conversion of substituted 4-hydrazinoquinazolines or substituted 2-aminobenzonitriles and carboxylic acid derivatives to the target products. The possible molecular mechanism of the synthesized compounds (DNA gyrase inhibitors) was investigated and discussed using molecular docking, and their further study for antistaphylococcal activity was substantiated. Results: A significant part of the obtained compounds showed high antibacterial activity against *Staphylococcus aureus* (MIC: 10.1–62.4 µM) and 5-bromo-2-(3-(furan-3-yl)-1*H*-1,2,4-triazol-5-yl)aniline and 5-fluoro-2-(3-(thiophen-3-yl)-1*H*-1,2,4-triazol-5-yl)aniline, with MICs of 5.2 and 6.1 µM, respectively, approaching the strength of the effect of the reference drug, “Ciprofloxacin” (MIC: 4.7 µM). The conducted SAR and ADME analyses confirm the prospects of the further structural modification of these compounds. The obtained [2-(3-R-1*H*-[1,2,4]-triazol-5-yl)phenyl]amines reveal significant antimicrobial activity and deserve further structural modification and detailed study as effective antistaphylococcal agents. The SAR analysis revealed that the presence of a cycloalkyl or electron-rich heterocyclic fragment in the third position of the triazole ring was essential for the antibacterial activity of the obtained compounds. At the same time, the introduction of a methyl group into the aniline moiety led to an enhancement of activity. The introduction of halogen into the aniline fragment has an ambiguous effect on the level of antistaphylococcal activity and depends on the nature of the substituent in the third position. Conclusions: Obtained [2-(3-R-1*H*-[1,2,4]-triazol-5-yl)phenyl]amines reveal significant antistaphylococcal activity and deserve for further detailed study as effective antibacterial agents.

## 1. Introduction

Despite the progress of antibiotic therapy, the sensitivity of microorganisms to existing antimicrobials is significantly decreasing from year to year, and antibiotic-resistant strains of microorganisms are emerging as hospital and non-hospital infections. This is due to the limited number of effective medicines used to treat infections [1,2], and to their uncontrolled use, which leads to synergistic combinations of known and new resistance mechanisms [3,4].

Especially dangerous are infections caused by *Staphylococcus aureus*, which is primarily associated with many virulence factors, including toxins, superantigens and exoproteins that are associated with the cell membrane. In addition, the emergence of methicillin-resistant *Staphylococcus aureus* (MRSA) strains both in health care settings and in the community has increased the risk of infections, as they usually have multiple drug resistance. This results in serious health problems for patients, increased morbidity and mortality, an increased length of hospital stays and a significant economic burden on the health care sector. At the same time, a wide arsenal of natural antibiotics and their constant replenishment with new semi-synthetic and synthetic antimicrobial drugs [5,6] has not solved the mentioned problem, despite the fact that the design and search for antibacterial agents have undergone significant changes [7,8]. The problem of treating infections caused by MRSA is further complicated by the fact that MRSA acquires antibiotic resistance genes in various ways [1,4,6]. This became particularly acute after the registration of strains resistant to Vancomycin, which was the drug of choice for the treatment of MRSA [9].

**Figure 1:**
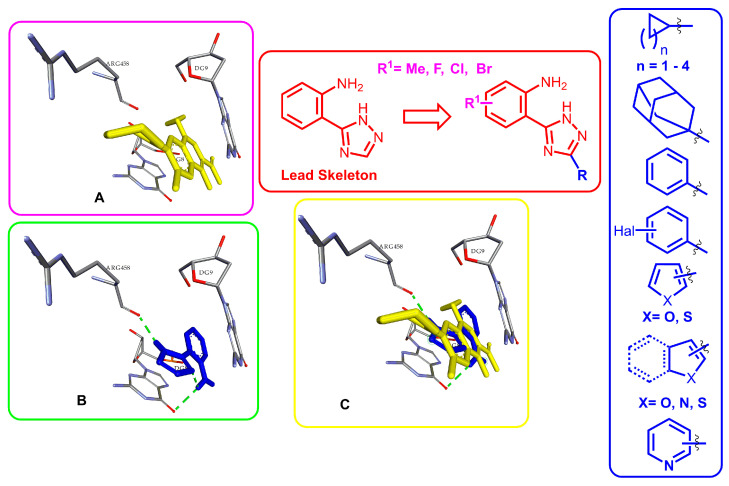
The designed structures of the target compounds and conformations of the standard ligand, Ciprofloxacin (**A**), and 2-(1,2,4-triazol-5-yl)aniline (**B**) and their combined conformation (**C**) in the active site of 2XCT DNA gyrase.

An important strategy for the search for effective antistaphylococcal agents is research aimed at inhibiting the growth of bacteria by blocking the transmission of information from bacterial DNA and RNA [10,11,12,13,14,15]. Essential enzymes for DNA replication and transcription in this case are DNA gyrase, topoisomerase IIA and topoisomerase IV—important targets for bacterial inhibitors [16,17]. To date, a significant number of noted inhibitors have been synthesized, but attempts to introduce new antimicrobial agents of DNA gyrase inhibitors have been unsuccessful (Novobiocin) [10,12], and the latest development (Gepotidacin, GSK2140944) is in the third phase of clinical trials [18]. Therefore, the complexity and variety of resistance mechanisms remain the main factors that prompt scientists to further develop new antibacterial drugs. In most cases, the design is aimed at the modification of known antibiotics and antibacterial drugs [15,19,20,21], the synthesis of new “small” molecules with a narrow spectrum of activity [15,22,23], the development of antibacterial polypeptides [24], antibacterial complexes with transition metals [25], etc. An important class among “small” molecules are also new hybrid structures based on 1,2,4-triazole, which have significant potential for the realization of a polytargeted mechanism of activity and promising broad-spectrum antibacterial activity against a number of clinically important pathogens, including antibiotic-resistant strains [26,27,28,29,30]. In particular, it has been shown that 1,2,4-triazole-azoles, 1,2,4-triazole-coumarins, 1,2,4-triazole-β-lactams, 1,2,4-triazole-pyrimidines, 1,2,4-triazole-quinolines and 1,2,4-triazole-quinazolines are highly active against both sensitive and resistant pathogens and are not inferior in effectiveness to first-line antibacterial drugs. Additionally, it has been shown that 1,2,4-triazole-3-thiones are able to restore the β-lactam susceptibility of resistant *Escherichia coli* laboratory strains and *Klebsiella pneumoniae* clinical isolates via the inhibition of metallo-β-lactamase [31]. So, interesting objects in the direction of this research could be substituted 2-(3-R-1,2,4-triazol-5-yl)anilines; their choice is not accidental and, first of all, is related to the peculiarity of the structure [32,33]. Thus, the specified structures are characterized by conformational and configurational isomerism and, in most cases, have a low molecular weight and the required number of donors and acceptors in the molecule due to the structural modification of the benzene ring and the third position of the triazole ring. It is also important to be able to change several physicochemical parameters, such as the solubility and lipophilicity. Another important selection criterion was a small topological polar surface area, which indicates a high ability to easily penetrate the blood–brain barrier and flexibly interact with the biological target. In addition, the performed molecular docking gave us a general idea about the interactions and similar arrangement in the active site of the DNA gyrase (2XCT) of 2-(1,2,4-triazol-5-yl)aniline (TA) and the standard ligand, Ciprofloxacin (Figure 1).

Therefore, in this study, we aimed to develop methods of synthesis, to evaluate the antistaphylococcal effect of little-known hybrid molecules created by combining 2-(1,2,4-triazol-5-yl)anilines with various molecular “pharmacophore” fragments (Figure 1) and to conduct molecular docking and the qualitative and quantitative analyses of the “structure–activity” relationship to understand their potential as effective antistaphylococcal agents.

## 2. Results and Discussion

### 2.1. Chemical Studies

Within the framework of this work, we were interested in the development of a “one-pot” synthesis of [2-(3-R-1H-[1,2,4]-triazol-5-yl)phenyl]amines (**2**), which, in addition, would allow for their further application as building blocks for the formation of new biologically active heterocycles and the study of their potential antistaphylococcal activity. Moreover, the methods of their synthesis are multistep [34,35,36] or based on the degradation of triazolo[c]quinazoline systems [37]. In search of a convenient method for the synthesis of new [2-(3-R-1H-[1,2,4]triazol-5-yl)phenyl]amines (**2**), we drew attention to the ease of the Dimroth rearrangement and the nucleophilic opening of the pyrimidine ring in a series of triazolo[c]quinazolines [38]. Theoretical calculations of the mechanisms of these reactions showed that acid-catalytic hydrolysis with the participation of an equimolecular amount of water is necessary for the Dimroth rearrangement, and for the opening of the pyrimidine cycle, the same acid hydrolysis with an excess of water is necessary [38].

**Scheme 1:**
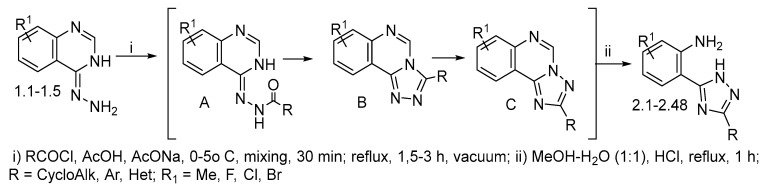
Synthesis of target compounds **2** using substituted 4-hydrazinoquinazolines as initial compounds.

Our attempt to conduct a “one-pot” synthesis of 2-(3-cyclopropyl-1H-1,2,4-triazol-5-yl)-aniline (**2.1**) was successful (Scheme 1). Indeed, the acylation of compound **1.1** by cyclopropane carbonyl chloride in acetic acid in the presence of sodium acetate quantitatively yielded hydrazide (A), which was subjected to heterocyclization without isolation (Method A). The subsequent process of the nucleophilic opening of the triazolo[c]quinazoline ring (C) required the removal of the solvent and the addition of a methanol–water mixture (5:1) acidified with a mineral acid. At the same time, compound **2.1** was formed with an almost quantitative yield (98%). Inspired by these results, we carried out a “one-pot” synthesis of compounds **2.2-2.48** (Scheme 1). The developed procedure has some peculiarities, including the commercial unavailability of some acyl halides, which led to certain modifications of the method, namely, the in situ preparation of acyl chlorides.

**Scheme 2:**
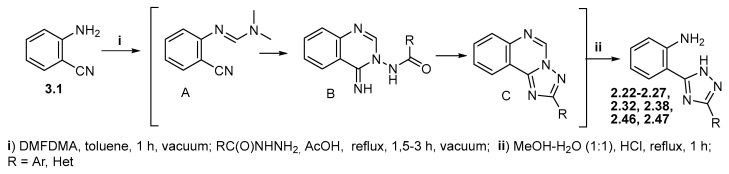
Synthesis of target compounds **2** using 2-aminobenzonitriles as initial compounds.

Another alternative “one-pot” synthesis of compounds **2** was tested by us starting from 2-aminobenzonitrile (**3.1**, Scheme 2). Thus, the latter under the action of DMF/DMA was transformed into N’-(2-cyanophenyl)-N,N-dimethylformimidamides (A), which, after removing the excess reagent and solvent, were subjected to heterocyclization with hydrazides of carboxylic acids in acetic acid (Method B). At the same time, the triazolo[c]quinazoline cycle (C) was quantitatively formed after the removal of the solvent. The last technological process completely repeated the previous Method A. At the same time, the target products **2.22-2.27**, **2.32**, **2.38**, **2.46** and **2.47** were formed with almost quantitative yields.

The structure and purity of the obtained compounds were verified by a complex of physicochemical methods, including elemental analysis, LC/MS, ^1^H and ^13^C NMR, and X-ray. It was shown that in the LC-MS spectra of all the synthesized compounds, signals with m/z values that correspond to the proposed structures were present.

The formation of [2-(3-R-1H-[1,2,4]-triazol-5-yl)phenyl]amines (**2**) drastically changes the pattern of ^1^H NMR spectra in comparison with intermediate [1,2,4]triazolo[1,5-c]quinazolines (C) [39]. Firstly, it is the absence in the ^1^H NMR spectra of the singlet signal of the fifth-position proton of the tricyclic system (C) in a low field (9.70–9.25 ppm), whereas, in the ^1^H NMR spectra of compounds **2**, there are signals of protons of the NH_2_ group of the aniline fragment, which are registered in the spectrum in the form of a broadened singlet or a doubled singlet at 6.72–5.97 ppm (**2.1**, **2.2**, **2.4-2.6**, **2.8-2.14**, **2.16-2.18**, **2.20-2.23**, **2.26**, **2.28**, **2.30**, **2.31**, **2.33**-**2.42**, **2.45**, **2.46**), in the form of a multiplet together with aromatic protons (**2.3**, **2.7**, **2.15**, **2.19**, **2.24**, **2.25**, **2.27**, **2.29**, **2.32**, **2.47**, **2.48**) or that are absent from the spectrum (**2.43**, **2.44**,). The broadening and doubling of the noted protons in the ^1^H NMR spectrum can be explained by the azole–azole (prototropic) tautomerism of compounds **2** [32,37]. In favor of tautomeric transitions in molecules, the broadening, doubling or absence (**2.24**, **2.25**, **2.27**, **2.32**, **2.38**, **2.43**) of the singlet NH-proton signal of the triazole ring in a low magnetic field is also indicated. In addition, the ^1^H NMR spectra of compounds **2** are characterized by the signals of the aromatic protons of the aniline fragment, which undergo a diamagnetic shift due to the electron-donating effect of the amino group. Synthesized compounds are additionally characterized by signals of protons of substituents at the third position of the triazole ring, the chemical shift and multiplicity of which are determined by the nature of the substituent [40]. The ^13^C NMR spectra of compounds **2** are characterized by a significant paramagnetic shift of the C1 atom of the aniline fragment (147.7–141.4 ppm) compared to other carbon atoms of the aromatic system, due to the donor effect of the amino group, which indicates the hydrolytic cleavage of the pyrimidine ring. The signals of the C3 and C5 atoms of the triazole ring in compounds 2 are registered as broadened singlets at 162.8–153.7 ppm and 171.5–154.1 ppm (**2.17**, **2.22**-**2.26**) or are absent (**2.1**, **2.13**, **2.32**, **2.46**) in the spectra, which also confirms tautomeric transitions in DMSO-d6 solutions.

**Figure 2:**
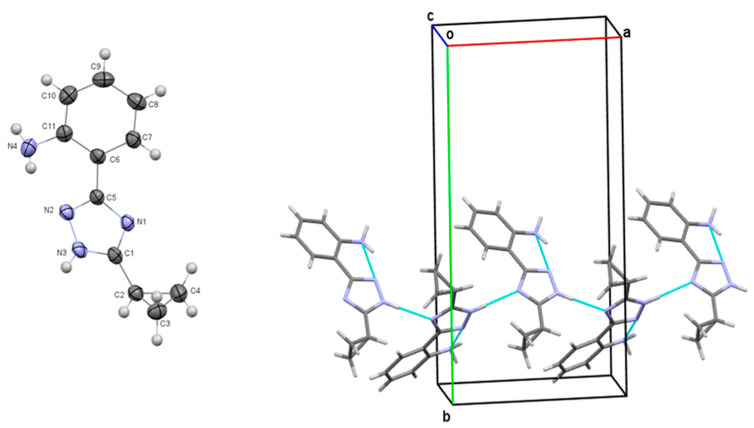
Molecular structure of compound **2.1** according to the X-ray diffraction data (thermal ellipsoids of non-hydrogen atoms are shown at 50% probability level) and hydrogen-bonded chain of molecules 2.1 in the crystal phase.

To unambiguously confirm the “one-pot” synthesis of compounds **2.1**, we performed an X-ray crystallographic study of compound **2.1**. The single crystal for the X-ray studies was grown by the crystallization of compound **2.1** from methanol. The triazole ring is not coplanar to the phenyl moiety (the C11–C6–C5–N2 torsion angle is 16.0(6)°) due to the opposite influences of two factors: (1) the intramolecular hydrogen bond N4–H…N2′ (the H…N distance is 2.02 Å; the N–H…N angle is 142°) and (2) the steric repulsion between two aromatic rings (the short intramolecular contact H7…N1 2.57 Å compared to the van der Waals radius sum [41] 2.67 Å). The amino group has a pyramidal configuration, and the sum of the bond angles centered at the N4 atom is 328°. The cyclopropyl fragment is turned in such a way that the C2–H bond is sin-periplanar to the C1–N3 endocyclic bond (the N3–C1–C2–H2 torsion angle is 11.6°). In the crystal phase, molecules of compound 2.1 form chains in the crystallographic direction (Figure 2) due to the intermolecular hydrogen bonds N3–H…N1′ (symmetry operation: 0.5+x,1.5-y,z; the H…N distance is 2.01 Å; the N–H…N angle is 172°).

### 2.2. Molecular Docking Studies

To optimize further in vitro studies on the antistaphylococcal activity of the synthesized derivatives, as well as to determine the possible molecular mechanism of their action, a procedure of molecular docking to the active site of DNA gyrase inhibitors was carried out. The results of the affinity calculation in kcal/mol and the details of the interactions with respect to the reference ligand—”Ciprofloxacin”—are shown in Table 1 and Table S1. It was established that almost all the studied ligands (except for compounds **2.17-2.21**) demonstrated a high degree of affinity for the site of the DNA gyrase enzyme inhibitor, and the affinity ranged from −5.6 to −9.2 kcal/mol compared to −6.7 kcal/mol for “Ciprofloxacin”.

**Figure 3:**
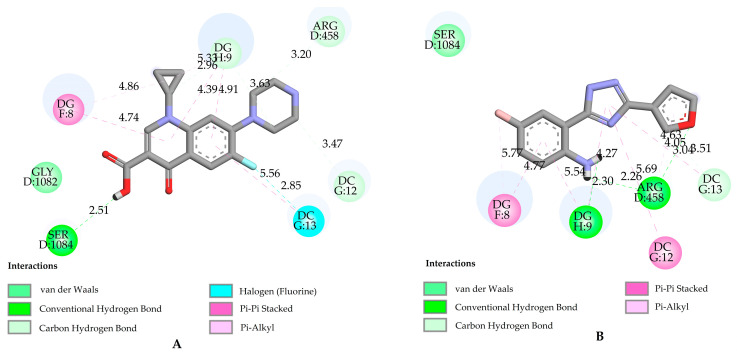
Interactions of “Ciprofloxacin” (**A**) and compound **2.31** (**B**) with amino acid residues and nucleotides of the active site of DNA gyrase in 2D conformation.

**Figure 4:**
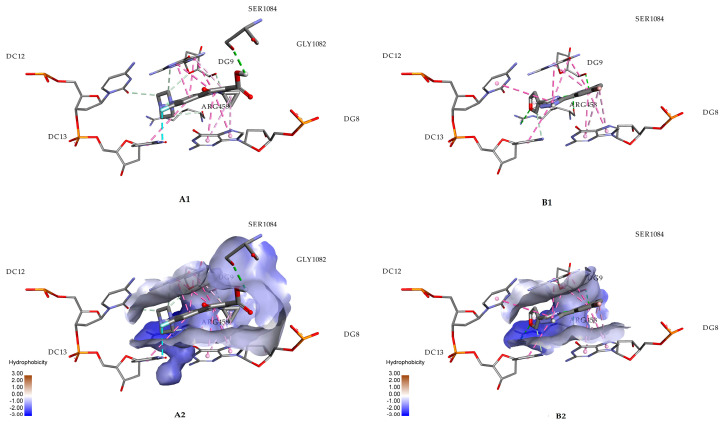
Interaction of “Ciprofloxacin” (**A1**,**A2**) and compound **2.31** (**B1**,**B2**) with amino acid residues and nucleotides of the active site of DNA gyrase in 3D conformation.

As visualized in Figure 3A, the standard ligand, “Ciprofloxacin”, has hydrogen bonds in the -COOH group of the third position with SER1048 (2.51 Å) and in the NH group of the piperazine ring with ARG458 (3.20 Å), a fluorine atom with the nucleotide base DC13 (2.85 Å) and van der Waals interactions with the nucleotides DG9 (2.96; 3.01; 3.25 Å) and G:DC12 (3.47 Å). In addition, the standard ligand is characterized by a number of hydrophobic π–π-shaped and π–alkyl interactions with the nucleotide bases DG8 (4.11; 4.33; 5.05; 5.50 Å), G:DC13 (5.56 Å) and DG9 (3.43; 4.10; 4.18; 4.33 Å) of DNA, which stabilize it within the active site (Table 1, Table S1, Figure 4A1,A2).

The conformations of the key 2-(1,2,4-triazol-5-yl)aniline fragments in all the studied ligands in the active site are practically the same and occupy a place similar to the quinoline cycle (Table S1, Figure 3 and Figure 4). In our opinion, this is related to the small topological polar surface area, which outlines the large number of similar interactions with the experimentally determined amino acids or nucleotides in the active site (Table S1). A significant difference from the standard ligand is that the studied ligands did not interact with the SER1048 residue, which was provided by the -COOH group of “Ciprofloxacin”.

At the same time, 2-(1,2,4-triazol-5-yl)anilines with acceptor substituents and, as a result, high affinity were fixed in the active site of DNA gyrase due to the much larger number of hydrogen bonds with ARG458 and nucleotide bases (DG8, DG9, DC12 and DC13) (Table S1). For example (Figure 3B), the stable “ligand–receptor” conformation for **2.31** was fixed due to the predicted five hydrogen bonds with ARG458 (2.26; 3.04; 3.43 Å), DG9 nucleotides (2.30 Å) and G:DC13 (3.51 Å), as well as the hydrophobic π–π-stacked interactions with DG8 (4.21; 4.36 Å), DG9 (3.74; 4.05; 4.15; 4.71 Å), DC12 (5.69 Å) and DC13 (4.63 Å) (Figure 4B1,B2). In addition, ligand **2.31** has an intramolecular hydrogen bond, N4-NH2 (1.85 Å), which is also predicted in other compounds (Figure 2, Table S1), and which apparently stabilizes the molecule in a certain more favorable conformational form.

Thus, the detailed conformational analysis of the investigated ligands **2**, exemplified by **2.31**, and “Ciprofloxacin” (Table S1, Figure 4), which exhibit high affinity, demonstrated similarity in their spatial arrangements. This confirms their ability to penetrate the hydrophilic pocket of DNA gyrase and form stable conformations within the active site. The aforementioned observations indicate a high probability that the synthesized ligands will manifest antistaphylococcal activity through DNA gyrase inhibition. For the in vitro investigation, all the synthesized compounds were selected to gain a more comprehensive understanding of the structure–activity relationship.

### 2.3. Antistaphylococcal Activity of Synthesized Compounds

The antistaphylococcal activity of compounds **2** was studied against the Staphylococcus aureus ATCC 25923 strain (Table 2). Most of the synthesized compounds showed antibacterial activity against S. aureus (MIC: 5.2–933.4 μM; MBC: 10.4–933.4 μM). Thus, among the cycloalkyl-substituted (**2.1-2.21**) compounds, **2.1**, **2.5**, **2.9**, **2.12**, **2.17** and **2.18** show a high antibacterial effect, their MICs are in the range of 10.1–438.0 μM and their MBCs are 20.2–438.0 μM. The highest antistaphylococcal activity is characteristic of compounds **2.17** and **2.18**; namely, their MICs are 10.1–10.6 μM, their MBCs are 20.2–21.2 μM and they are closest in effect to the reference drug, “Ciprofloxacin” (MIC: 4.7 μM; MBC: 9.6 μM). The 2-(3-Aryl-1H-1,2,4-triazol-5-yl)anilines (2.22–2.26) also exhibited antistaphylococcal activity (MICs: 12.4–317.3 μM; MBCs: 24.8–786.6 μM), and, importantly, compound 2.26 showed the highest antibacterial activity (MIC: 12.4 μM; MBC: 24.8 μM). Among the 2-(3-hetaryl-1H-1,2,4-triazol-5-yl)anilines (**2.27-2.48**), high antistaphylococcal activity (MICs: 5.5–25.6 μM; MBCs: 10.4–52.8 μM) was shown for compounds **2.28-2.31**, **2.33-2.35**, **2.39, 2.41** and **2.46**, and all the other compounds showed slightly lower activity (MICs: 42.5–221.0 μM; MBCs: 84.9–442.0 μM).

The most active among the investigated compounds against the S. aureus strain were found to be 5-bromo-2-(3-(furan-3-yl)-1H-1,2,4-triazol-5-yl)aniline (**2.31**) and 5-fluoro-2-(3-(thiophen-3-yl)-1H-1,2,4-triazol-5-yl)aniline (**2.35**), with MICs of 5.2 and 6.1 μM, respectively, approaching the reference drug, “Ciprofloxacin”, in potency (Table 2). In addition, based on the MBC/MIC ratio (Table 2), it was established that all these compounds (except **2.9**, **2.13**) exhibit bactericidal activity, which has a certain advantage over bacteriostatic activity.

### 2.4. SAR Analysis

On the basis of the molecular docking and the obtained results of the synthesized compounds’ antibacterial activity (Table 2) against S. aureus, the “structure–activity” can be generalized as follows:

- The introduction of the cyclopropane fragment to the third position of the triazole fragment of 2-(1*H*-1,2,4-triazol-5-yl)aniline leads to the appearance of an antibacterial effect against *S. aureus.* The extension of the aliphatic cycle by one or more homologous units increases the antibacterial effect, and the presence of the classic “pharmacophoric” fragment of adamantane in the molecule leads to a high antistaphylococcal effect. Conversely, the modification of the aniline moiety of the molecule through the introduction of halogens results in a loss of antibacterial activity in nearly all instances;
- Replacing the cycloalkyl fragment at the third position of the triazole cycle with the phenyl fragment does not lead to a loss of antistaphylococcal activity, whereas the introduction of a halogen to the phenyl fragment in the third position leads to its reduction, and the relocation of fluorine to the ortho position results in a significant increase thereof;
- The introduction of five- or six-membered heterocyclic fragments to the third position of the triazole cycle, which are electron donors due to the heteroatom (O, N, S), unambiguously leads to high antistaphylococcal activity. The aforementioned phenomenon is associated with an increase in π–electron interactions with nucleotides and, consequently, a greater similarity of contents in the active site of the enzyme. The introduction of the methyl group to the aniline moiety leads to an enhancement of activity. Unlike 2-(3-cycloalkyl-1*H*-1,2,4-triazol-5-yl)anilines, the introduction of halogens to the aniline moiety of 2-(3-hetheryl-1*H*-1,2,4-triazol-5-yl)anilines leads to an enhancement of activity.

Thus, the emergence of antistaphylococcal activity in the synthesized compounds can be attributed to their structural features, which are responsible for interactions with the active center of DNA gyrase. It can be observed that the H-bonding domain of 2-(1,2,4-triazol-5-yl)anilines is similar to that of the standard ligand, “Ciprofloxacin”. Certain differences, such as the absence of the -COOH group, are compensated for by acceptor substituents at positions three and four(five) of the molecule.

### 2.5. SwissADME Analysis

The concept of “drug-likeness” plays a pivotal role in the discovery of novel therapeutic agents, offering critical guidelines during the initial phases of drug development and enhancing the probability of success in clinical trials [42]. These characteristics significantly impact pharmacokinetic properties—such as absorption, distribution, metabolism, and excretion—which, in turn, affect the pharmacological activity and overall efficacy of the medicinal agents under investigation. The “drug-likeness” criteria for the most potent compounds (**2.17**, **2.18**, **2.26**, **2.28**, **2.30**, **2.31**, **2.34**, **2.35**, **2.39**, **2.46**) and “Ciprofloxacin” are presented in Table 3.

The results of the virtual screening demonstrated that the compounds, as well as the reference drug, Ciprofloxacin, comply with the “drug-likeness” requirements according to the criteria of the MW (Da) (<500), n-HBA (<10), n-HBD (≤5) and TPSA (<140 Å^2^). The satisfactory TPSA value (<140 Å^2^) correlates well with passive molecular transport across membranes, and the compounds exhibit a high capacity to penetrate the blood–brain barrier and flexibly interact with the macromolecular target. It was shown that the reference drug, Ciprofloxacin, had the lowest LogP value among the studied molecules. The LogP value of Compound **2.46** was similar to that of Ciprofloxacin, which was probably associated with the presence of the pyridine moiety. All the other studied molecules are more lipophilic than Ciprofloxacin, and the high LogP values, especially, are characteristic of adamantane-containing molecules. The investigated structures also showed favorable results in a bioavailability assessment [48], with an index of 0.55. Furthermore, the compounds were evaluated using five different filters (Lipinski, Veber, Muegge, Ghose, Egan) [43,44,45,46,47] employed by pharmaceutical companies to analyze molecules with the aim of enhancing the quality of their chemical collections. As observed, the calculated/predicted physicochemical descriptors indicate that the investigated compounds, in most cases, meet the requirements of all the filters without deviations. Finally, the majority of the compounds demonstrate a high level of drug-likeness and are suitable for further optimization. Thus, the satisfactory parameters from the SwissADME analysis allow for the subsequent modification of promising compounds in this class to achieve the desired parameters.

## 3. Materials and Methods

### 3.1. Synthetic Section

Melting points were measured in open capillary tubes using a «Mettler Toledo MP 50» apparatus (Columbus, USA). Elemental analyses (C, H, N) were conducted on an ELEMENTAR vario EL cube analyzer (Langenselbold, Germany), with the results for elements or functional groups deviating by no more than ± 0.3% from the theoretical values. The ^1^H and ^13^C NMR spectra (500 MHz) were obtained on a Varian Mercury 500 spectrometer (Varian Inc., Palo Alto, CA, USA), using TMS as an internal standard in a DMSO-d_6_ solution. LC-MS data were acquired using a chromatography/mass spectrometric system comprising the high-performance liquid chromatography «Agilent 1100 Series» (Agilent, Palo Alto, CA, USA) equipped with a diode-matrix detector and mass-selective detector, «Agilent LC/MSD SL» (Agilent, Palo Alto, USA), with atmospheric pressure chemical ionization (APCI).

*General procedure for the synthesis of [2-(3-R-1H-[1,2,4]triazol-5-yl)phenyl]amines* (**2.1-2.48**).

*Method A.* An amount of 0.82 g (0.01 M) of sodium acetate was added to a suspension of 0.01 M of substituted 4-hydrazinoquinazolines (**1.11.5**) in 15 mL of glacial acetic acid, and the formed mixture was cooled to 0–5 °C. An amount of 0.01 M of commercially available acyl chlorides in 5 mL of glacial acetic acid (or a freshly prepared solution of 0.01 mol of the corresponding acyl chloride in 15 mL of dioxane) was added dropwise and the reactional mixture was stirred continuously for 30 min. After that, the reaction mixture was refluxed for 1.5–3 h. While refluxing, water (or the water–dioxane azeotrope) was removed by distillation with a Dean–Stark trap. After the completion of the reaction, the solvent was completely removed under vacuum, and 10 mL of methanol, 10 mL of water and 1 mL of concentrated hydrochloric acid were added to the residue and refluxed for 1 h. After cooling, the reaction mixture was poured into a saturated solution of sodium acetate, controlling the pH to 4–5. The resulting precipitate was filtered off and dried. The compounds were crystallized from methanol or propan-2-ol.

*Method B.* To the solution of 1.18 g (0.01 M) of 2-aminobenzonitrile (**3.1**) in 5 mL of toluene, 2.38 g (0.02 M) of N,N-dimethylformamide dimethyl acetal (DMF-DMA) and acetic acid (0.10 mL) were added and heated at 60 °C for 60 min. Toluene and excess DMF-DMA were completely removed under vacuum [49]. Amounts of 0.01 M of the corresponding carboxylic acid hydrazide and 10 mL of glacial acetic acid were added to the residue. The reaction mixture was refluxed for 1.5–3 h with the removal of water with the Dean–Stark trap. After the procedure, the solvent was removed under vacuum to dryness, and 10 mL of methanol, 10 mL of water and 1 mL of concentrated hydrochloric acid were added to the residue and refluxed for 1 h. After cooling, the reaction mixture was poured into a saturated solution of sodium acetate, controlling the pH to 4–5. The resulting precipitate was filtered off and dried. The compounds were crystallized from methanol or propan-2-ol.

*2-(3-Cyclopropyl-1H-1,2,4-triazol-5-yl)aniline* (**2.1**): yield: 98.0% (Method A); mp 209–211 °C; ^1^H NMR, δ = 1.16–0.81 (m, 4H, cyclopropyl H-2,2,3,3), 2.13–1.97 (m, 1H, cyclopropyl H-1), 6.26 (br.s, 2H, NH_2_), 6.52 (t, J = 7.5 Hz, 1H, H-4), 6.69 (d, J = 8.1 Hz, 1H, H-6), 7.00 (t, J = 7.8 Hz, 1H, H-5), 7.76 (d, J = 6.9 Hz, 1H, H-3), 13.46 (br.s, 1H, NH); ^13^C NMR, δ 147.1 (aniline C-1), 130.1 (aniline C-3), 127.9 (aniline C-5), 116.2 (aniline C-4), 115.6 (aniline C-6), 101.7 (aniline C-2), 39.6 (cyclopropyl C-1), 8.2 (cyclopropyl C-2,3); LC-MS, *m*/*z* = 201 [M+1]; calculated for C_11_H_12_N_4_: C, 65.98; H, 6.04; N, 27.98; found: C, 65.96; H, 6.05; N, 27.97.

*2-(3-Cyclopropyl-1H-1,2,4-triazol-5-yl)-6-methylaniline* (**2.2**): yield: 90.1% (Method A); mp 158–160 °C; ^1^H NMR, δ = 0.92/1.05 (d, J = 6.7 Hz, 4H, cyclopropane H-2,2,3,3), 2.11–1.96 (m, 1H, cyclopropyl H-1), 2.15 (s, 3H, CH_3_), 6.30/5.93 (bs, 2H, NH_2_), 6.61–6.41 (m, 1H, H-4), 6.98/6.91 (d, J = 7.1 Hz, 1H, H-5), 7.79/7.51 (d, J = 7.9 Hz, 1H, H-3), 13.51/13.43 (br.s, 1H, NH); calculated for C_12_H_14_N_4_: C, 67.27; H, 6.59; N, 26.15; found: C, 67.26; H, 6.60; N, 26.15.

*2-(3-Cyclopropyl-1H-1,2,4-triazol-5-yl)-5-fluoroaniline* (**2.3**): yield: 97.7% (Method A); mp 242–244 °C; ^1^H NMR, δ = 1.04/0.91 (d, J = 6.7Hz, 4H, cyclopropyl H-2,2,3,3), 2.12–1.91 (m, 1H, cyclopropyl H-1), 6.33–6.16 (m, 1H, H-4), 6.53–6.33 (m, 3H, NH_2_, H-6), 6.86–6.77 (m, 1H, H-6), 7.87/7.63 (d, J = 7.8 Hz, 1H, H-3), 13.48/13.40 (br.s, 1H, NH); LC-MS, *m*/*z* = 219 [M+1]; calculated for C_11_H_11_FN_4_: C, 60.54; H, 5.08; N, 25.67; found: C, 60.51; H, 5.09; N, 25.66.

*4-Chloro-2-(3-cyclopropyl-1H-1,2,4-triazol-5-yl)aniline* (**2.4**): yield: 98.7% (Method A); mp 237–239 °C; ^1^H NMR, δ = 1.05/0.91 (d, J = 6.8 Hz, 4H, cyclopropyl H-2,2,3,3), 2.14–1.95 (m, 1H, cyclopropyl H-1), 6.28 (bs, 2H, NH_2_), 6.81–6.55 (m, 2H, H-6), 7.07–6.84 (m, 1H, H-5), 7.84/7.70 (s, 1H, H-3), 13.60/13.52 (br.s, 1H, NH); LC-MS, *m*/*z* = 235 [M+1]; calculated for C_11_H_11_ClN_4_: C, 56.30; H, 4.72; N, 23.87; found: C, 56.28; H, 4.74; N, 23.86.

*2-(3-Cyclobutyl-1H-1,2,4-triazol-5-yl)aniline* (**2.5**): yield: 77.1% (Method A); mp 147–149 °C; ^1^H NMR, δ = 2.18–1.88 (m, 2H, cyclobutyl H-3,3), 2.47–2.23 (m, 4H, H-2,2,4,4), 3.74–3.55 (m, 1H, cyclobutyl H-1), 6.27 (br. s, 2H, NH_2_), 6.54 (t, J = 7.5 Hz, 1H, H-4), 6.71 (d, J = 8.2 Hz, 1H, H-6), 7.13–6.94 (m, 1H, H-5), 7.92/7.66 (m, 1H, H-3), 13.58/13.45 (br.s, 1H, NH); LC-MS, *m*/*z* = 215 [M+1]; calculated for C_12_H_14_N_4_: C, 67.27; H, 6.59; N, 26.15; found: C, 67.26; H, 6.60; N, 26.15.

*2-(3-Cyclobutyl-1H-1,2,4-triazol-5-yl)-6-methylaniline* (**2.6**): yield: 92.2% (Method A); mp 127–129 °C; ^1^H NMR, δ = 2.12–1.90 (m, 2H, cyclobutyl H-3,3), 2.17 (s, 3H, CH_3_), 2.47–2.26 (m, 4H, cyclobutyl H-2,2,4,4), 3.82–3.49 (m, 1H, cyclobutyl H-1), 6.39/5.99 (bs, 2H, NH_2_), 6.52 (t, J = 7.6 Hz, 1H, H-4), 6.98/6.93 (m, 1H, H-5), 7.81/7.55 (m, 1H, H-3), 13.58/13.45 (bs, 1H, NH); LC-MS, *m*/*z* = 229 [M+1]; calculated for C_13_H_16_N_4_: C, 68.39; H, 7.06; N, 24.54; found: C, 68.38; H, 7.07; N, 24.55.

*2-(3-Cyclobutyl-1H-1,2,4-triazol-5-yl)-5-fluoroaniline* (**2.7**): yield: 94.8% (Method A); mp 165–167 °C; ^1^H NMR, δ = 2.17–1.86 (m, 2H, cyclobutyl H-3,3), 2.46–2.29 (m, 4H, cyclobutyl H-2,2,4,4), 3.76–3.47 (m, 1H, cyclobutyl H-1), 6.26 (d, J = 8.3 Hz, 1H, H-4), 6.61–6.33 (m, 3H, NH_2_, H-6), 7.07–6.79 (m, 1H, H-6), 7.93/7.65 (t, J = 8.0 Hz, 1H, H-3), 13.55/13.42 (bs, 1H, NH); LC-MS, *m*/*z* = 233 [M+1]; calculated for C_12_H_13_FN_4_: C, 62.06; H, 5.64; N, 24.12; found: C, 62.04; H, 5.65; N, 24.11.

*4-Chloro-2-(3-cyclobutyl-1H-1,2,4-triazol-5-yl)aniline* (**2.8**): yield: 99.3% (Method A); mp 176–178 °C; ^1^H NMR, δ = 2.21–1.86 (m, 2H, cyclobutyl H-3,3), 2.49–2.20 (m, 4H, cyclobutyl H-2,2,4,4), 3.78–3.52 (m, 1H, cyclobutyl H-1), 6.33 (bs, 2H, NH_2_), 6.72 (d, J = 8.6 Hz, 1H, H-6), 6.96 (d, J = 8.7 Hz, 1H, H-5), 7.90/7.73 (s, 1H, H-3), 13.65/13.53 (bs, 1H, NH); LC-MS, *m*/*z* = 249 [M+1]; calculated for C_12_H_13_ClN_4_: C, 57.95; H, 5.27; N, 22.53; found: C, 57.93; H, 5.29; N, 22.51.

*2-(3-Cyclopentyl-1H-1,2,4-triazol-5-yl)aniline* (**2.9**): yield: 65.7% (Method A); mp 100–102 °C; ^1^H NMR, δ = 2.19–1.55 (m, 8H, cyclopentyl H-2,2,3,3,4,4,5,5), 3 3.34–2.96 (m, 1H, cyclopentyl H-1), 6.18 (bs, 2H, NH_2_), 6.54 (t, J = 7.5 Hz, 1H, H-4), 6.79–6.65 (m, 1H, H-6), 7.15–6.85 (m, 1H, H-5), 7.92/7.63 (m, 1H, H-3), 13.55/13.42 (bs, 1H, NH); LC-MS, *m*/*z* = 229 [M+1]; calculated for C_13_H_16_N_4_: C, 68.39; H, 7.06; N, 24.54; found: C, 68.38; H, 7.08; N, 24.53.

*2-(3-Cyclopentyl-1H-1,2,4-triazol-5-yl)-6-methylaniline* (**2.10**): yield: 85.8% (Method A); mp 124–126 °C; ^1^H NMR, δ = 2.12–1.13 (m, 8H, cyclopentyl H-2,2,3,3,4,4,5,5), 2.16 (s, 3H, CH3) 3.34–3.10 (m, 1H, cyclopentyl H-1), 6.37/5.97 (s, 2H, NH_2_), 6.55–6.45 (m, 1H, H-4), 6.98/6.92 (d, J = 6.8 Hz, 1H, H-5), 7.84/7.54 (d, J = 6.8 Hz, 1H, H-3), 13.52/13.40 (s, 1H, NH); LC-MS, *m*/*z* = 243 [M+1]; calculated for C_14_H_18_N_4_: C, 69.39; H, 7.49; N, 23.12; found: C, 69.38; H, 7.50; N, 23.13.

*2-(3-Cyclopentyl-1H-1,2,4-triazol-5-yl)-5-fluoroaniline* (**2.11**): yield: 80.8% (Method A); mp 112–114 °C; ^1^H NMR, δ = 2.18–1.54 (m, 8H, cyclopentyl H-2,2,3,3,4,4,5,5), 3.20 (p, J = 8.2 Hz, 1H, cyclopentyl H-1), 6.26 (t, J = 8.5 Hz, 1H, H-4), 6.46 (d, J = 8.5 Hz, 1H, H-6), 6.72 (bs, 2H, NH_2_), 8.02–7.65 (m, 1H, H-3), 13.39 (bs, 1H, NH); LC-MS, *m*/*z* = 247 [M+1]; calculated for C_13_H_15_FN_4_: C, 63.40; H, 6.14; N, 22.75; found: C, 63.38; H, 6.15; N, 22.76.

*4-Chloro-2-(3-cyclopentyl-1H-1,2,4-triazol-5-yl)aniline* (**2.12**): yield: 96.3% (Method A); mp 161–163 °C; ^1^H NMR, δ = 2.19–1.51 (m, 8H, cyclopentyl H-2,2,3,3,4,4,5,5), 3.21 (p, J = 8.4 Hz, 1H, cyclopentyl H-1), 6.33 (bs, 2H, NH_2_), 6.72 (d, J = 8.8 Hz, 1H, H-6), 6.96 (d, J = 8.6 Hz, 1H, H-5), 7.88 (s, 1H, H-3), 13.51 (bs, 1H, NH); LC-MS, *m*/*z* = 263 [M+1]; calculated for C_13_H_15_ClN_4_: C, 59.43; H, 5.75; N, 21.32; found: C, 59.42; H, 5.76; N, 21.31.

*2-(3-Cyclohexyl-1H-1,2,4-triazol-5-yl)aniline* (**2.13**): yield: 89.3% (Method A); mp 152–154 °C; ^1^H NMR, δ = 1.78–1.22 (m, 6H, cyclohexyl H-3eq, 4eq, 5eq, 3ax, 4ax, 5ax), 1.91–1.78 (m, 2H, cyclohexyl H-2ax, 6ax), 2.10–1.95 (m, 2H, cyclohexyl H-2eq, 6eq), 2.90–2.63 (m, 1H, cyclohexyl H-1), 6.17 (bs, 1H, NH_2_), 6.63–6.42 (m, 2H, H-4, NH_2_), 6.81–6.63 (m, 1H, H-6), 7.12–6.90 (m, 1H, H-5), 7.91/7.62 (d, J = 7.9 Hz, 1H, H-3), 13.53/13.37 (bs, 1H, NH); ^13^C NMR, δ = 147.1 (aniline C-1), 130.0 (aniline C-3), 127.9 (aniline C-5), 116.2 (aniline C-4), 115.6 (aniline C-6), 98.3 (aniline C-2), 36.2 (cyclohexane C-1), 31.6 (cyclohexane C-2,6), 25.9 (cyclohexane C-3,5), 25.8 (cyclohexane C-4); calculated for C_14_H_18_N_4_: C, 69.39; H, 7.49; N, 23.12; found: C, 69.37; H, 7.51; N, 23.13.

*2-(3-Cyclohexyl-1H-1,2,4-triazol-5-yl)-6-methylaniline* (**2.14**): yield: 91.3% (Method A); mp 135–137 °C; ^1^H NMR, δ = 1.79–1.16 (m, 6H, cyclohexyl H-3eq, 4eq, 5eq, 3ax, 4ax, 5ax), 1.92–1.79 (m, 2H, cyclohexyl H-2ax, 6ax), 2.10–1.95 (m, 2H, cyclohexyl H-2eq, 6eq), 2.17 (s, 3H, CH_3_), 2.85–2.73 (m, 1H, cyclohexyl H-1), 6.20 (bs, 2H, NH_2_), 6.51 (t, J = 7.5 Hz, 1H, H-4), 6.95 (d, J = 7.2 Hz, 1H, H-5), 7.93–7.55 (m, 1H, H-3), 13.50 (bs, 1H, NH); LC-MS, *m*/*z* = 257 [M+1]; calculated for C_15_H_20_N_4_: C, 70.28; H, 7.86; N, 21.86; found: C, 70.26; H, 7.88; N, 21.85.

*2-(3-Cyclohexyl-1H-1,2,4-triazol-5-yl)-5-fluoroaniline* (**2.15**): yield: 89.0% (Method A); mp 151–153 °C; ^1^H NMR, δ = 1.77–1.20 (m, 6H, cyclohexyl H-3eq, 4eq, 5eq, 3ax, 4ax, 5ax), 1.93–1.78 (m, 2H, cyclohexyl H-2ax, 6ax), 2.14–1.93 (m, 2H, cyclohexyl H-2eq, 6eq), 2.89–2.62 (m, 1H, cyclohexyl H-1), 6.33–6.13 (m, 1H, H-4), 6.66–6.33 (m, 2H, NH_2_, H-6), 6.91 (bs, 1H, NH), 7.91/7.65 (d, J = 10.3 Hz, 1H, H-3), 13.50/13.36 (s, 1H, NH); LC-MS, *m*/*z* = 261 [M+1]; calculated for C_14_H_17_FN_4_: C, 64.60; H, 6.58; N, 21.52; found: C, 64.59; H, 6.59; N, 21.51.

*4-Chloro-2-(3-cyclohexyl-1H-1,2,4-triazol-5-yl)aniline* (**2.16**): yield: 93.9% (Method A); mp 200–202 °C; ^1^H NMR, δ = 1.77–1.16 (m, 6H, cyclohexyl H-3eq, 4eq, 5eq, 3ax, 4ax, 5ax), 1.92–1.78 (m, 2H, cyclohexyl H-2ax, 6ax), 2.15–1.92 (m, 2H, cyclohexyl H-2eq, 6eq), 2.90–2.62 (m, 1H, cyclohexyl H-1), 6.33 (bs, 2H, NH_2_), 6.71 (d, J = 8.6 Hz, 1H, H-6), 6.95 (d, J = 8.6 Hz, 1H, H-5), 7.88/7.73 (s, 1H, H-3), 13.62/13.47 (bs, 1H, NH); LC-MS, *m*/*z* = 277 [M+1]; calculated for C_14_H_17_ClN_4_: C, 60.76; H, 6.19; N, 20.24; found: C, 60.75; H, 6.21; N, 20.23.

*2-(3-(Adamantan-1-yl)-1H-1,2,4-triazol-5-yl)aniline* (**2.17**): yield: 94.1% (Method A), 73.4% (Method B); mp 150–152 °C; ^1^H NMR, δ = 1.81–1.71 (m, 6H, adamantyl-4,4,6,6,10,10), 2.09–1.98 (m, 9H, adamantyl-2,2,3,5, 7,8,8,9,9), 5.49 (bs, 2H, NH_2_), 6.90 (t, J = 7.4 Hz, 1H, H-4), 7.04 (d, J = 7.8 Hz, 1H, H-6), 7.24 (d, J = 7.2 Hz, 1H, H-5), 7.97 (d, J = 7.4 Hz, 1H, H-3); ^13^C NMR, δ = 171.5 (triazole C-5), 158.1 (triazole C-3), 141.4 (aniline C-1), 132.1 (aniline C-3), 131.4 (aniline C-5), 120.2 (aniline C-4), 119.3 (aniline C-6), 40.9 (adamantane C-2, 8, 9), 38.9 (adamantane C-6), 36.4 (adamantane C-4, 6, 10), 28.0 (adamantane C-3, 5, 7); LC-MS, *m*/*z* = 295 [M+1]; calculated for C_18_H_22_N_4_: C, 73.44; H, 7.53; N, 19.03; found: C, 73.42; H, 7.55; N, 19.05.

*2-(3-(Adamantan-1-yl)-1H-1,2,4-triazol-5-yl)-6-methylaniline* (**2.18**): yield: 96.3% (Method A); mp 197–199 °C; ^1^H NMR, δ = 1.91–1.70 (m, 6H, adamantyl-4,4,6,6,10,10), 2.14–1.94 (m, 9H, adamantyl-2,2,3,5, 7,8,8,9,9), 2.17 (s, 3H, adamantyl-CH_3_), 6.20 (bs, 2H, NH_2_), 6.52 (t, J = 7.5 Hz, 1H, H-4), 6.95 (d, J = 7.2 Hz, 1H, H-5), 7.75 (d, J = 7.5 Hz, 1H, H-3), 13.45 (br.s, 1H, NH); LC-MS, m/z = 309 [M+1]; calculated for C_19_H_24_N_4_: C, 73.99; H, 7.84; N, 18.17; found: C, 73.98; H, 7.85; N, 18.17.

*2-(3-(Adamantan-1-yl)-1H-1,2,4-triazol-5-yl)-5-fluoroaniline* (**2.19**): yield: 97.9% (Method A); mp 274–276 °C; ^1^H NMR, δ = 1.92–1.68 (m, 6H, adamantyl-4,4,6,6,10,10), 2.21–1.92 (m, 9H, adamantyl-2,2,3,5, 7,8,8,9,9), 6.23 (d, J = 8.7 Hz, 1H, H-4), 6.94–6.33 (m, 3H, NH_2_, H-6), 8.02–7.73 (m, 1H, H-3), 13.33 (s, 1H, NH); LC-MS, *m*/*z* = 313 [M+1]; calculated for C_18_H_21_FN_4_: C, 69.21; H, 6.78; N, 17.94; found: C, 69.20; H, 6.79; N, 17.94.

*2-(3-(Adamantan-1-yl)-1H-1,2,4-triazol-5-yl)-4-chloroaniline* (**2.20**): yield: 96.4% (Method A); mp 243–245 °C; ^1^H NMR, δ = 1.90–1.66 (m, 6H, adamantyl-4,4,6,6,10,10), 2.20–1.91 (m, 9H, adamantyl-2,2,3,5, 7,8,8,9,9), 6.32 (bs, 2H, NH_2_), 6.71 (d, J = 8.9 Hz, 1H, H-6), 6.95 (d, J = 8.6 Hz, 1H, H-5), 7.90/7.73 (s, 1H, H-3), 13.61/13.46 (s, 1H, NH); LC-MS, *m*/*z* = 329 [M+1]; calculated for C_18_H_21_ClN_4_: C, 65.74; H, 6.44; N, 17.04; found: C, 65.73; H, 6.44; N, 17.03.

*2-(3-(Adamantan-1-yl)-1H-1,2,4-triazol-5-yl)-4-bromoaniline* (**2.21**): yield: 88.9% (Method A); mp 249–251 °C; ^1^H NMR, δ = 1.89–1.69 (m, 6H, adamantyl-4,4,6,6,10,10), 2.22–1.92 (m, 9H, adamantyl-2,2,3,5, 7,8,8,9,9), 6.36 (br.s, 2H, NH_2_), 6.68 (d, J = 8.7 Hz, 1H, H-6), 7.07 (d, J = 8.6 Hz, 1H, H-5), 8.01 (s, 1H, H-3), 13.45 (bs, 1H, NH); LC-MS, *m*/*z* = 373 [M+1]; calculated for C_18_H_21_BrN_4_: C, 57.92; H, 5.67; N, 15.01; found: C, 57.90; H, 5.69; N, 15.03.

*2-(3-Phenyl-1H-1,2,4-triazol-5-yl)aniline* (**2.22**): yield: 96.9% (Method A), 94.9% (Method B); mp 189–191 °C; ^1^H NMR, δ = 6.63 (t, J = 7.4 Hz, 1H, H-4), 6.72 (br s, 2H, NH_2_), 6.83 (d, J = 7.7 Hz, 1H, H-6), 7.14 (t, J = 7.5, 1H, H-5), 7.49 (m, 3H, 3-Ar H-3,4,5), 7.78 (d, J = 7.7 Hz, 1H, H-3), 8.09 (d, J = 7.0 Hz, 2H, 3-Ar H-2,6), 14.48/14.20 (br.s, 1H, NH); ^13^C NMR, δ = 160.7 (triazole C-3), 154.1 (triazole C-5), 147.4 (aniline C-1), 131.4 (phenyl C-1, 3, 4, 5), 129.2 (aniline C-5), 127.4 (phenyl C-2,6), 126.4 (aniline C-3), 116.5 (aniline C-4), 115.2 (aniline C-6), 108.9 (aniline C-2); LC-MS, *m*/*z* = 237 [M+1]; calculated for C_14_H_12_N_4_: C, 71.17; H, 5.12; N, 23.71; found: C, 71.23; H, 5.19; N, 23.75.

*2-(3-(4-Fluorophenyl)-1H-1,2,4-triazol-5-yl)aniline* (**2.23**): yield: 93.6% (Method A), 90.6% (Method B); mp 209–211 °C; ^1^H NMR, δ = 6.64 (t, J = 7.0 Hz, 1H, H-4), 6.85 (d, J = 7.8 Hz, 1H, H-6), 7.15 (t, J = 7.3 Hz, 1H, H-5), 7.34 (t, J = 7.7 Hz, 2H, 3-Ar H-3,5), 7.92–7.75 (d, J = 7.3 Hz, 1H, H-3), 8.13 (t, J = 6.7 Hz, 2H, 3-Ar H-2,6), 14.36 (bs, 1H, NH); ^13^C NMR, δ = 162.7 (d, J = 253.4 Hz, phenyl C-4), 159.4 (triazole C-3), 155.6 (triazole C-5), 146.7 (aniline C-1), 130.5 (phenyl C-1), 130.0 (aniline C-5), 128.0 (d, J = 7.7 Hz, phenyl C-2,6), 127.2 (aniline C-3), 116.1 (aniline C-4), 115.7 (d, J = 22.3 Hz, phenyl C-3,5), 115.2 (aniline C-6), 108.0 (aniline C-2); LC-MS, *m*/*z* = 255 [M+1]; calculated for C_14_H_11_FN_4_: C, 66.13; H, 4.36; N, 22.03; found: C, 66.17; H, 4.41; N, 22.27.

*2-(3-(4-Chlorophenyl)-1H-1,2,4-triazol-5-yl)aniline* (**2.24**): yield: 92.6% (Method A), 93.2% (Method B); mp 289–291 °C; ^1^H NMR, δ = 6.99–6.51 (m, 3H, H-4, NH_2_), 7.16 (m, 1H. H-6), 7.57 (d, J = 6.3 Hz, 1H, H-5), 7.98–7.77 (m, 2H, 3-Ar H-3,5), 8.11 (d, J = 7.0 Hz, 1H, H-3), 8.30 (d, J = 7.1 Hz, 2H, 3-Ar H-2,6; ^13^C NMR, δ = 161.9 (triazole C-3), 157.5 (triazole C-5), 146.9 (aniline C-1), 134.1 (phenyl C-4), 130.5 (aniline C-5), 128.8 (phenyl C-3,5), 126.4 (phenyl C-2,6), 125.7 (aniline C-3), 116.0 (aniline C-4), 115.1 (aniline C-6), 108.7 (aniline C-2); LC-MS, *m*/*z* = 271 [M+1]; calculated for C_14_H_11_ClN_4_: C, 62.11; H, 4.10; N, 20.70; found: C, 62.19; H, 4.16; N, 20.77.

*2-(3-(4-Bromophenyl)-1H-1,2,4-triazol-5-yl)aniline* (**2.25**): yield: 95.5% (Method A), 96.2% (Method B); mp 216–218 °C; ^1^H NMR, δ = 6.63 (t, J = 7.3 Hz, 1H, H-4); 6.85 (d, J = 8.0 Hz, 1H, H-6), 7.15 (t, J = 7.3 Hz, 1H, H-5), 7.62 (d, J = 8.0 Hz, 1H, H-3), 7.66 (d, J = 7.9 Hz, 2H, 3-Ar H-3,5), 7.85 (d, J = 8.0 Hz, 2H, 3-Ar H-2,6); ^13^C NMR, δ = 162.6 (triazol C-3), 158.8 (triazol C-5), 146.7 (aniline C-1), 131.5 (phenyl C-3,5), 131.1 (phenyl C-2,6), 130.9 (aniline C-5), 129.8 (phenyl C-1), 124.8 (aniline C-3), 122.0 (phenyl C-4), 116.1 (aniline C-4), 115.2 (aniline C-6), 110.9 (aniline C-2); LC-MS, *m*/*z* = 316 [M+1]; calculated for C_14_H_11_BrN_4_: C, 53.35; H, 3.52; N, 17.78; found: C, 53.41; H, 3.57; N, 17.82.

*2-(3-(2-Fluorohenyl)-1H-1,2,4-triazol-5-yl)aniline* (**2.26**): yield: 91.3% (Method A), 89.6% (Method B); mp 195–197 °C; ^1^H NMR, δ = 6.65 (t, J = 7.3 Hz, 1H, H-4), 6.75 (bs, 2H, NH_2_), 6.85 (d, J = 8.1 Hz, 1H, H-6), 7.15 (t, J = 7.2 Hz, 1H, H-5), 7.41–7,30 (m, 2H, 3-Ar H-3, 5), 7.55–7.46 (m, 1H, 3-Ar H-4), 7.93–7.81 (m, 1H, H-3), 8.11 (t, J = 7.3 Hz, 1H, 3-Ar H-6), 14.36 (bs, 1H, NH); ^13^C NMR, δ = 162.8 (triazol C-3), 159.3 (d, J = 253.2 Hz, phenyl C-2), 154.7 (triazol C-5), 146.8 (aniline C-1), 131.1 (aniline C-5), 130.3 (phenyl C-4), 129.7 (d, J = 2.6 Hz, phenyl C-5), 127.2 (phenyl C-6), 124.6 (aniline C-3), 116.4 (d, J = 21.2 Hz, phenyl C3), 116.0 (aniline C-4), 115.2 (aniline C-6), 109.3 (aniline C-2); LC-MS, *m*/*z* = 255 [M+1]; calculated for C_14_H_11_FN_4_: C, 66.13; H, 4.36; N, 22.03; found: C, 66.19; H, 4.39; N, 22.17.

*2-(3-(Furan-2-yl)-1H-1,2,4-triazol-5-yl)aniline* (**2.27**): yield: 86.3% (Method A), 73.10% (Method B); mp 206–208 °C; ^1^H NMR, δ = 6.71–6.61 (m, 2H, H-4, furan, H-4), 6.85 (d, 1H, J = 8.2 Hz, H-6), 7.10–6.99 (m, 1H, furan, H-3), 7.17 (t, 1H, J = 8.2 Hz, H-5), 7.94–7.80 (m, 2H, H-6, furan H-3); calculated for C_12_H_10_N_4_O: C, 63.71; H, 4.46; N, 24.76; found: C, 63.70; H, 4.47; N, 24.75.

*2-(3-(Furan-3-yl)-1H-1,2,4-triazol-5-yl)-6-methylaniline* (**2.28**): yield: 72.25% (Method B); mp 182–184 °C; ^1^H NMR, δ = 2.19 (s, 3H, CH3), 6.42/6.02 (br. s, 2H, NH_2_), 6.55 (t, J = 7.2 Hz, 1H, H-4), 7.09–6.81 (m, 2H, H-5, furan, H-4), 7.73–7.50 (m, 2H, H-3, furan, H-5), 7.91 (d, J = 7.9 Hz, 1H, H-3), 8.25/8,03 (bs, 1H, furan, H-2), 14.07/13.93 (s, 1H, NH); LC-MS, m/z = 241 [M+1]; calculated for C_13_H_12_N_4_O: C, 64.99; H, 5.03; N, 23.32; found: C, 64.97; H, 23.33.

*5-Fluoro-2-(3-(furan-3-yl)-1H-1,2,4-triazol-5-yl)aniline* (**2.29**): yield: 79.98% (Method A); mp 204–206 °C; ^1^H NMR, δ = 6.66–6.15 (m, 3H, H-4, NH_2_), 7.11–6.79 (m, 2H, H-6, furan H-4), 7.64/7.57 (m, 1H, H-5), 7.99/7.72 (t, J = 7.8 Hz, 1H, H-3), 8.21/8.08 (s, 1H, furan, H-2), 14.04/13.90 (s, 1H, NH); LC-MS, *m*/*z* = 245 [M+1]; calculated for C_12_H_9_FN_4_O: C, 59.02; H, 3.71; N, 22.94; found: C, 59.00; H, 3.71; N, 22.95.

*4-Chloro-2-(3-(furan-3-yl)-1H-1,2,4-triazol-5-yl)aniline* (**2.30**): yield: 77.99% (Method A); mp 248–250 °C; ^1^H NMR, δ = 6.38 (s, 2H, NH_2_), 6.85–6.70 (m, 1H, H-6), 7.10–6.86 (m, 2H, H-5, furan, H-4), 7.66/7.58 (m, 1H, furan, H-5), 7.96/7.79 (s, 1H, H-3), 8.24/8.08 (m, 1H, furan H-2), 14.15/14.01 (s, 1H, NH); LC-MS, *m*/*z* = 261 [M+1]; anal. calcd. for C_12_H_9_ClN_4_O: C, 55.29; H, 3.48; N, 21.49; found: C, 55.27; H, 3.49; N, 21.50.

*4-Bromo-2-(3-(furan-3-yl)-1H-1,2,4-triazol-5-yl)aniline* (**2.31**): yield: 56.00% (Method A); mp 228–230 °C; ^1^H NMR, δ = 6.51 (s, 2H, NH_2_), 6.73 (d, J = 8.8 Hz, 1H, H-6), 7.06–6.84 (m, 1H, H-5), 7.12 (d, J = 8.2 Hz, 1H, furan H-4), 7.63 (d, J = 7.8 Hz, 1H, furan, H-5), 8.37–7.86 (m, 2H, H-3, furan H-2), 14.18 (s, 1H, NH); calculated for C_12_H_9_BrN_4_O: C, 47.24; H, 2.97; N, 18.36; found: C, 47.21; H, 2.98; N, 18.37.

*2-(3-(Thiophen-2-yl)-1H-1,2,4-triazol-5-yl)aniline* (**2.32**): yield: 98.63% (Method A), 92.3% (Method B); mp 189–191 °C; ^1^H NMR, δ = 6.65 (t, 1H, J = 8.3 Hz, H-4), 6.85 (d, 1H, J = 8.3 Hz, H-6), 7.19 (m, 2H, H-4, thiophen, H-5), 7.66 (d, 1H, thiophen, H-3), 7.72 (d, 1H, thiophen, H-5), 7,81 (d, 1H, H-3); ^13^C NMR, δ = 147.6 (aniline C-1), 134.4 (thiophen C-2), 131.5 (aniline C-3), 128.4 (aniline C-5), 127.4 (thiophen C-5), 127.3 (thiophen C-4), 126.3 (thiophen C-3), 116.8 (aniline C-4), 115.7 (aniline C-6), 107.9 (aniline C-2); LC-MS, *m*/*z* = 243 [M+1]; calculated for C_12_H_10_N_4_S: C, 59.48; H, 4.16; N, 23.12; found: C, 59.46; H, 4.18; N, 20.85.

*5-Fluoro-2-(3-(thiophen-2-yl)-1H-1,2,4-triazol-5-yl)aniline* (**2.33**): yield: 96.76% (Method A); mp 233–235 °C; 1H NMR, δ = 6.31 (bs, 2H, NH_2_), 6.69–6.42 (m, 1H, H-4), 7.01–6.81 (m, 1H, H-6), 7.19–7.05 (m, ^1^H, thiophene, H-4), 7.47–7.27 (m, 1H, thiophene, H-3), 7.63/7.56 (m, 1H, thiophene, H-5), 8.01/7.73 (m, 1H, H-3), 14.26/13.99 (s, 1H, NH); LC-MS, *m*/*z* = 261 [M+1]; calculated for C_12_H_9_FN_4_S: C, 55.37; H, 3.49; N, 21.53; found: C, 55.35; H, 3.49; N, 21.50.

*6-Methyl-2-(3-(thiophen-3-yl)-1H-1,2,4-triazol-5-yl)aniline* (**2.34**): yield: 98.75% (Method A); mp 168–170 °C; ^1^H NMR, δ = 2.20 (s, 3H, CH_3_), 6.35 (s, 2H, NH_2_), 6.56 (t, J = 7.4 Hz, 1H, H-4), 7.00 (d, J = 5.6 Hz, 1H, thiophene H-4), 7.59–7.42 (m, 1H, H-5), 7.78 -7.60 (m, 2H, H-2, 5 thiophene), 8.09–7.80 (bs, 3H, H-3), 13.97 (s, 1H, NH); LC-MS, *m*/*z* = 257 [M+1]; calculated for C_13_H_12_N_4_S: C, 60.92; H, 4.72; N, 21.86; found: C, 60.91; H, 4.74; N, 21.86.

*5-Fluoro-2-(3-(thiophen-3-yl)-1H-1,2,4-triazol-5-yl)aniline* (**2.35**): yield: 80.18% (Method A); mp 212–214 °C; ^1^H NMR, δ = 6.30 (bs, 1H, NH2), 6.69–6.41 (m, 2H, H-4, NH), 7.11–6.92 (m, 1H, H-6), 7.66–7.33 (m, 2H, H-4.5 thiophene), 8.01/7.71 (m, 1H, H-3), 8.11/7.93 (m, 1H, H-2 thiophene), 14.12/13.91 (s, 1H, NH); LC-MS, *m*/*z* = 261 [M+1]; calculated for C_12_H_9_FN_4_S: C, 55.37; H, 3.49; N, 21.53; found: C, 55.35; H, 3.52; N, 21.54.

*4-Chloro-2-(3-(thiophen-3-yl)-1H-1,2,4-triazol-5-yl)aniline* (**2.36**): yield: 75.28% (Method A); mp 240–242 °C; ^1^H NMR, δ = 6.41 (bs, 2H, NH_2_), 6.90–6.66 (m, 1H, H-6), 7.10–6.92 (m, 1H, H-5), 7.55/7.46 (m, 1H, H-4 thiophene), 7.71/7.64 (m, 1H, H-5 thiophene), 7.97 (s, 1H, H-3), 8.14 (s, 1H, H-2 thiophene), 14.24/14.03 (s, 1H, NH); LC-MS, *m*/*z* = 277 [M+1]; calculated for C_12_H_9_ClN_4_S: C, 52.08; H, 3.28; N, 20.25; found: C, 52.06; H, 3.30; N, 20.26.

*4-Bromo-2-(3-(thiophen-3-yl)-1H-1,2,4-triazol-5-yl)aniline* (**2.37**): yield: 77.13% (Method A); mp 235–236 °C; ^1^H NMR, δ = 6.44 (s, 2H, NH_2_), 6.97–6.60 (m, 1H, H-6), 7.24–7.02 (m, 1H, H-5), 7.55/7.46 (m, 1H, H-4 thiophene), 7.71/7.63 (m, 1H, H-5 thiophene), 7.94 (s, 1H, H-3), 8.12 (s, 1H, H-2 thiophene), 14.23/14.02 (s, 1H, NH); LC-MS, *m*/*z* = 322 [M+1]; calculated for C_12_H_9_BrN_4_S: C, 44.87; H, 2.82; N, 17.44; found: C, 44.86; H, 2.85; N, 17.45.

*2-(3-(Benzofuran-2-yl)-1H-1,2,4-triazol-5-yl)aniline* (**2.38**): yield: 65.21% (Method A), 71.3% (Method B); mp 235–237 °C; ^1^H NMR, δ = 5.45 (bs, 2H, NH_2_), 6.71 (t, 1H, J = 8.3 Hz, H-4), 6.90 (d, 1H, J = 8.3 Hz, H-6), 7.45–7.12 (m, 3H, H-5, benzofuran, H-5, H-6), 7.56 (s, 1H, benzofuran, H-3), 7.76–7.64 (m, benzofuran, H-4,7), 7.91 (d, J = 7.1 Hz, 1H, H-3); ^13^C NMR, δ = 158.4 (triazole C-5), 154.9 (benzofuran C-7a), 154.7 (triazole C-3), 148.9 (benzofuran C-2), 147.6 (aniline C-1), 131.5 (aniline C-3), 128.4 (aniline C-5), 127.8 (benzofuran C-3a), 124.1 (benzofuran C-6), 123.1 (benzofuran C-5), 122.2 (benzofuran C-4), 116.7 (aniline C-4), 115.8 (aniline C-6), 111.9 (benzofuran C-7), 109.2 (benzofuran C-3), 106.0 (aniline C-2); LC-MS, *m*/*z* = 277 [M+1]; calculated for C_16_H_12_N_4_O: C, 69.55; H, 4.38; N, 20.28; found: C, 69.55; H, 4.40; N, 20.29.

*2-(3-(Benzofuran-2-yl)-1H-1,2,4-triazol-5-yl)-6-methylaniline* (**2.39**): yield: 92.50% (Method A); mp 200–201 °C; ^1^H NMR, δ = 2.21 (s, 3H, CH_3_), 6.57 (m, 3H, H-4, NH_2_), 7.05 (d, J = 7.2 Hz, 1H, H-5), 7.48–7.16 (m, 3H, H-3, 5, 6 benzofuran), 7.84–7.48 (m, 3H, H-3, benzofuran, H-4,7), 14.72/14.31 (s, 1H, NH); LC-MS, *m*/*z* = 291 [M+1]; anal. calcd. for C_17_H_14_N_4_O: C, 70.33; H, 4.86; N, 19.30; found: C, 70.30; H, 4.88; N, 19.31.

*2-(3-(Benzofuran-2-yl)-1H-1,2,4-triazol-5-yl)-5-fluoroaniline* (**2.40**): yield: 50.78% (Method A); mp 251–253 °C; ^1^H NMR, δ = 6.32 (s, 2H, NH_2_), 6.55 (t, J = 8.0 Hz, 1H, H-4), 7.09–6.99 (m, 1H, H-6), 7.44–7.12 (m, 3H, H-3,5,6 benzofuran), 8.07–7.88 (m, 2H, H-4,7 benzofuran), 8.17 (dt, J = 8.9, Hz, 1H, H-3), 12.21 (s, 1H, NH); LC-MS, *m*/*z* = 295 [M+1]; calculated for C_16_H_11_FN_4_O: C, 65.30; H, 3.77; N, 19.04; found: C, 65.29; H, 3.79; N, 19.05.

*2-(3-(Benzofuran-2-yl)-1H-1,2,4-triazol-5-yl)-4-chloroaniline* (**2.41**): yield: 75.72% (Method A); mp 245–246 °C; ^1^H NMR, δ = 6.43 (bs, 2H, NH_2_), 6.88–6.74 (m 1H, H-6), 7.13–6.93 (m, 1H, H-5), 7.46–7.18 (m, 3H, H-3,5,6 benzofuran), 7.72–7.50 (m, 2H, H-4,7 benzofuran), 7.84 (s, 1H, H-3), 14.81/14.42 (s, 1H, NH); LC-MS, *m*/*z* = 311 [M+1]; anal. calcd. for C_16_H_11_ClN_4_O: C, 61.84; H, 3.57; N, 18.03; found: C, 61.84; H, 3.59; N, 18.04.

*2-(3-(Benzofuran-2-yl)-1H-1,2,4-triazol-5-yl)-4-bromoaniline* (**2.42**): yield: 90.58% (Method A); mp 224–226 °C; ^1^H NMR, δ = 6.47 (bs, 2H, NH_2_), 6.84–6.66 (d, 1H, H-6), 6.97–6.81 (m, 1H, H-5), 7.20–7.04 (m, 1H, H-5 benzofuran), 7.29–7.22 (t, 1H, H-6 benzofuran), 7.38–7.28 (m, 1H, H-3 benzofuran), 7.87–7.50 (m, 2H, H-4,7 benzofuran), 7.98 (s, 1H, H-3), 14.80/14.39 (s, 1H, NH); LC-MS, *m*/*z* = 356 [M+1]; calculated for C_16_H_11_BrN_4_O: C, 54.10; H, 3.12; N, 15.77; found: C, 54.08; H, 3.14; N, 15.78.

*2-(3-(Benzo[b]thiophen-2-yl)-1H-1,2,4-triazol-5-yl)aniline* (**2.43**): yield: 98.6% (Method A); mp 192–196 °C; ^1^H NMR, δ = 6.70 (t, 1H, J = 8.3 Hz, H-5), 6.90 (d, 1H, J = 8.3 Hz, H-3), 7.21 (t, 1H, J = 8.3 Hz, H-4), 7.44 (m, 2H, H-5 H6 benzo[b]thiophen), 7.83 (d, 1H, J = 8,3 Hz, H-6), 7.97 (d, J = 5.0 Hz, 1H, H-4 benzo[b]thiophen ), 8,03 (d, J = 5.0 Hz, 1H, H-7 benzo[b]thiophen), 8,08 (s, 1H, H-3 benzo[b]thiophen); LC-MS, *m*/*z* = 293 [M+1]; calculated for C_16_H_12_N_4_S: C, 65.73; H, 4.14; N, 19.16; found: C, 65.78; H, 4.19; N, 19.22.

*2-(3-(1H-Indol-2-yl)-1H-1,2,4-triazol-5-yl)aniline* (2.44): yield: 61.70% (Method A); mp 242–244 °C; ^1^H NMR, δ = 6.70 (t, J = 8.1 Hz, 1H, H-5), 6.91 (d, J = 8.1 Hz, 1H, H-3), 7.07 (m, 2H, indol, H-5, H-6), 7.23 (m, 2H, indol, H-3, H-4), 7.48 (d, 1H, indol, H-4), 7.62 (d, 1H, indol, H-7), 7.89 (d, 1H, J = 7.8 Hz, H-6), 11.86 (s, 1H, NH), 12.14 (s, 1H, NH); LC-MS, *m*/*z* = 276 [M+1]; calculated for C_16_H_13_N_5_: C, 69.80; H, 4.76; N, 25.44; found: C, 69.78; H, 4.77; N, 25.45.

*2-(3-(Pyridin-2-yl)-1H-1,2,4-triazol-5-yl)aniline* (**2.45**): yield: 86.21% (Method A); mp 185–186 °C; ^1^H NMR, δ = 6.28 (s, 2H, NH_2_), 6.59 (t, J = 7.5 Hz, 1H, H-4), 6.75 (m, 1H, H-6), 7.05 (t, J = 7.8 Hz, H-5), 7.46/7.35 (m, 1H, H-3), 7.95 (t, J = 7.7 Hz, 1H, pyridine, H-5), 8.04 (d, J = 7.8 Hz, 1H, pyridine, H-3), 8.23 (d, J = 7.8 Hz, 1H, pyridine, H-6), 8.69 (m, 1H, pyridine, H-4), 14.58/14.23 (s, 1H, NH); ^13^C NMR, δ = 162.8 (triazole C-5), 153.7 (triazole C-3), 150.1 (pyridine C-6), 147.1 (aniline C-1), 146.6 (pyridine C-2), 138.4 (pyridine C-4), 131.5 (aniline C-3), 128.5 (aniline C-5), 125.7 (pyridine C-5), 122.0 (pyridine C-3), 116.7 (aniline C-4), 115.8 (aniline C-6), 112.9 (aniline C-2); LC-MS, *m*/*z* = 238 [M+1]; calculated for C_13_H_11_N_5_: C, 65.81; H, 4.67; N, 29.52; found: C, 65.82; H, 4.68; N, 29.54.

*2-(3-(Pyridin-3-yl)-1H-1,2,4-triazol-5-yl)aniline* (**2.46**): yield: 81.3% (Method A), 90.3% (Method B); mp 245–246 °C; ^1^H NMR, δ = 6.59 (t, J = 7.4 Hz, 1H, H-4), 6.67 (s, 1H, NH_2_), 6.81 (d, J = 8.2 Hz, 1H, H-6), 7.11 (t, J = 7.6 Hz, 1H, H-5), 7.41 (t, J = 6.3, Hz, 1H, pyridine, H-5), 7.72 (d, J = 7.5 Hz, 1H, H-3), 8.39 (d, J = 8.2 Hz, 1H, pyridine, H-4), 8.57 (d, J = 8.4 Hz, 1H, pyridine, H-6), 9.26 (s, 1H, pyridine, H-2), 14.51/14.20 (s, 1H, NH); ^13^C NMR, δ = 150.5 (pyridine C-6), 147.6 (pyridine C-2), 147.5 (aniline C-1), 133.7 (pyridine C-4), 131.1 (aniline C-3), 127.7 (aniline C-5), 126.8 (pyridine C-3), 124.4 (pyridine C-5), 116.6 (aniline C-4), 115.7 (aniline C-6), 109.4 (aniline C-2); LC-MS, *m*/*z* = 238 [M+1]; calculated for C_13_H_11_N_5_: C, 65.81; H, 4.67; N, 29.52; found: C, 65.80; H, 4.68; N, 29.55.

*2-(3-(Pyridin-4-yl)-1H-1,2,4-triazol-5-yl)aniline* (**2.47**): yield: 88.93% (Method A), 91.8% (Method B); mp 261–263 °C; ^1^H NMR, δ = 6.69–6.52 (m, 3H, H-4, NH_2_), 6.81 (d, J = 8.2 Hz, 1H, H-6), 7.11 (t, J = 7.8 Hz, 1H, H-5), 7.83–7.70 (d, 1H, H-3), 7.99 (d, J = 5.0 Hz, 2H, pyridine, H-3,5), 8.63 (d, J = 5.1 Hz, 2H, pyridine, H-2,6), 14.35 (s, 1H, NH); ^13^C NMR, δ = 150.9 (pyridine C-3,5), 147.7 (aniline C-1), 131.4 (aniline C-3), 127.6 (aniline C-5), 120.6 (pyridine C-2,6), 116.8 (aniline C-4), 115.7 (aniline C-6); LC-MS, *m*/*z* = 238 [M+1]; calculated for C_13_H_11_N_5_: C, 65.81; H, 4.67; N, 29.52; found: C, 65.78; H, 4.69; N, 29.54.

*4-Bromo-2-(3-(pyridin-4-yl)-1H-1,2,4-triazol-5-yl)aniline* (**2.48**): yield: 81.82% (Method A); mp 277–279 °C; ^1^H NMR, δ = 6.88–6.65 (m, 3H, H-6, NH_2_), 7.17 (t, J = 8.8 Hz, 1H, H-4), 7.99 (m, 3H, H-3, pyridine, H-3,5), 8.64 (m, 2H, pyridine, H-2.6), 14.50 (s, 1H, NH); LC-MS, *m*/*z* = 317 [M+1]; calculated for C_13_H_10_BrN_5_: C, 49.39; H, 3.19; N, 22.15; found: C, 49.44; H, 3.23; N, 22.18.

### 3.2. X-Ray Crystallographic Study of 2-(3-Cyclopropyl-1H-1,2,4-triazol-5-yl)aniline (**2.1**)

The yellow crystals of compound **2.1** (C_11_H_12_N_4_) are orthorhombic at 173 K a = 9.6093(7), b = 19.1643(12), c = 5.3530(4) Å, V = 985.78(12) Å3, Mr = 200.25, Z = 4, space group Pna21, dcalc = 1.349 g/cm^3^, μ(MoK*α*) = 0.086 mm^−1^ and F(000) = 424. Intensities of 13,137 reflections (1742 independent, Rint = 0.078) were measured on the Bruker APEX II diffractometer (Billerica, MA, USA) (graphite-monochromated MoKα radiation, CCD detector, φ- and ω-scanning, 2Θmax = 50^o^). The structure was solved by the direct method using the SHELXTL package [50]. Positions of the hydrogen atoms were located from electron density difference maps and refined using the “riding” model with unrestricted Uiso. Full-matrix least-squares refinement against F2 in anisotropic approximation for non-hydrogen atoms using 1742 reflections was converged to wR2 = 0.1053 (R1 = 0.0511 for 1420 reflections with F > 4σ(F), S = 1.094). The final atomic coordinates and crystallographic data for molecule **2.1** have been deposited at the Cambridge Crystallographic Data Centre, 12 Union Road, CB2 1EZ, UK (fax: +44-1223-336033; e-mail: deposit@ccdc.cam.ac.uk), and are available upon request quoting the deposition number CCDC 2352489.

### 3.3. Molecular Docking

This research was carried out using flexible molecular docking as a method to identify molecules with affinities for specific biological targets. Macromolecules from the Protein Data Bank (PDB) were selected as biological targets, specifically DNA gyrase (PDB ID: 2XCT) [51]. The selection of these biological targets was based on literature data regarding the mechanism of antistaphylococcal activity [16,17].

#### 3.3.1. Ligand Preparation

The compounds were drawn using MarvinSketch 20.21.0 and saved in mol format [52]. Subsequently, they were optimized using Chem3D with the molecular mechanics MM2 algorithm and saved as PDB files. The application of molecular mechanics allowed for the generation of more accurate geometries for organic molecules due to its extensive parameterization. Using AutoDockTools-1.5.6, the PDB files were converted to PDBQT format, and the number of active torsions was set to the default value [53].

#### 3.3.2. Protein Preparation

PDB files were obtained from the Protein Data Bank. Water molecules and ligands were removed from the structures using Discovery Studio v21.1.0.20298, and the resulting protein structures were saved as PDB files [54]. Polar hydrogens were then added in AutoDockTools-1.5.6, and the files were saved in PDBQT format. The grid box was set as follows: center_x = −12.436; center_y = 34.791; center_z = 67.712; size_x = 8; size_y = 8; size_z = 12. Vina was used to carry out the docking [51]. For visualization, Discovery Studio v21.1.0.20298 was used.

To validate the docking method, the root-mean-squared deviation (RMSD) value was used as a measure to assess the reliability of the docking process. The reference ligand was first extracted and subsequently redocked into the binding site [55]. A docking attempt is generally considered successful if the resulting pose exhibits an RMSD value of less than 2 Å compared to the X-ray crystallographic conformation [56]. The RSMD value between the experimental and reference conformation ligand was calculated to be 1.268 Å via DockRMSD, available online. [57] Therefore, the studies are considered reliable.

### 3.4. Antimicrobial Activity

The sensitivity of microorganisms to the synthesized compounds was assessed using previously described methods [58]. The assay was performed on Mueller–Hinton agar using a two-fold serial dilution of the compound in 1 mL, followed by the addition of 0.1 mL of microbial inoculum (150 × 10⁶ CFU/mL, where CFU stands for colony-forming units). The minimal inhibitory concentration (MIC) of the compound was defined as the lowest concentration at which no visible microbial growth was observed in the test tube. The minimal bactericidal concentration (MBC) was determined by the absence of microbial growth on agar medium after transferring samples from transparent test tubes. DMSO was employed as the solvent, with an initial solution concentration of 1 mg/mL. *Staphylococcus aureus ATCC 25923* served as the test strain for the preliminary evaluation of antibacterial activity. Additional quality control of the culture media and solvents was conducted following standard procedures [58]. To account for the molar mass of the respective derivatives, the MIC and MBC values were expressed in molar concentrations (μM). The *Staphylococcus aureus ATCC 25923* test strain was obtained from the bacteriological laboratory at the Zaporizhzhia Regional Laboratory Center of the State Sanitary and Epidemiological Service of Ukraine.

### 3.5. SwissADME Analysis

The SwissADME virtual laboratory was utilized to calculate the physicochemical descriptors and predict the ADME parameters, pharmacokinetic properties and drug-likeness. The fundamental approaches and methodology underlying SwissADME, a free web-based tool designed for evaluating pharmacokinetics and drug-likeness, are detailed in the scientific literature [59,60,61].

## 4. Conclusions

This work presents a “one-pot” synthesis of 48 compounds of [2-(3-R-1*H*-[1,2,4]-triazol-5-yl)phenyl]amines, which was based on the transformation of substituted 4-hydrazinoquinazolines or substituted 2-aminobenzonitriles and carboxylic acid derivatives to the target products. The reactions showed regioselectivity and good reproducibility, producing the desired products in high yields. The evaluation of the synthesized compounds by the in silico and in vitro methodology made it possible to identify promising antibacterial agents against *S. aureus ATCC 25923*, which compete with the reference drug, “Ciprofloxacin”. The research results indicate the significant dependence of antibacterial activity on the “pharmacophore” fragment at the third position of 1,2,4-triazole and substituents in the aniline fragment of the molecule. Thus, the structure–activity relationship requires additional studies on this series of compounds. Summarizing the above screening results, we can say that the synthesized [2-(3-R-1*H*-[1,2,4]-triazol-5-yl)phenyl]amines deserve further structural modification and detailed study as effective antistaphylococcal agents. Further studies on the obtained compounds’ antibacterial activity against standard and clinically isolated MRSA strains will allow for the enhancement of their potential as promising antistaphylococcal agents.

## Figures and Tables

**Table 1:** The results of studies on the docking of ligand **2** and the native inhibitor to the active site of DNA gyrase (2XCT).

Compound	Affinity (kcal/mol)	Compound	Affinity (kcal/mol)	Compound	Affinity (kcal/mol)
TA *	−6.3	**2.17**	−5.5	**2.34**	−8.1
**2.1**	−6.7	**2.18**	−5.6	**2.35**	−7.9
**2.2**	−7.4	**2.19**	−6.1	**2.36**	−8.3
**2.3**	−8.4	**2.20**	−6.3	**2.37**	−8.3
**2.4**	−7.1	**2.21**	−6.0	**2.38**	−8.9
**2.5**	−7.1	**2.22**	−7.9	**2.39**	−8.5
**2.6**	−7.6	**2.23**	−8.3	**2.40**	−7.9
**2.7**	−7.4	**2.24**	−8.4	**2.41**	−9.2
**2.8**	−7.6	**2.25**	−8.0	**2.42**	−8.1
**2.9**	−7.5	**2.26**	−8.5	**2.43**	−8.6
**2.10**	−8.6	**2.27**	−8.2	**2.44**	−8.9
**2.11**	−8.4	**2.28**	−8.6	**2.45**	−8.5
**2.12**	−7.8	**2.29**	−8.9	**2.46**	−8.0
**2.13**	−8.1	**2.30**	−8.6	**2.47**	−7.8
**2.14**	−8.3	**2.31**	−8.7	**2.48**	−8.7
**2.15**	−7.8	**2.32**	−7.5	**Ciprofloxacin**	−6.7
**2.16**	−8.1	**2.33**	−7.8	-	-

* TA: (2-(1,2,4-triazol-5-yl)aniline.

**Table 2:** Antimicrobial activity of compounds **2** against *Staphylococcus aureus ATCC 25923* strain.

Compounds	R	R^1^	MIC *, μM	MBC **, μM	MBC/MIC
**2.1**	cyclopropyl	H	62.4	124.8	2
**2.2**	cyclopropyl	6-Me	933.4	933.4	1
**2.3**	cyclopropyl	5-F	458.2	916.5	2
**2.4**	cyclopropyl	4-Cl	852.2	852.2	1
**2.5**	cyclobutyl	H	14.6	23.3	1.5
**2.6**	cyclobutyl	6-Me	876.1	876.1	1
**2.7**	cyclobutyl	5-F	430.6	861.2	2
**2.8**	cyclobutyl	4-Cl	402.1	804.2	2
**2.9**	cyclopentyl	H	27.4	438.0	16
**2.10**	cyclopentyl	6-Me	825.4	825.4	1
**2.11**	cyclopentyl	5-F	203.0	406.0	2
**2.12**	cyclopentyl	4-Cl	47.6	47.6	1
**2.13**	cyclohexyl	H	26.8	206.3	7.7
**2.14**	cyclohexyl	6-Me	390.1	390.1	1
**2.15**	cyclohexyl	5-F	192.1	768.3	4
**2.16**	cyclohexyl	4-Cl	361.3	723.6	2
**2.17**	adamantyl-1	H	10.6	21.2	2
**2.18**	adamantyl-1	6-Me	10.1	20.2	2
**2.19**	adamantyl-1	5-F	320.1	640.2	2
**2.20**	adamantyl-1	4-Cl	304.1	608.2	2
**2.21**	adamantyl-1	4-Br	267.9	535.7	2
**2.22**	Ph	H	26.4	211.6	8
**2.23**	4-FC_6_H_4_	H	196.6	786.6	4
**2.24**	4-ClC_6_H_4_	H	92.3	184.6	2
**2.25**	4-BrC_6_H_4_	H	317.3	634.6	2
**2.26**	2-FC_6_H_4_	H	12.4	24.8	2
**2.27**	furan-2-yl	H	221.0	442.0	2
**2.28**	furan-3-yl	6-Me	13.0	52.0	4
**2.29**	furan-3-yl	5-F	25.6	51.2	2
**2.30**	furan-3-yl	4-Cl	11.9	23.8	2
**2.31**	furan-3-yl	4-Br	5.2	10.4	2
**2.32**	thiophen-2-yl	H	103.2	206.4	2
**2.33**	thiophen-2-yl	5-F	24.0	48.0	2
**2.34**	thiophen-3-yl	6-Me	12.2	12.2	1
**2.35**	thiophen-3-yl	5-F	6.1	48.0	8
**2.36**	thiophen-3-yl	4-Cl	45.2	180.8	4
**2.37**	thiophen-3-yl	4-Br	77.8	311.3	4
**2.38**	benzofuran-2-yl	H	180.9	361.8	2
**2.39**	benzofuran-2-yl	6-Me	10.7	21.4	2
**2.40**	benzofuran-2-yl	5-F	42.5	84.9	2
**2.41**	benzofuran-2-yl	4-Cl	20.1	40.2	2
**2.42**	benzofuran-2-yl	4-Br	140.7	140.7	1
**2.43**	benzothiophen-2-yl	H	171.0	342.0	2
**2.44**	indol-2-yl	H	181.6	363.2	2
**2.45**	pyridin-2-yl	H	105.3	210.6	2
**2.46**	pyridin-3-yl	H	13.2	52.8	4
**2.47**	pyridin-4-yl	H	105.3	210.6	2
**2.48**	pyridin-4-yl	Br	79.1	158.2	2
**Ciprofloxacin**			4.7	9.6	2

* MIC—minimal inhibitory concentration; ** MBC—minimum bactericidal concentration.

**Table 3:** Physicochemical descriptors and pharmacokinetic properties of compounds **2** provided by SwissADME.

Physicochemical Descriptors and Predicted Pharmacokinetic Properties *	Compounds										
	2.17	2.18	2.26	2.28	2.30	2.31	2.34	2.35	2.39	2.46	CF **
MW (Da) (<500)	294.39	308.42	252.27	240.26	260.68	305.13	256.33	260.29	290.32	237.26	331.34
n-ROTB (<10)	2	2	2	2	2	2	2	2	2	2	3
n-HBA (<10)	2	2	2	3	3	3	2	3	3	3	5
n-HBD (≤5)	2	2	2	2	2	2	2	2	2	3	2
TPSA (<140, Å^2^)	67.59	67.59	87.82	80.73	80.73	80.73	95.83	95.83	80.73	80.48	74.57
logP (≤5)	3.21	3.54	2.05	2.06	2.30	2.34	2.74	2.69	3.07	1.66	1.10
Molar refractivity	88.11	93.08	73.68	68.89	68.94	71.63	74.50	69.49	86.40	69.45	95.25
Gastrointestinal absorption	high	high	high	high	high	high	high	high	high	high	high
**Drug-likeness**											
Lipinski (Pfizer) filter [43]	yes	yes	yes	yes	yes	yes	yes	yes	yes	yes	yes
Veber (GSK) filter [44]	yes	yes	yes	yes	yes	yes	yes	yes	yes	yes	yes
Muegge (Bayer) filter [45]	yes	yes	yes	yes	yes	yes	yes	yes	yes	yes	yes
Ghose filter [46]	yes	yes	yes	yes	yes	yes	yes	yes	yes	yes	yes
Egan filter [47]	yes	yes	yes	yes	yes	yes	yes	yes	yes	yes	yes
Bioavailability score [48]	0.55	0.55	0.55	0.55	0.55	0.55	0.55	0.55	0.55	0.55	0.55
Lead-likeness	no	no	yes	no	yes	yes	yes	yes	yes	no	yes

* MW: molecular weight; n-ROTB: number of rotatable bonds; n-HBA: number of hydrogen bond acceptors; n-HBD: number of hydrogen bond donors; TPSA: topological polar surface area; ** CF: Ciprofloxacin.

## Data Availability

All the data generated during this research are included in the manuscript and Supplementary Materials.

## Supplementary Materials

The following supporting information can be downloaded at https://www.mdpi.com/article/10.3390/ph18010083/s1, Table S1: The results of the docking studies of the ligands 2 and the native inhibitor to the active site of DNA gyrase (2XCT); HPLC-MS data, ^1^H NMR data and ^13^C NMR data.

## References

[1] El-Aleam R.H.A., George R.F., Georgey H.H., Abdel-Rahman H.M. (2021). Bacterial virulence factors: A target for heterocyclic compounds to combat bacterial resistance. RSC Adv..

[2] Koulenti D., Xu E., Mok I.Y.S., Song A., Karageorgopoulos D.E., Armaganidis A., Lipman J., Tsiodras S. (2019). Novel Antibiotics for Multidrug-Resistant Gram-Positive Microorganisms. Microorganisms.

[3] Theuretzbacher U. (2011). Resistance drives antibacterial drug development. Curr. Opin. Pharmacol..

[4] Vimalah V., Getha K., Mohamad Z.N., Mazlyzam A.L. (2020). A Review on Antistaphylococcal Secondary Metabolites from Basidiomycetes. Molecules.

[5] Brown E., Wright G. (2016). Antibacterial drug discovery in the resistance era. Nature.

[6] Esposito S., Blasi F., Curtis N., Kaplan S., Lazzarotto T., Meschiari M., Mussini C., Peghin M., Rodrigo C., Vena A. (2023). New Antibiotics for *Staphylococcus aureus* Infection: An Update from the World Association of Infectious Diseases and Immunological Disorders (WAidid) and the Italian Society of Anti-Infective Therapy (SITA). Antibiotics.

[7] Wright P.M., Seiple I.B., Myers A.G. (2014). The evolving role of chemical synthesis in antibacterial drug discovery. Angew. Chem. Int. Ed..

[8] Doytchinova I. (2022). Drug Design-Past, Present, Future. Molecules.

[9] Anstead G.M., Cadena J., Javeri H. (2014). Treatment of infections due to resistant *Staphylococcus aureus*. Methods Mol. Biol..

[10] Bisacchi G.S., Manchester J.I. (2015). A New-Class Antibacterial–Almost. Lessons in Drug Discovery and Development: A Critical Analysis of More than 50 Years of Effort toward ATPase Inhibitors of DNA Gyrase and Topoisomerase IV. ACS Infect. Dis..

[11] Khan T., Sankhe K., Suvarna V., Sherje A., Patel K., Dravyakar B. (2018). DNA gyrase inhibitors: Progress and synthesis of potent compounds as antibacterial agents. Biomed. Pharmacother..

[12] Durcik M., Tomašič T., Zidar N., Zega A., Kikelj D., Mašič L.P., Ilaš J. (2019). ATP-competitive DNA gyrase and topoisomerase IV inhibitors as antibacterial agents. Expert Opin. Ther. Pat..

[13] Dighe S.N., Collet T.A. (2020). Recent advances in DNA gyrase-targeted antimicrobial agents. Eur. J. Med. Chem..

[14] Man R.-J., Zhang X.-P., Yang Y.-S., Jiang A.-Q., Zhu H.-L. (2021). Recent Progress in Small Molecular Inhibitors of DNA Gyrase. Curr. Med. Chem..

[15] Poonam P., Ajay K., Akanksha K., Tamanna V., Vritti P. (2024). Recent Development of DNA Gyrase Inhibitors: An Update. Mini-Rev. Med. Chem..

[16] Ashley R.E., Dittmore A., McPherson S.A., Turnbough C.L., Neuman K.C., Osheroff N. (2017). Activities of gyrase and topoisomerase IV on positively supercoiled DNA. Nucleic Acids Res..

[17] Hiasa H. (2018). DNA Topoisomerases as Targets for Antibacterial Agents. Methods Mol. Biol..

[18] Watkins R.R., Thapaliya D., Lemonovich T.L., Bonomo R.A. (2023). Gepotidacin: A novel, oral, ‘first-in-class’ triazaacenaphthylene antibiotic for the treatment of uncomplicated urinary tract infections and urogenital gonorrhoea. J. Antimicrob. Chemother..

[19] Terreni M., Taccani M., Pregnolato M. (2021). New Antibiotics for Multidrug-Resistant Bacterial Strains: Latest Research Developments and Future Perspectives. Molecules.

[20] Naeem A., Badshah S.L., Muska M., Ahmad N., Khan K. (2016). The Current Case of Quinolones: Synthetic Approaches and Antibacterial Activity. Molecules.

[21] Millanao A.R., Mora A.Y., Villagra N.A., Bucarey S.A., Hidalgo A.A. (2021). Biological Effects of Quinolones: A Family of Broad-Spectrum Antimicrobial Agents. Molecules.

[22] Fesatidou M., Anthi P., Geronikaki A. (2020). Heterocycle Compounds with Antimicrobial Activity. Curr. Pharm. Des..

[23] Murugaiyan J., Kumar P.A., Rao G.S., Iskandar K., Hawser S., Hays J.P., Mohsen Y., Adukkadukkam S., Awuah W.A., Jose R.A.M. (2022). Progress in Alternative Strategies to Combat Antimicrobial Resistance: Focus on Antibiotics. Antibiotics.

[24] Boparai J.K., Sharma P.K. (2020). Mini Review on Antimicrobial Peptides, Sources, Mechanism and Recent Applications. Protein Pept. Lett..

[25] Nasiri Sovari S., Zobi F. (2020). Recent Studies on the Antimicrobial Activity of Transition Metal Complexes of Groups 6–12. Chemistry.

[26] Feng G., Tengfei W., Jiaqi X., Gang H. (2019). Antibacterial activity study of 1,2,4-triazole derivatives. Eur. J. Med. Chem..

[27] Strzelecka M., Świątek P. (2021). 1,2,4-Triazoles as Important Antibacterial Agents. Pharmaceuticals.

[28] Kazeminejad Z., Marzi M., Shiroudi A., Kouhpayeh S.A., Farjam M., Zarenezhad E. (2022). Novel 1,2,4-Triazoles as Antifungal Agents. Biomed. Res. Int..

[29] Xuemei G., Zhi X. (2020). 1,2,4-Triazole hybrids with potential antibacterial activity against methicillin-resistant *Staphylococcus aureus*. Arch. Pharm..

[30] Jie L., Junwei Z. (2022). The Antibacterial Activity of 1,2,3-triazole- and 1,2,4-Triazole-containing Hybrids against *Staphylococcus aureus*: An Updated Review (2020–Present). Curr. Top. Med. Chem..

[31] Verdirosa F., Gavara L., Sevaille L., Tassone G., Corsica G., Legru A., Feller G., Chelini G., Mercuri P.S., Tanfoni S. (2022). 1,2,4-Triazole-3-Thione Analogues with a 2-Ethylbenzoic Acid at Position 4 as VIM-type Metallo-β-Lactamase Inhibitors. ChemMedChem.

[32] Sergeieva T., Bilichenko M., Kholodnyak S., Monaykina Y., Okovytyy S., Kovalenko S., Voronkov E., Leszczynski J. (2016). Origin of Substituent Effect on Tautomeric Behavior of 1,2,4-Triazole Derivatives. Combined Spectroscopic and Theoretical Study. J. Phys. Chem. A.

[33] Pylypenko O.O., Okovytyy S.I., Sviatenko L.K., Voronkov E.O., Shabelnyk K.P., Kovalenko S.I. (2023). Tautomeric behavior of 1,2,4-triazole derivatives: Combined spectroscopic and theoretical study. Struct. Chem..

[34] Francis J.E., Cash W.D., Psychoyos S., Ghai G., Wenk P., Friedmann R.C., Atkins C., Warren V., Furness P., Hyun J.L. (1988). Structure-activity profile of a series of novel triazoloquinazoline adenosine antagonists. J. Med. Chem..

[35] Balo C., López C., Brea J.M., Fernánde F., Caamaño O. (2007). Synthesis and Evaluation of Adenosine Antagonist Activity of a Series of [1,2,4]Triazolo[1,5-c]quinazolines. Chem. Pharm. Bull..

[36] Khan G., Sreenivasa S., Govindaiah S., Chandramohan V., Shetty P.R. (2020). Synthesis, biological screening, in silico study and fingerprint applications of novel 1,2,4-triazole derivatives. J. Het. Chem..

[37] Kholodnyak S.V., Schabelnyk K.P., Zhernova G.O., Sergeieva T.Y., Ivchuk V.V., Voskoboynik O.Y., Kovalenko S.I., Trzhetsinskii S.D., Okovytyy S.I., Shishkina S.V. (2015). Hydrolytic cleavage of pyrimidine ring in 2-aryl-[1,2,4]triazolo[1,5-c]-quinazolines: Physico-chemical properties and hypoglycemia activity of the synthesized compounds. News Pharm..

[38] Pylypenko O.O., Sviatenko L.K., Shabelnyk K.P., Kovalenko S.I., Okovytyy S.I. (2024). Synthesis and hydrolytic decomposition of 2-hetaryl[1,2,4]triazolo[1,5-c]quinazolines: DFT Study. Struct. Chem..

[39] Kovalenko S.I., Antypenko L.M., Bilyi A.K., Kholodnyak S.V., Karpenko O.V., Antypenko O.M., Mykhaylova N.S., Los T.I., Kolomoets O.S. (2013). Synthesis and Anticancer Activity of 2-(Alkyl-, Alkaryl-, Aryl-, Hetaryl-)-[1,2,4]triazolo[1,5-c]quinazolines. Sci. Pharm..

[40] Breitmaier E. (2002). Structure Elucidation by NMR in Organic Chemistry: A Practical Guide.

[41] Zefirov Y.V. (1997). Reduced intermolecular contacts and specific interactions in molecular crystals. Crystallogr. Rep..

[42] Tao L., Zhang P., Qin C., Chen S.Y., Zhang C., Chen Z., Zhu F., Yang S.Y., Wei Y.Q., Chen Y.Z. (2015). Recent progresses in the exploration of machine learning methods as in-silico ADME prediction tools. Adv. Drug Deliv. Rev..

[43] Lipinski C.A., Lombardo F., Dominy B.W., Feeney P.J. (2001). Experimental and computational approaches to estimate solubility and permeability in drug discovery and development settings. Adv. Drug Deliv. Rev..

[44] Veber D.F., Johnson S.R., Cheng H.-Y., Smith B.R., Ward K.W., Kopple K.D. (2002). Molecular properties that influence the oral bioavailability of drug candidates. J. Med. Chem..

[45] Muegge I., Heald S.L., Brittelli D. (2001). Simple selection criteria for drug-like chemical matter. J. Med. Chem..

[46] Ghose A.K., Viswanadhan V.N., Wendoloski J.J. (1999). A knowledge-based approach in designing combinatorial or medicinal chemistry libraries for drug discovery. 1. A qualitative and quantitative characterization of known drug databases. J. Combin. Chem..

[47] Egan W.J., Merz K.M., Baldwin J.J. (2000). Prediction of drug absorption using multivariate statistics. J. Med. Chem..

[48] Martin Y.C. (2005). A bioavailability score. J. Med. Chem..

[49] Zhang Y., Chen L., Xu H., Li X., Zhao L., Wang W., Li B., Zhang X. (2018). 6,7-Dimorpholinoalkoxy quinazoline derivatives as potent EGFR inhibitors with enhanced antiproliferative activities against tumor cells. Eur. J. Med. Chem..

[50] Sheldrick G.M. (2008). A Short History of SHELX. Acta Crystallogr..

[51] Kumar H.S.S., Kumar S.R., Kumar N.N., Ajith S. (2021). Molecular docking studies of gyrase inhibitors: Weighing earlier screening bedrock. In Silico Pharmacol..

[52] MarvinSketch version 20.21.0, ChemAxon. http://www.chemaxon.com.

[53] Trott O., Olson A.J. (2010). AutoDock Vina: Improving the speed and accuracy of docking with a new scoring function, efficient optimization, and multithreading. J. Comput. Chem..

[54] Discovery Studio Visualizer v21.1.0.20298 Accelrys Software Inc. https://www.3dsbiovia.com.

[55] Baber J.C., Thompson D.C., Cross J.B., Humblet C. (2009). GARD: A Generally Applicable Replacement for RMSD. J. Chem. Inf. Model..

[56] Warren G.L., Andrews C.W., Capelli A.-M., Clarke B., LaLonde J., Lambert M.H., Lindvall M., Nevins N., Semus S.F., Senger S. (2005). A critical assessment of docking programs and scoring functions. J. Med. Chem..

[57] DockRMSD (2022). Docking Pose Distance Calculation. https://zhanggroup.org/DockRMSD/.

[58] Wayne P.A., CLSI (2020). Performance Standards for Antimicrobial Susceptibility Testing.

[59] SwissADME. http://www.swissadme.ch/index.php#.

[60] Bilyi A.K., Antypenko L.M., Ivchuk V.V., Kamyshnyi O.M., Polishchuk N.M., Kovalenko S.I. (2015). 2-Heteroaryl-[1,2,4]triazolo[1,5-c]quinazoline-5(6 H)-thiones and Their S-Substituted Derivatives: Synthesis, Spectroscopic Data, and Biological Activity. ChemPlusChem.

[61] Nosulenko I.S., Voskoboynik O.Y., Berest G.G., Safronyuk S.L., Kovalenko S.I., Kamyshnyi O.M., Polishchuk N.M., Sinyak R.S., Katsev A.V. (2014). Synthesis and Antimicrobial Activity of 6-Thioxo-6,7-dihydro-2H-[1,2,4]triazino[2,3-c]-quinazolin-2-one Derivatives. Sci. Pharm..

