# Supplementary material for: Antistaphylococcal Triazole-Based Molecular Hybrids: Design, Synthesis and Activity
Source: Pharmaceuticals (Basel). 2025 Jan 11;18(1):83. doi: 10.3390/ph18010083 (PMC11769325; doi:10.3390/ph18010083)

pharmaceuticals

MDPI

Supplementary Materials

An Antistaphylococcal Triazole-based Molecular Hybrids: Design, Synthesis and Activity.

Kostiantyn Shabelnyk [1,*], Alina Fominichenko [2], Oleksii Antypenko [1], Olexandr Gaponov [3], Svitlana Koptieva [3], Svitlana Shyshkina [4], Oleksii Voskoboinik [5], Sergiy Okovytyy [3], Serhii Kovalenko [3], Valentyn Oksenych [6] and Oleksandr Kamyshnyi [7,*]

1 Department of Pharmaceutical, organic and bioorganic chemistry, Zaporizhzhia State Medical and Pharmaceutical University, 69000, Zaporizhzhia, Ukraine; antypenkoan@gmail.com (O.A.)
2 Bacteriological Laboratory, Zaporizhzhia Regional Hospital, 69600, Zaporizhzhia, Ukraine; fominichenkoalina@gmail.com (A.F.)
3 Oles Honchar Dnipro National University, 49000, Dnipro, Ukraine; gaponov.aa.58@gmail.com (O.G.), svetkopteva@gmail.com (Sv.K.), sokovyty@icnanotox.org (S.O.), kovalenkosergiy@gmail.com (S.K.)
4 SSI "Institute for Single Crystals" of the National Academy of Sciences of Ukraine, 61072, Kharkiv, Ukraine; sveta@xray.isc.kharkov.com (S.S.)
5 National University «Zaporizhzhia Polytechnic», 69063, Zaporizhzhia, Ukraine; a.yu.voskoboynik@gmail.com (O.V.)
6 Department of Clinical Science, University of Bergen, 5020 Bergen, Norway.
7 Department of Microbiology, Virology and Immunology, I. Horbachevsky Ternopil State Medical University, 46001 Ternopil, Ukraine; kamyshnyi_om@tdmu.edu.ua (O.K.)

* Correspondence: kshabelnik@gmail.com (K.S.), valentyn.oksenych@uib.no (V.O.)

Content

Table S1	S3
HPLC data of compound **2.1**	S6
^{1}H NMR spectrum of compound **2.1**	S7
^{13}C NMR spectrum of compound **2.1**	S8
^{1}H NMR spectrum of compound **2.2**	S9
HPLC MS data of compound **2.3**	S10
^{1}H NMR spectrum of compound **2.3**	S11
HPLC MS data of compound **2.4**	S12
^{1}H NMR spectrum of compound **2.4**	S13
HPLC MS data of compound **2.5**	S14
^{1}H NMR spectrum of compound **2.5**	S15
HPLC MS data of compound **2.6**	S16
^{1}H NMR spectrum of compound **2.6**	S17
HPLC MS data of compound **2.7**	S18
^{1}H NMR spectrum of compound **2.7**	S19
HPLC MS data of compound **2.8**	S20
^{1}H NMR spectrum of compound **2.8**	S21
HPLC MS data of compound **2.9**	S22
^{1}H NMR spectrum of compound **2.9**	S23
HPLC MS data of compound **2.10**	S24
^{1}H NMR spectrum of compound **2.10**	S25
HPLC MS data of compound **2.11**	S26
^{1}H NMR spectrum of compound **2.11**	S27
HPLC MS data of compound **2.12**	S28
^{1}H NMR spectrum of compound **2.12**	S29
^{1}H NMR spectrum of compound **2.13**	S30
^{13}C NMR spectrum of compound **2.13**	S31
HPLC MS data of compound **2.14**	S32
^{1}H NMR spectrum of compound **2.14**	S33
HPLC MS data of compound **2.15**	S34
^{1}H NMR spectrum of compound **2.15**	S35
HPLC MS data of compound **2.16**	S36
^{1}H NMR spectrum of compound **2.16**	S37

HPLC MS data of compound **2.17** S38
^{1}H NMR spectrum of compound **2.17** S39
^{13}C NMR spectrum of compound **2.17** S40
HPLC MS data of compound **2.18** S41
^{1}H NMR spectrum of compound **2.18** S42
HPLC MS data of compound **2.19** S43
^{1}H NMR spectrum of compound **2.19** S44
HPLC MS data of compound **2.20** S45
^{1}H NMR spectrum of compound **2.20** S46
HPLC MS data of compound **2.21** S47
^{1}H NMR spectrum of compound **2.21** S48
^{1}H NMR spectrum of compound **2.22** S49
^{1}H NMR spectrum of compound **2.23** S50
^{1}H NMR spectrum of compound **2.24** S51
^{1}H NMR spectrum of compound **2.25** S52
^{1}H NMR spectrum of compound **2.26** S53
^{13}C NMR spectrum of compound **2.26** S54
^{1}H NMR spectrum of compound **2.27** S55
HPLC MS data of compound **2.28** S56
^{1}H NMR spectrum of compound **2.28** S57
HPLC MS data of compound **2.29** S58
^{1}H NMR spectrum of compound **2.29** S59
HPLC MS data of compound **2.30** S60
^{1}H NMR spectrum of compound **2.30** S61
^{1}H NMR spectrum of compound **2.31** S62
^{1}H NMR spectrum of compound **2.32** S63
^{13}C NMR spectrum of compound **2.32** S64
HPLC data of compound **2.33** S65
^{1}H NMR spectrum of compound **2.33** S66
HPLC MS data of compound **2.34** S67
^{1}H NMR spectrum of compound 2.34 S68
HPLC MS data of compound **2.35** S69
^{1}H NMR spectrum of compound **2.35** S70
HPLC MS data of compound **2.36** S71
^{1}H NMR spectrum of compound **2.36** S72
HPLC MS data of compound **2.37** S73
^{1}H NMR spectrum of compound **2.37** S74
^{1}H NMR spectrum of compound **2.38** S75
^{13}C NMR spectrum of compound **2.38** S76
^{1}H NMR spectrum of compound **2.39** S77
^{1}H NMR spectrum of compound **2.40** S78
HPLC MS data of compound **2.41** S79
^{1}H NMR spectrum of compound **2.41** S80
HPLC MS data of compound **2.42** S81
^{1}H NMR spectrum of compound **2.42** S82
^{1}H NMR spectrum of compound **2.45** S83
^{13}C NMR spectrum of compound **2.45** S84
^{1}H NMR spectrum of compound **2.46** S85
^{13}C NMR spectrum of compound **2.46** S86
HPLC MS data of compound **2.47** S87
^{1}H NMR spectrum of compound **2.47** S88
^{13}C NMR spectrum of compound **2.47** S89
HPLC MS data of compound **2.48** S90
^{1}H NMR spectrum of compound **2.48** S91

Table S1. The results of the docking studies of the ligands 2 and the native inhibitor to the active site of DNA gyrase (**2XCT**)

Compounds	Affinity (kcal/mol)	Amino acid residues and nucleotides interaction *
**TA****	-6.3	F:DG8 (2.39 Å)[a], N4-NH_2 (2.26 Å)[a], D:ARG458 (2.28 Å)[a], F:DG8 (3.73; 4.44; 4.87; 4.99 Å)[b], DG9 (4.16 Å)[b], H:DG9 (4.69 Å)[b]
**2.1**	-6.7	F:DG8 (3.37 Å)[a], G:DC13 (2.72 Å)[a], H:DG9 (3.88; 4.73 Å)[b], G:DC12 (5.85 Å)[c], F:DG8 (4.08; 4.32 Å)[d], H:DG9 (4.14; 4.39 Å)[d], ARG458 (4.36 Å)[d]
**2.2**	-7.4	F:DG8 (2.79 Å)[a], H:DG9 (2.25 Å)[a], ARG458 (2.29 Å)[a], N4-NH_2 (1.85 Å)[a], F:DG8 (4.21; 4.57 Å)[b], H:DG9 (3.65; 4.16; 4.43; 4.61 Å)[b], DC12 (5.89 Å)[b], DC13 (4.72 Å)[b], D:ARG458 (4.77 Å)[e], G:DC13 (4.75 Å)[d], DG9 (5.44 Å)[d]
**2.3**	-8.4	B:ARG1122 (3,25 Å)[g], D:ARG458 (2,42 Å)[a], N4-NH_2 (1,70 Å)[a], H:DG9 (2,06 Å)[a], F:DG8 (2,99 Å)[a], F:DG8 (5,55 Å)[b], H:DG9 (3,57; 4,14; 4,56; 4,57 Å)[b], F:DG8 (4,33 Å)[b], DC12 (5,93 Å)[b], DC13 (4,77 Å)[b], D:ARG458 (4,63 Å)[e], G:DC13 (5,05 Å)[d], H:DG9 (5,05 Å)[e]
**2.4**	-7.1	D:SER1084 (3.00 Å)[f], F;DG8 (2.70 Å)[a], D:ARG458 (2.58 Å)[a], F:DG8 (3.73; 4.02; 4.42; 5.17 Å)[b], H:DG9 (3.74; 4.82; 4.56; 5.20 Å)[b], G:DC13 (4.98 Å)[b], D;ARG458 (4.88 Å)[e], F:DG8 (5.24 Å)[d], G:DC12 (5.50 Å)[d], G:DC13 (4.96 Å)[d], H:DG9 (4.79 Å)[d]
**2.5**	-7.1	F:DG8 (2.62 Å)[a], D:ARG458 (2.36 Å)[a], F:DG8 (3.75; 4.20; 4.25; 5.52 Å)[b], H:DG9 (3.70; 4.61; 4.73; 4.81 Å)[b], G:DC12 (5.90 Å)[b], G:DC13 (4.61 Å)[b], D:ARG458 (4.45 Å)[e], G:DC13 (5.31 Å)[d], H:DG9 (5.03 Å)[d]
**2.6**	-7.6	G:DC13 (2.85 Å)[a], D:ARG458 (2.32 Å)[a], N4-NH_2 (2.08 Å)[a], F:DG8 (3.80; 4.19 Å)[b], H:DG9 (3.70; 4.48; 4.55; 4.67 Å)[b], G:DC13 (4.96 Å)[b], D:ARG458 (4.17 Å)[e], H:DG9 (5.41 Å)[d]
**2.7**	-7.4	D:ARG458 (2.59 Å)[a], H:DG9 (2.64 Å)[a], N4-NH_2 (2.18 Å)[a], F:DG8 (4.17 Å)[b], F:DG8 (4.48 Å)[b], H:DG9 (3.64; 4.13; 4.41; 4.59 Å)[b], G:DC13 (4.74 Å)[b], D:ARG458 (4.40 Å)[d], G:DC13 (5.10 Å)[e]
**2.8**	-7.6	G:DC13 (3.13 Å)[a], F:DG8 (2.63 Å)[a], H:DG9 (2.24; 2.64 Å)[a], D:ARG458 (1.88 Å)[a], H:DG9 (3.70; 4.78 Å)[b], G:DC12 (5.63 Å)[b], G:DC13 (4.48 Å)[b], D:ARG458 (4.34 Å)[e], F:DG8 (4.22; 4.51; 4.54; 5.49)[d], G:DC13 (5.49)[d], H:DG9 (4.11; 4.37; 5.22)[d]
**2.9**	-7.5	F:DG8 (3.24 Å)[a], G:DC13 (2.70 Å)[a], H:DG9 (3.89; 4.81 Å)[b], G:DC13 (4.61 Å)[b], F:DG8 (4.29; 4.35Å)[d], H:DG9 (3.95; 4.24 Å)[d], D:ARG458 (4.31 Å)[d]
**2.10**	-8.6	F:DG8 (2.74 Å)[a], H:DG9 (3.17 Å)[a], F:DG8 (3.73; 3.98Å)[b], H:DG9 (3.74; 4.51; 4.74; 4.79 Å)[b], G:DC13 (4.49 Å)[b], D:ARG458 (4.27 Å)[e]
**2.11**	-8.4	F:DG8 (2.57; 2.60 Å)[a], F:DG8 (3.90; 4.10; 4.52; 5.27 Å)[b], H:DG9 (3.66; 4.62; 4.77; 4.92 Å)[b], G:DC13 (4.82 Å)[b], D:ARG458 (4.54 Å)[e], G:DC135.25 Å)[d], H:DG9 (5.15 Å)[d]
**2.12**	-7.8	F:DG8 (2.84 Å)[a], H:DG9 (2.14 Å)[a], D:ARG458 (2.33 Å)[a], N4-NH_2 (2.11 Å)[a], F:DG8 (4.12; 4.25; 4.56; 5.50 Å)[b], H:DG9 (3.73; 4.32; 4.56; 4.63 Å)[b], G:DC13 (4.77 Å)[b], D:ARG458 (4.40 Å)[e], F:DG8 (4.52; 5.48 Å)[d], H:DG9 (5.27 Å)[d]
**2.13**	-8.1	F:DG8 (2.93 Å)[a], H:DG9 (1.92 Å)[a], D:ARG458 (1.92 Å)[b], F:DG8 (4.08; 4.39 Å)[b], H:DG9 (3.64; 4.21; 4.48; 4.62 Å)[b], G:DC13 (4.70 Å)[b], D:ARG458 (4.31 Å)[e]
**2.14**	-8.3	F:DG8 (2.82 Å)[a], H:DG9 (2.33 Å)[a], N4-NH_2 (2.54 Å)[a], D:ARG458 (2.62 Å)[a], F:DG8 (4.04; 4.43 Å)[b], H:DG9 (3.62; 4.33; 4.55; 4.91 Å)[b], D:ARG458 (4.45 Å)[e]
**2.15**	-7.8	F:DG8 (2.65 Å)[a], D:ARG458 (2.09 Å)[a], N4-NH_2 (2.55 Å)[a], F:DG8 (3.74; 4.23; 4.30; 5.43 Å)[b], H:DG9 (3.62; 4.56; 4.70; 5.00 Å)[b], G:DC13 (4.83 Å)[b], D:ARG458 (4.25 Å)[e], H:DG9 (5.47 Å)[d]
**2.16**	-8.1	D:SER1084 (3.23 Å)[f], F:DG8 (2.96 Å)[a], H:DG9 (2.37 Å)[a], N4-NH_2 (2.53 Å)[a], F:DG8 (4.11; 4.47Å)[b], H:DG9 (3.71; 4.31; 4.42; 4.55 Å)[b], G:DC13 (4.85 Å)[b], D:ARG458 (4.38 Å)[e]

**2.17**	-5.5	D:ARG458 (2.34 Å)[a], H:DG9 (2.15; 3.64Å)[a], F:DG8 (3.79; 4.37 Å)[b], G:DC12 (5.25 Å)[b], G:DC12 (4.47 Å)[b], H:DG9 (3.84; 4.19; 4.52 Å)[b], F:DG8 (4.60; 4.71; 5.46Å)[d], H:DG9 (4.51; 5.43 Å)[d], D:ARG458 (5.45 Å)[d]
**2.18**	-5.6	H:DG9 (2.54 Å)[a], D:ARG458 (2.41 Å)[a], F:DG8 (3.99; 5.19 Å)[b], H:DG9 (3.77; 4.31; 4.69; 4.77 Å)[b], G:DC13 (4.71 Å)[b], D:ARG458 (4.25; 4.66 Å)[e], G:DC13 (4.97; 5.23 Å)[d], H:DG9 (4.73; 5.43 Å)[d]
**2.19**	-6.1	B:ARG1122 (3.19 Å)[g], H:DG9 (2.42 Å)[a], D:ARG458 (2.85 Å)[a], F:DG8 (5.24 Å)[b], H:DG9 (4.23; 4.62; 4.68 Å)[b], F:DG8 (4.06 Å)[b], G:DC13 (4.76)[b], H:DG9 (3.70)[b], D:ARG458 (4.32; 4.60)[e], G:DC13 (4.96; 5.32)[d], H:DG9 (4.81)[d]
**2.20**	-6.3	D:ARG458 (2.48 Å)[a], G:DC13 (2.09 Å)[a], H:DT10 (3.22 Å)[f], H:DG9 (3.47; 3.58; 4.06 Å)[b], F:DG8 (4.01; 4.74 Å)[d], G:DC12 (5.32 Å)[d], H:DG9 (4.22; 5.29; 5.34 Å)[d], H:DT10 (4.98 Å)[d], D:ARG458 (4.12 Å)[d]
**2.21**	-6.0	G:DC13 (2.13 Å)[a], H:DG9 (3.47 Å)[b], F:DG8 (4.23; 5.15 Å)[b], H:DG9 (3.57; 4.04 Å)[b], F:DG8 (4.04; 4.79 Å)[d], H:DG9 (4.26; 5.34; 5.38 Å)[d], H:DT10 (5.12 Å)[d], D:ARG458 (4.09 Å)[d]
**2.22**	-7.9	G:DC13 (3.11 Å)[a], F:DG8 (2.47 Å)[a], H:DG9 (1.96 Å)[a], D:ARG458 (2.21 Å)[a], NH_2 (2.44 Å)[a], F:DG8 (4.18; 4.73 Å)[b], H:DG9 (3.67; 4.35; 4.54; 4.75 Å)[b], G:DC13 (4.92 Å)[b], D:ARG458 (4.63 Å)[e], G:DC13 (5.15 Å)[d], H:DG9 (5.33 Å)[d]
**2.23**	-8.3	F:DG8 (3.15 Å)[a], G:DC13 (2.13 Å)[a], F:DG8 (3.75; 3.96Å)[b], H:DG9 (3.85; 4.48; 4.69; 4.71 Å)[b], D:ARG458 (4.27 Å)[d]
**2.24**	-8.4	F:DG8 (3.30 Å)[a], G:DC13 (2.01 Å)[a], F:DG8 (3.79; 3.92 Å)[b], H:DG9 (3.88; 4.79 Å)[b], G:DC13 (4.62 Å)[b], G:DC12 (5.78 Å)[b], F:DG8 (4.61 Å)[d], D:ARG458 (4.40 Å)[d]
**2.25**	-8.0	G:DC13 (1.80 Å)[a], H:DG9 (3.58; 4.25; 4.27; 4.78 Å)[b], G:DC13 (5.27 Å)[b], F:DG8 (4.78 Å)[d], D:ARG458 (4.17 Å)[d]
**2.26**	-8.5	H:DG9 (2.49 Å)[a], F:DG8 (2.42 Å)[a], G:DC13 (3.03 Å)[a], D:ARG458 (3.11 Å)[l], H:DG9 (3.64 Å)[l], G:DC13 (3.78 Å)[j], F:DG8 (2.51 Å)[j], F:DG8 (3.80; 4.36 Å)[b], H:DG9 (4.64; 5.06 Å)[b], D:ARG458 (4.57 Å)[e], G:DC13 (5.29 Å)[d]
**2.27**	-8.2	D:ARG458 (2.16 Å)[a], F:DG8 (2.59 Å)[a], F:DG8 (3.71; 4.25 Å)[b], H:DG9 (3.65; 4.54; 4.71; 4.97 Å)[b], G:DC13 (4.86 Å)[b], D:ARG458 (4.43 Å)[d]
**2.28**	-8.6	D:ARG458 (2.12; 3.05 Å)[a], H:DG9 (2.14 Å)[a], N4-NH_2 (2.05 Å)[a], G:DC13 (3.46 Å)[a], F:DG8 (4.21; 4.44 Å)[b], H:DG9 (3.72: 4.06: 4.27: 4.63 Å)[b], G:DC13 (4.74 Å)[b], G:DC12 (6.00 Å)[c], D:ARG458 (4.11 Å)[d]
**2.29**	-8.9	B:ARG1122 (3.68 Å)[g], D:ARG458 (2.02; 3.21; 3.73Å)[a], N4-NH_2 (1.91 Å)[a], H:DG9 (2.22 Å)[a], G:DC13 (3.35 Å)[a], F:DG8 (4.32; 4.52; 4.59; 5.84 Å)[b], H:DG9 (3.77; 3.99; 4.25; 4.69 Å)[b], G:DC12 (5.86; 5.90 Å)[b], G:DC13 (4.67 Å)[b], D:ARG458 (4.25 Å)[d]
**2.30**	-8.6	D:ARG458 (2.53; 3.06; 3.47 Å)[a], H:DG9 (2.46 Å)[a], N4-NH_2 (1.97 Å)[a], G:DC13 (3.33 Å)[a], F:DG8 (4.21; 4.41 Å)[b], H:DG9 (3.71; 4.05; 4.22; 4.66 Å)[b], G:DC12 (5.76 Å)[b], G:DC13 (4.68 Å)[b], F:DG8 (4.68; 5.33 Å)[d], D:ARG458 (4.07 Å)[d]
**2.31**	-8.7	D:ARG458 (2.26; 3.04; 3.43 Å)[a], H:DG9 (2.30 Å)[a], N4-NH_2 (1.85 Å)[a], G:DC13 (3.51 Å)[a], F:DG8 (4.21; 4.36 Å)[b], H:DG9 (3.74; 4.05; 4.15; 4.71Å)[b], G:DC12 (5.69 Å)[b], G:DC13 (4.63 Å)[b], F:DG8 (4.64; 5.29Å)[d], D:ARG458 (4.05 Å)[d]
**2.32**	-7.5	D:ARG458 (2.43 Å)[a], F:DG8 (2.62 Å)[a], D:ARG458 (2.43 Å)[a], F:DG8 (2.62 Å)[a], F:DG8 (3.86; 4.27Å)[b], H:DG9 (3.67; 4.50; 4.62; 4.69 Å)[b], G:DC13 (4.73 Å)[b], D:ARG458 (4.32 Å)[d]
**2.33**	-7.8	D:ARG458 (1.96 Å)[a], G:DC12 (5.56 Å)[i], F:DG8 (3.72; 4.35Å)[b], H:DG9 (3.61; 4.40; 4.77; 5.14 Å)[b], G:DC13 (5.08 Å)[b], D:ARG458 (4.14 Å)[d]
**2.34**	-8.1	D:ARG458 (2.51 Å)[a], N4-NH_2 (1.85 Å)[a], H:DG9 (2.32 Å)[a], G:DC13 (3.27 Å)[a], F:DG8 (2.55 Å)[a], F:DG8 (4.01; 4.37; 4.96; 5.15 Å)[b], H:DG9 (4.25; 4.57; 4.76 Å)[b], G:DC13 (3.69; 4.94 Å)[b], D:ARG458 (4.92 Å)[e], G:DC12 (5.46 Å)[d], G:DC13 (4.62 Å)[d], H:DG9 (5.28 Å)[d]

**2.35**	-7.9	B:ARG1122 (2.92 Å)[g], D:ARG458 (2.83 Å)[a], N4-NH_2 (2.14 Å)[a], F:DG8 (2.84 Å)[a], H:DG9 (3.76; 4.18; 4.20; 4.66; 4.67; 5.23Å)[j], F:DG8 (3.95; 5.12 Å)[b], G:DC13 (4.71; 4.85 Å)[b], D:ARG458 (4.85 Å)[d]
**2.36**	-8.3	D:ARG458 (2.25 Å)[a], N4-NH_2 (2.02 Å)[a], H:DG9 (2.42 Å)[a], F:DG8 (2.87 Å)[a], F:DG8 (4.18; 4.45; Å)[b], H:DG9 (4.12; 4.34; 4.62 Å)[b], G:DC12 (5.81 Å)[b], G:DC13 (4.70 Å)[b], H:DG9 (3.66 Å)[b], F:DG8 (4.69; 5.43 Å)[d], D:ARG458 (4.43 Å)[d]
**2.37**	-8.3	N4-NH_2 (2.05 Å)[a], F:DG8 (2.97 Å)[a], F:DG8 (4.19; 4.46Å)[b], H:DG9 (4.11 Å)[b], H:DG9 (4.30; 4.61 Å)[b], G:DC12 (5.83 Å)[b], G:DC13 (4.74 Å)[b], H:DG9 (3.68Å)[b], F:DG8 (4.64; 5.44Å)[d], D:ARG458 (4.37 Å)[d]
**2.38**	-8.9	H:DG9 (3.03 Å)[a], G:DC13 (2.15 Å)[a], B:ARG1122 (4.16 Å)[h], F:DG8 (3.91 Å)[k], H:DG9 (3.65; 4.17; 4.25; 4.92; 5.68 Å)[b], G:DC13 (5.49 Å)[b], D:ARG458 (4.22 Å)[d]
**2.39**	-8.5	F:DG8 (2.63 Å)[a], N4-NH_2 (2.07 Å)[a], ARG458 (4.20 Å)[h], H:DG9 (4.02 Å)[a], F:DG8 (4.00; 4.13; 4.76; 5.09; 5.34Å)[b], H:DG9 (3.65; 4.49; 4.70; 4.82; 4.88; 5.91 Å)[b], G:DC13 (5.45; 5.06Å)[b], D:ARG458 (4.12; 4.53 Å)[d]
**2.40**	-7.9	D:ARG458 (2.26 Å)[a], N4-NH_2 (1.91 Å)[a], H:DG9 (2.32 Å)[a], D:ARG458 (4.35 Å)[h], F:DG8 (4.14: 4.55 Å)[b], H:DG9 (3.91; 4.29; 4.51; 4.71;4.80; 5.37 Å)[b], G:DC13 (4.81 Å)[b], D:ARG458 (4.13; 4.38 Å)[d]
**2.41**	-9.2	ARG1122 (3.26 Å)[f], G:DC13 (3.32; 4.14 Å)[a], F:DG8 (2.01; 2.37 Å)[a], H:DG9 (2.86 Å)[a], F:DG8 (4.08; 4.84 Å)[b], H:DG9 (3.54; 4.43; 5.01; 5.15 Å)[b], G:DC13 (5.21 Å)[b], D:ARG458 (4.18 Å)[b]
**2.42**	-8.1	H:DG9 (3.11 Å)[a], H:DG9 (3.61; 4.06; 4.29; 4.58; 5.22 Å)[b], G:DC13 (5.24 Å)[b], D:ARG458 (3.40 Å)[e], D:ARG458 (4.45 Å)[d]
**2.43**	-8.6	H:DG9 (3.80; 2.32; 4.20 Å)[a], N4-NH_2 (2.07 Å)[a], D:ARG458 (2.15 Å)[a], B:ARG1122 (4.57 Å)[h], H:DG9 (3.91 Å)[i], F:DG8 (4.15; 4.05; 4.63; 5.38 Å)[b], G:DC12 (5.44 Å)[b], G:DC13 (4.88; 5.05 Å)[b], H:DG9 (3.55; 4.30; 4.42; 4.67; 5.92; 5.36 Å)[b], D:ARG458 (4.82 Å)[d]
**2.44**	-8.9	B:ARG1122 (4.18 Å)[h], F:DG8 (3.01 Å)[a], H:DG9 (3.05 Å)[a], F:DG8 (4.59 Å)[b], H:DG9 (3.77; 3.82; 4.69; 5.31; 5.76 Å)[b], ARG458 (4.35; 5.07 Å)[d]
**2.45**	-8.5	D:ARG458 (2.40 Å)[a], F:DG8 (2.83 Å)[a], F:DG8 (3.80; 4.23 Å)[b], H:DG9 (3.73; 4.55; 4.67; 4.75 Å)[b], G:DC12 (5.71; 5.99 Å)[b], G:DC13 (4.69 Å)[b], D:ARG458 (4.39 Å)[d]
**2.46**	-8.0	D:ARG458 (1.94 Å)[a], N4-NH_2 (2.19 Å)[a], F:DG8 (2.94 Å)[a], G:DC13 (3.33 Å)[a], F:DG8 (3.80; 4.69 Å)[b], H:DG9 (4.16 Å)[b], G:DC12 (5.91 Å)[b], G:DC13 (4.66 Å)[b], H:DG9 (3.73 Å)[b], D:ARG458 (4.30 Å)[e]
**2.47**	-7.8	D:ARG458 (2.24 Å)[a], N4-NH_2 (2.36 Å)[a], F:DG8 (3.06 Å)[a], G:DC12 (2.83 Å)[a], F:DG8 (3.94; 4.02; 4.55; 5.19 Å)[b], G:DC13 (4.57; 4.83 Å)[b], H:DG9 (3.71; 4.59; 4.64; 5.64 Å)[b], D:ARG458 (4.76 Å)[b]
**2.48**	-8.7	D:ARG458 (2.07 Å)[a], N4-NH_2 (1.85 Å)[a], H:DG9 (1.95 Å)[a], H:DG9 (4.66 Å)[b], G:DC12 (5.84 Å)[b], G:DC13 (4.67 Å)[b], H:DG9 (3.71 Å)[b], F:DG8 (4.25; 4.55; 4.56; 5.43 Å)[d], H:DG9 (4.11; 4.29 Å)[d], D:ARG458 (4.41 Å)[d]
**Ciprofloxacin**	-6.7	D:SER1084 (2.51 Å)[a], H:DG9 (2.96; 3.01; 3.25 Å)[a], G:DC12 (3.47 Å)[a], D:ARG458 (3.20 Å)[a], G:DC13 (2.85 Å)[g], F:DG8 (4.11; 4.33 Å)[b], G:DC13 (5.56 Å)[b], H:DG9 (3.43; 4.10; 4.18; 4.30 Å)[b], F:DG8 (5.05; 5.50 Å)[d],F;DG9 (5.33 Å)[d]

Note: * – [a] – Hydrogen Bond (Conventional Hydrogen Bond), [b] – Hydrophobic (Pi-Pi Stacked); [c]-Hydrophobic (Pi-Pi T-shaped); [d] – Hydrophobic (Pi-Alkyl); [e] – Hydrophobic (Alkyl); [f] – Conventional Hydrogen Bond (Cl, Br, I); [g] – Conventional Hydrogen Bon (F); [h] – Electrostatic (Pi-Cation); [i] – Pi-Sulfur; [j] –Pi-Donor Hydrogen Bond; [k] –Other (Pi-Sigma);
** – TA, (2-(1,2,4-triazol-5-yl)aniline.

HPLC MS (methanol-water, APCI) data of compound 2.1

MaxPeak: 100.00%
Ret_Time: 0.928 min

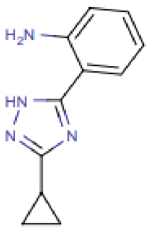

Mol Wt 200.24
Exact Mass 200.12

#	Time	Area%
1	0.928	100.00

RT 0.948

RT 0.946

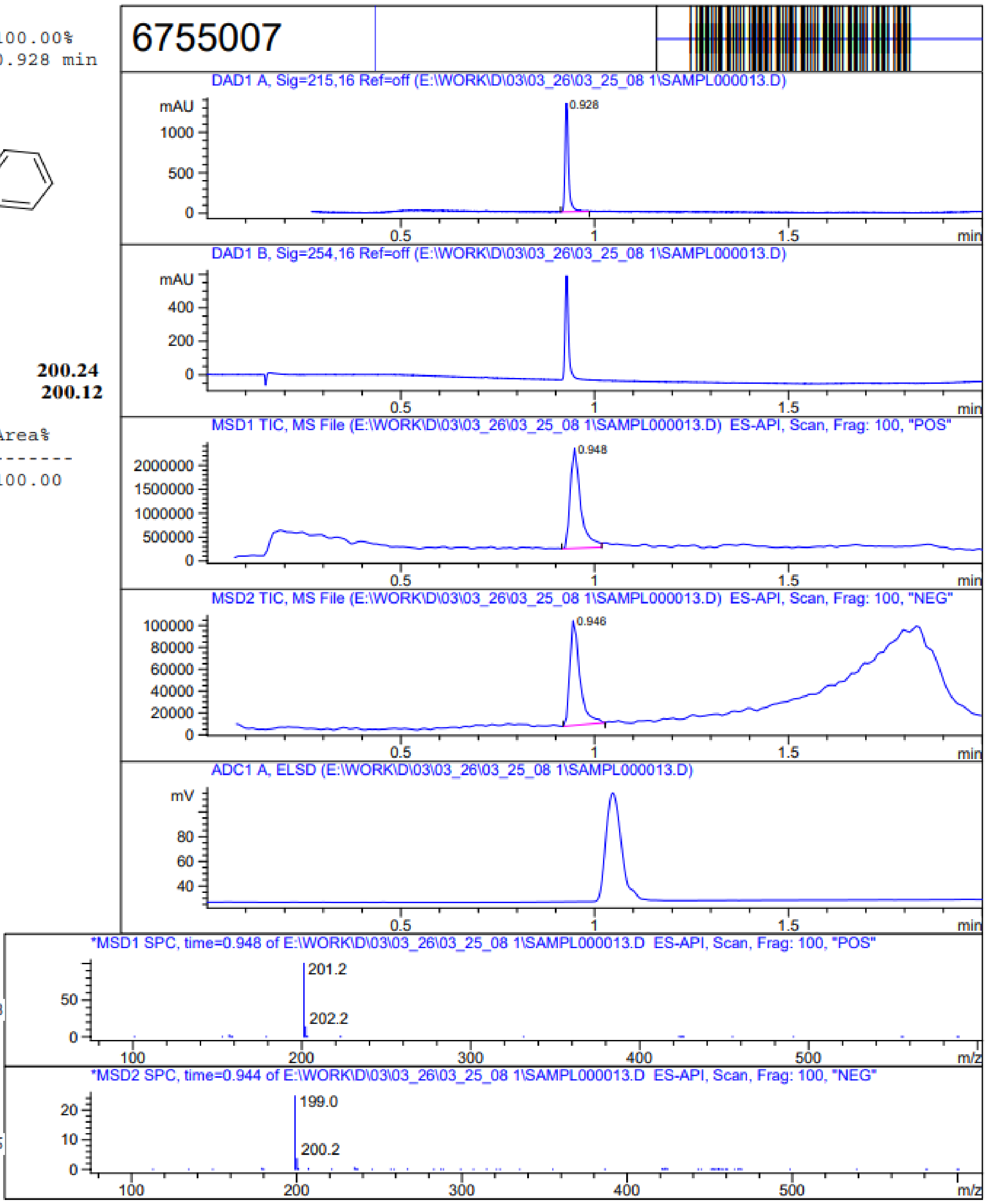

^{1}H NMR (500 MHz) spectrum of compound 2.1 (10 mM in DMSO-d6 at 298K).

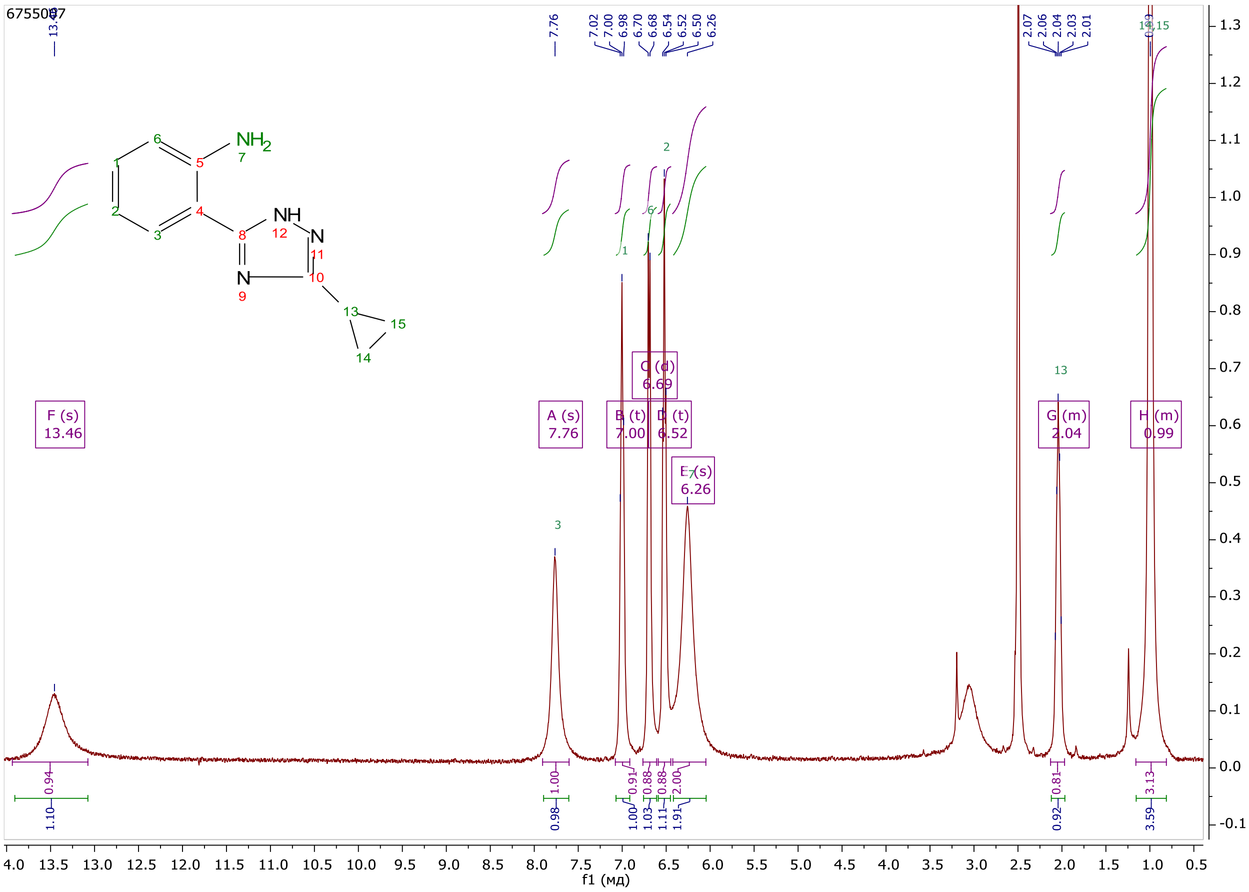

^{13}C NMR (125 MHz) spectrum of compound 2.1 (DMSO-d6 at 298K).

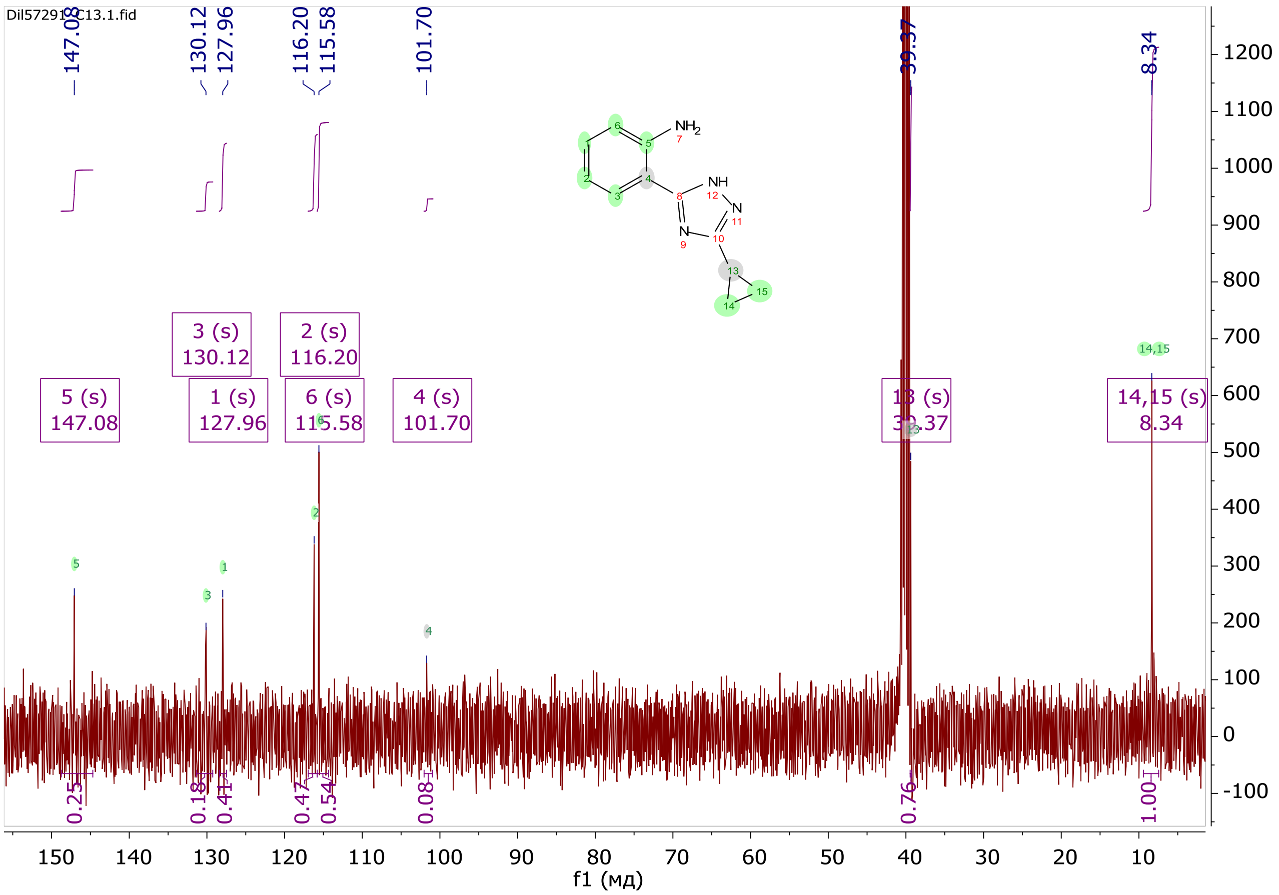

^{1}H NMR (500 MHz) spectrum of compound 2.2 (10 mM in DMSO-d6 at 298K).

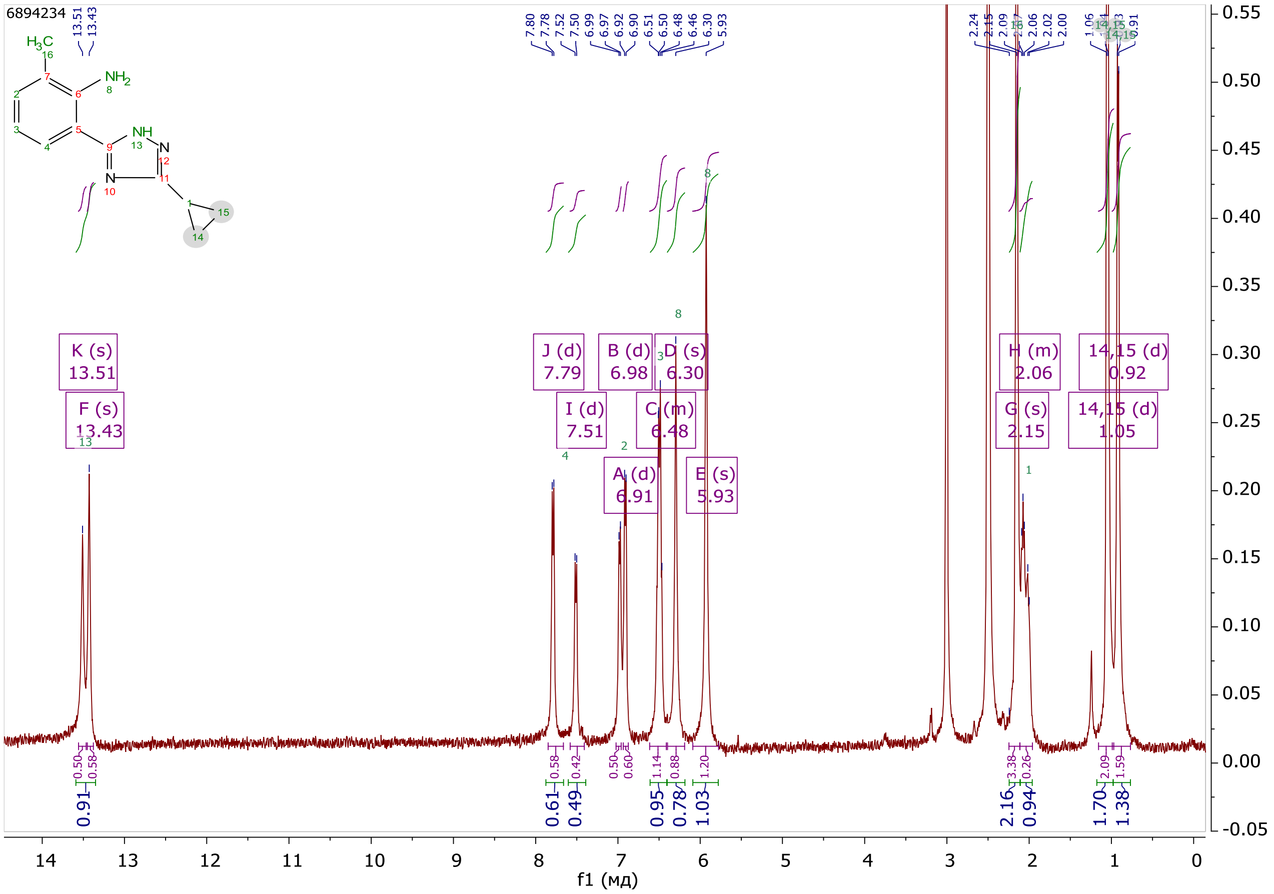

HPLC MS (methanol-water, APCI) data of compound 2.3

MaxPeak: 100.00%
Ret_Time: 1.019 min

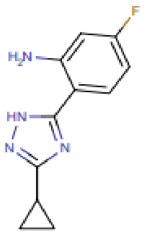

Mol Wt 218.23
Exact Mass 218.11

#	Time	Area%
1	1.019	100.00

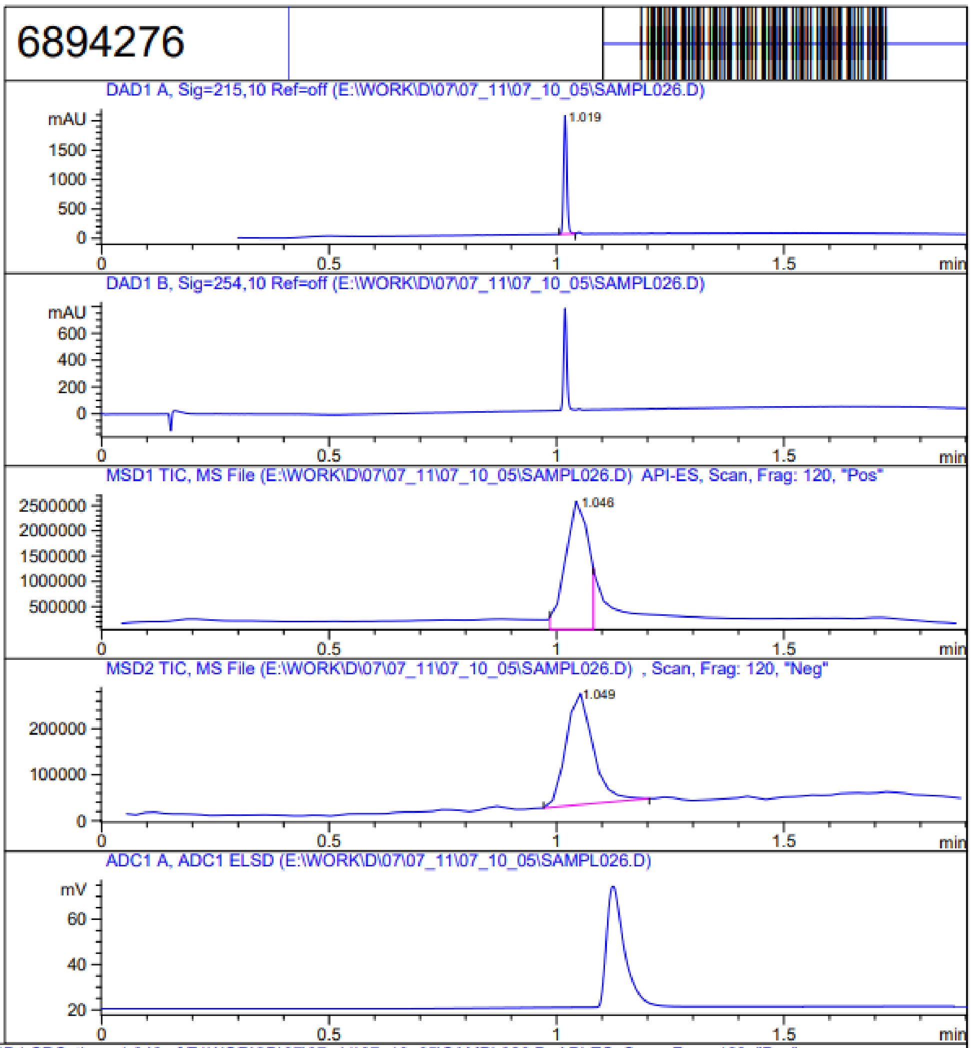

RT 1.046

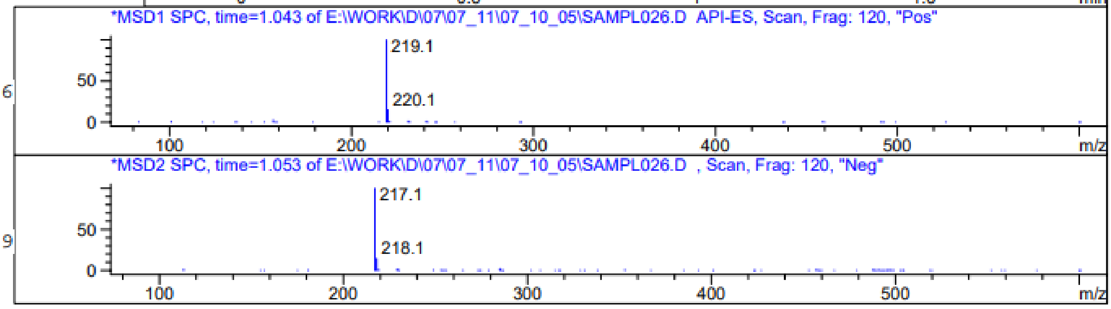

RT 1.049

^{1}H NMR (500 MHz) spectrum of compound 2.3 (10 mM in DMSO-d6 at 298K).

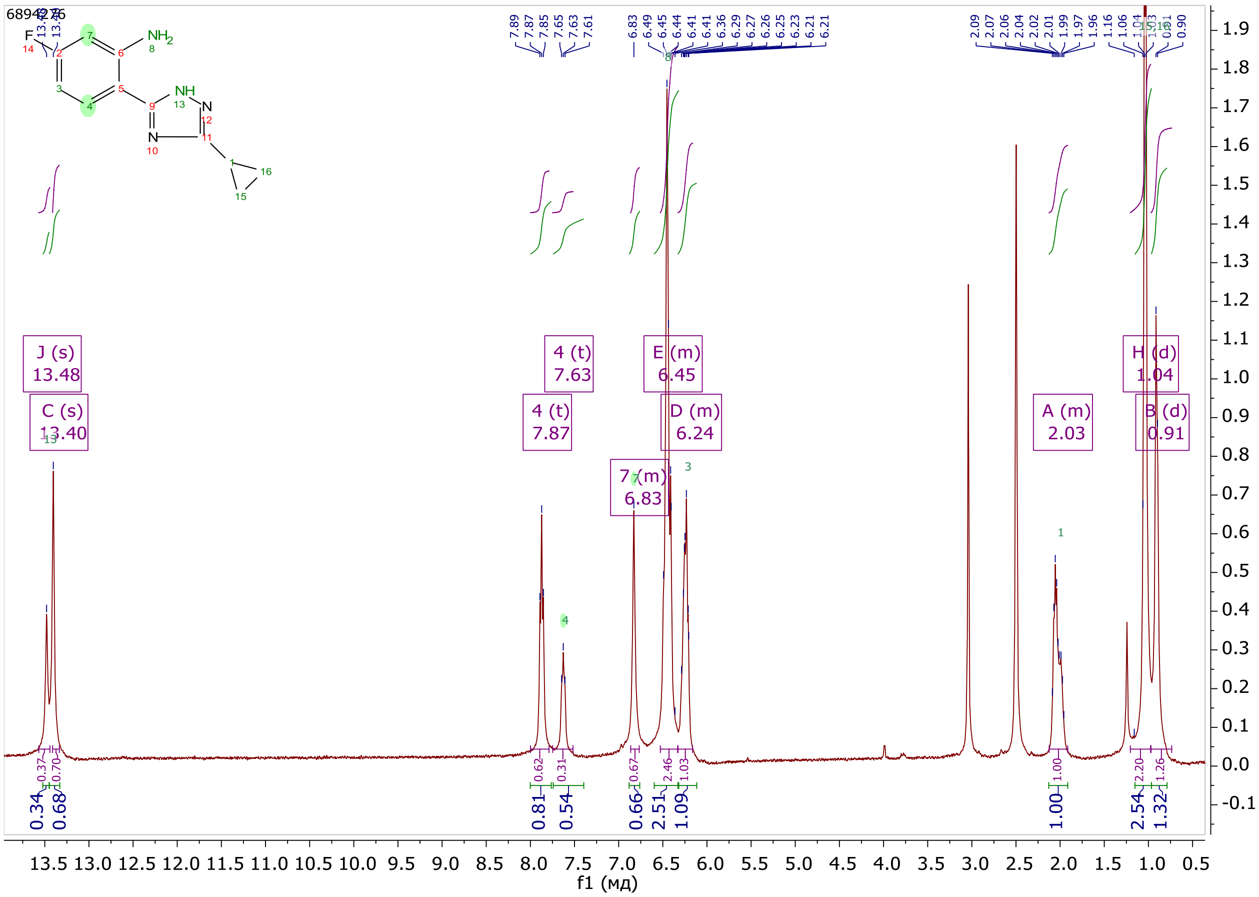

HPLC MS (methanol-water, APCI) data of compound 2.4

MaxPeak: 97.21%
Ret_Time: 1.125 min

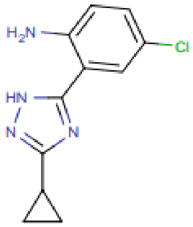

Mol Wt 234.68
Exact Mass 234.08

#	Time	Area%
1	1.125	97.21
2	1.160	1.53
3	1.179	1.26

RT 1.150

RT 1.158

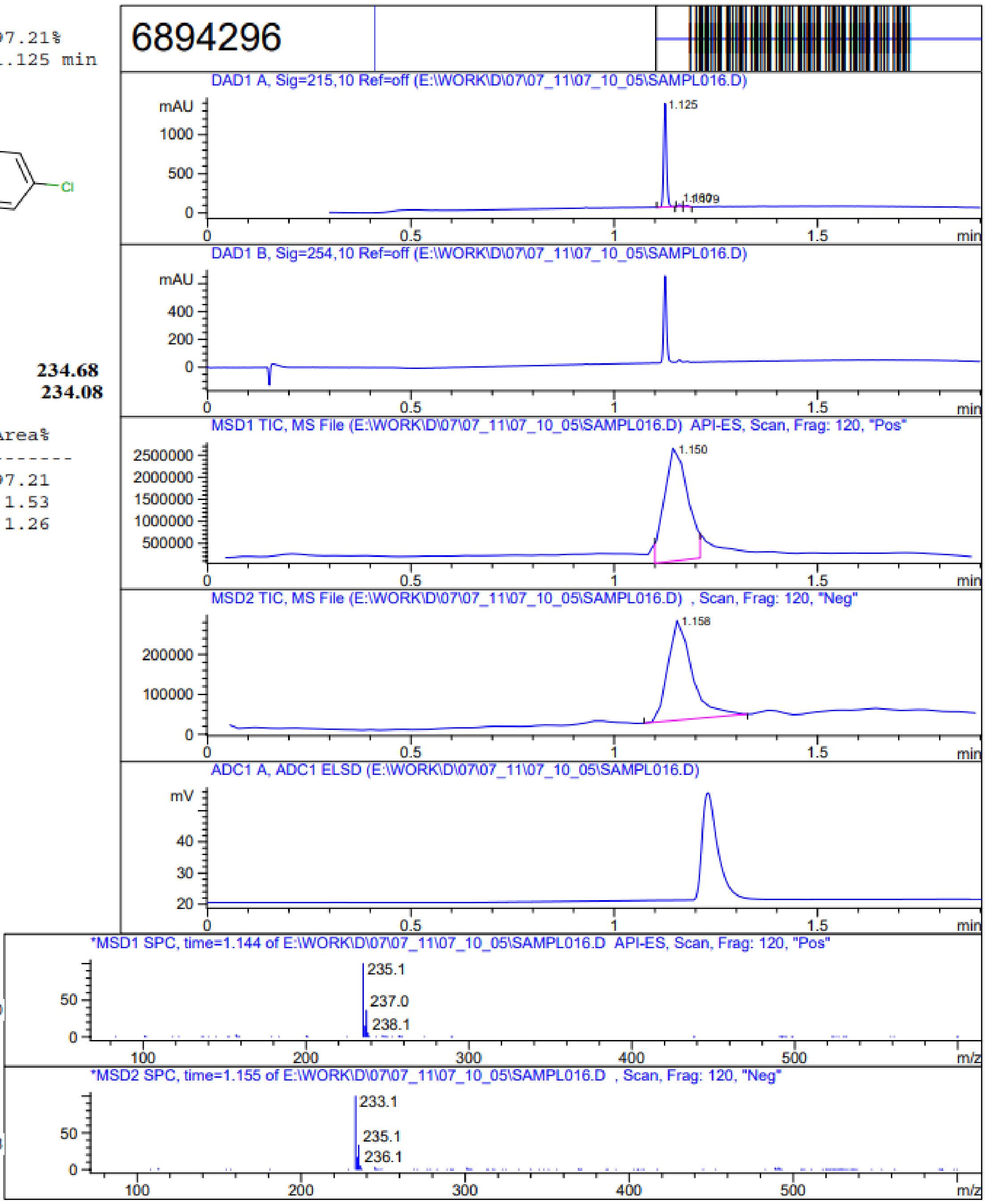

^{1}H NMR (500 MHz) spectrum of compound 2.4 (10 mM in DMSO-d6 at 298K).

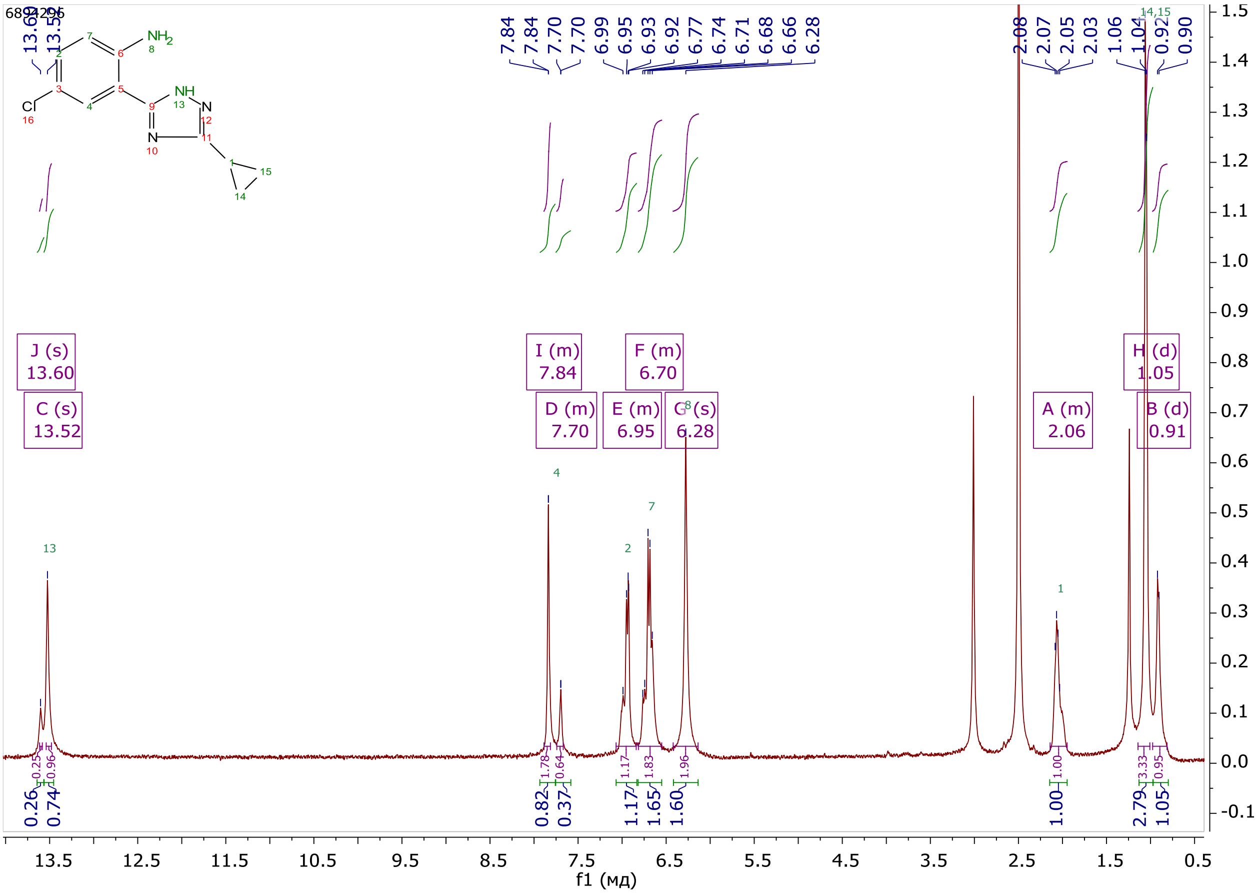

HPLC MS (methanol-water, APCI) data of compound 2.5

```
MaxPeak:  100.00%
Ret_Time: 1.016 min
```

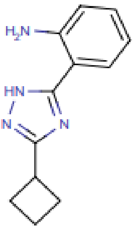

Mol Wt 214.27
Exact Mass 214.14

#	Time	Area%
1	1.016	100.00

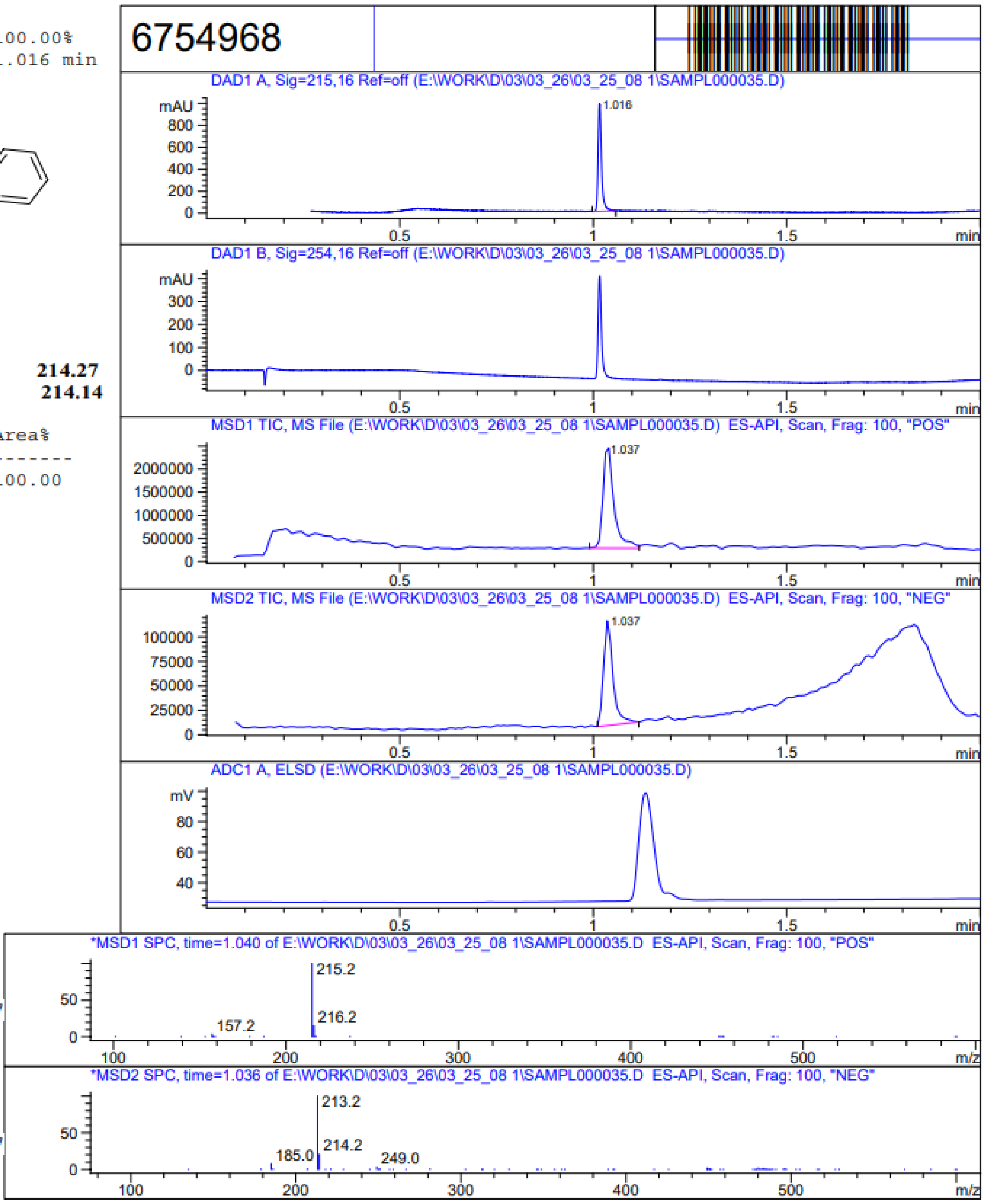

¹H NMR (500 MHz) spectrum of compound 2.5 (10 mM in DMSO-d6 at 298K).

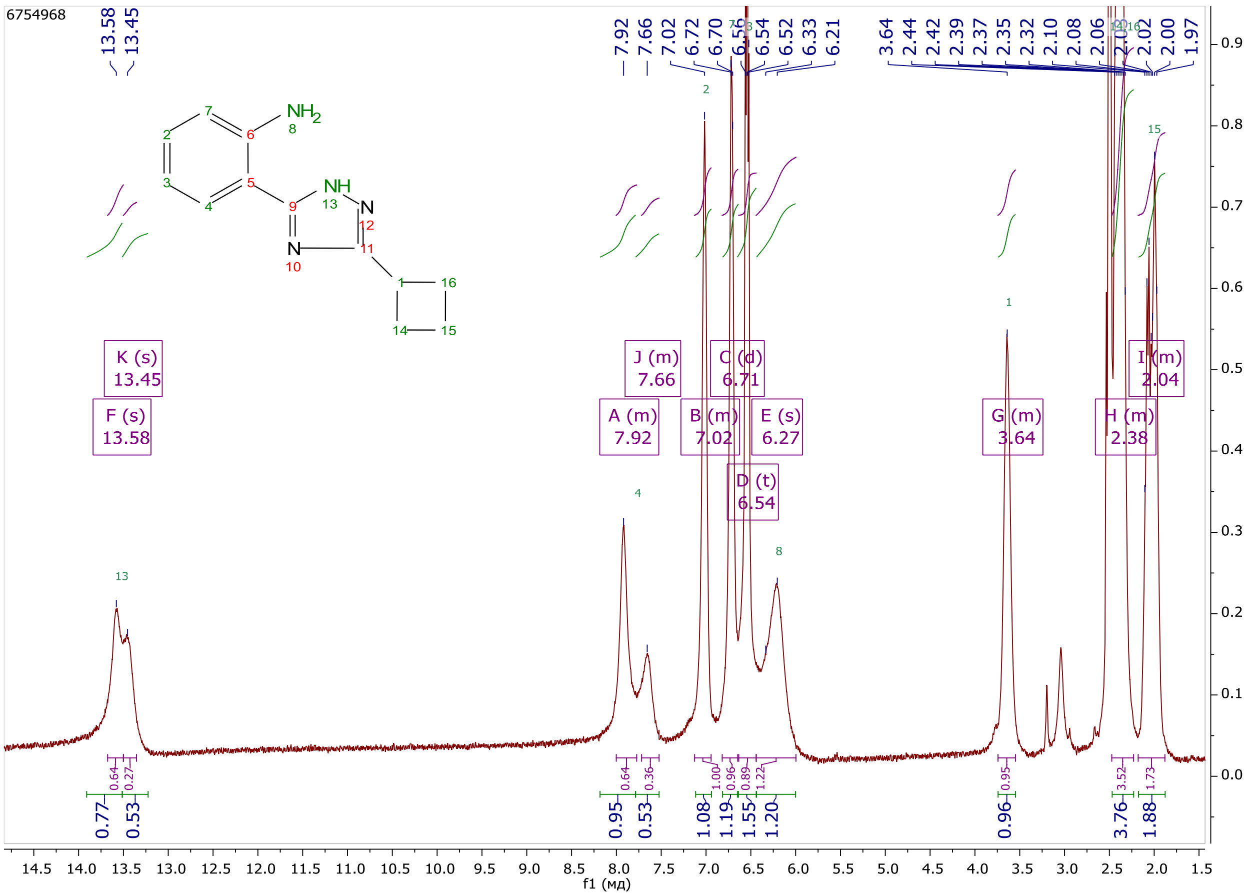

HPLC MS (methanol-water, APCI) data of compound 2.6

MaxPeak: 100.00%
Ret_Time: 1.083 min

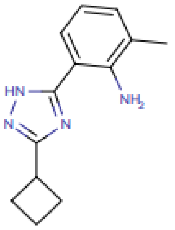

Mol Wt 228.29
Exact Mass 228.16

#	Time	Area%
1	1.083	100.00

RT 1.109

RT 1.117

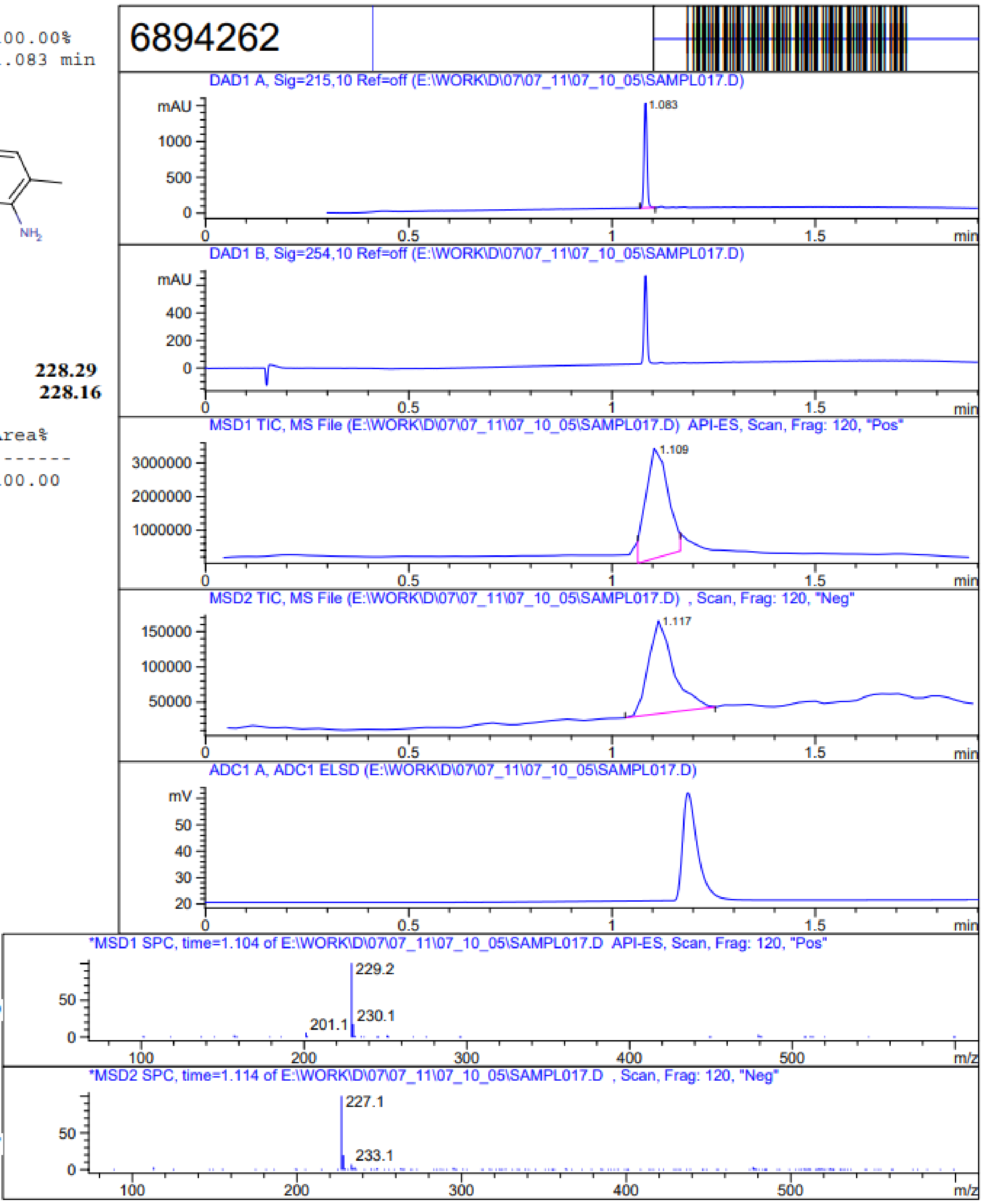

^{1}H NMR (500 MHz) spectrum of compound 2.6 (10 mM in DMSO-d6 at 298K).

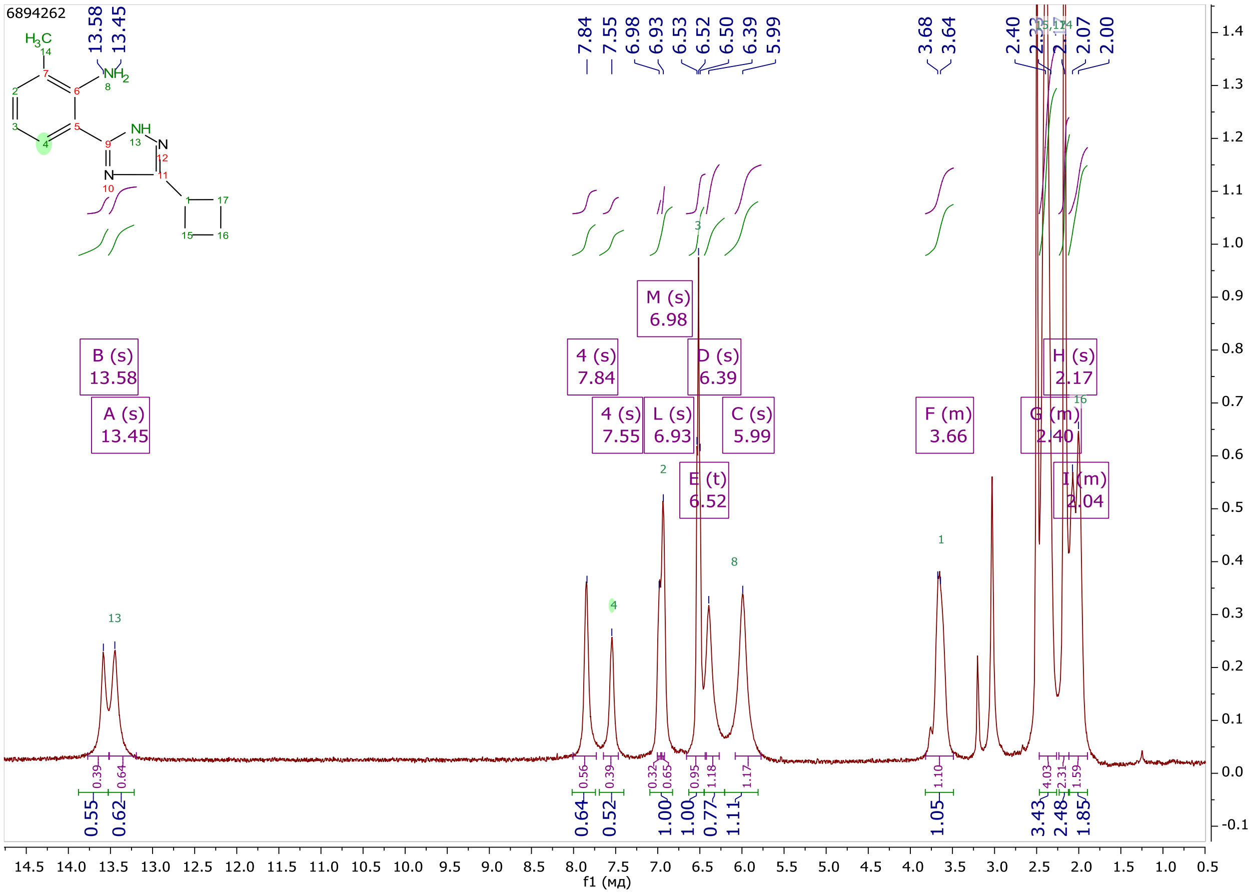

HPLC MS (methanol-water, APCI) data of compound 2.7

MaxPeak: 97.80%
Ret_Time: 1.097 min

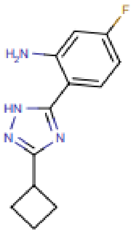

Mol Wt 232.26
Exact Mass 232.13

#	Time	Area%
1	1.097	97.80
2	1.126	2.20

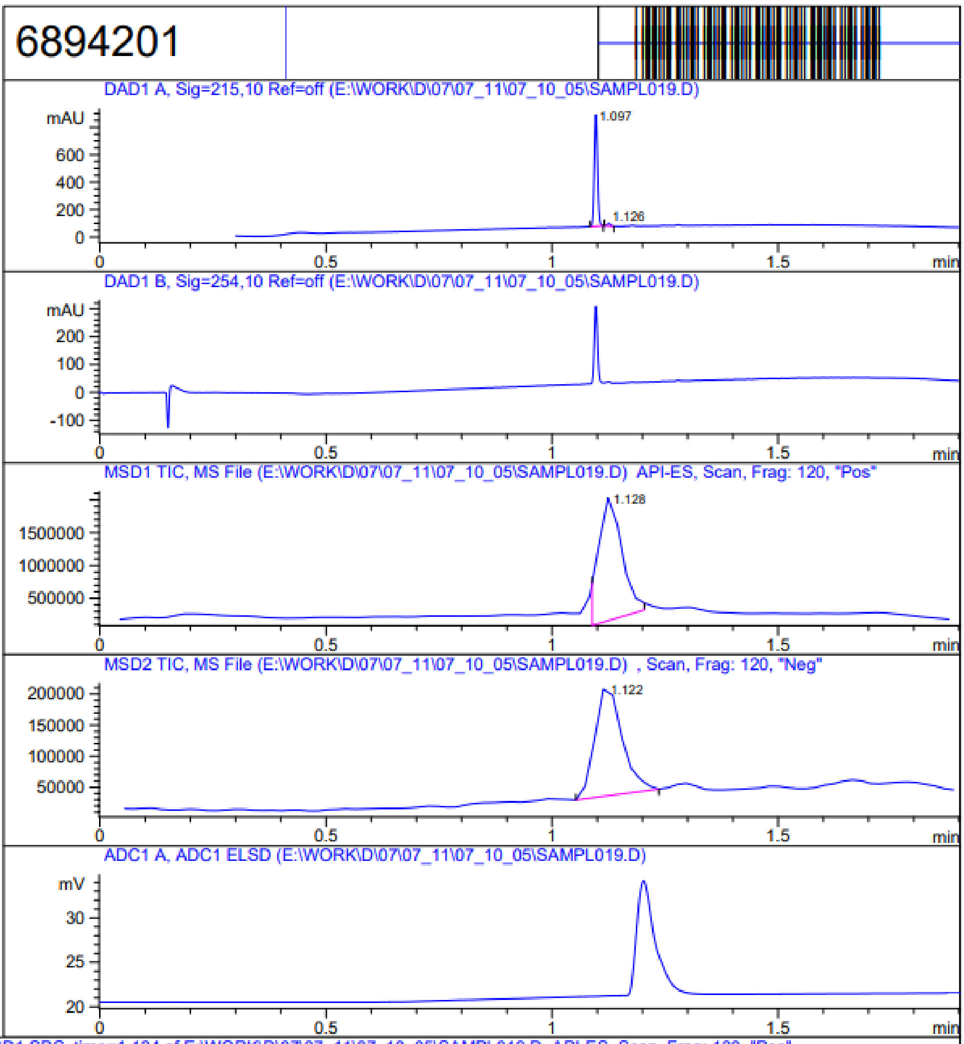

RT 1.128

RT 1.122

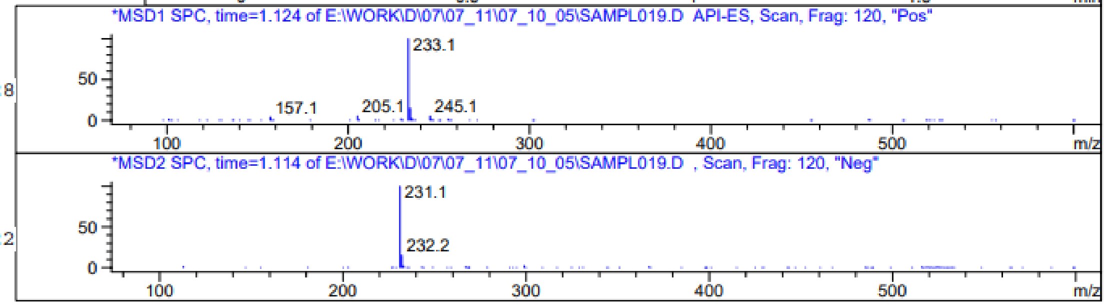

^{1}H NMR (500 MHz) spectrum of compound 2.7 (10 mM in DMSO-d6 at 298K).

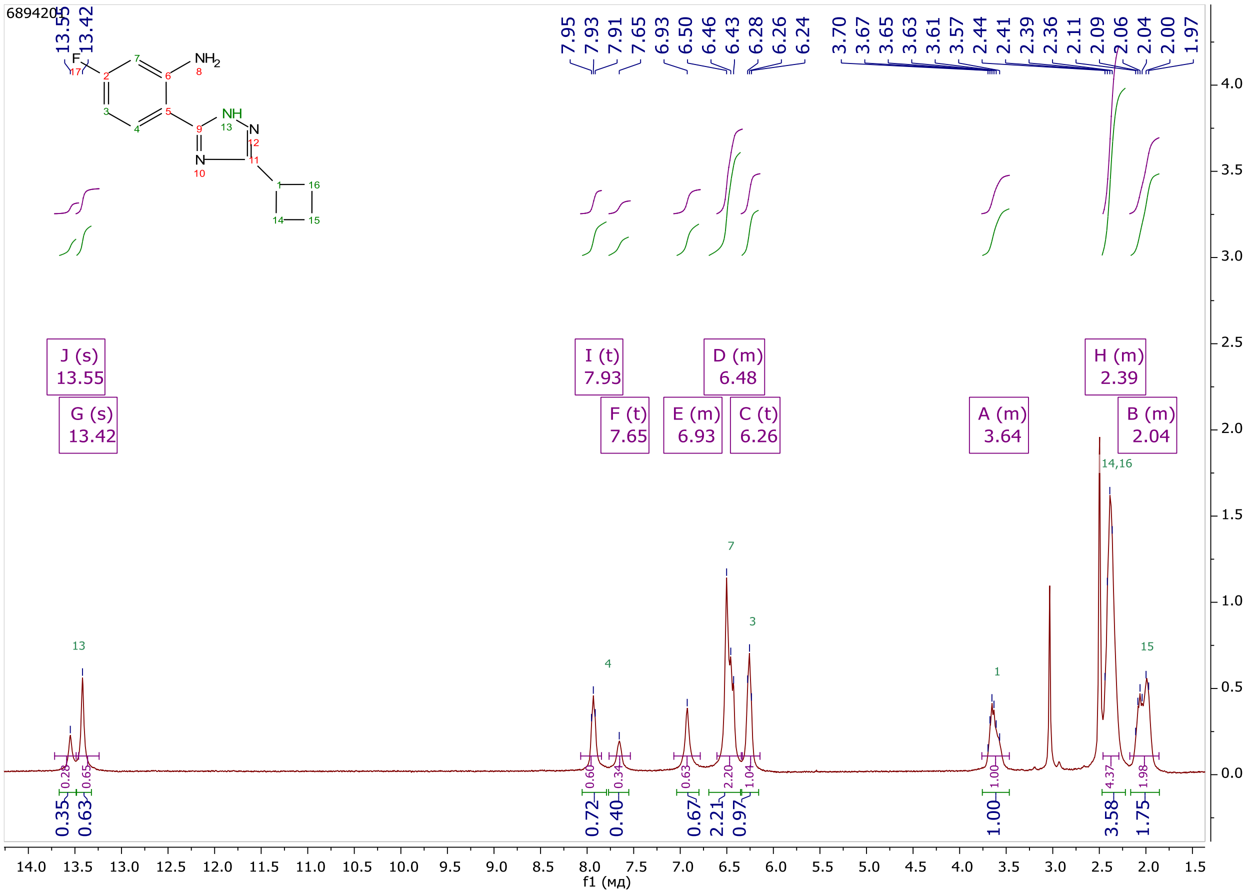

HPLC MS (methanol-water, APCI) data of compound 2.8

MaxPeak: 100.00%
Ret_Time: 1.205 min

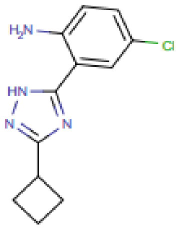

Mol Wt 248.71
Exact Mass 248.1

#	Time	Area%
1	1.205	100.00

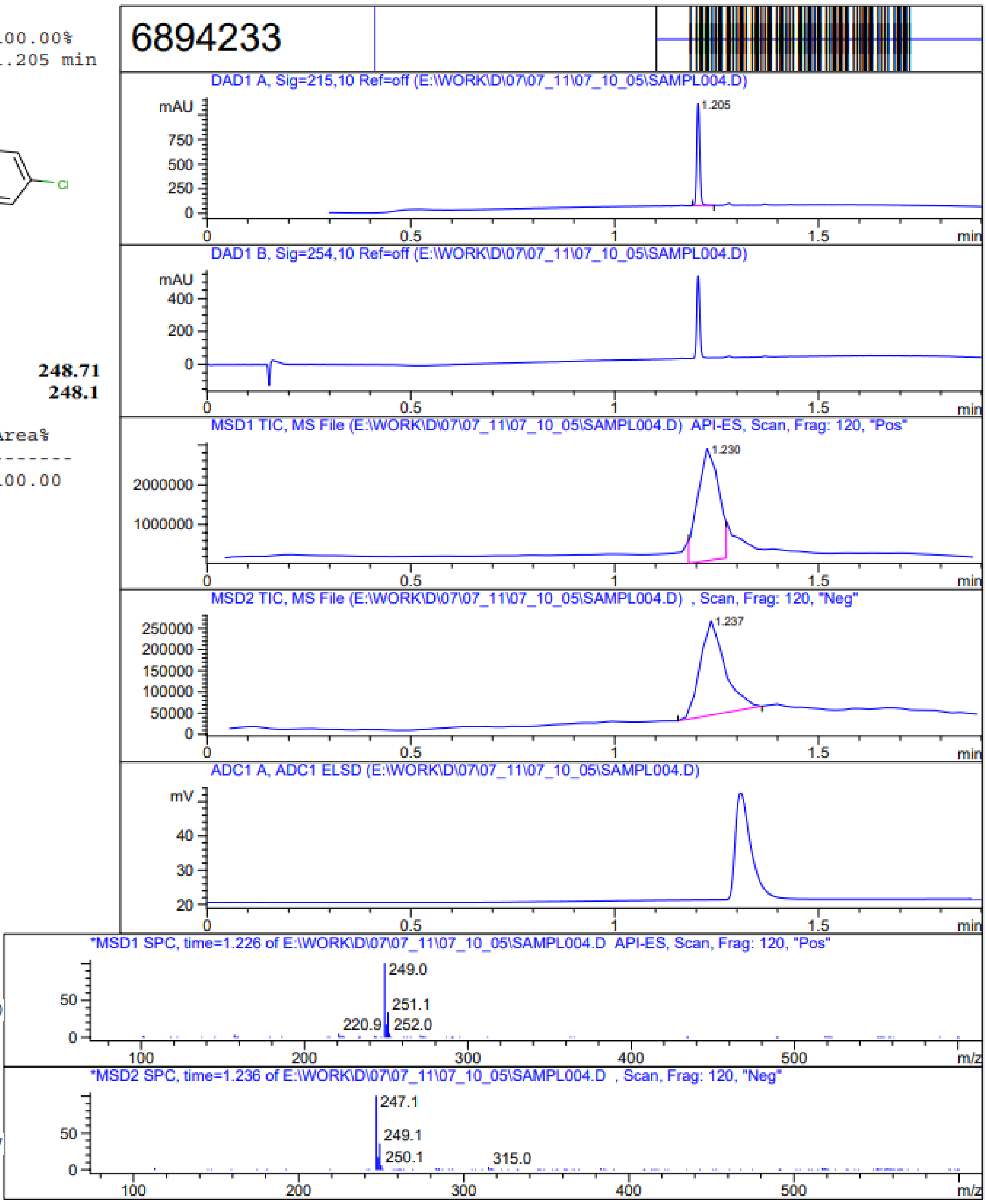

RT 1.230

RT 1.237

^{1}H NMR (500 MHz) spectrum of compound 2.8 (10 mM in DMSO-d6 at 298K).

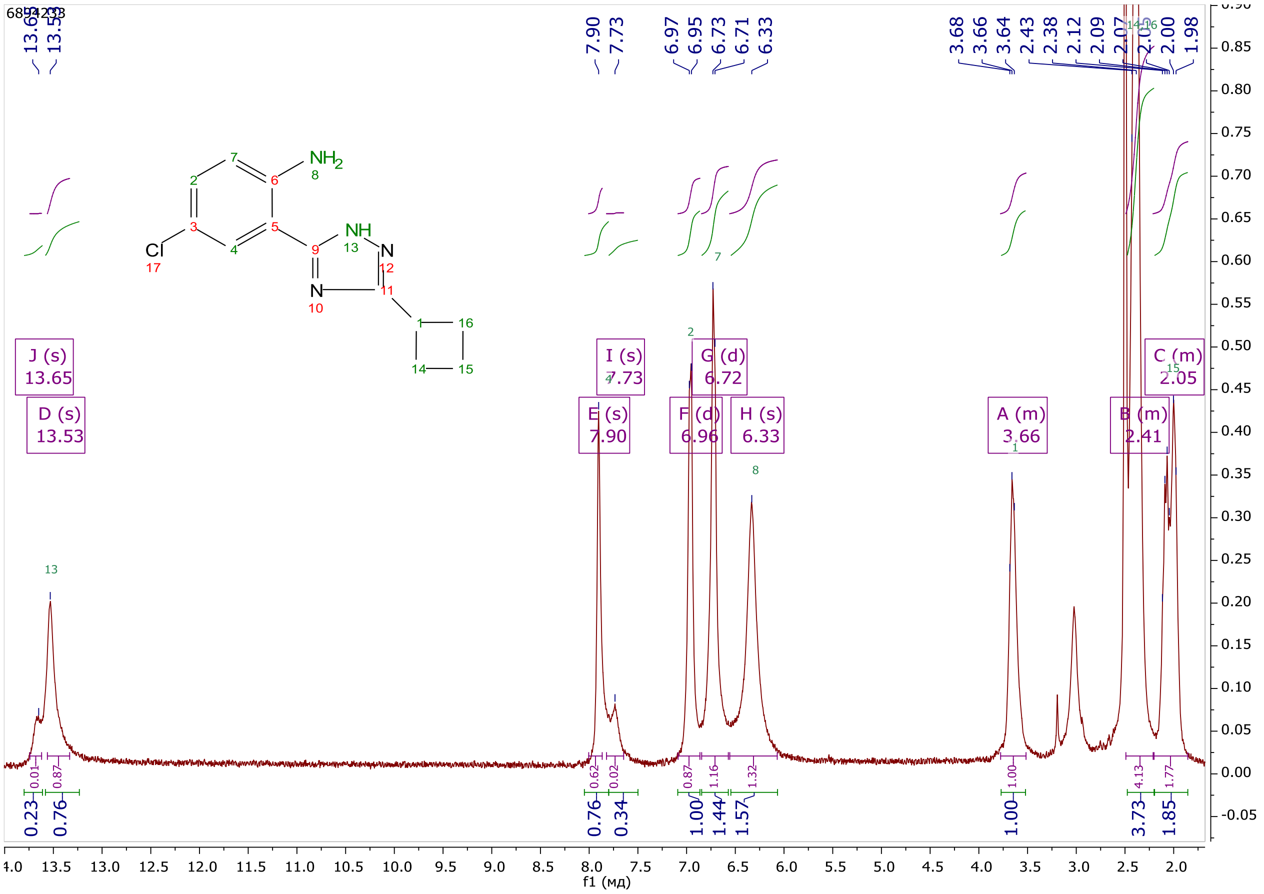

HPLC MS (methanol-water, APCI) data of compound 2.9

MaxPeak: 100.00%
Ret_Time: 1.045 min

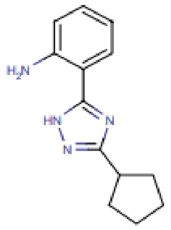

Mol Wt 228.29
Exact Mass 228.16

#	Time	Area%
1	1.045	100.00

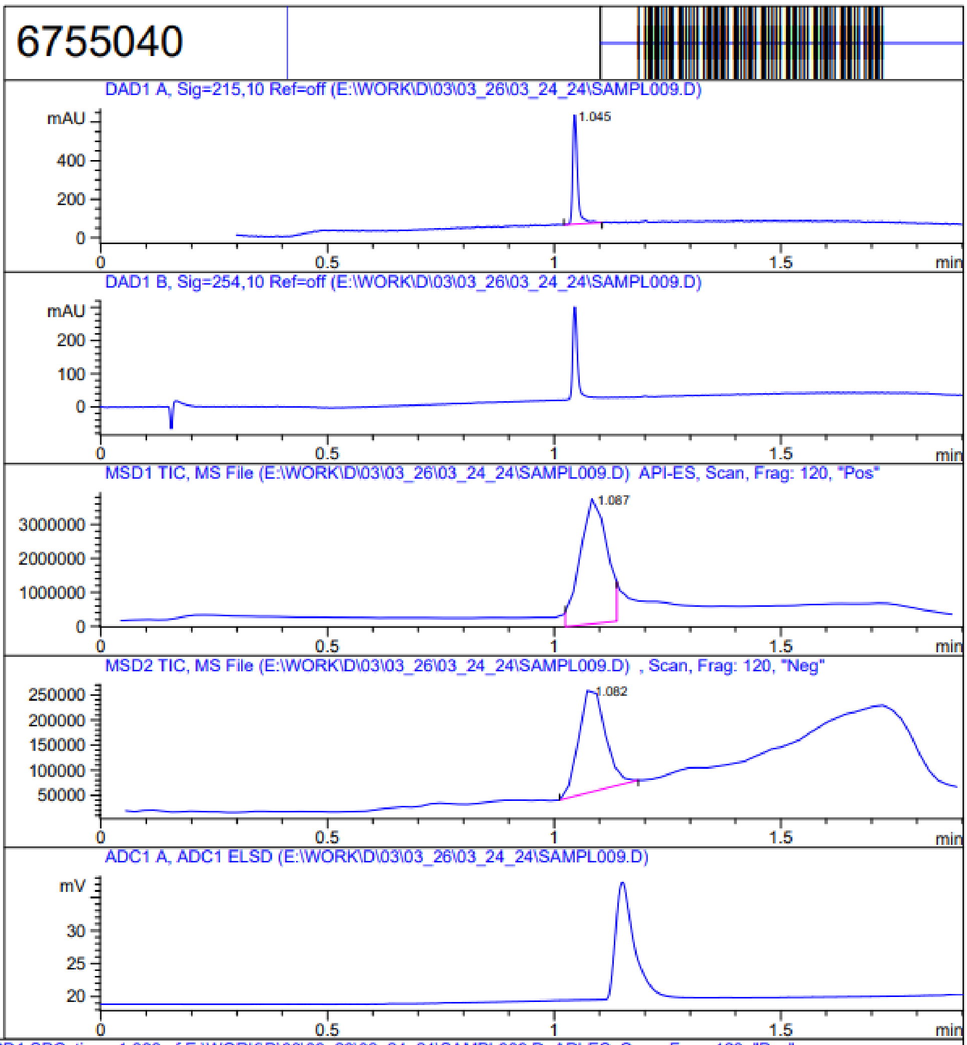

RT 1.087

RT 1.082

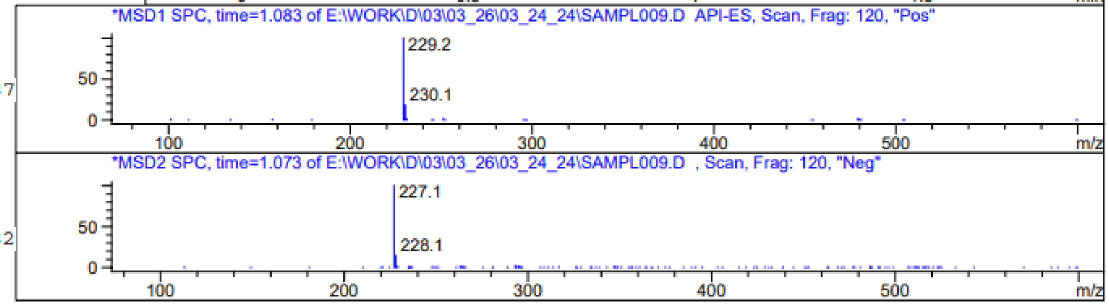

^{1}H NMR (500 MHz) spectrum of compound 2.9 (10 mM in DMSO-d6 at 298K).

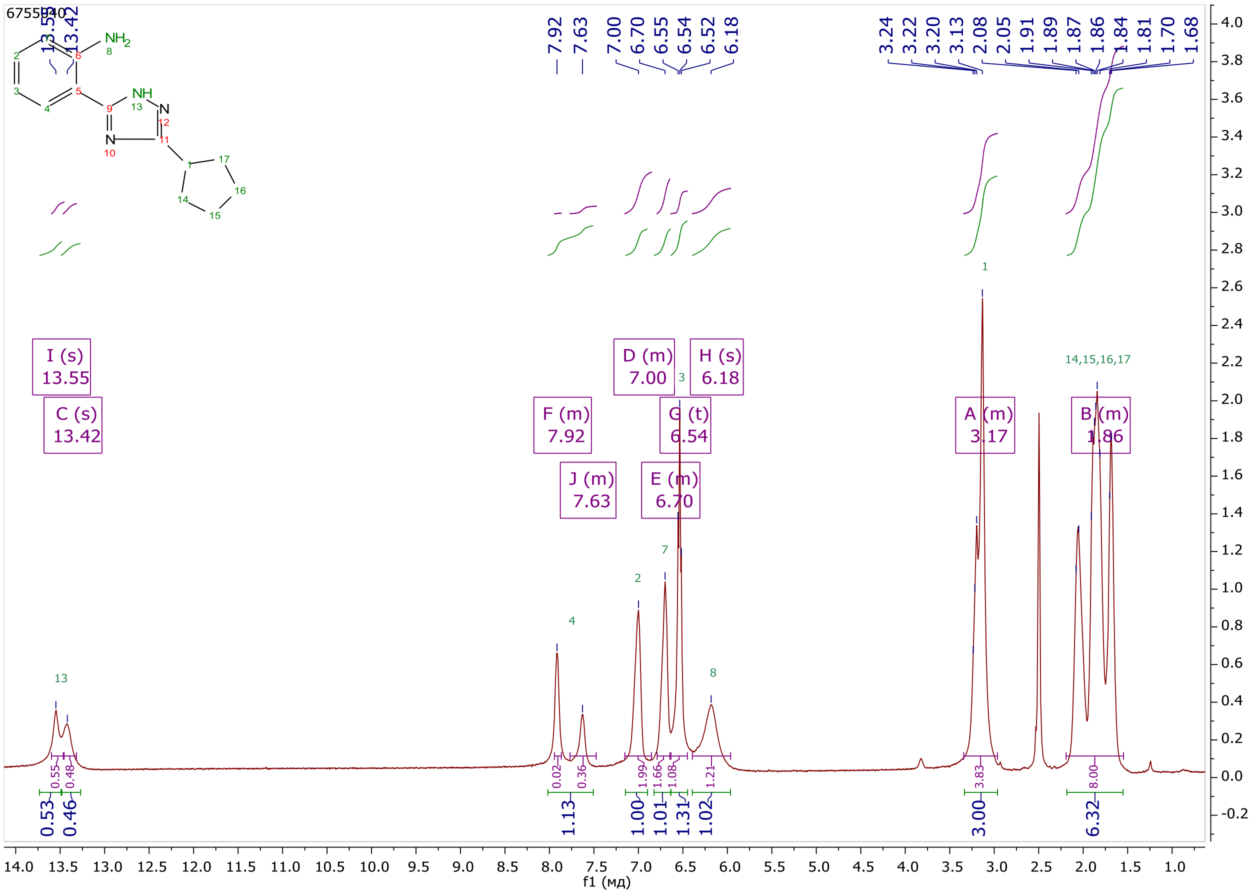

HPLC MS (methanol-water, APCI) data of compound 2.10

MaxPeak: 100.00%
Ret_Time: 1.167 min

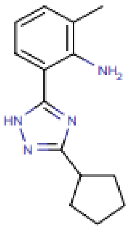

Mol Wt 242.32
Exact Mass 242.18

#	Time	Area%
1	1.167	100.00

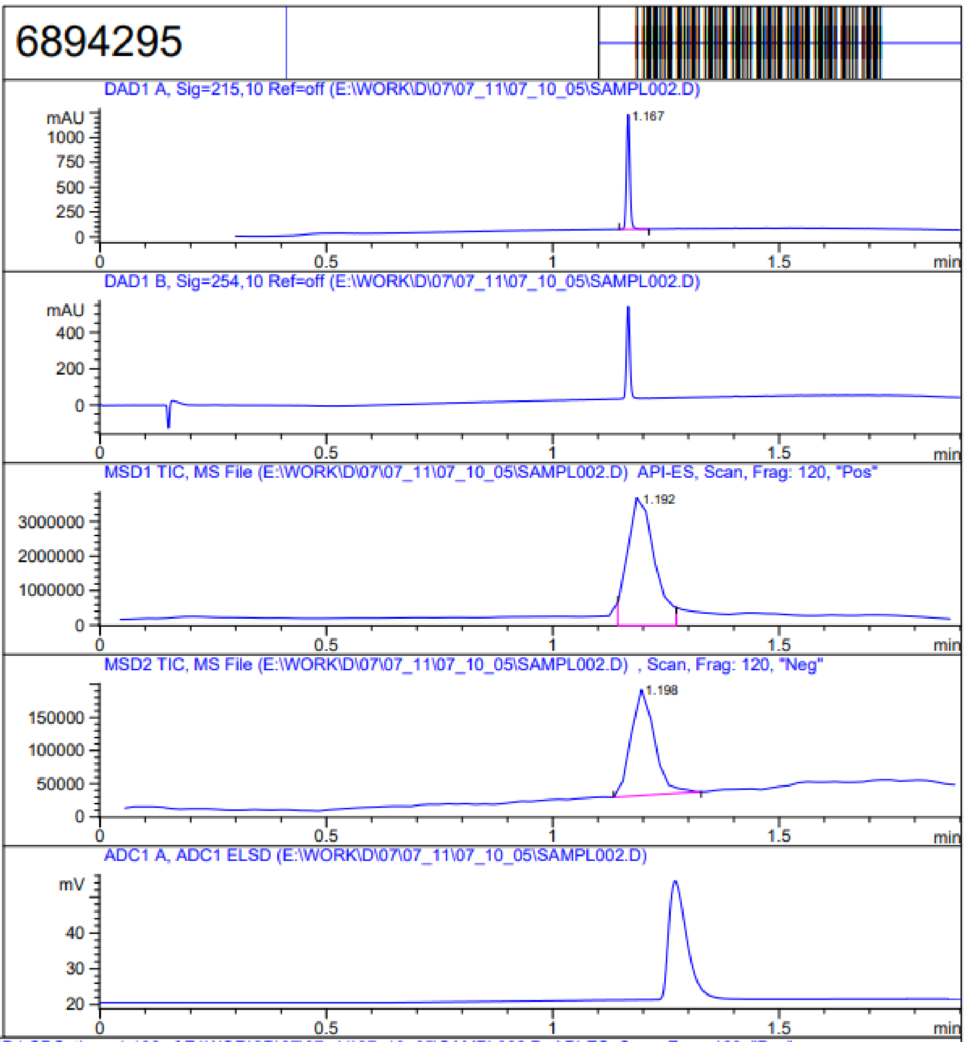

RT 1.192

RT 1.198

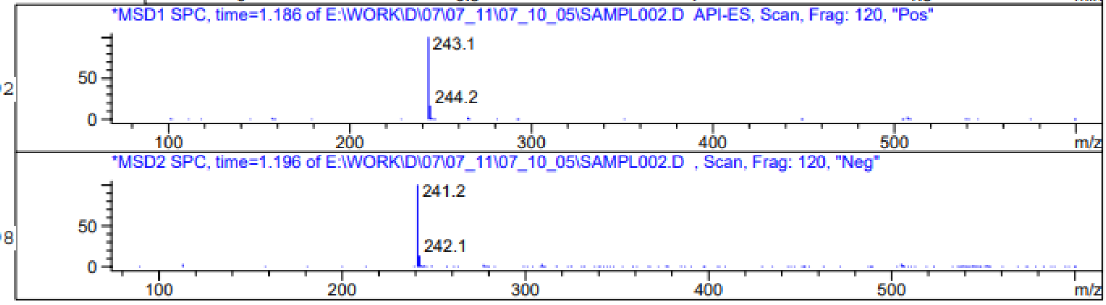

^{1}H NMR (500 MHz) spectrum of compound 2.10 (10 mM in DMSO-d6 at 298K).

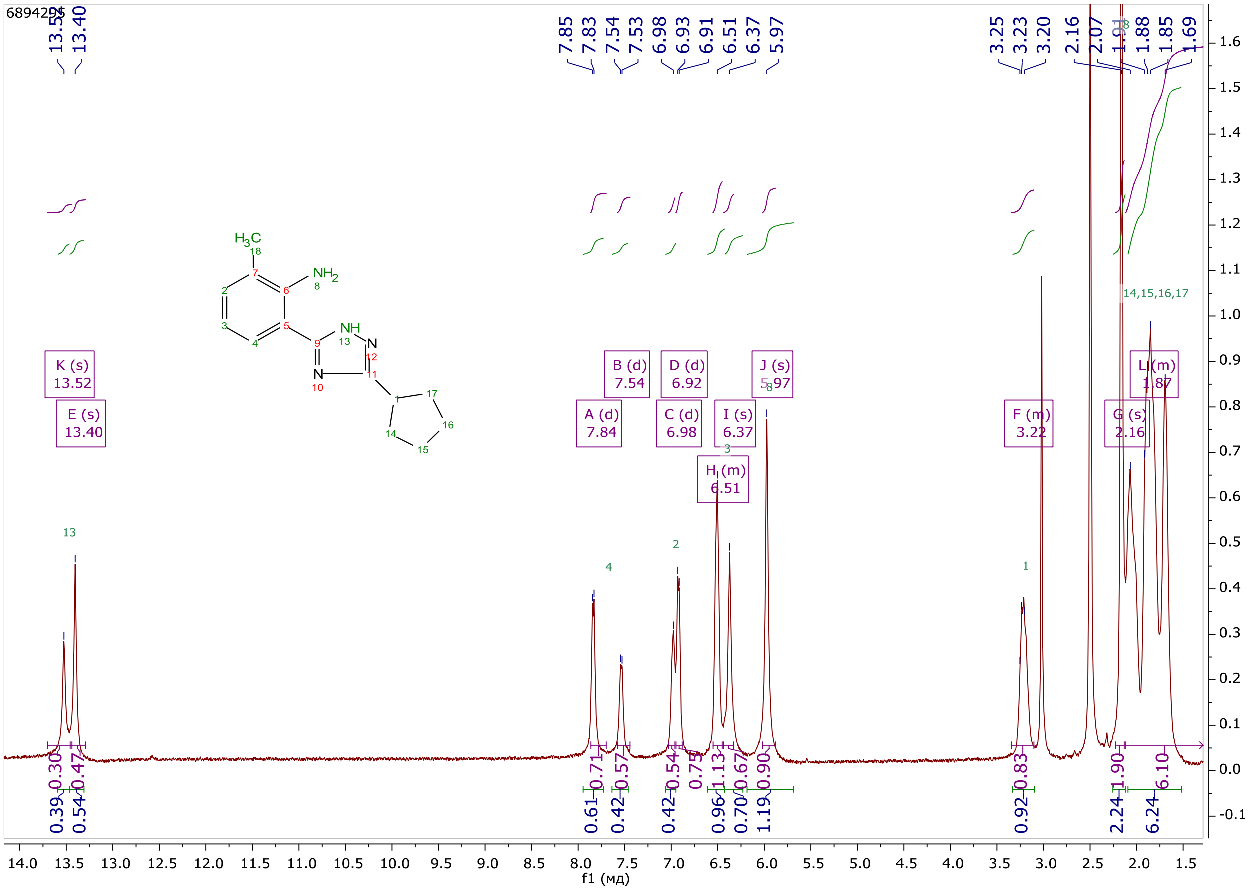

HPLC MS (methanol-water, APCI) data of compound 2.11

MaxPeak: 100.00%
Ret_Time: 1.181 min

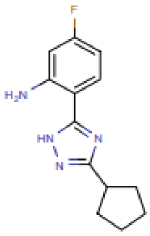

Mol Wt 246.28
Exact Mass 246.15

#	Time	Area%
1	1.181	100.00

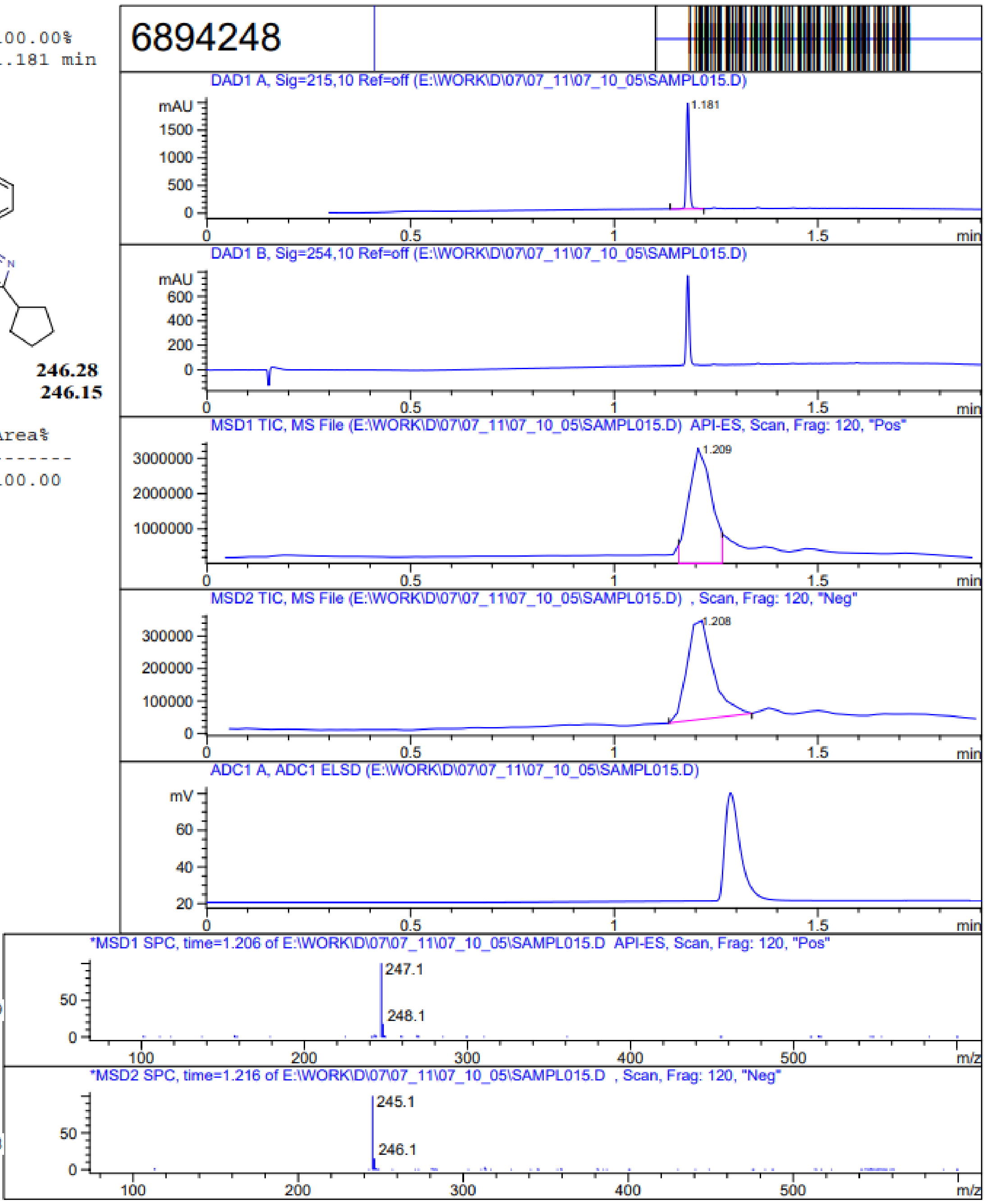

RT 1.209

RT 1.208

^{1}H NMR (500 MHz) spectrum of compound 2.11 (10 mM in DMSO-d6 at 298K).

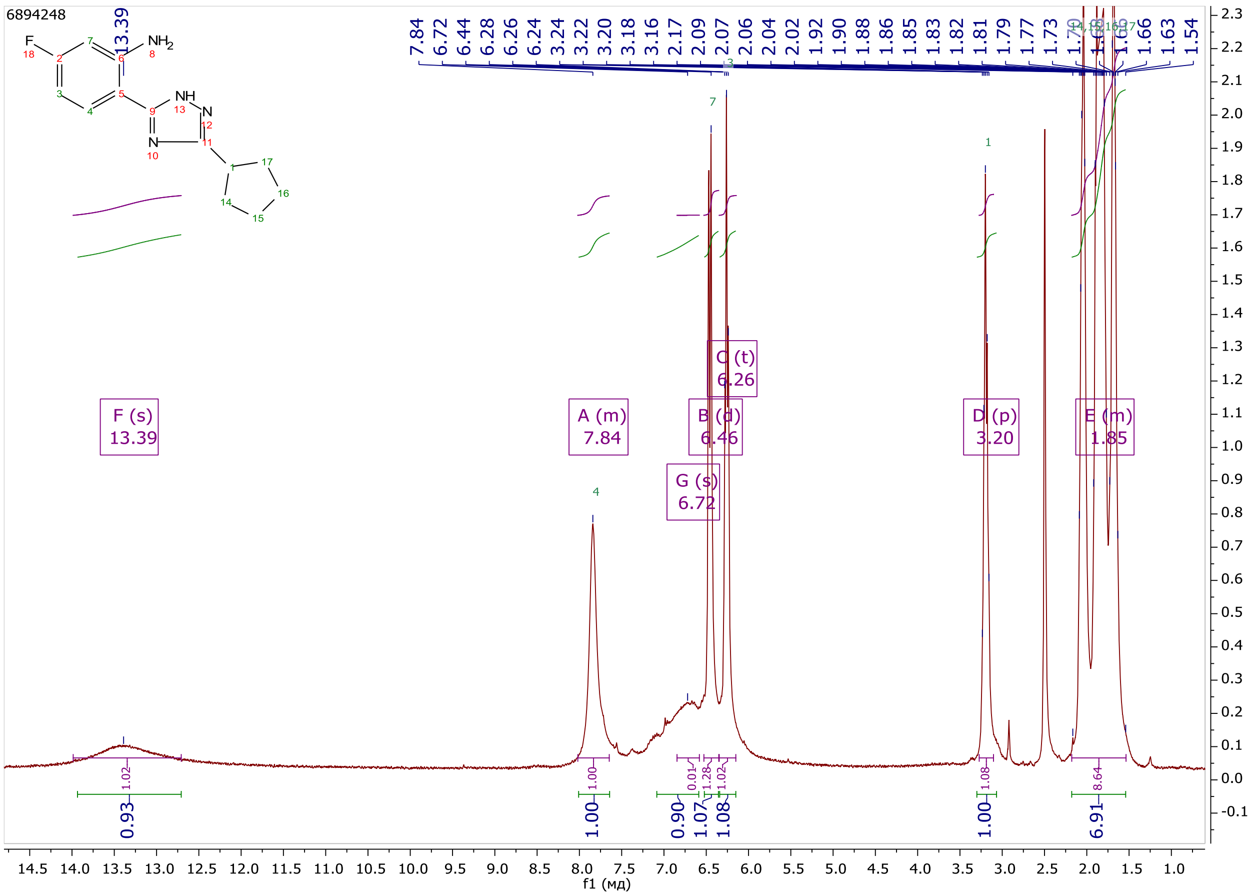

HPLC MS (methanol-water, APCI) data of compound 2.12

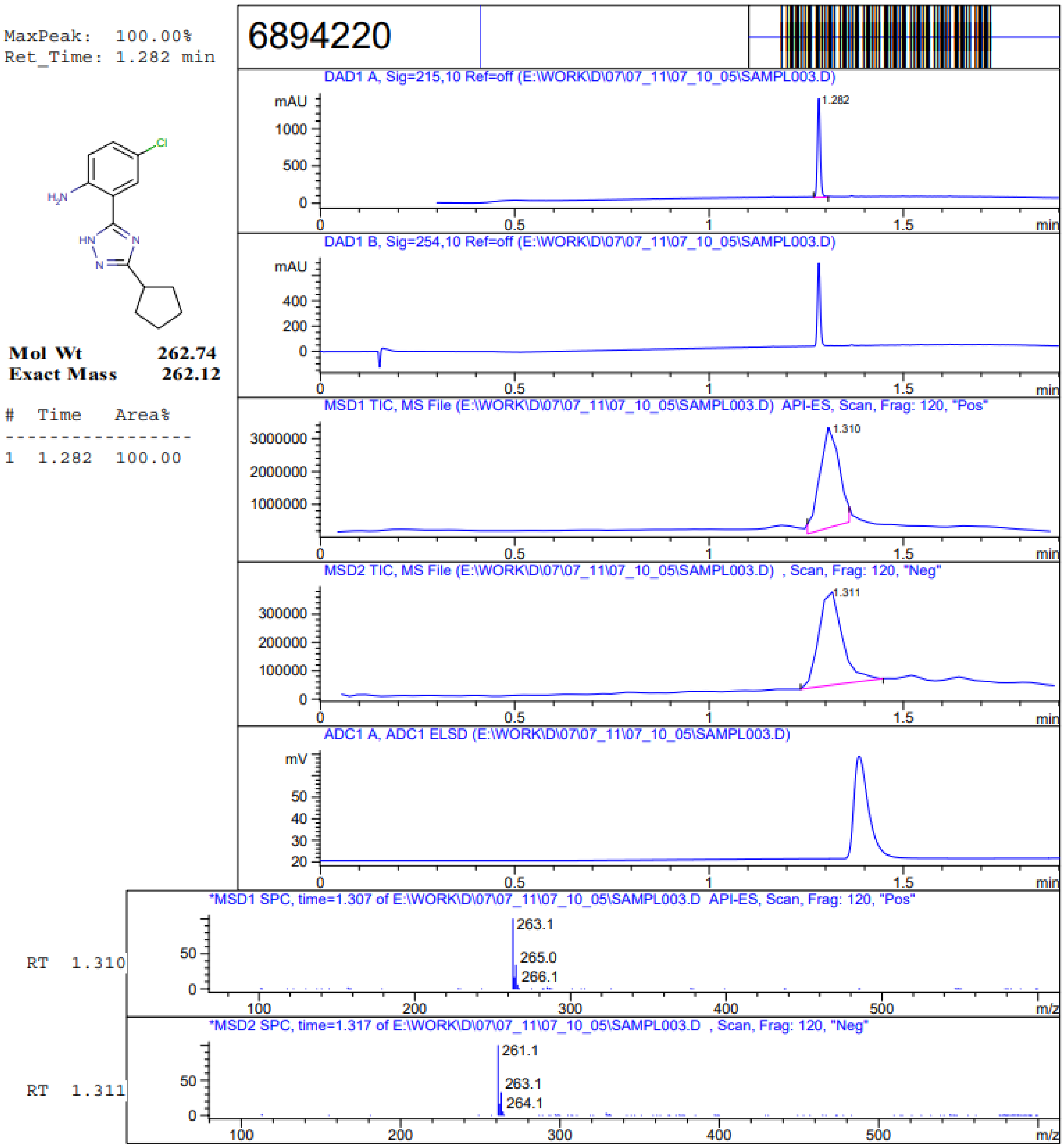

^{1}H NMR (500 MHz) spectrum of compound 2.12 (10 mM in DMSO-d6 at 298K).

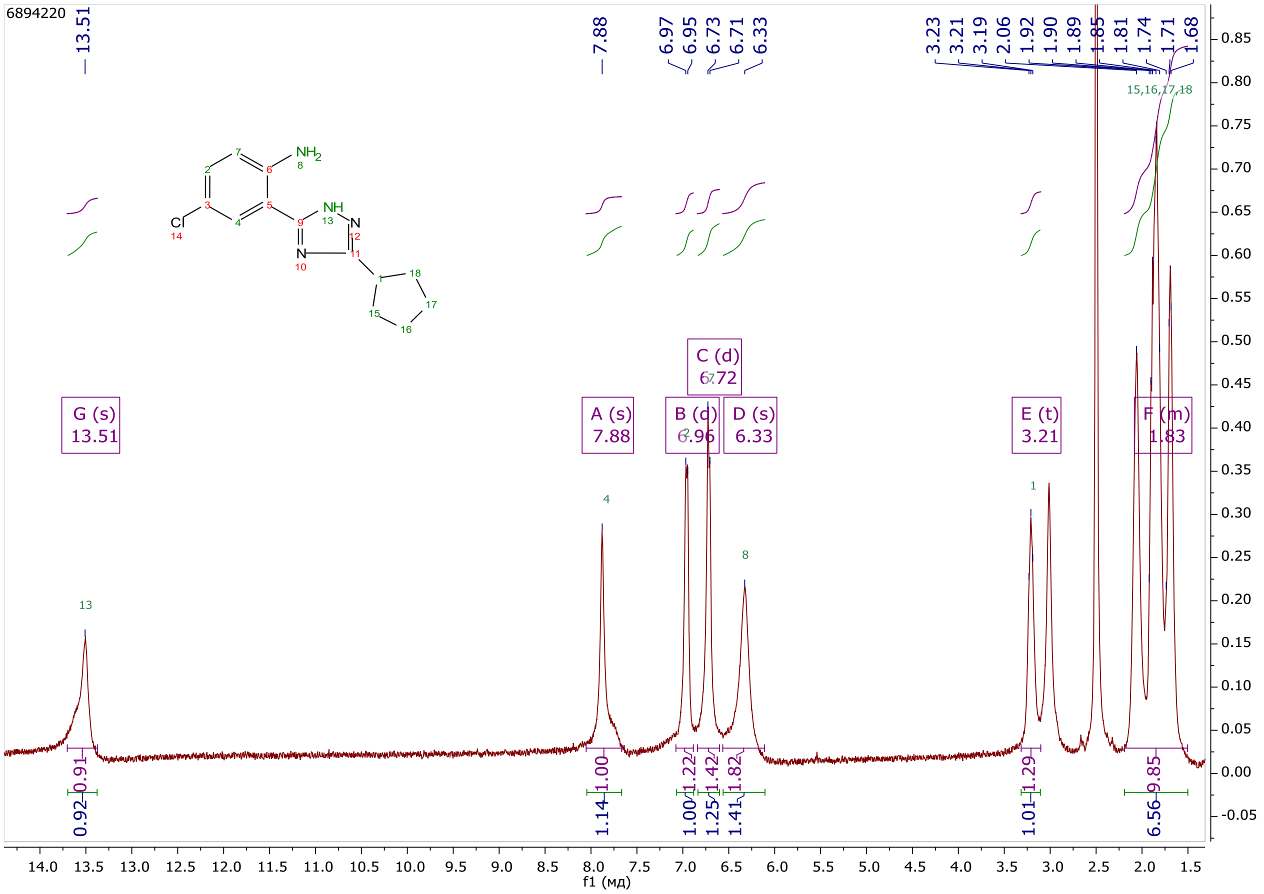

^{1}H NMR (500 MHz) spectrum of compound 2.13 (10 mM in DMSO-d6 at 298K).

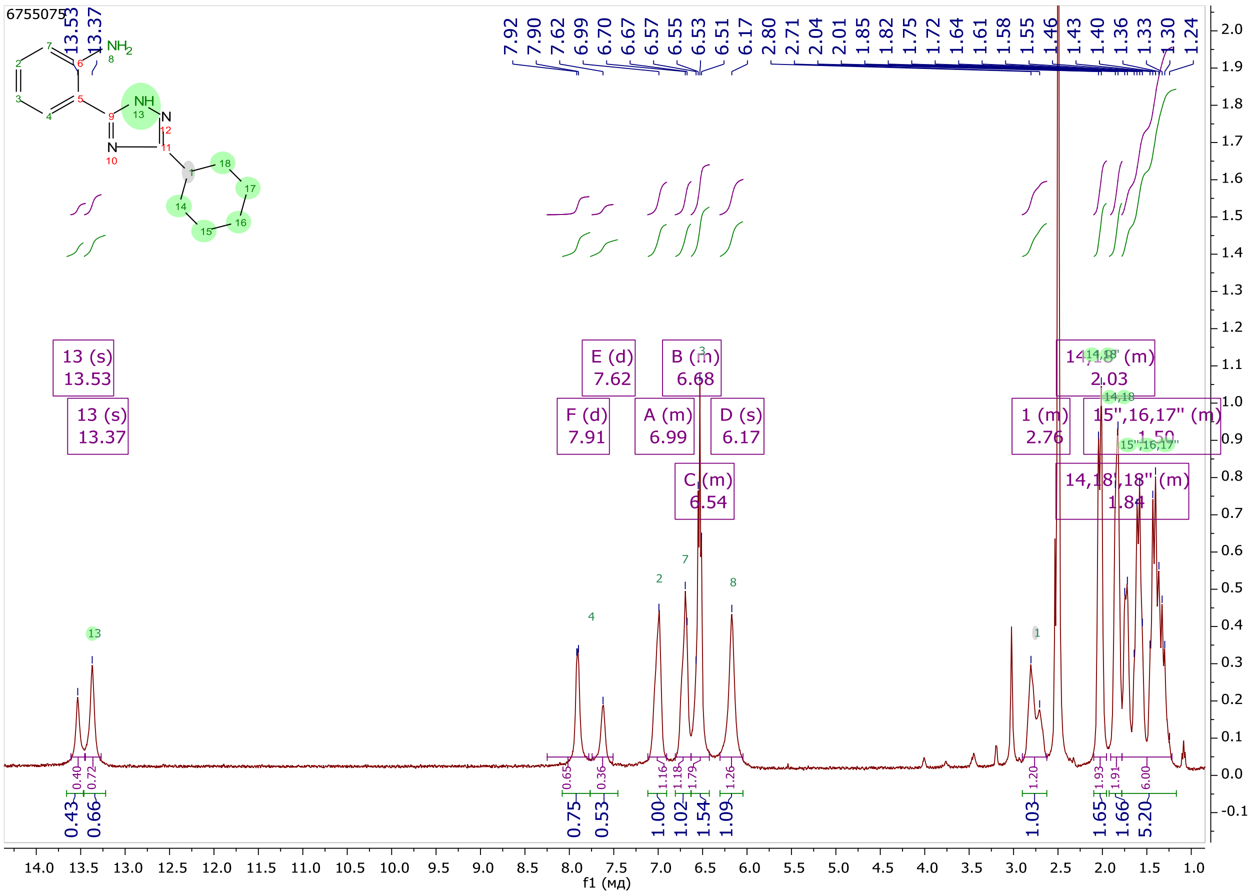

^{13}C NMR (125 MHz) spectrum of compound 2.13 (DMSO-d6 at 298K).

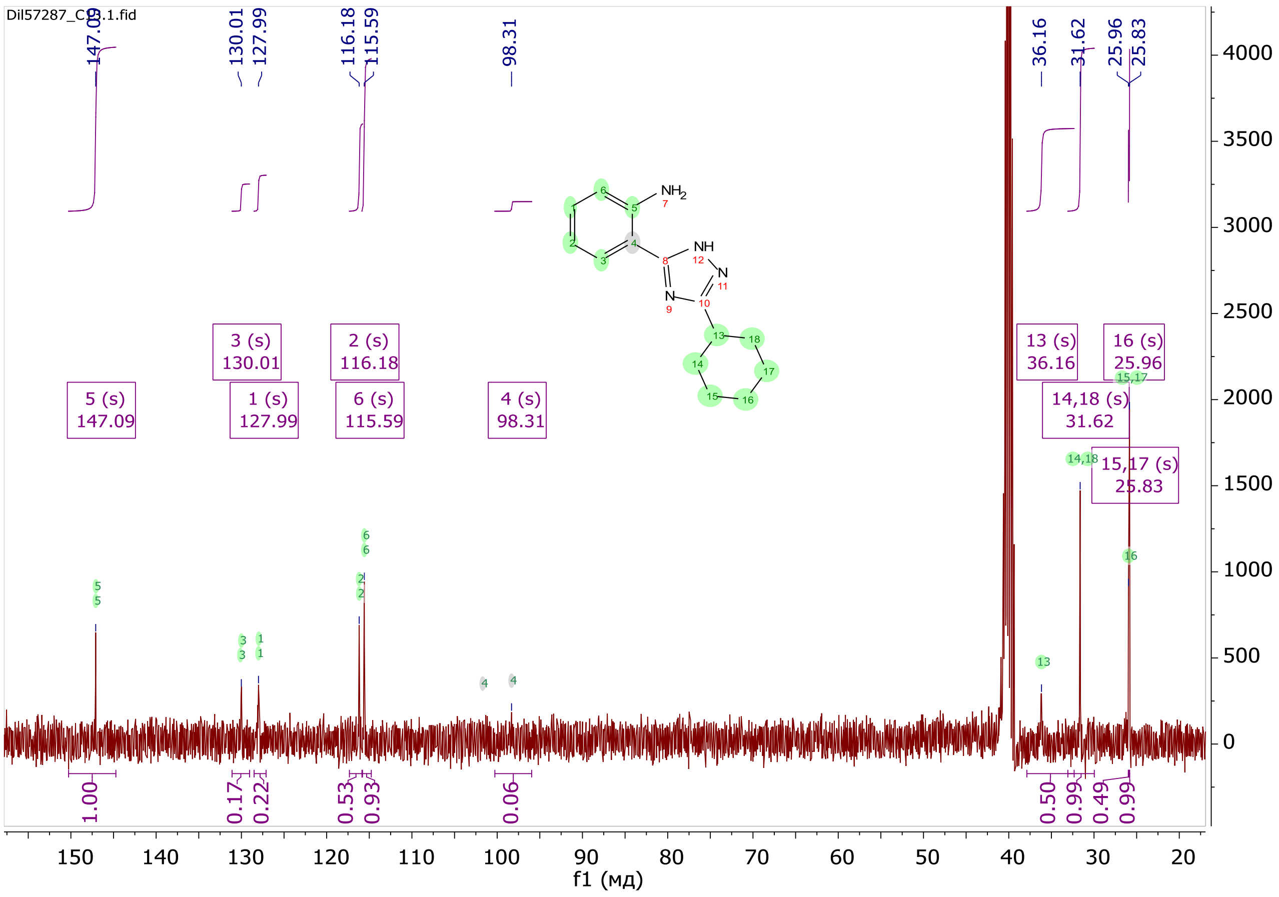

HPLC MS (methanol-water, APCI) data of compound 2.14

MaxPeak: 100.00%
Ret_Time: 1.252 min

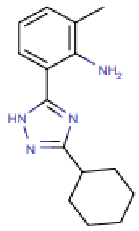

Mol Wt 256.35
Exact Mass 256.2

#	Time	Area%
1	1.252	100.00

RT 1.281

RT 1.279

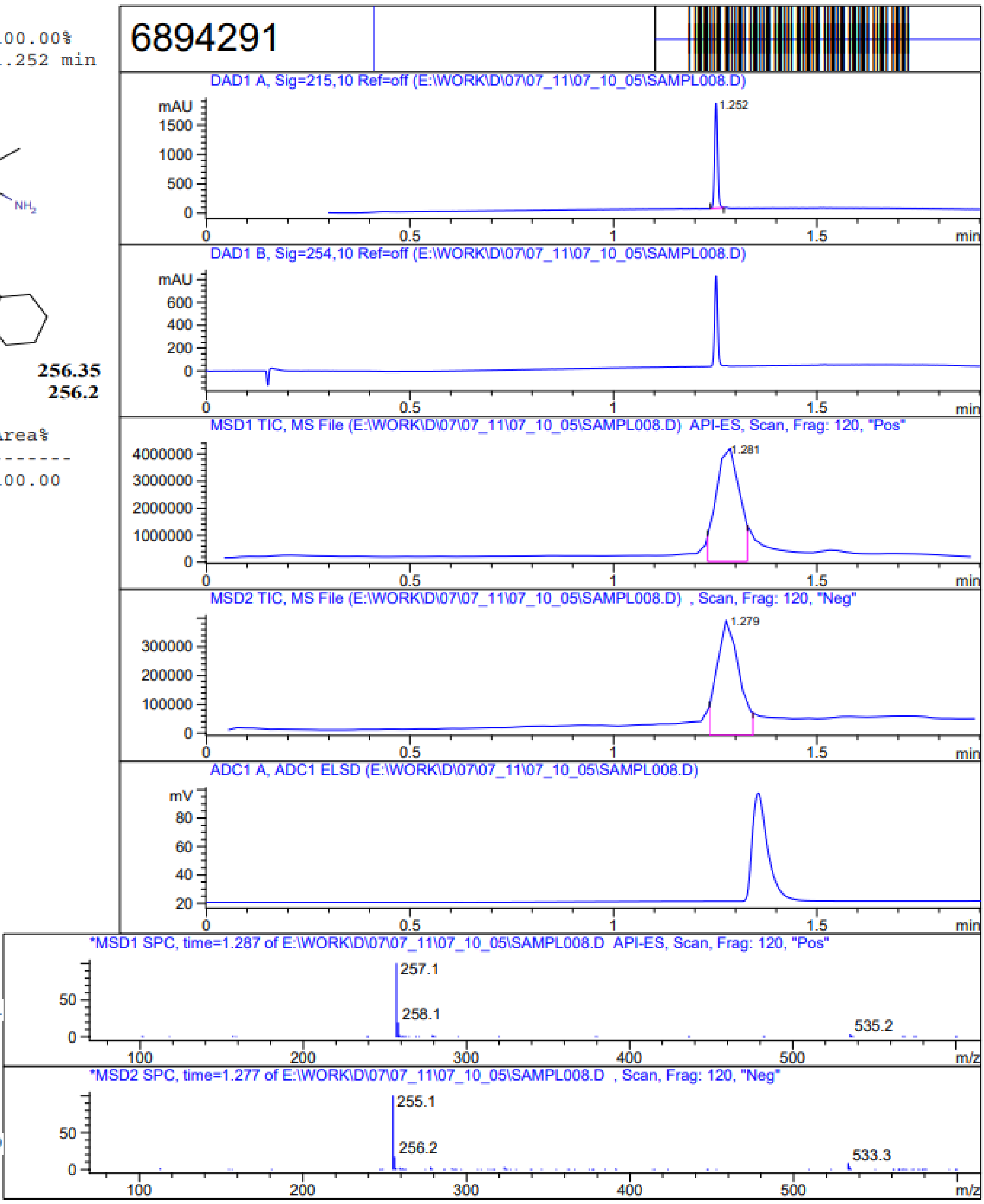

¹H NMR (500 MHz) spectrum of compound 2.14 (10 mM in DMSO-d6 at 298K).

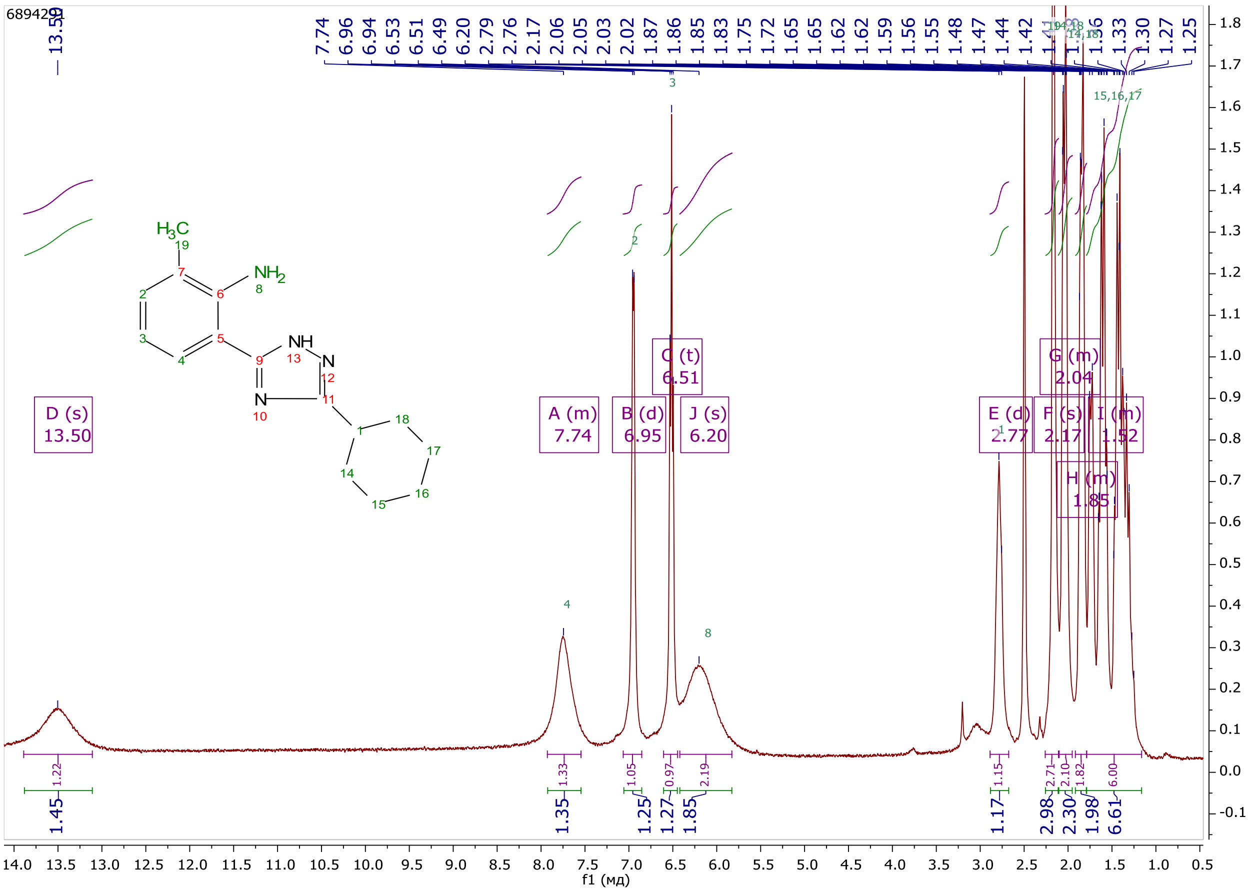

HPLC MS (methanol-water, APCI) data of compound 2.15

MaxPeak: 100.00%
Ret_Time: 1.261 min

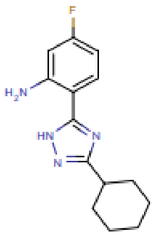

**Mol Wt**	**260.31**
**Exact Mass**	**260.17**

#	Time	Area%
1	1.261	100.00

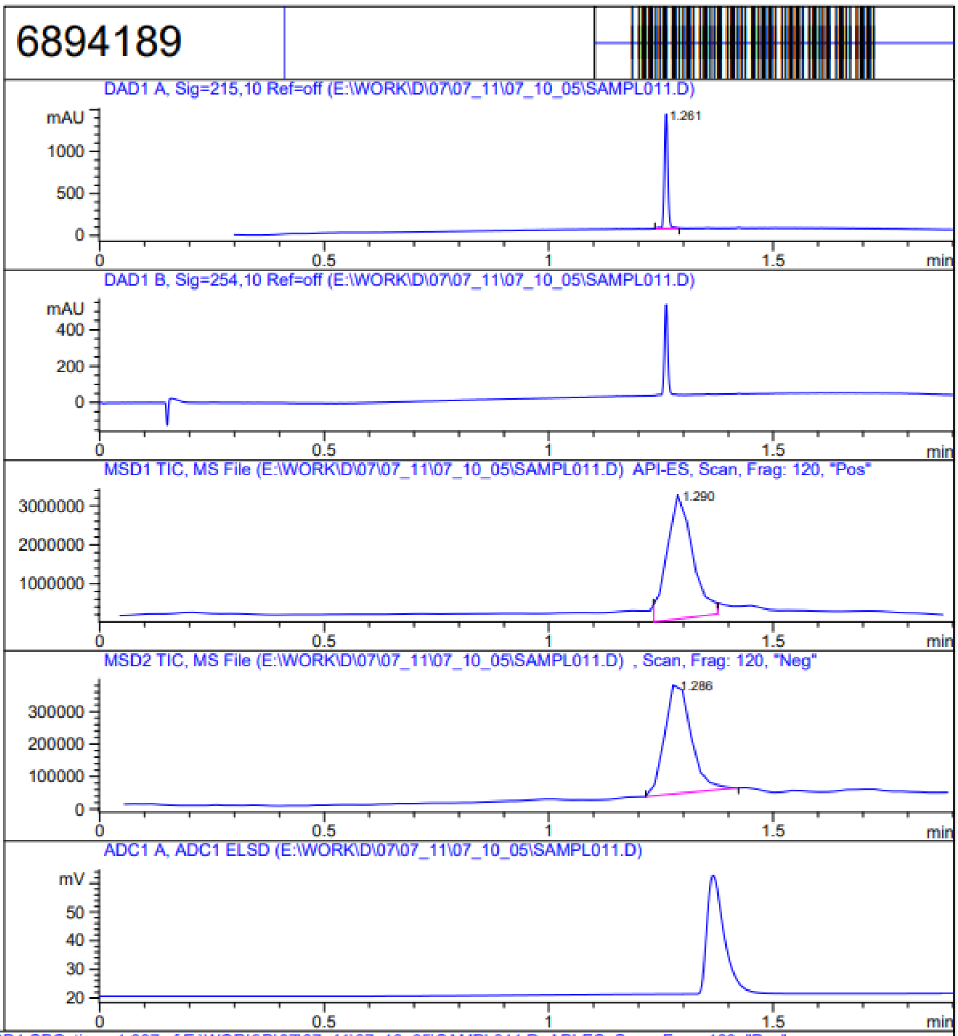

RT 1.290

RT 1.286

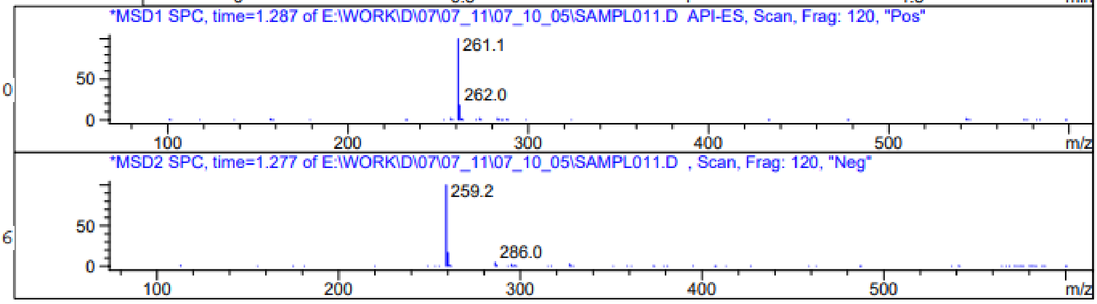

^{1}H NMR (500 MHz) spectrum of compound 2.15 (10 mM in DMSO-d6 at 298K).

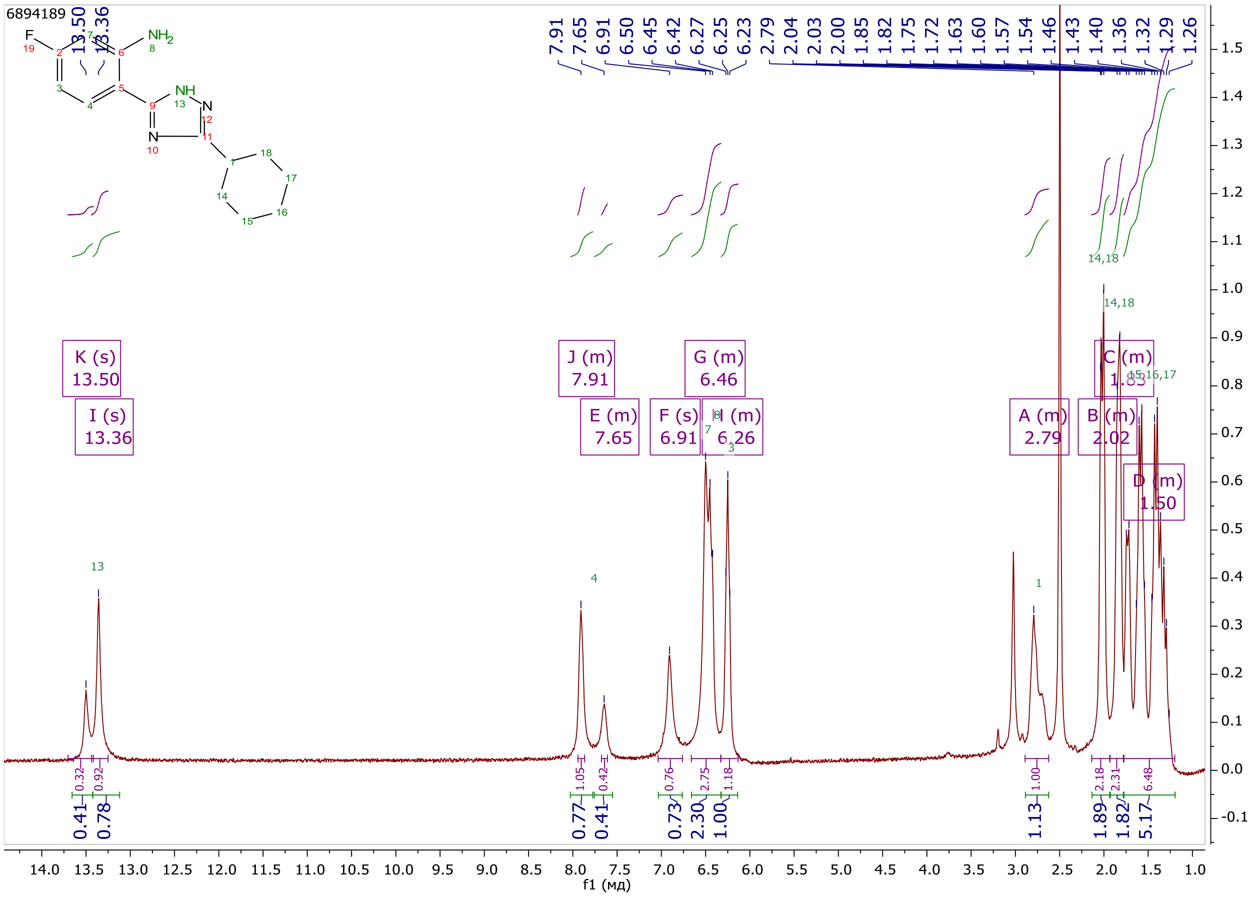

HPLC MS (methanol-water, APCI) data of compound 2.16

```
MaxPeak:   96.94%
Ret_Time:  1.356 min
```

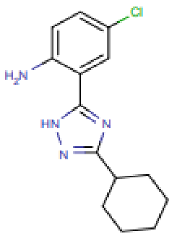

Mol Wt 276.76
Exact Mass 276.14

#	Time	Area%
1	1.356	96.94
2	1.391	3.06

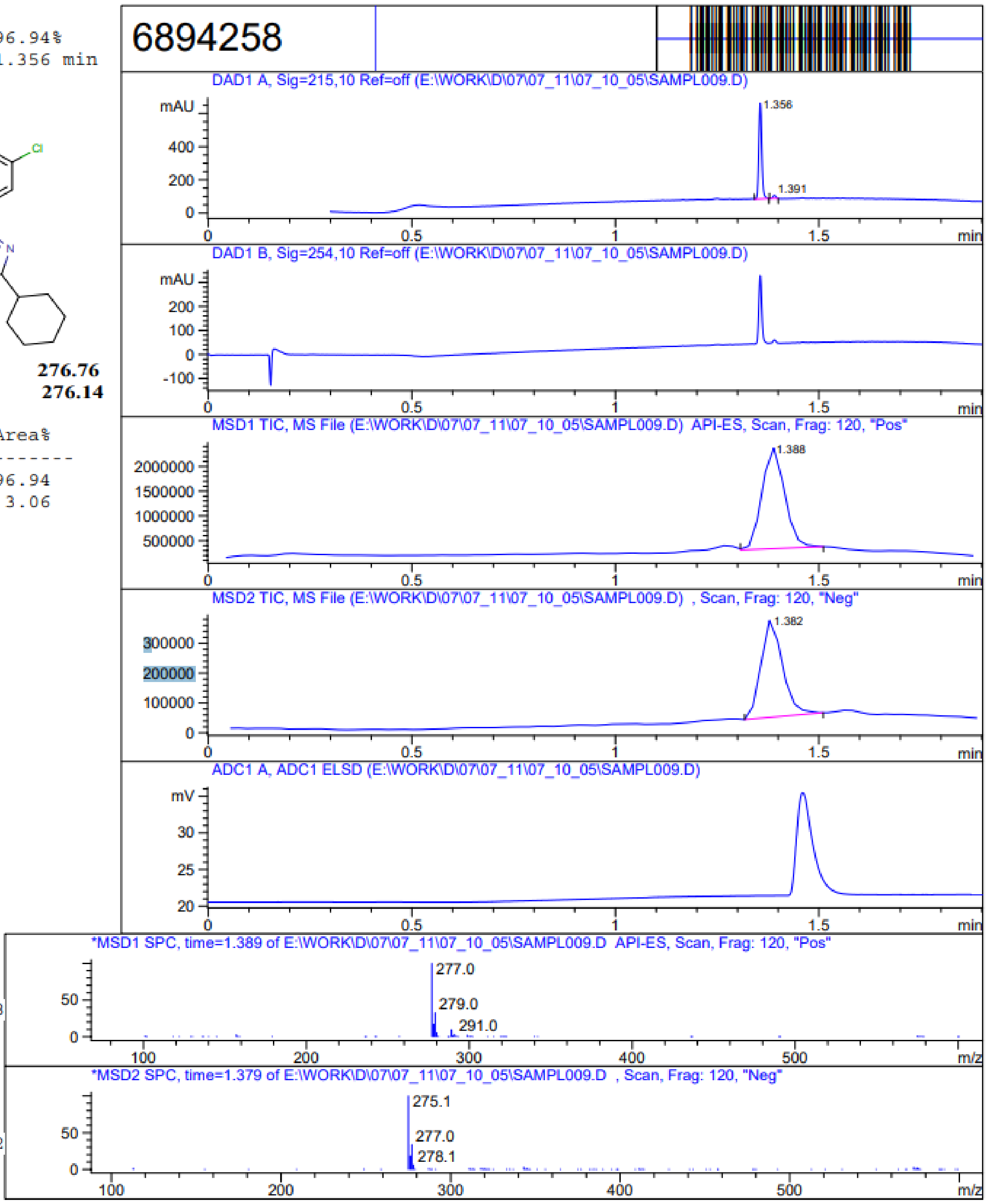

RT 1.388

RT 1.382

^{1}H NMR (500 MHz) spectrum of compound 2.16 (10 mM in DMSO-d6 at 298K).

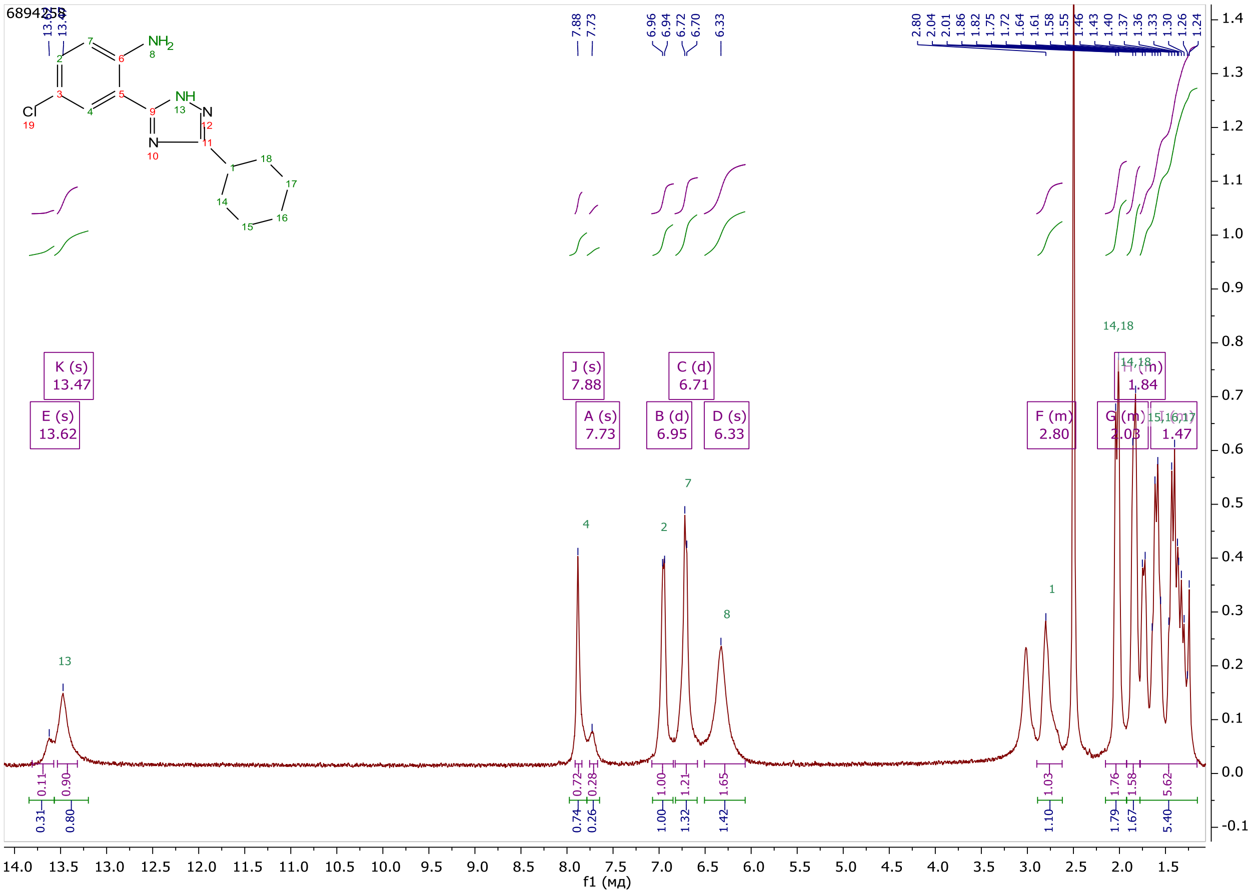

HPLC MS (methanol-water, APCI) data of compound 2.17

MaxPeak: 100.00%
Ret_Time: 1.362 min

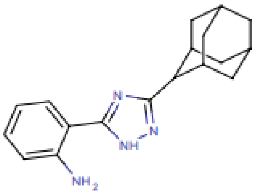

Mol Wt 294.39
Exact Mass 294.22

#	Time	Area%
1	1.362	100.00

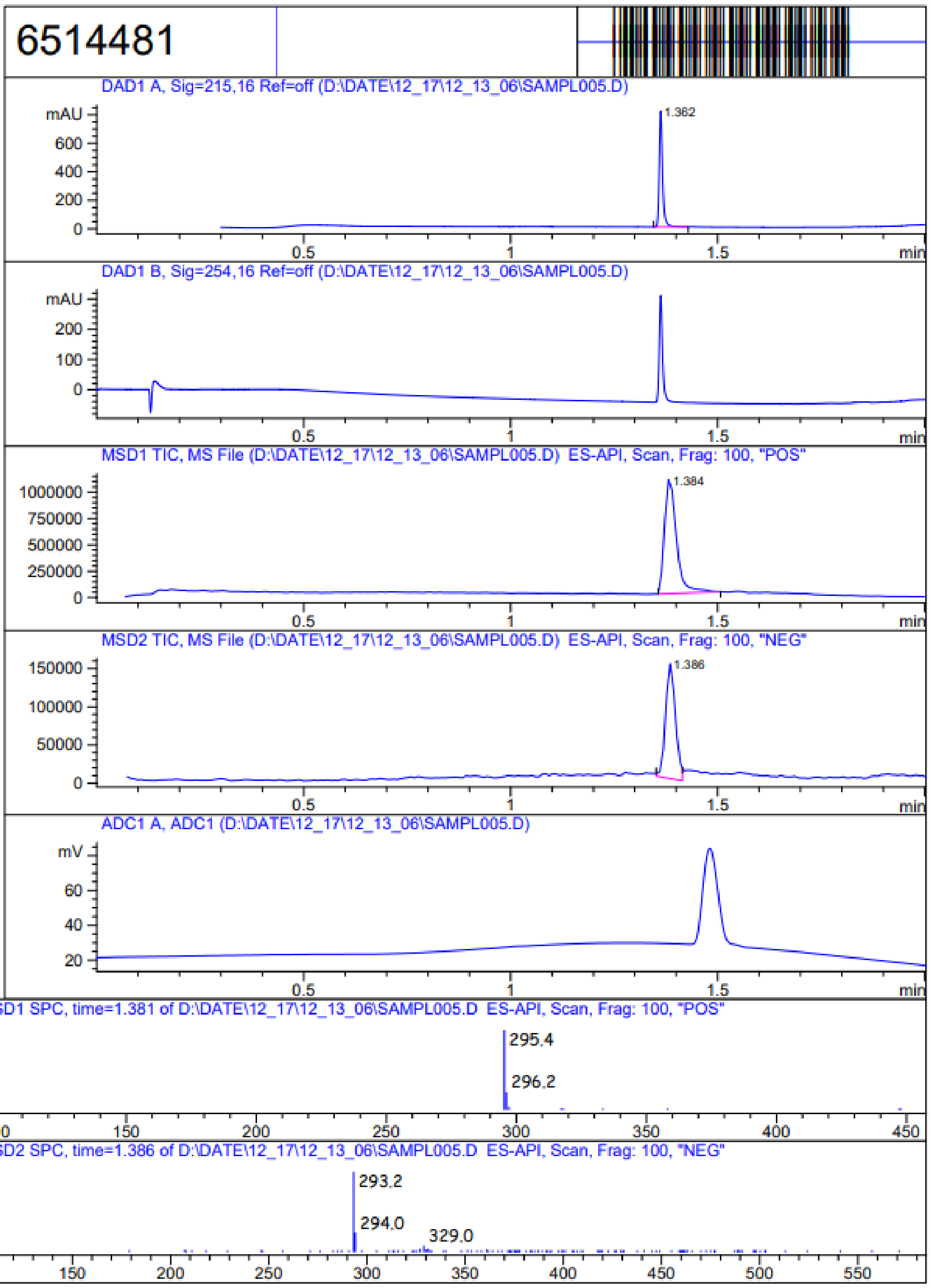

^{1}H NMR (500 MHz) spectrum of compound 2.17 (10 mM in DMSO-d6 at 298K)

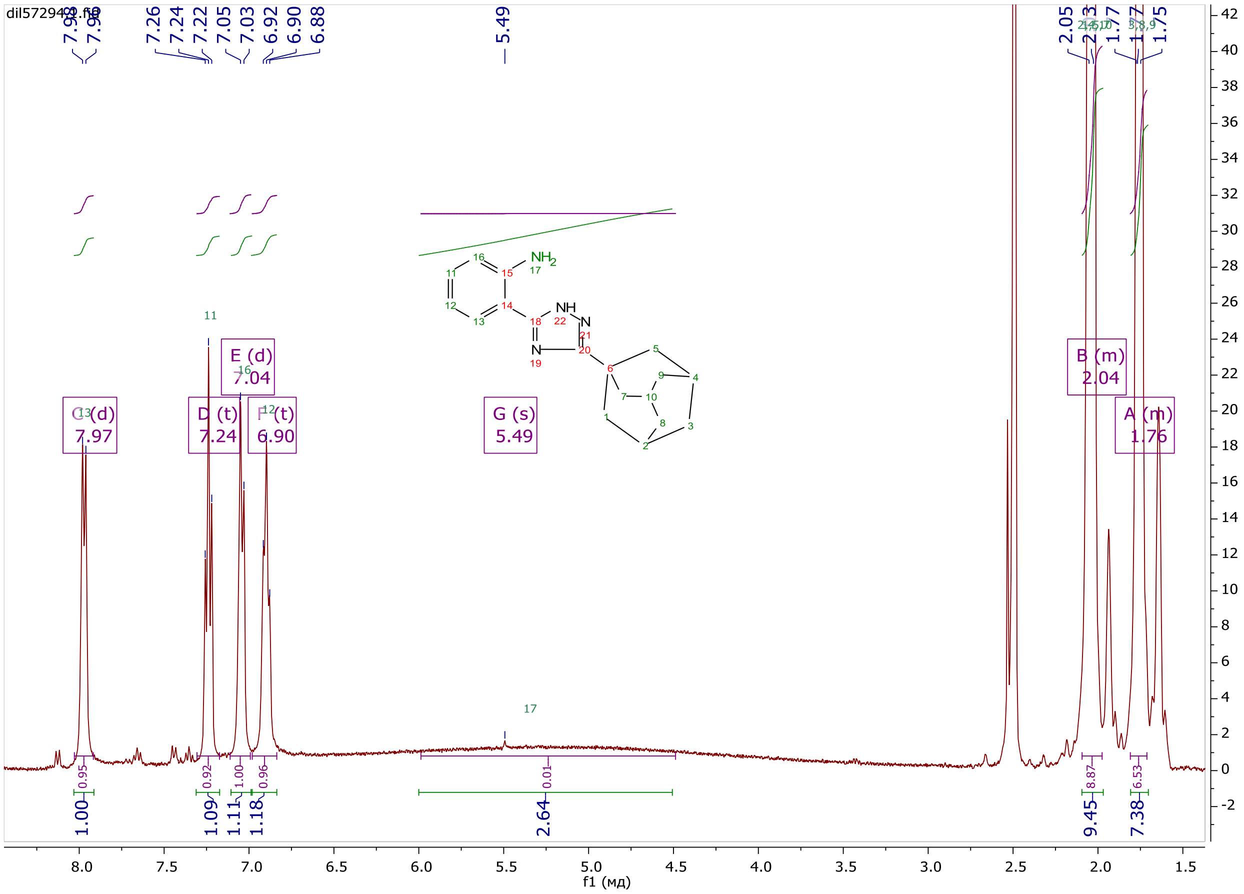

^{13}C NMR (125 MHz) spectrum of compound 2.17 (DMSO-d6 at 298K).

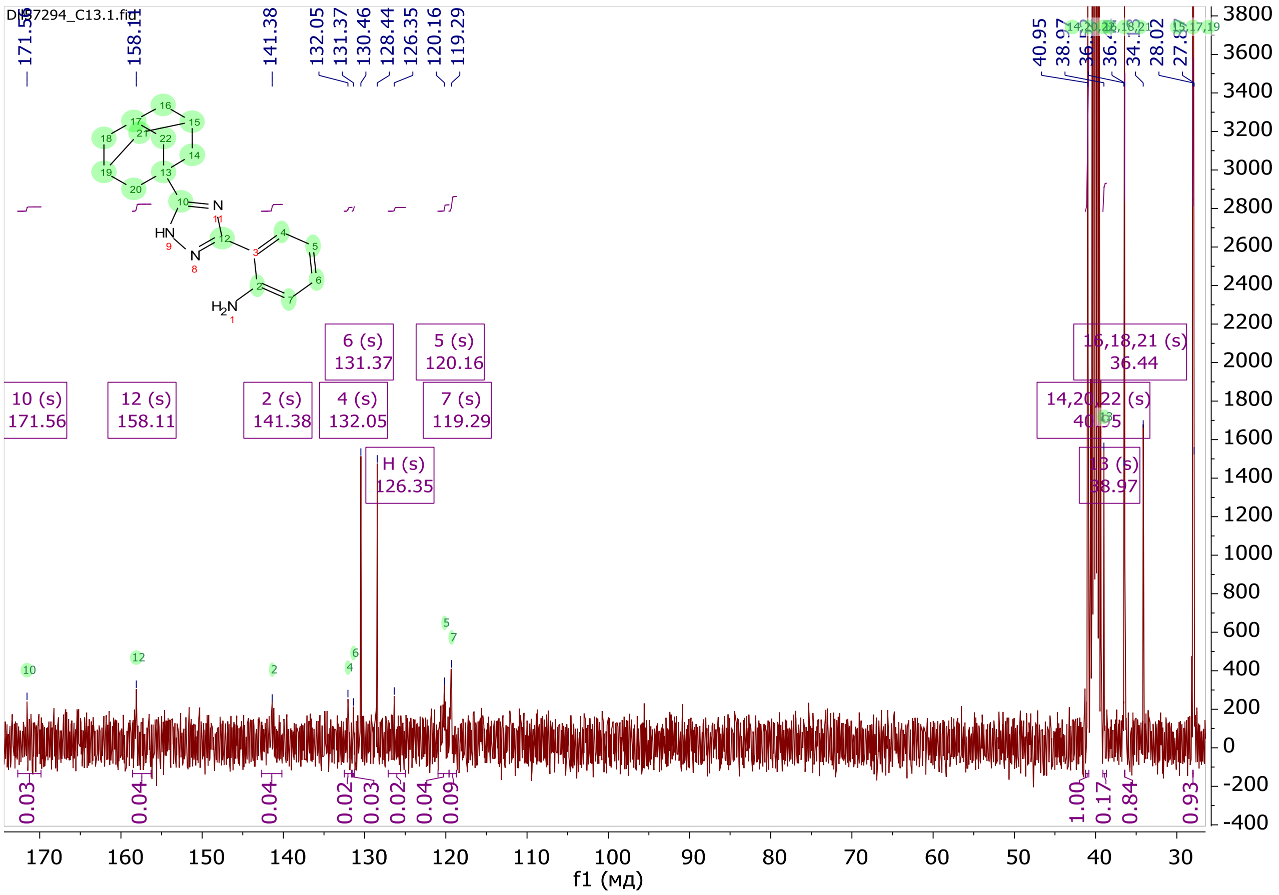

HPLC MS (methanol-water, APCI) data of compound 2.18

MaxPeak: 100.00%
Ret_Time: 1.418 min

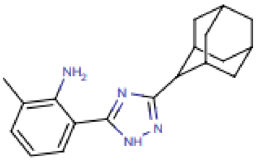

Mol Wt 308.42
Exact Mass 308.24

#	Time	Area%
1	1.418	100.00

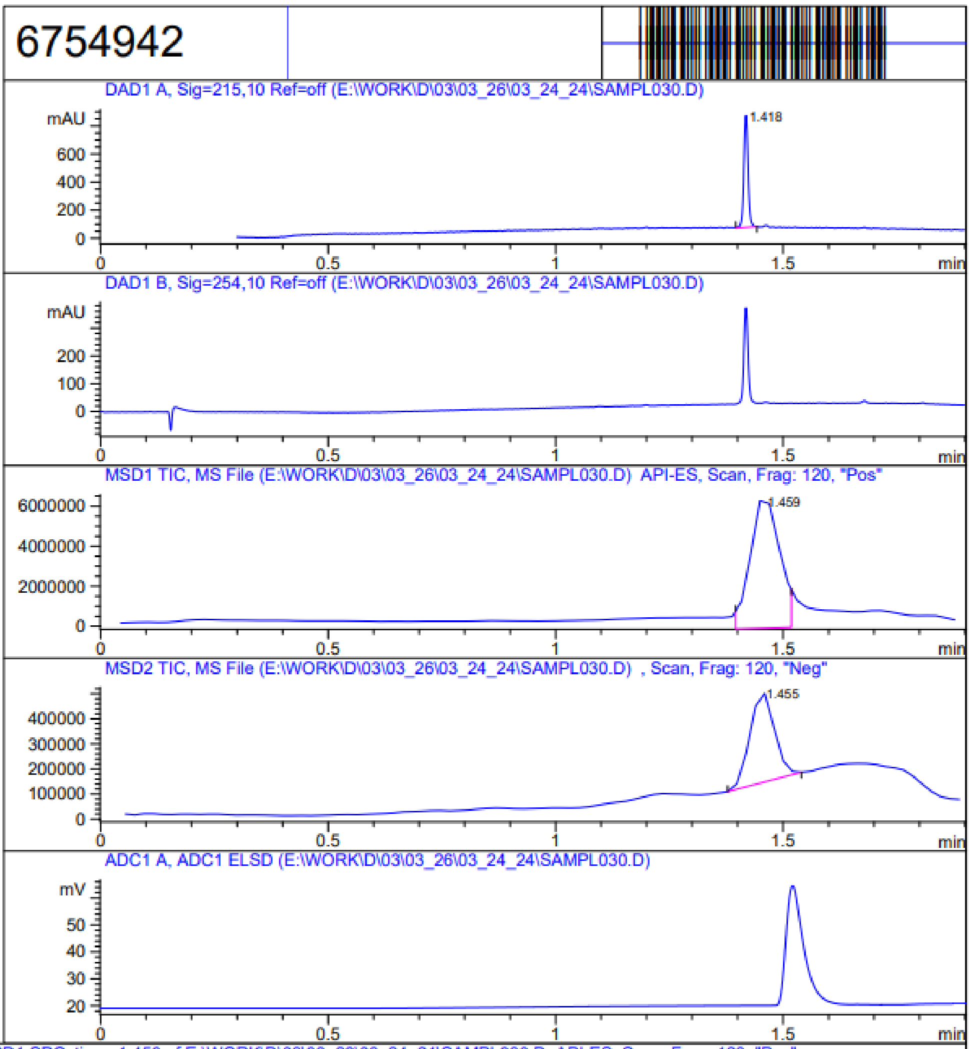

RT 1.459

RT 1.455

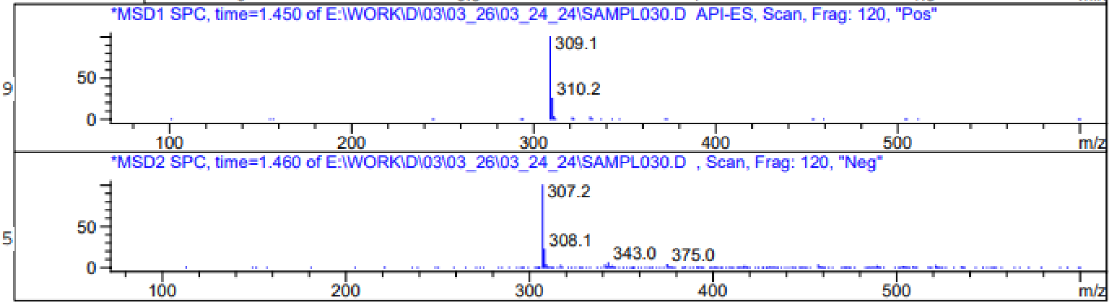

^{1}H NMR (500 MHz) spectrum of compound 2.18 (10 mM in DMSO-d6 at 298K).

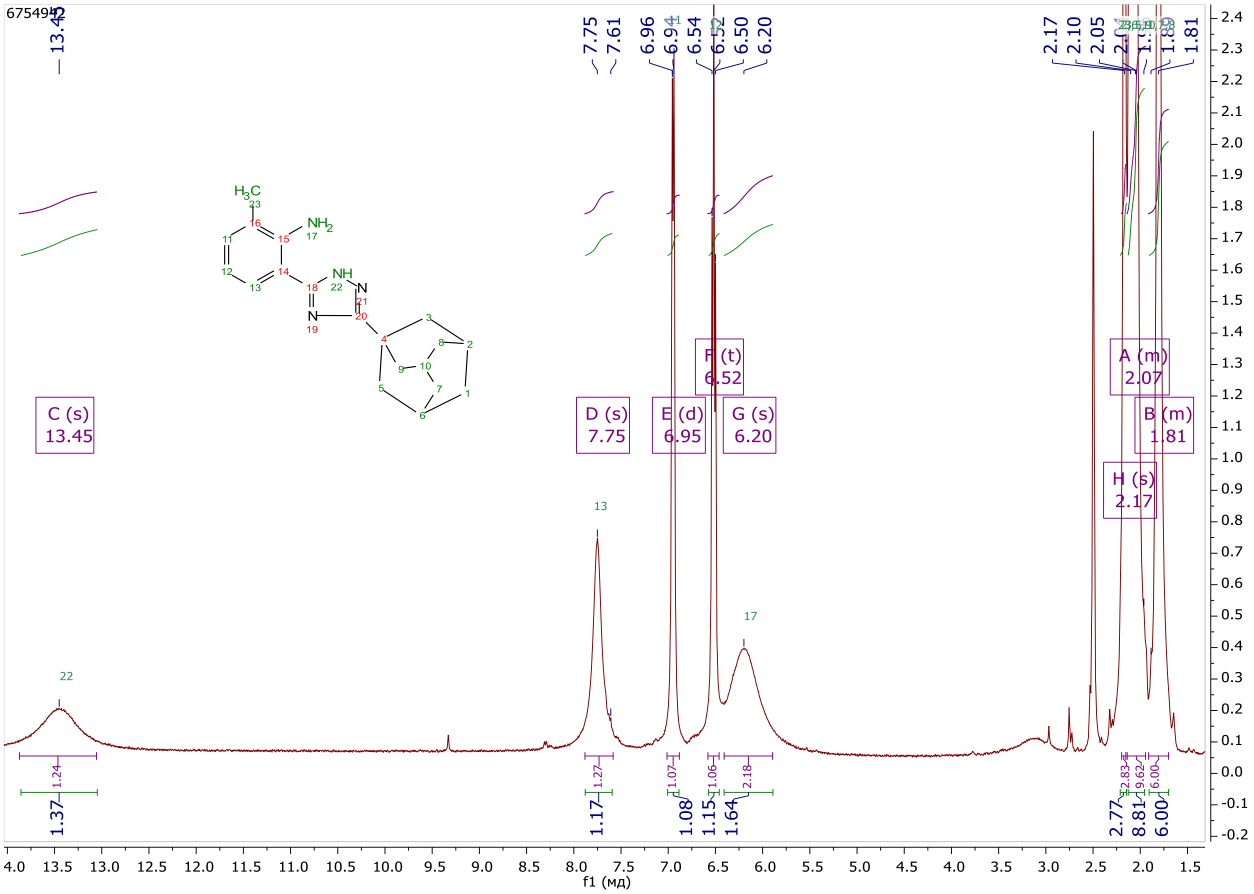

HPLC MS (methanol-water, APCI) data of compound 2.19

MaxPeak: 95.20%
Ret_Time: 1.486 min

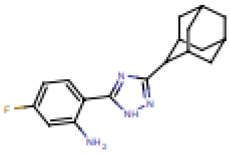

Mol Wt	312.38
Exact Mass	312.21

#	Time	Area%
1	0.922	4.80
2	1.486	95.20

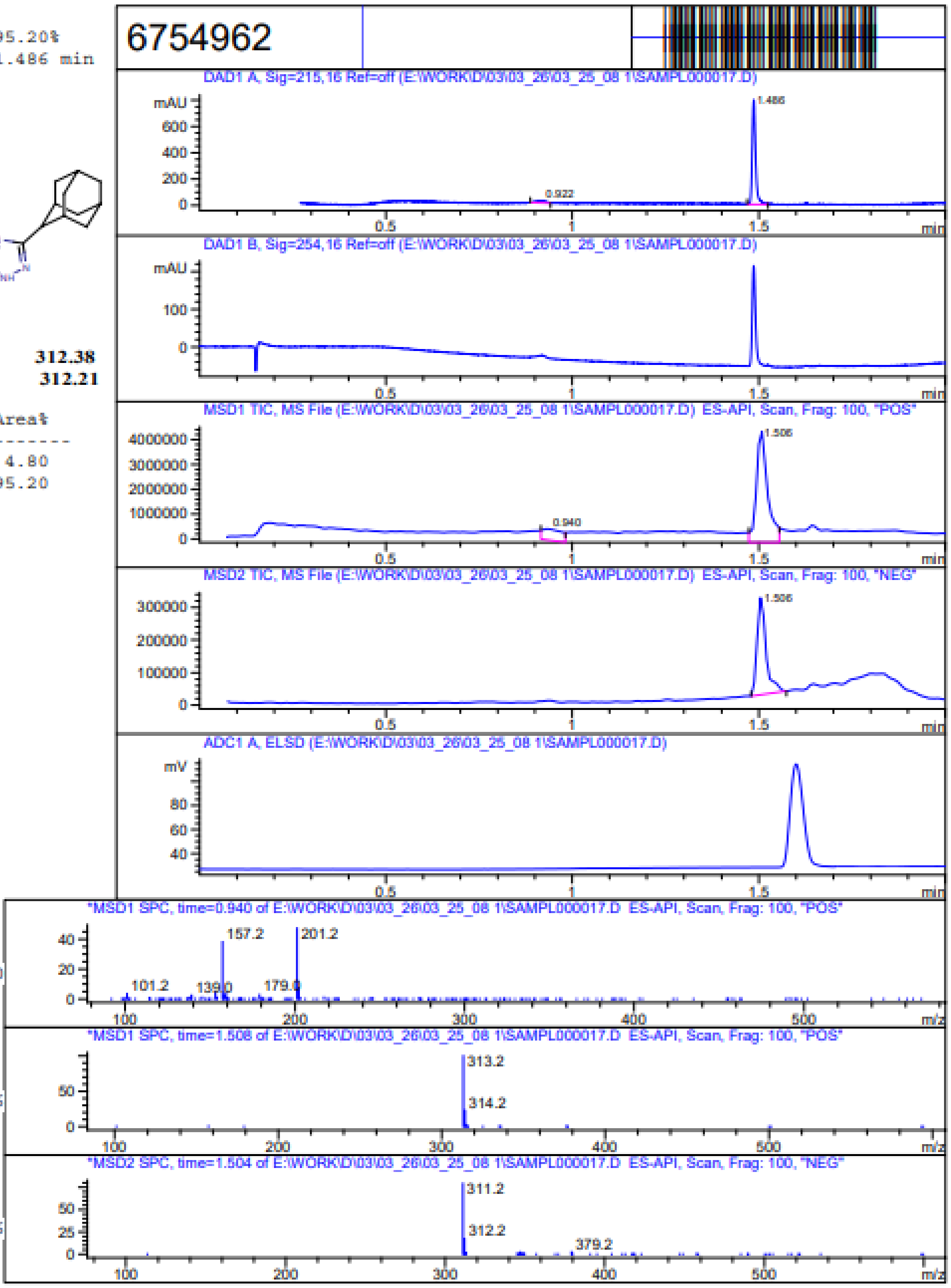

^{1}H NMR (500 MHz) spectrum of compound 2.19 (10 mM in DMSO-d6 at 298K).

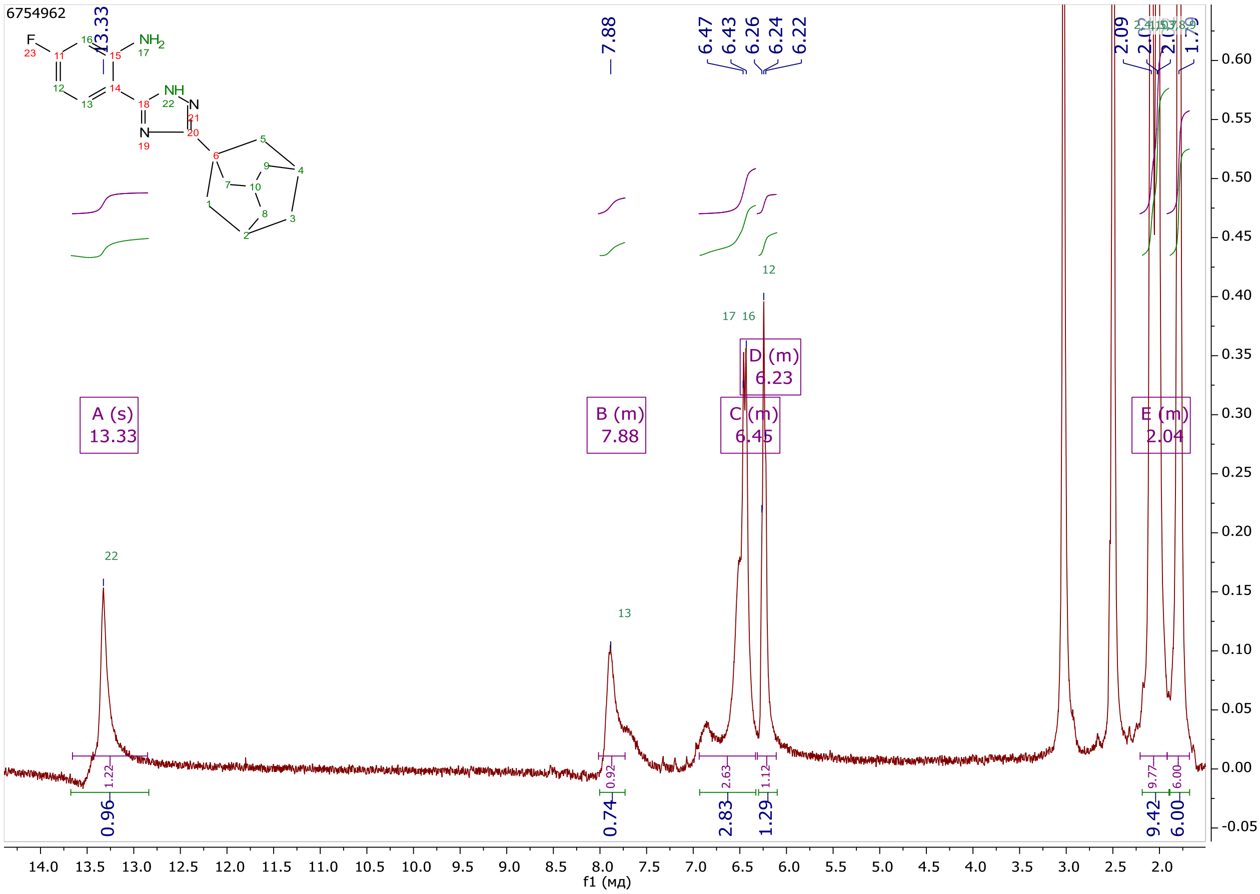

HPLC MS (methanol-water, APCI) data of compound 2.20

```
MaxPeak:  100.00%
Ret_Time: 1.505 min
```

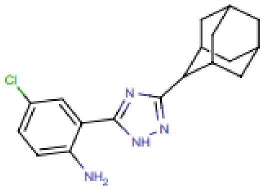

Mol Wt 328.84
Exact Mass 328.18

```
#  Time   Area%
-----------------
1  1.505  100.00
```

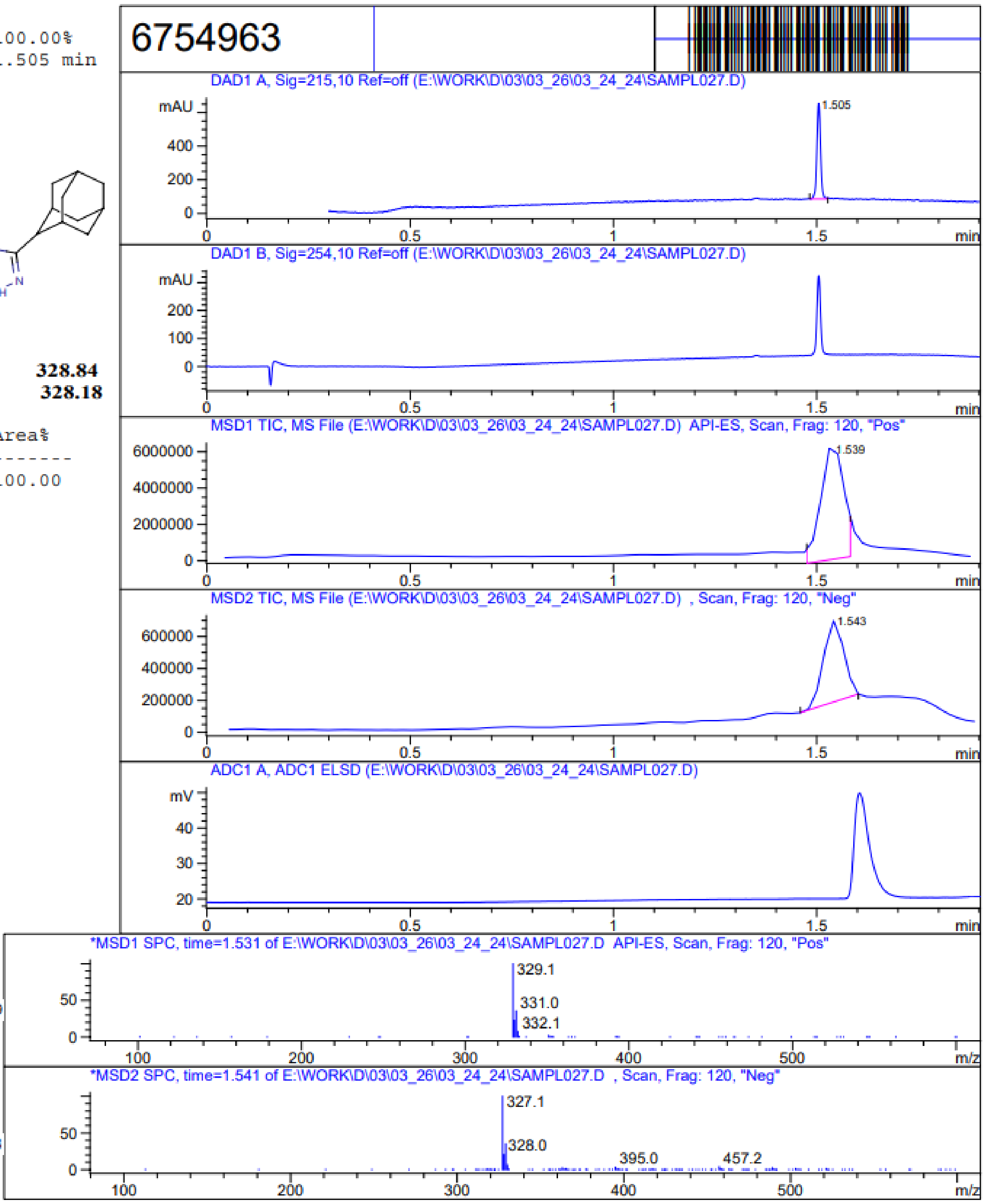

RT 1.539

RT 1.543

^{1}H NMR (500 MHz) spectrum of compound 2.20 (10 mM in DMSO-d6 at 298K).

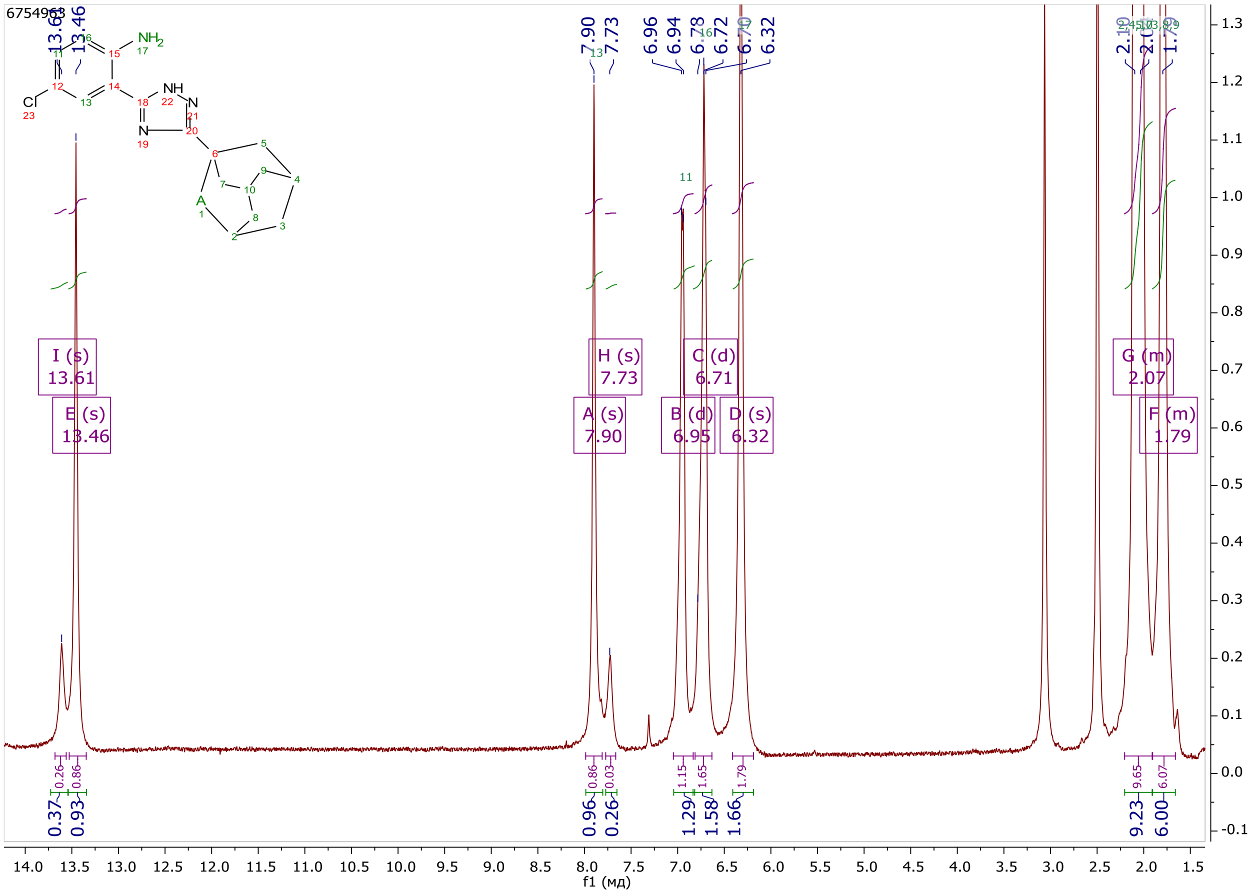

HPLC MS (methanol-water, APCI) data of compound 2.21

MaxPeak: 100.00%
Ret_Time: 1.524 min

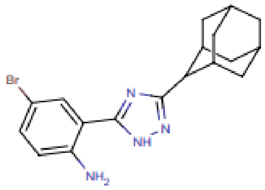

**Mol Wt**	**373.29**
**Exact Mass**	**372.13**

#	Time	Area%
1	1.524	100.00

RT 1.558

RT 1.563

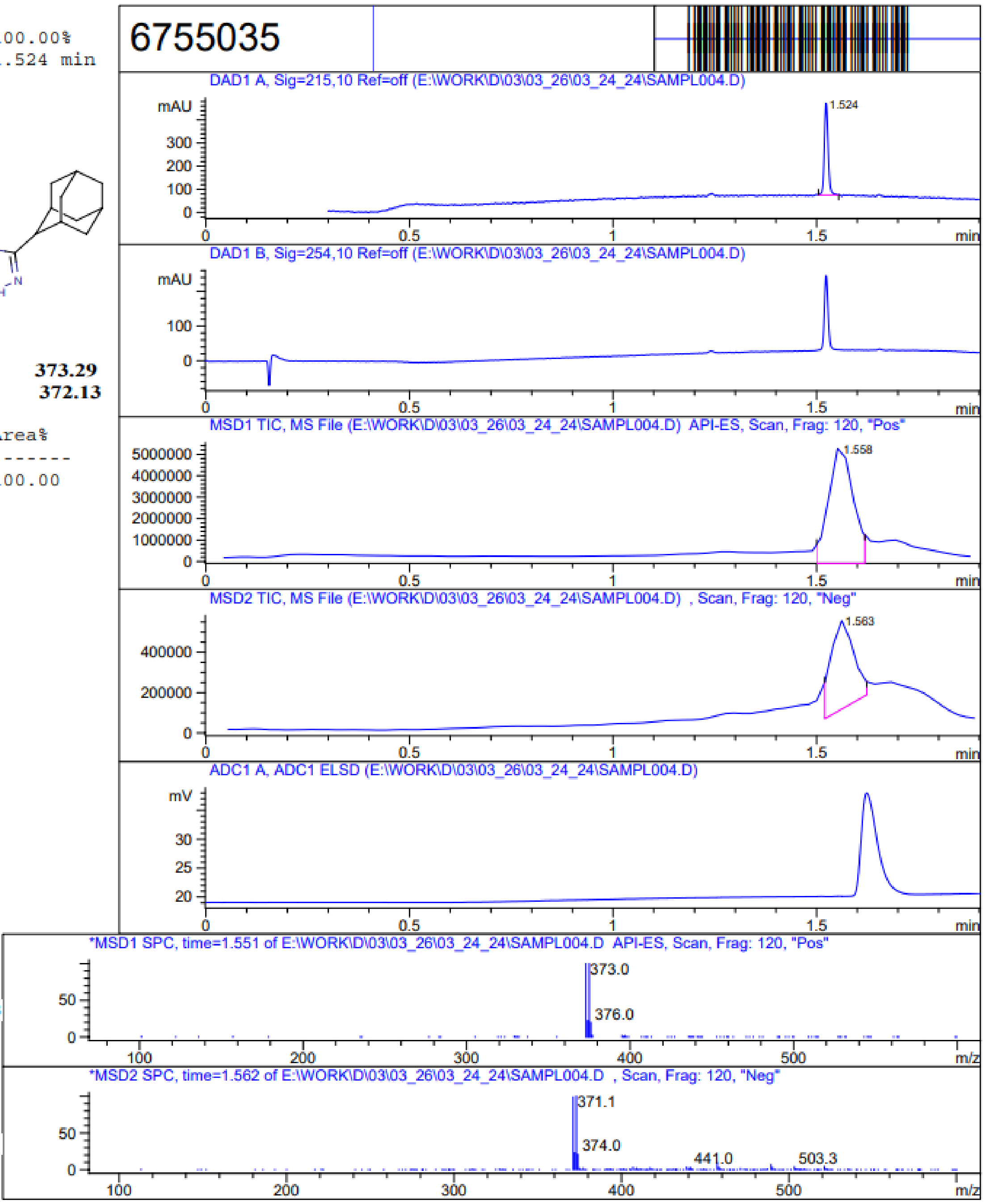

^{1}H NMR (500 MHz) spectrum of compound 2.21 (10 mM in DMSO-d6 at 298K).

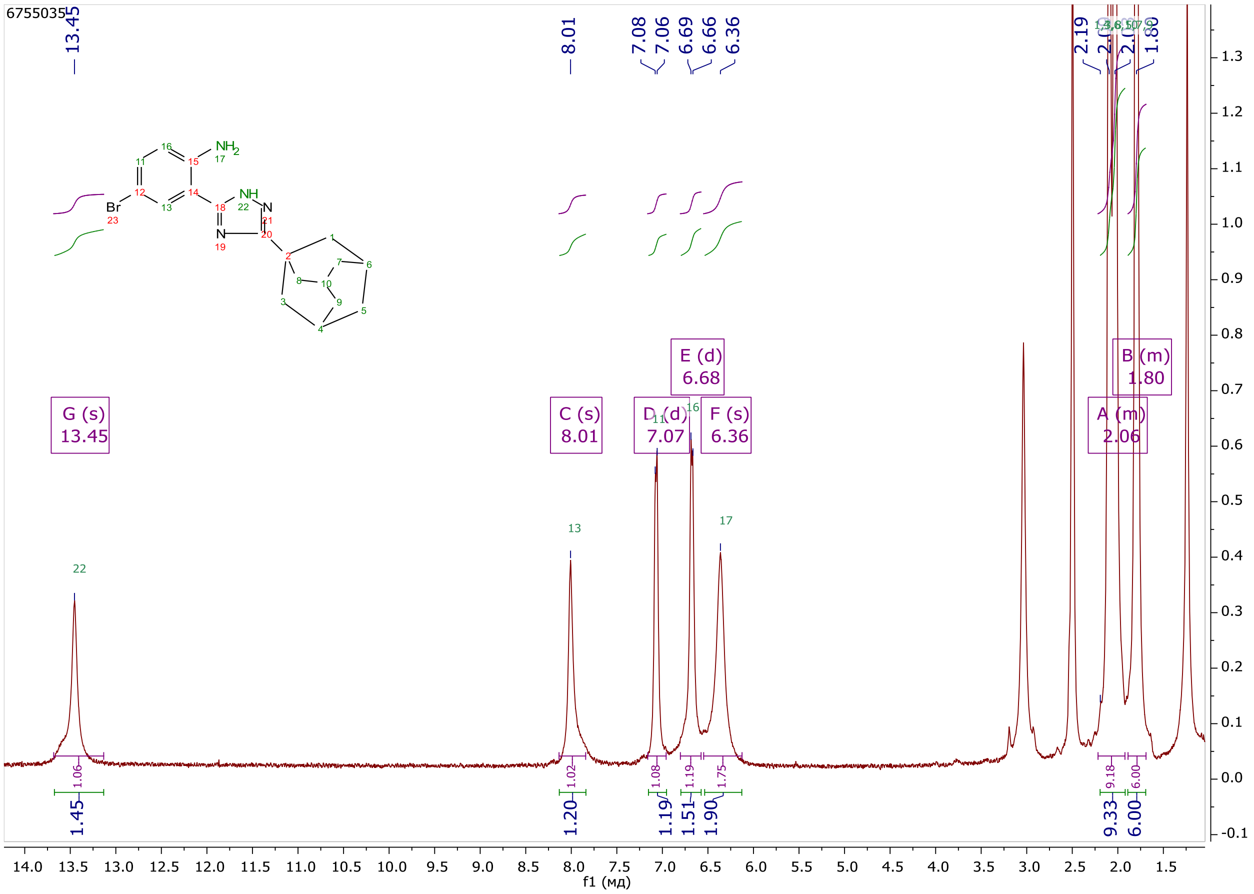

^{1}H NMR (500 MHz) spectrum of compound 2.22 (10 mM in DMSO-d6 at 298K).

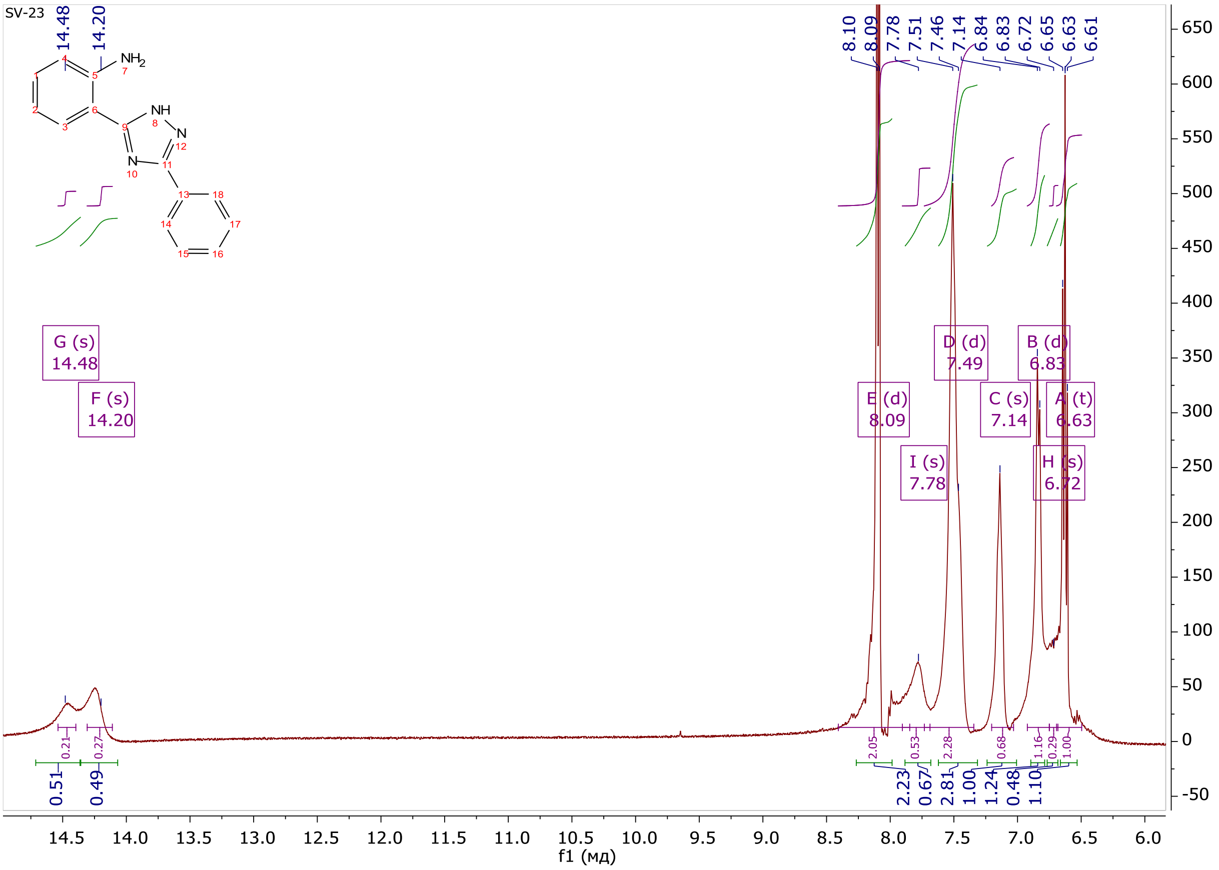

^{1}H NMR (500 MHz) spectrum of compound 2.23 (10 mM in DMSO-d6 at 298K).

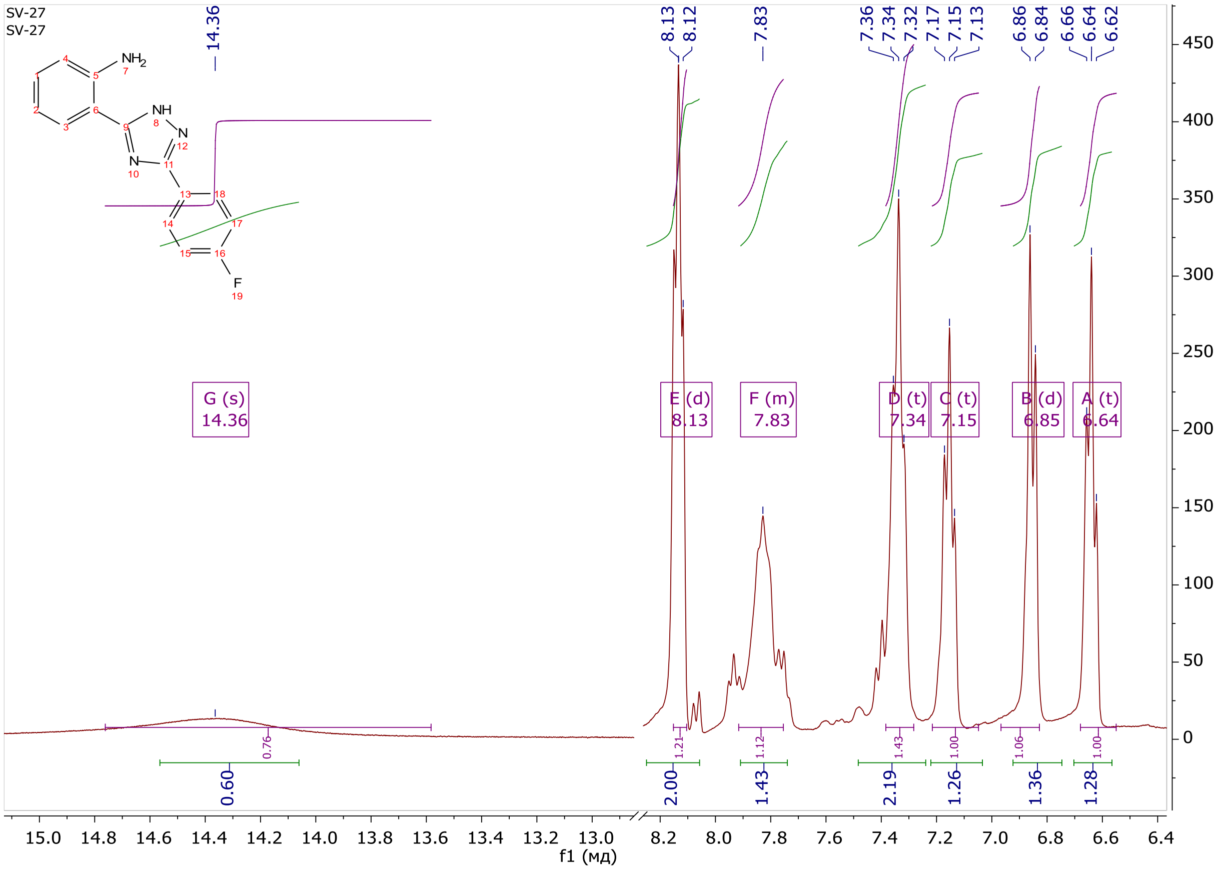

^{1}H NMR (500 MHz) spectrum of compound 2.24 (10 mM in DMSO-d6 at 298K).

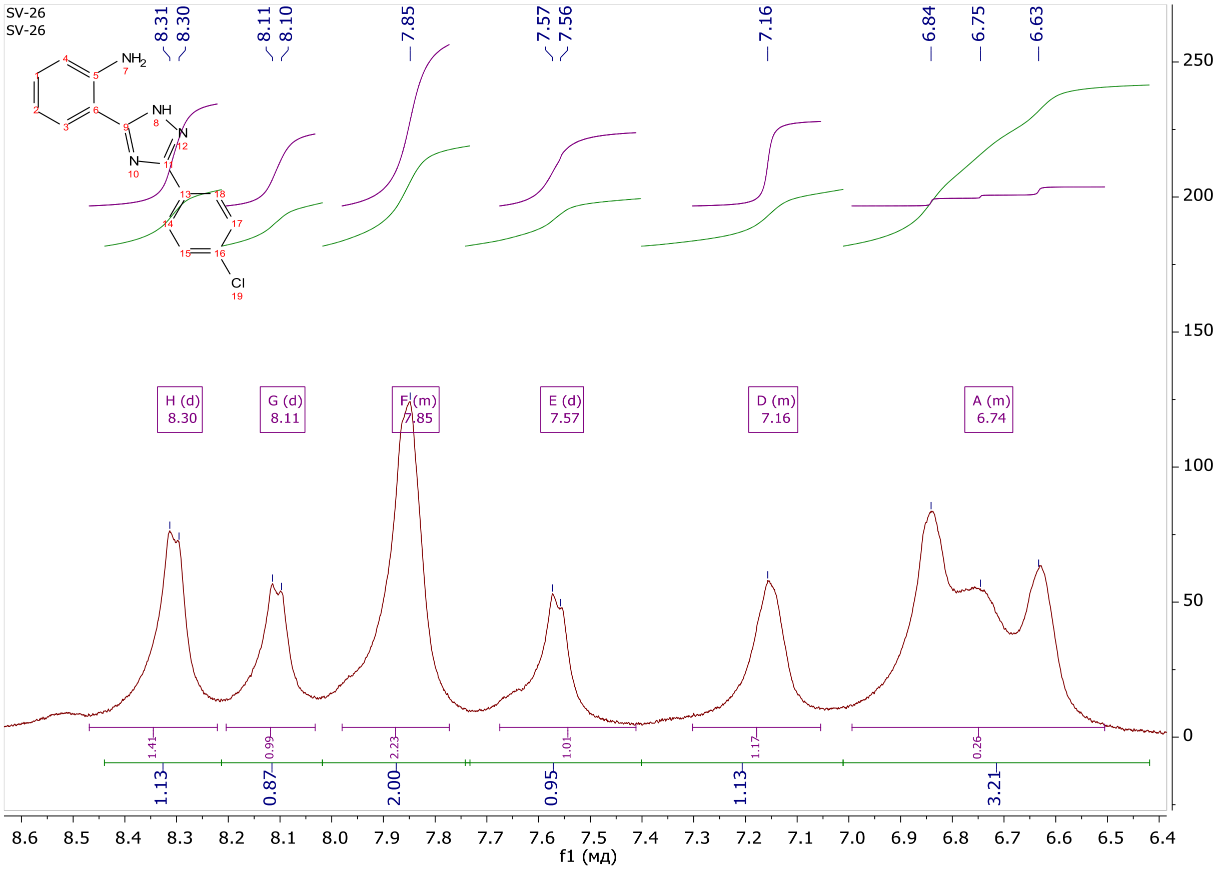

^{1}H NMR (500 MHz) spectrum of compound 2.25 (10 mM in DMSO-d6 at 298K).

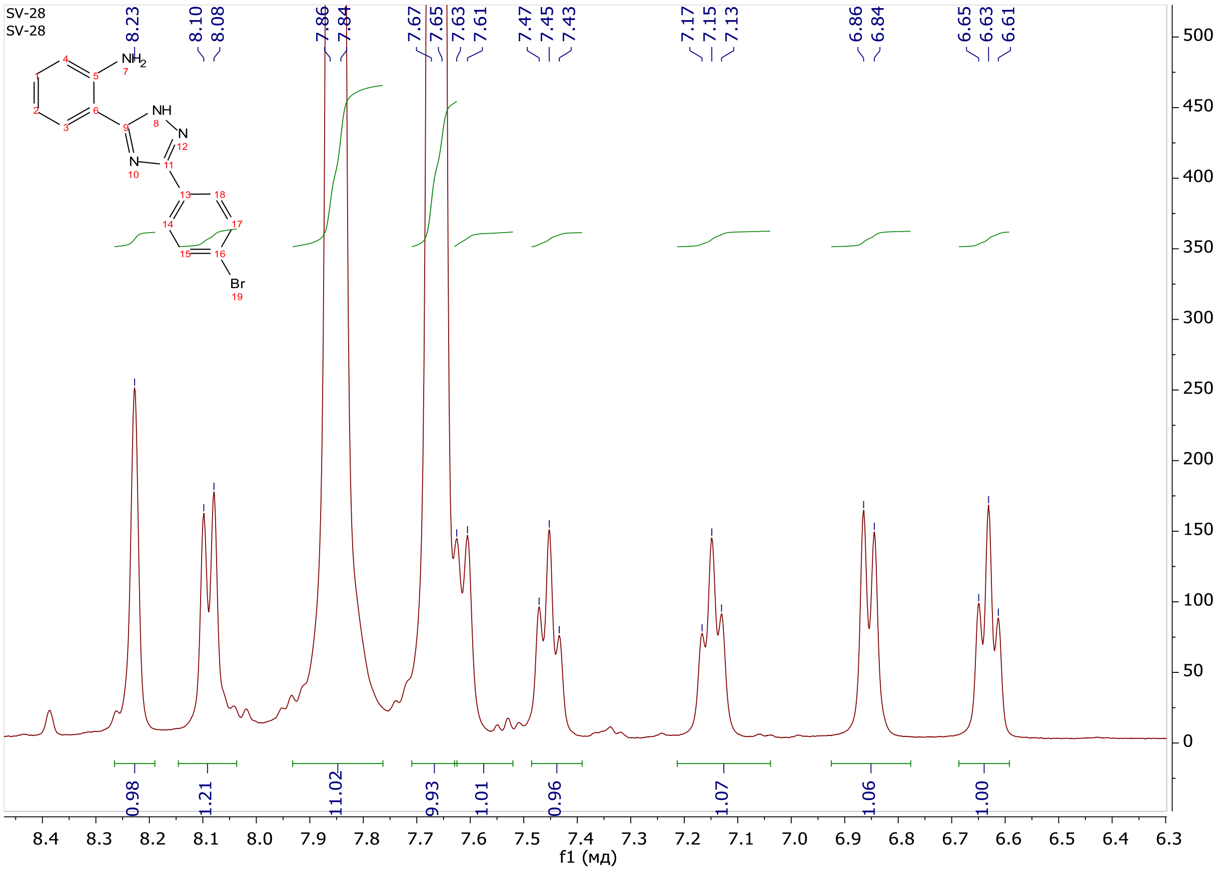

^{1}H NMR (500 MHz) spectrum of compound 2.26 (10 mM in DMSO-d6 at 298K).

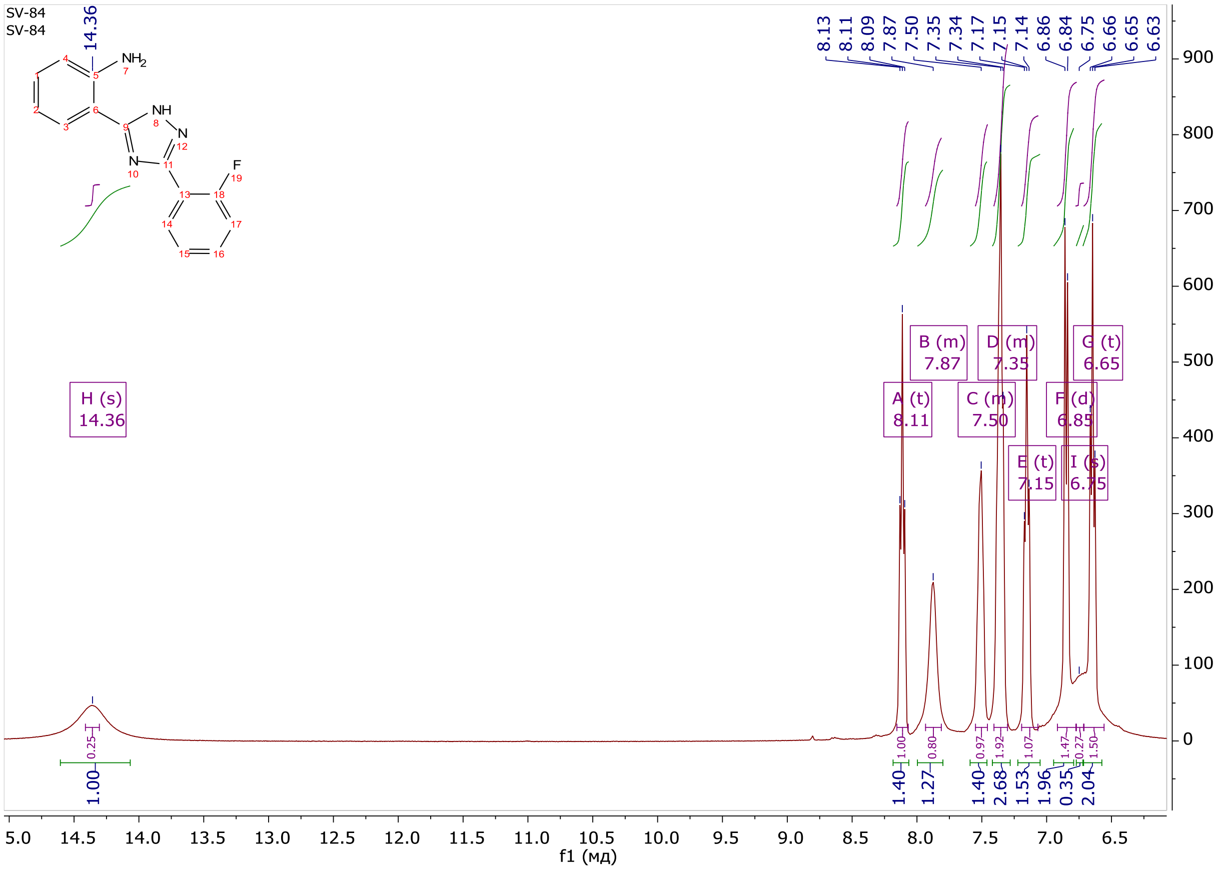

^{13}C NMR (125 MHz) spectrum of compound 2.26 (DMSO-d6 at 298K).

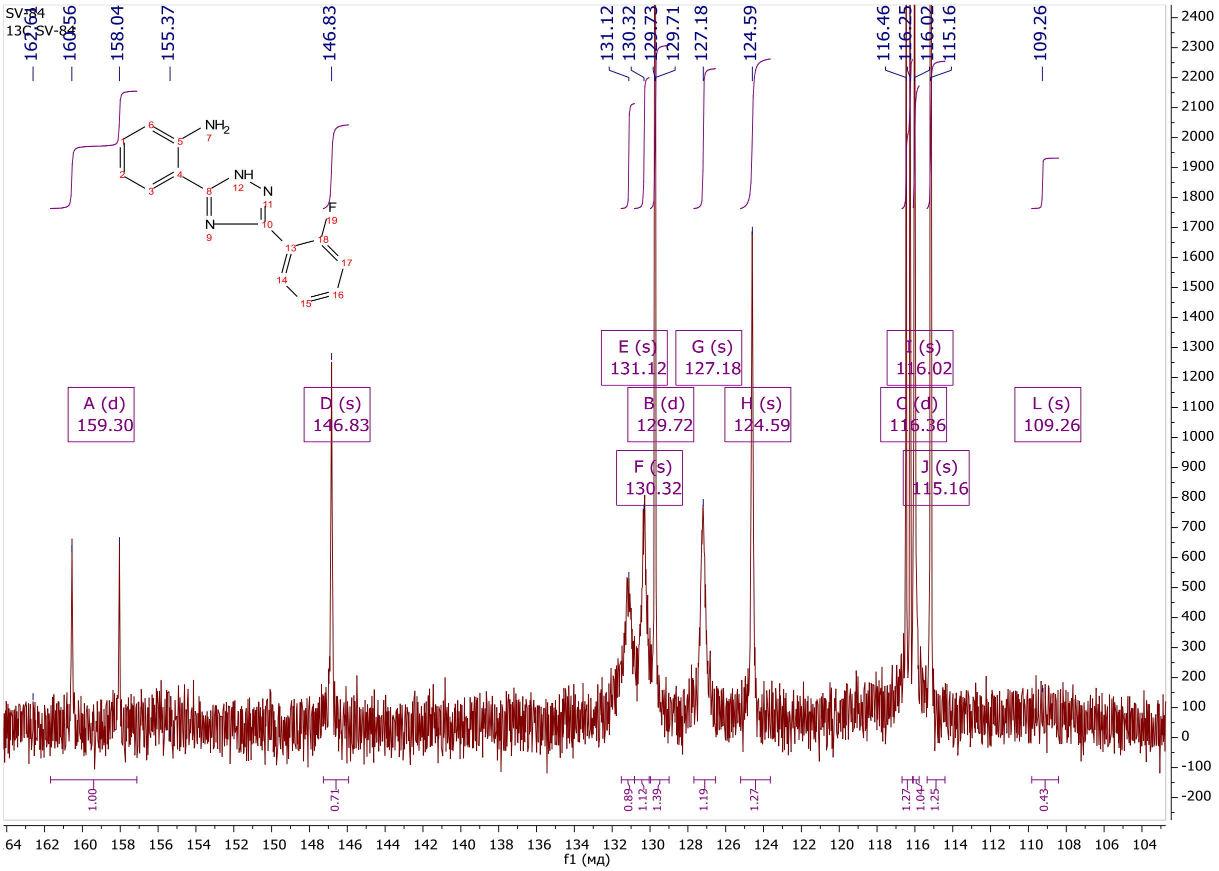

^{1}H NMR (500 MHz) spectrum of compound 2.27 (10 mM in DMSO-d6 at 298K).

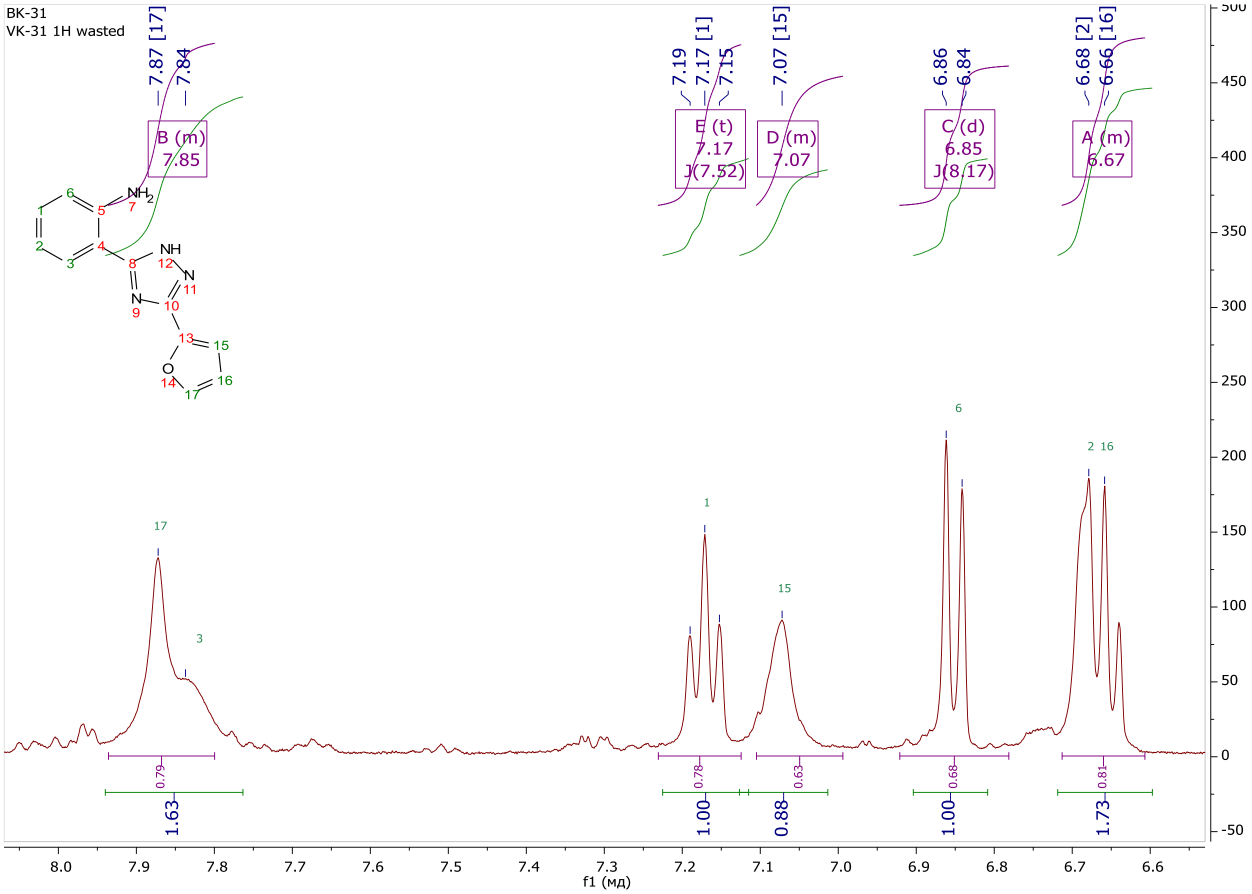

HPLC MS (methanol-water, APCI) data of compound 2.28

MaxPeak: 100.00%
Ret_Time: 1.101 min

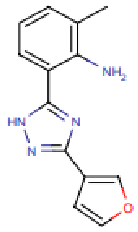

Mol Wt 240.26
Exact Mass 240.11

#	Time	Area%
1	1.101	100.00

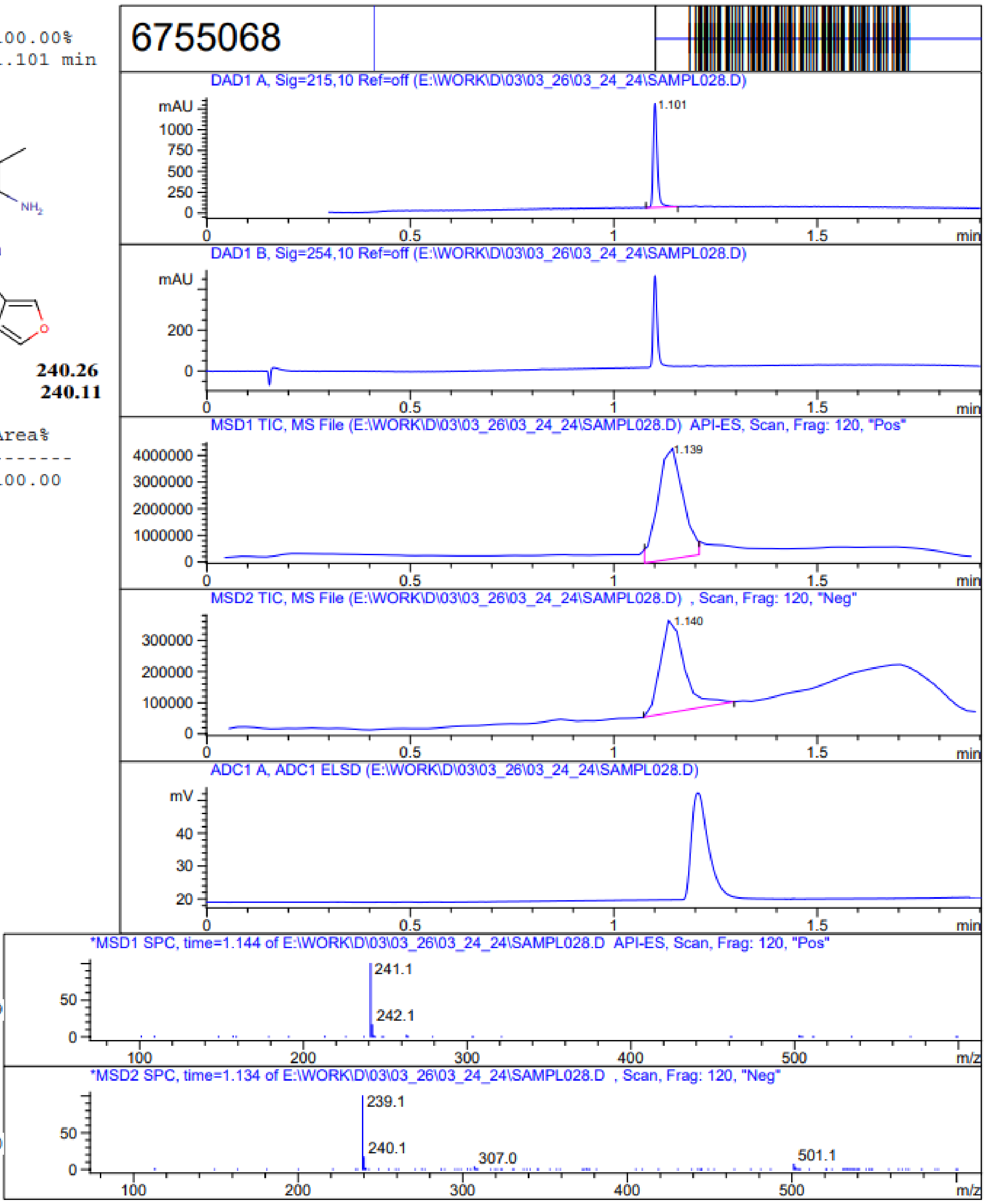

RT 1.139

RT 1.140

^{1}H NMR (500 MHz) spectrum of compound 2.28 (10 mM in DMSO-d6 at 298K).

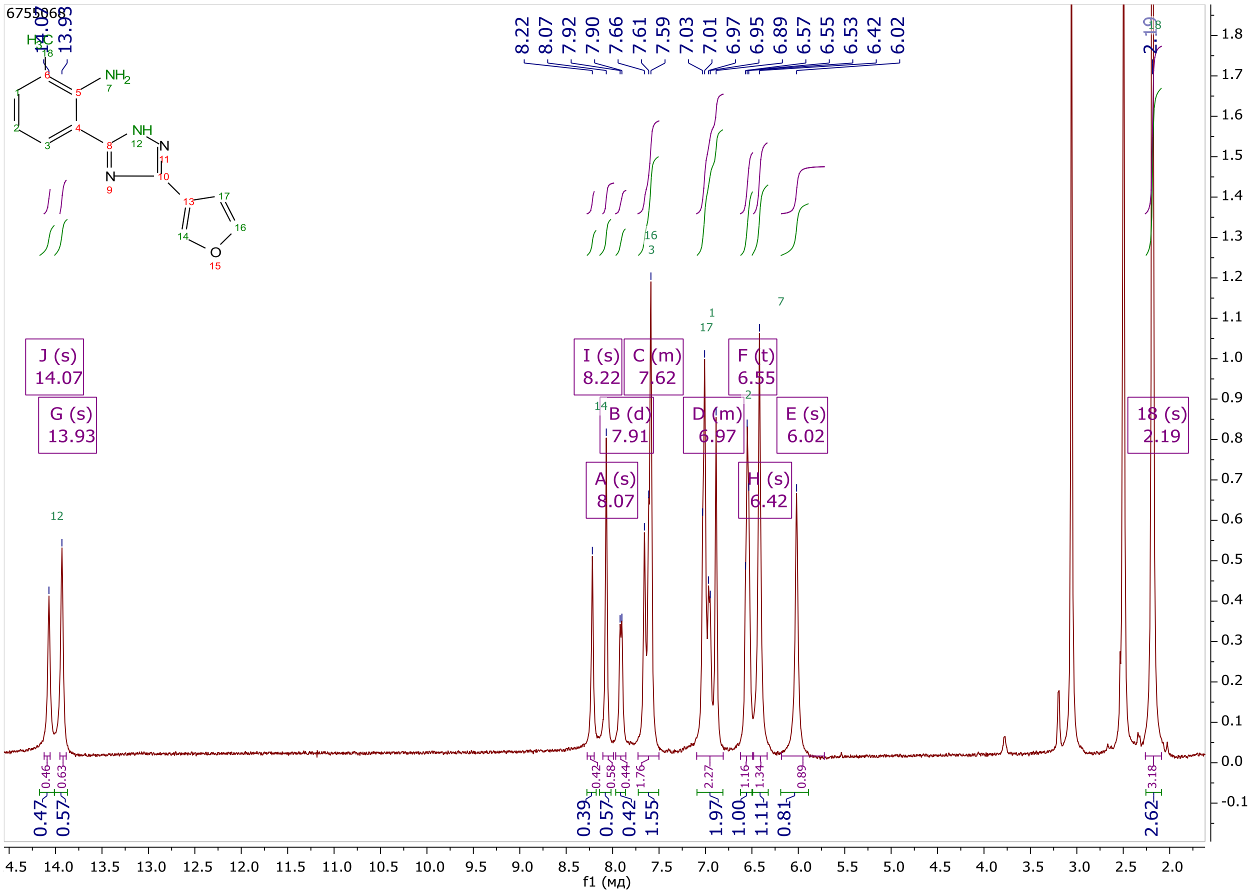

HPLC MS (methanol-water, APCI) data of compound 2.29

MaxPeak: 100.00%
Ret_Time: 1.094 min

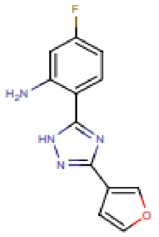

Mol Wt 244.22
Exact Mass 244.08

#	Time	Area%
1	1.094	100.00

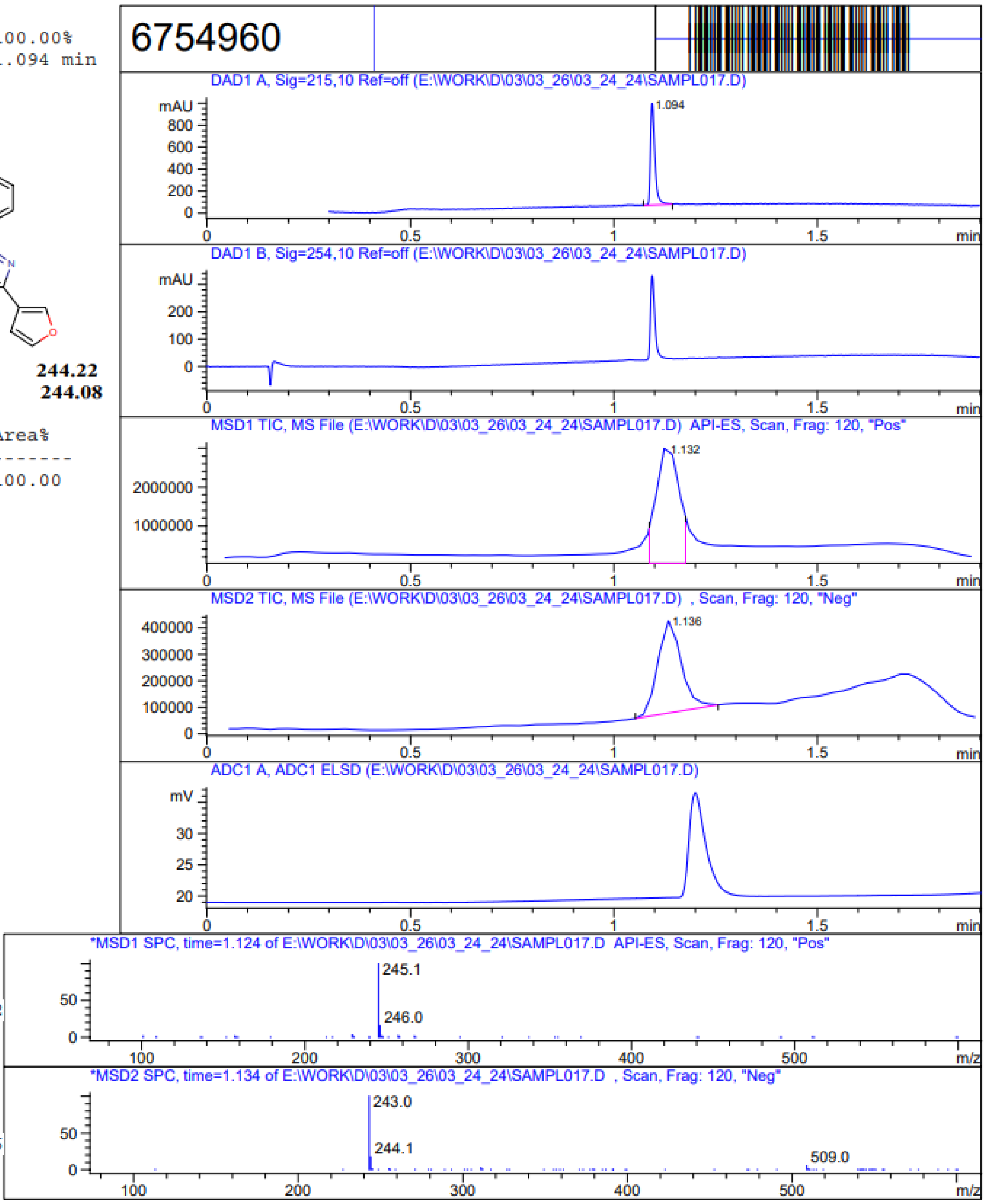

RT 1.132

RT 1.136

^{1}H NMR (500 MHz) spectrum of compound 2.29 (10 mM in DMSO-d6 at 298K).

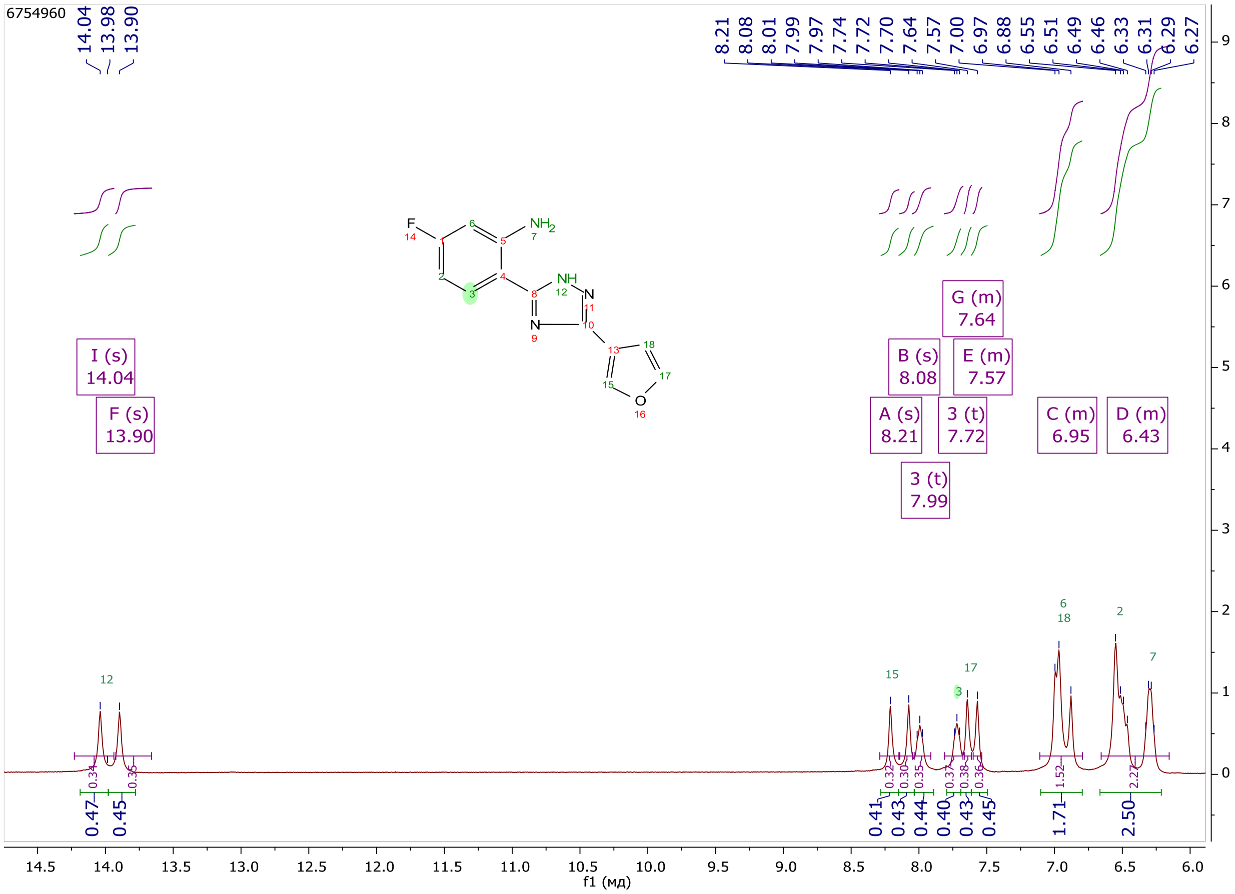

HPLC MS (methanol-water, APCI) data of compound 2.30

MaxPeak: 97.93%
Ret_Time: 1.174 min

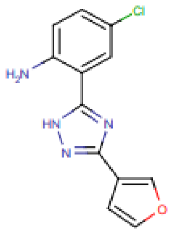

Mol Wt 260.68
Exact Mass 260.05

#	Time	Area%
1	1.174	97.93
2	1.523	2.07

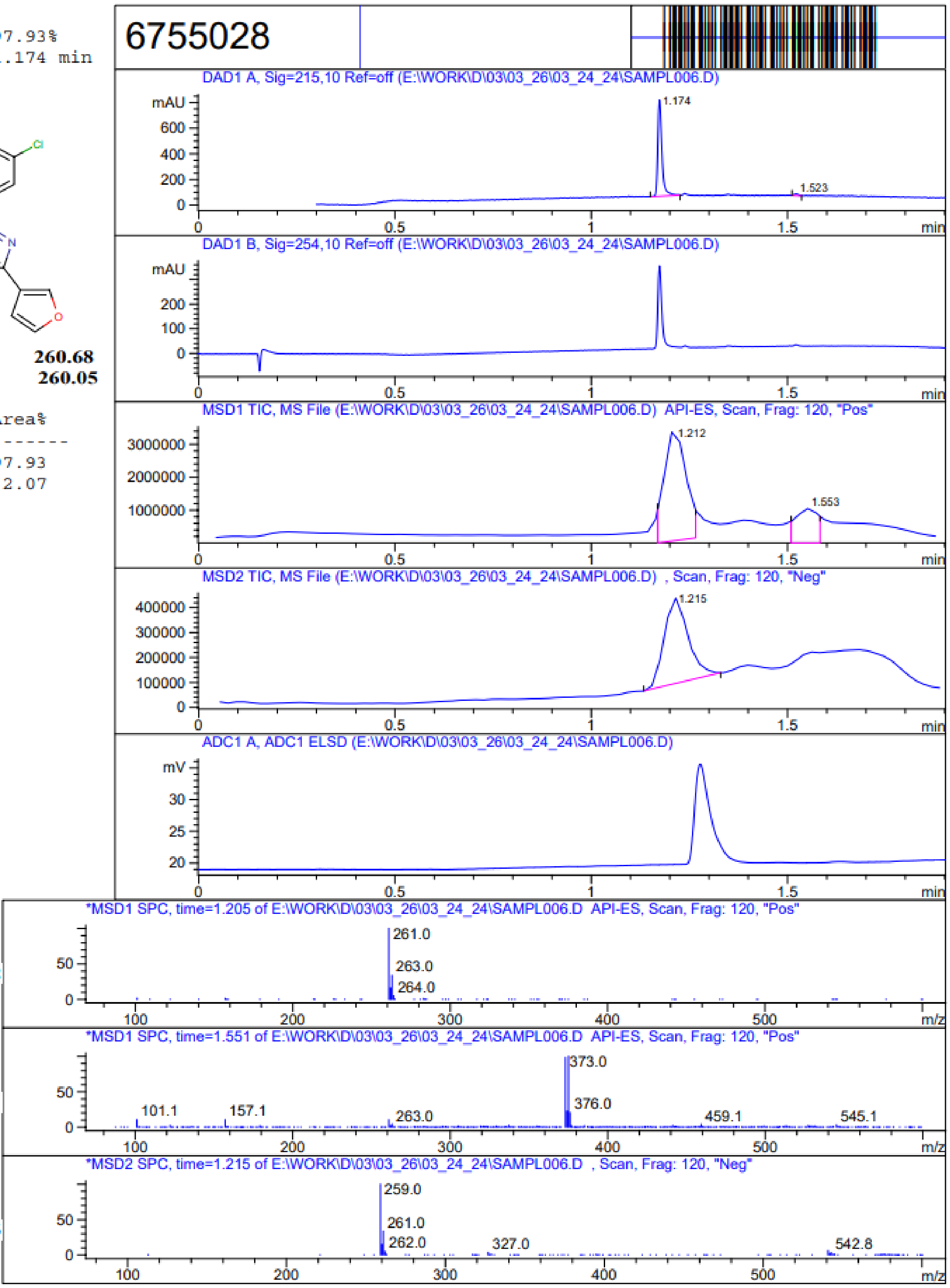

RT 1.212

RT 1.553

RT 1.215

^{1}H NMR (500 MHz) spectrum of compound 2.30 (10 mM in DMSO-d6 at 298K).

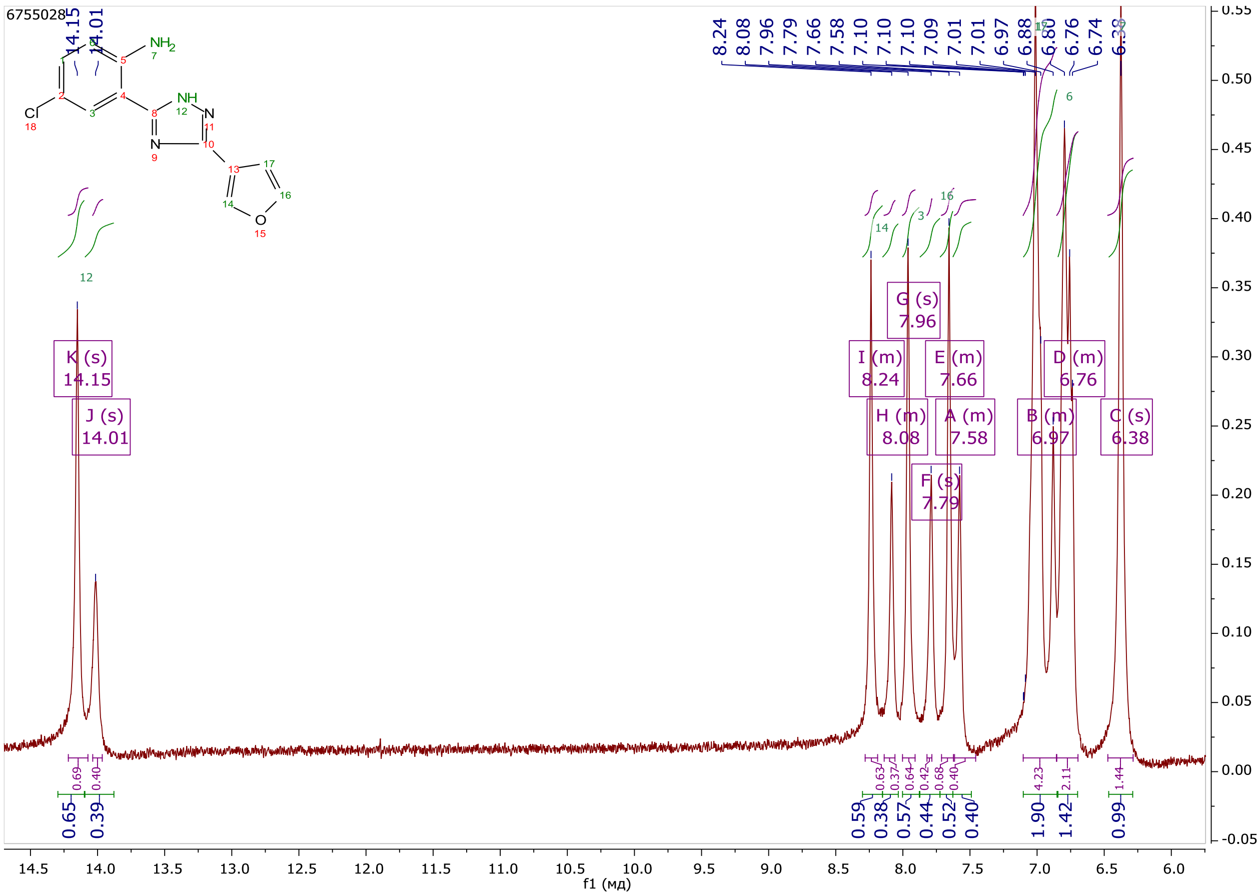

^{1}H NMR (500 MHz) spectrum of compound 2.31 (10 mM in DMSO-d6 at 298K).

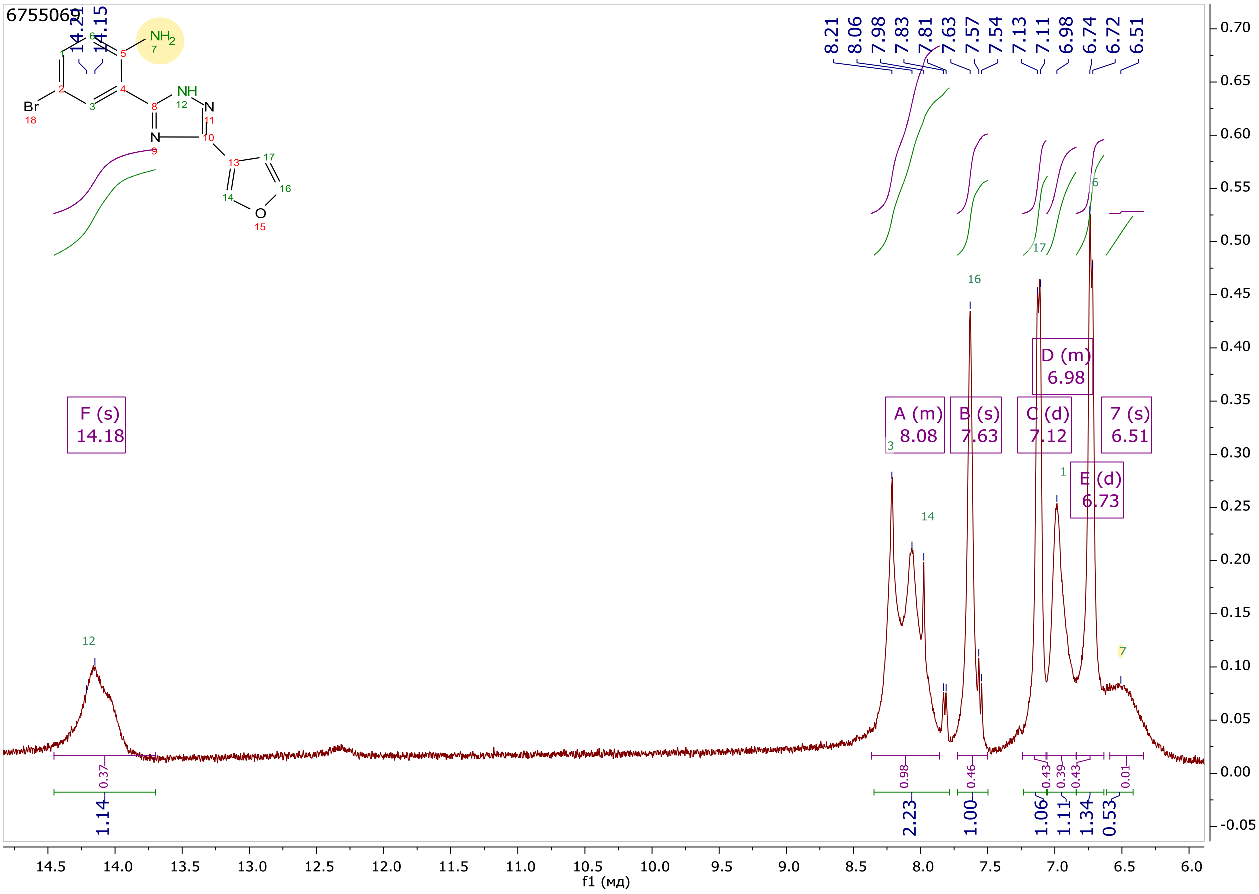

^{1}H NMR (500 MHz) spectrum of compound 2.32 (10 mM in DMSO-d6 at 298K).

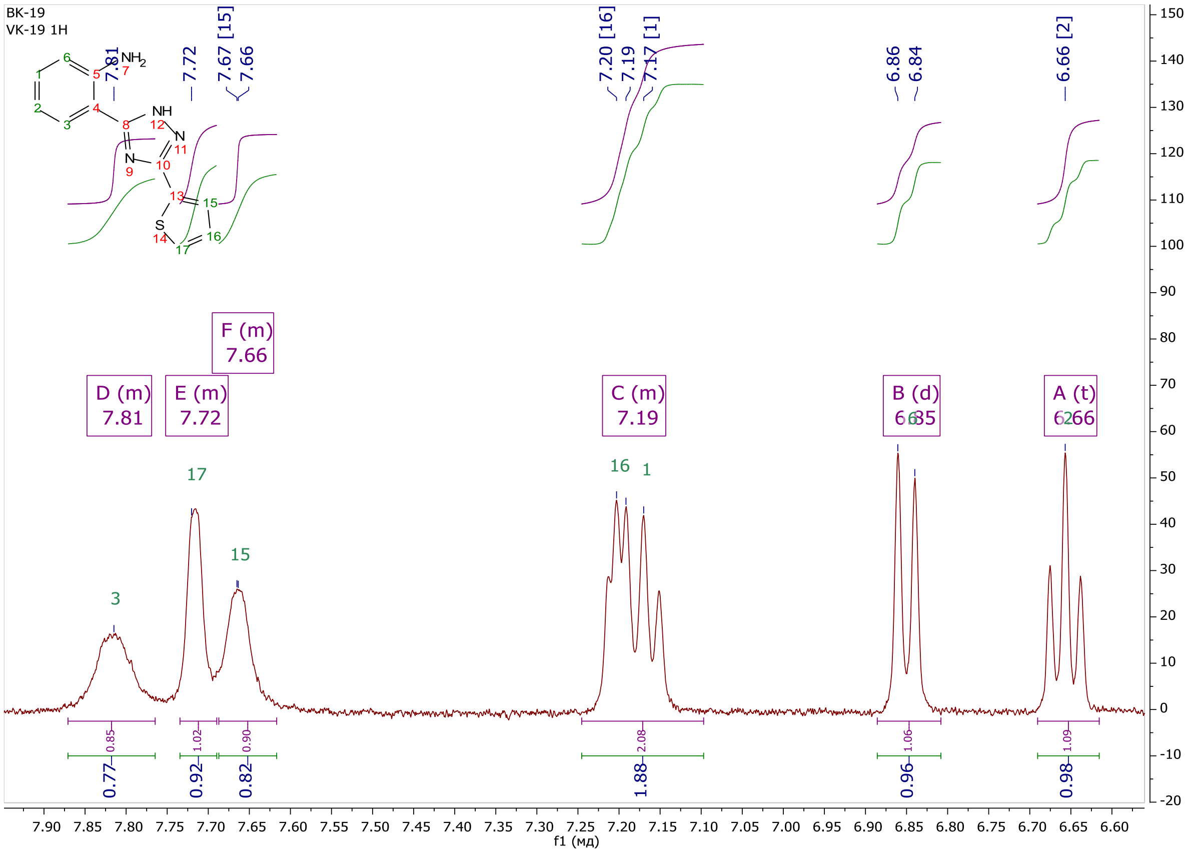

^{13}C NMR (125 MHz) spectrum of compound 2.32 (DMSO-d6 at 298K).

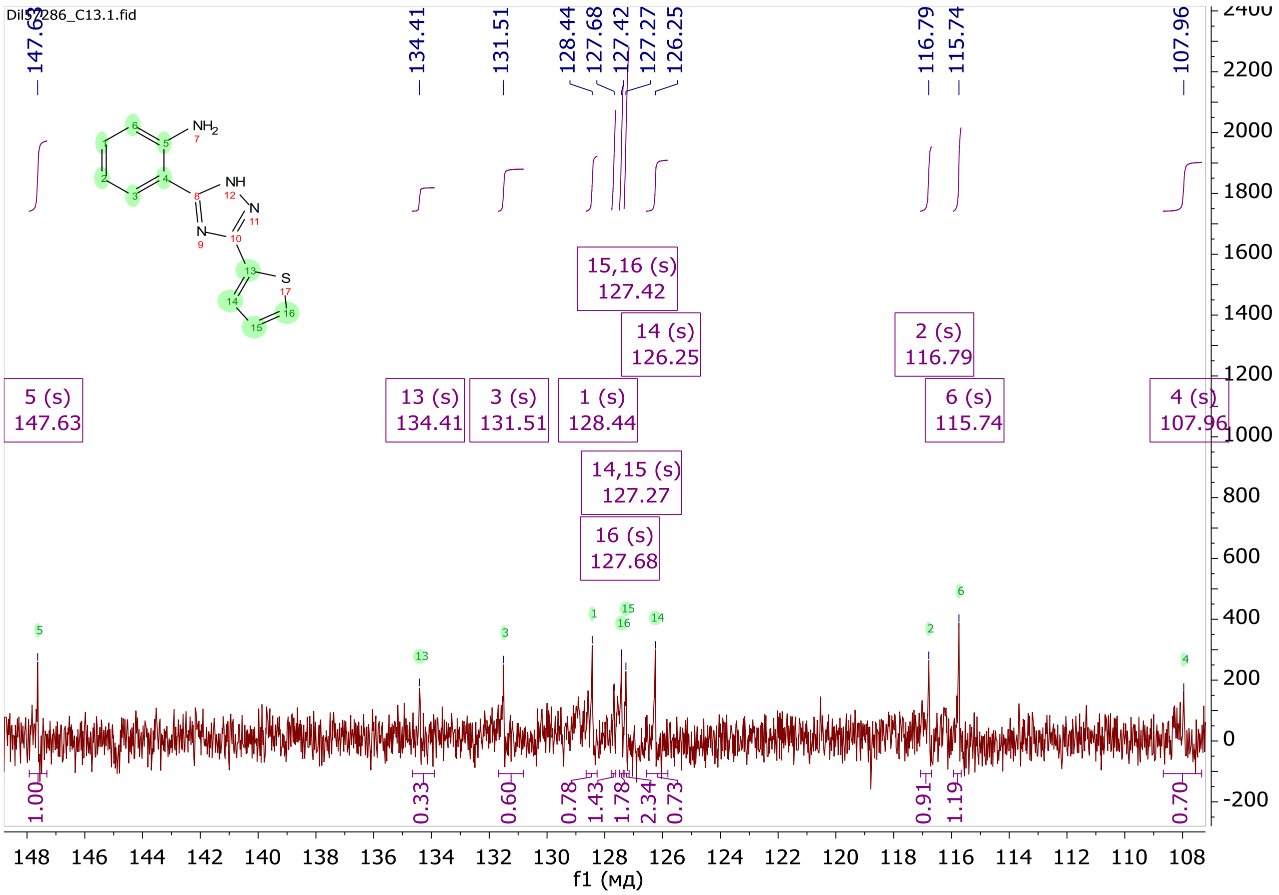

HPLC MS (methanol-water, APCI) data of compound 2.33

MaxPeak: 100.00%
Ret_Time: 1.244 min

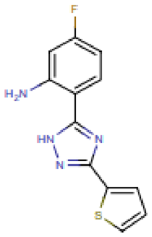

Mol Wt 260.29
Exact Mass 260.06

#	Time	Area%
1	1.244	100.00

RT 1.264

RT 1.264

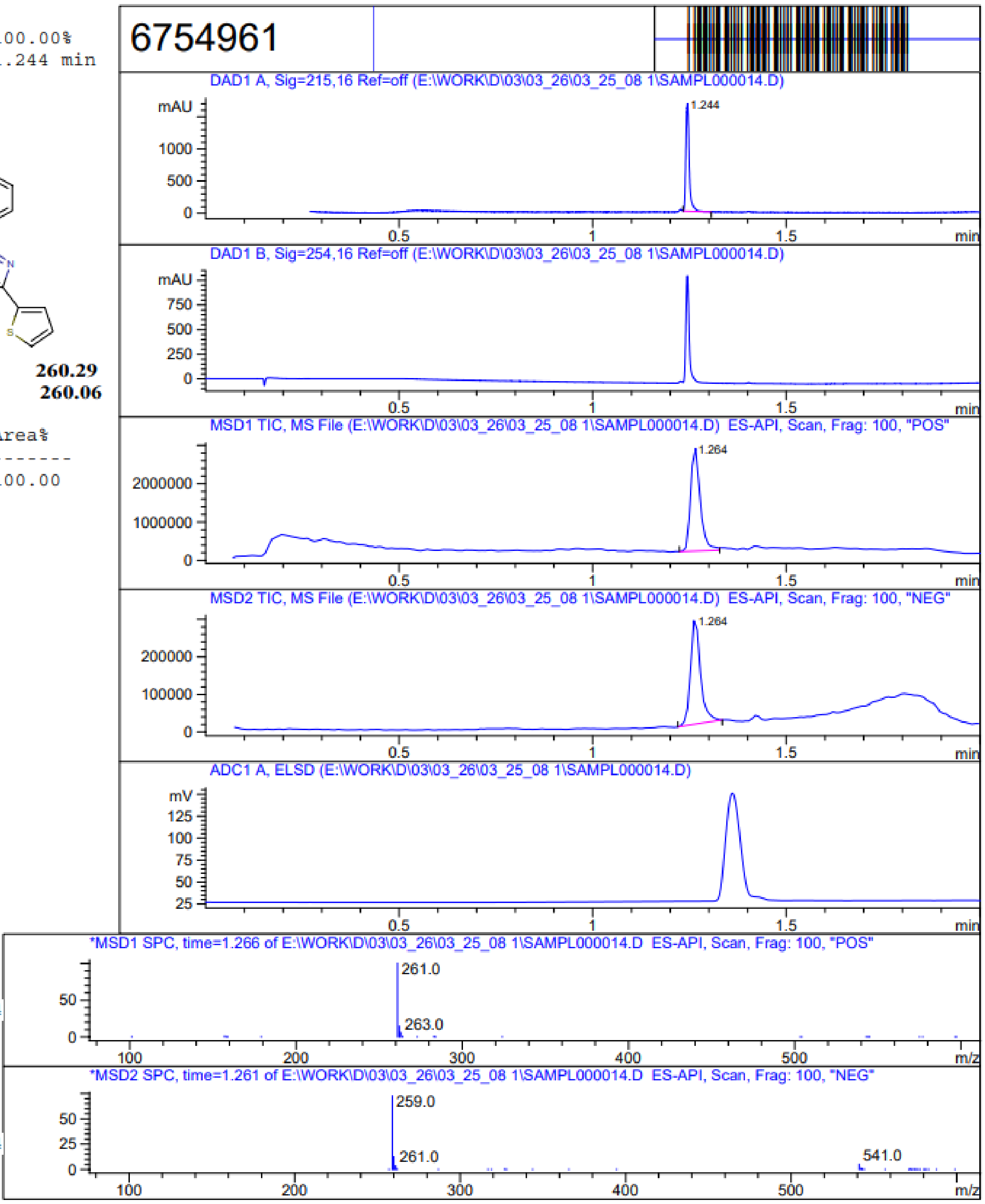

^{1}H NMR (500 MHz) spectrum of compound 2.33 (10 mM in DMSO-d6 at 298K).

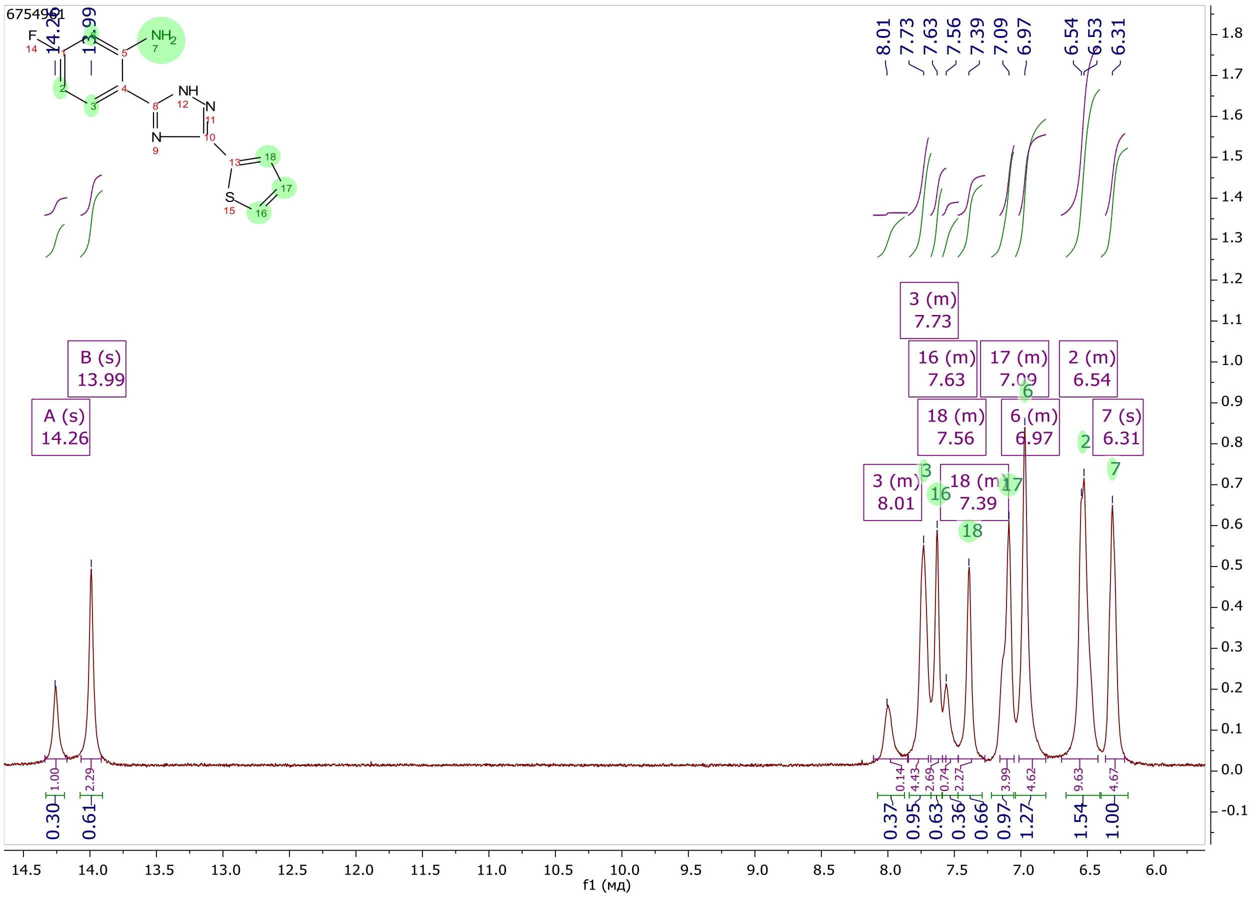

HPLC MS (methanol-water, APCI) data of compound 2.34

MaxPeak: 100.00%
Ret_Time: 1.237 min

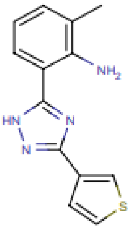

Mol Wt 256.33
Exact Mass 256.09

#	Time	Area%
1	1.237	100.00

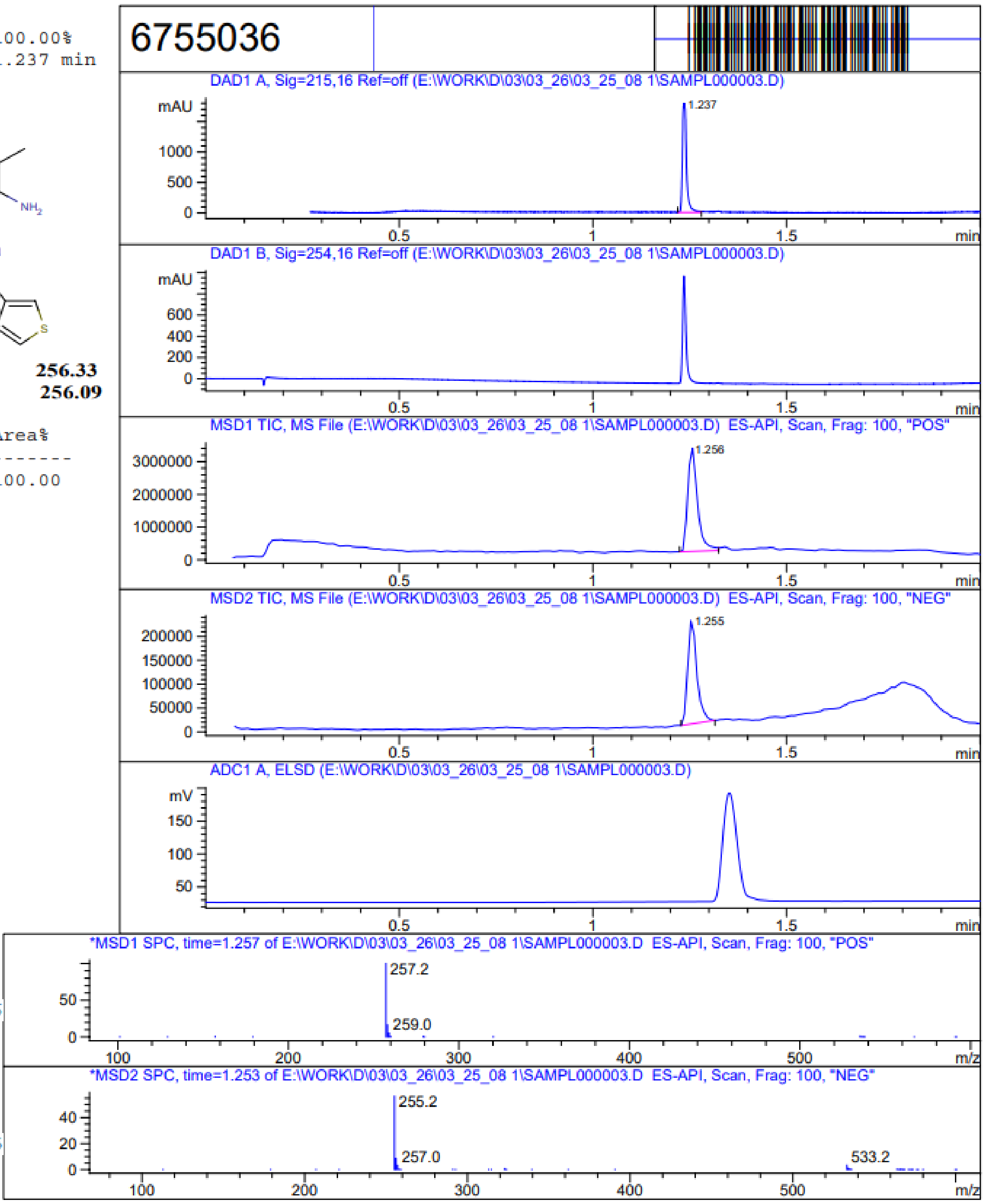

RT 1.256

RT 1.255

^{1}H NMR (500 MHz) spectrum of compound 2.34 (10 mM in DMSO-d6 at 298K).

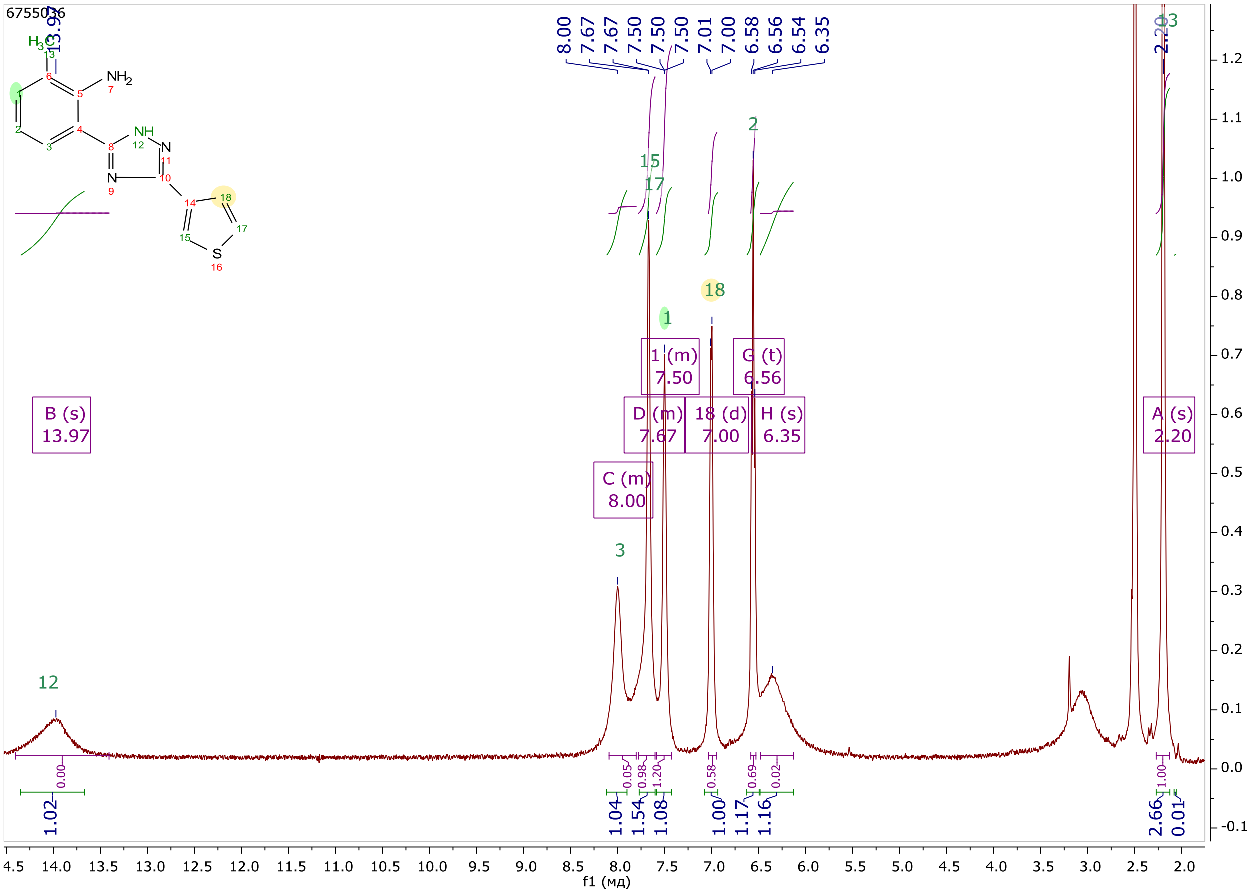

HPLC MS (methanol-water, APCI) data of compound 2.35

MaxPeak: 98.95%
Ret_Time: 1.229 min

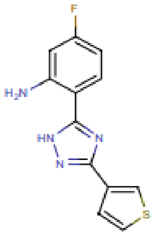

Mol Wt 260.29
Exact Mass 260.06

#	Time	Area%
1	1.229	98.95
2	1.528	1.05

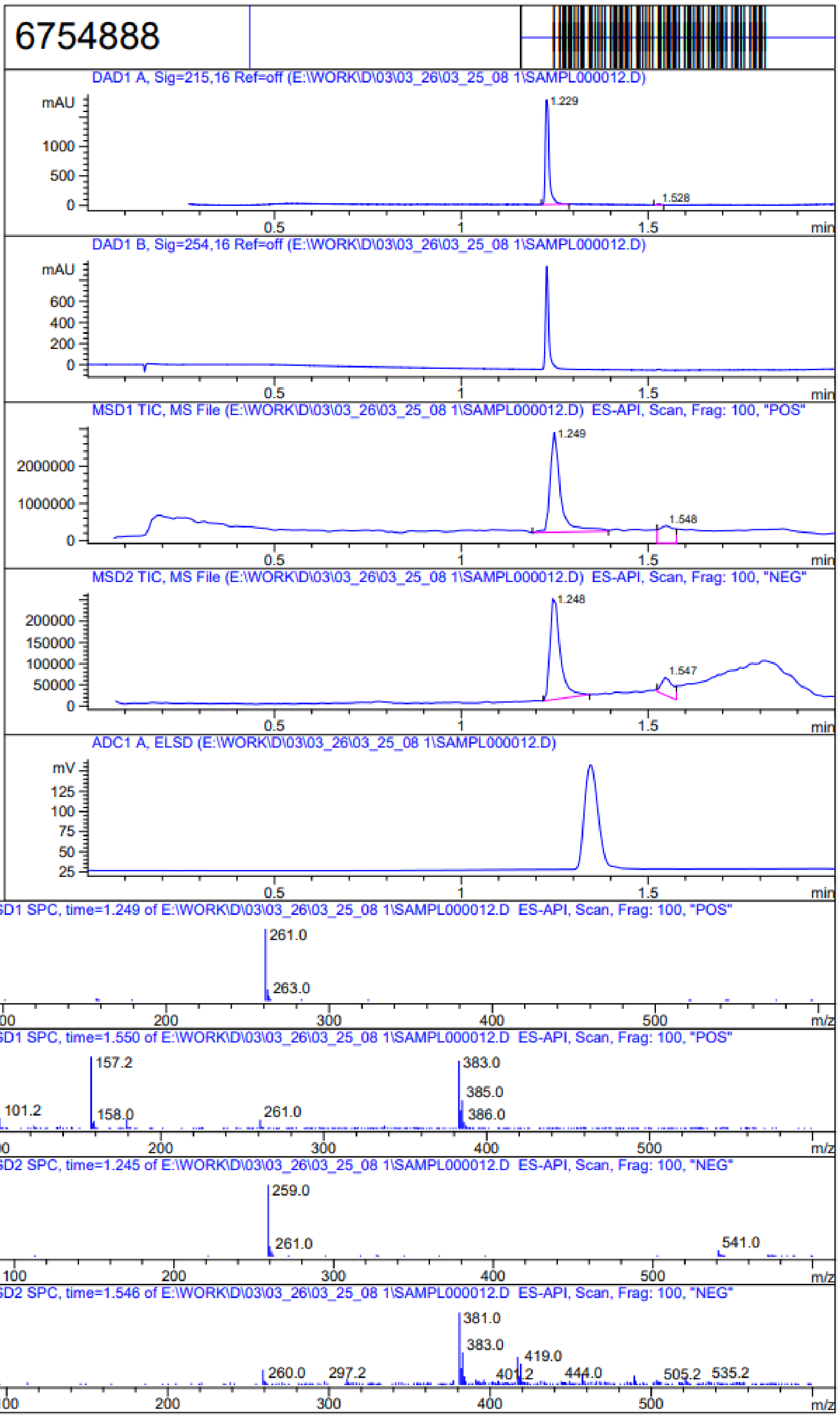

^{1}H NMR (500 MHz) spectrum of compound 2.35 (10 mM in DMSO-d6 at 298K).

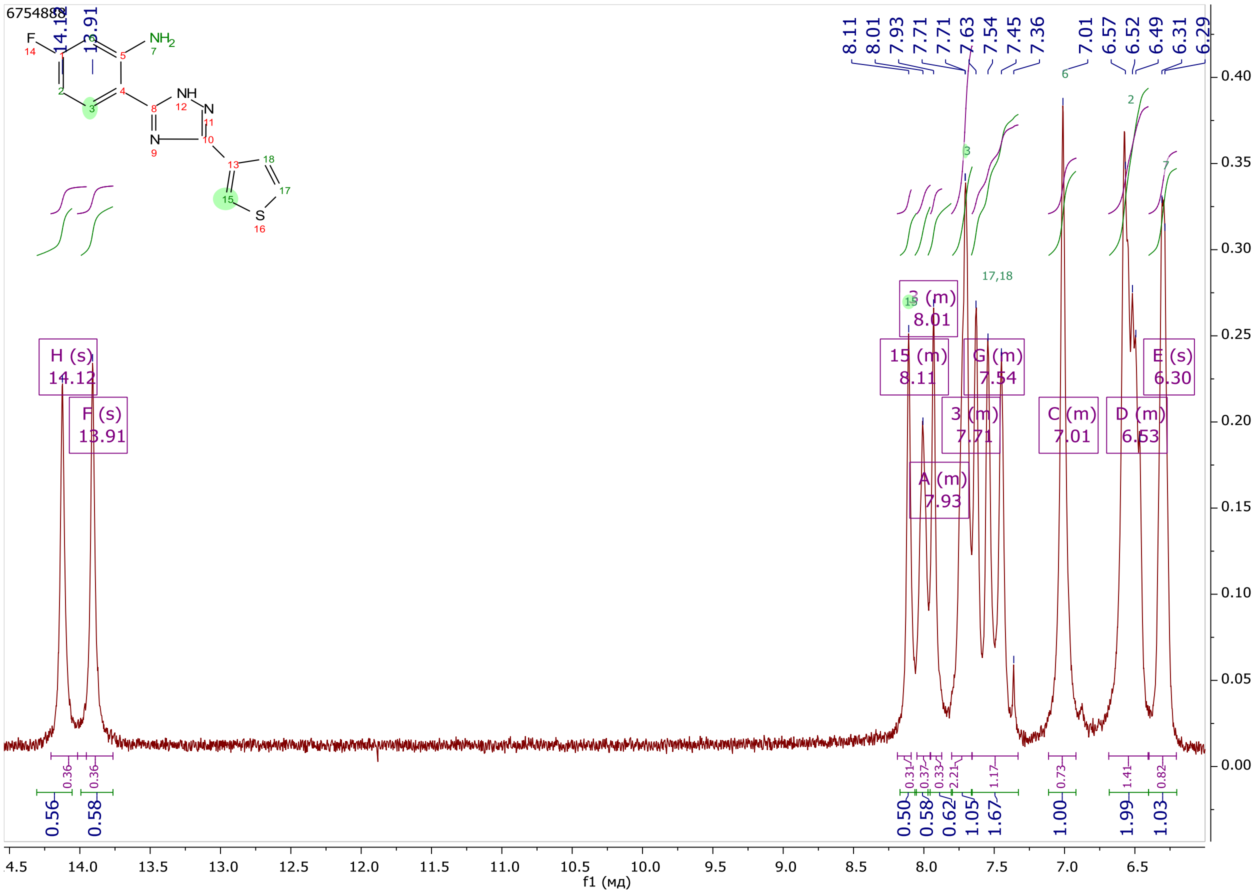

HPLC MS (methanol-water, APCI) data of compound 2.36

MaxPeak: 100.00%
Ret_Time: 1.241 min

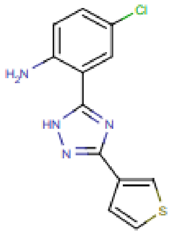

Mol Wt 276.74
Exact Mass 276.03

#	Time	Area%
1	1.241	100.00

RT 1.276

RT 1.282

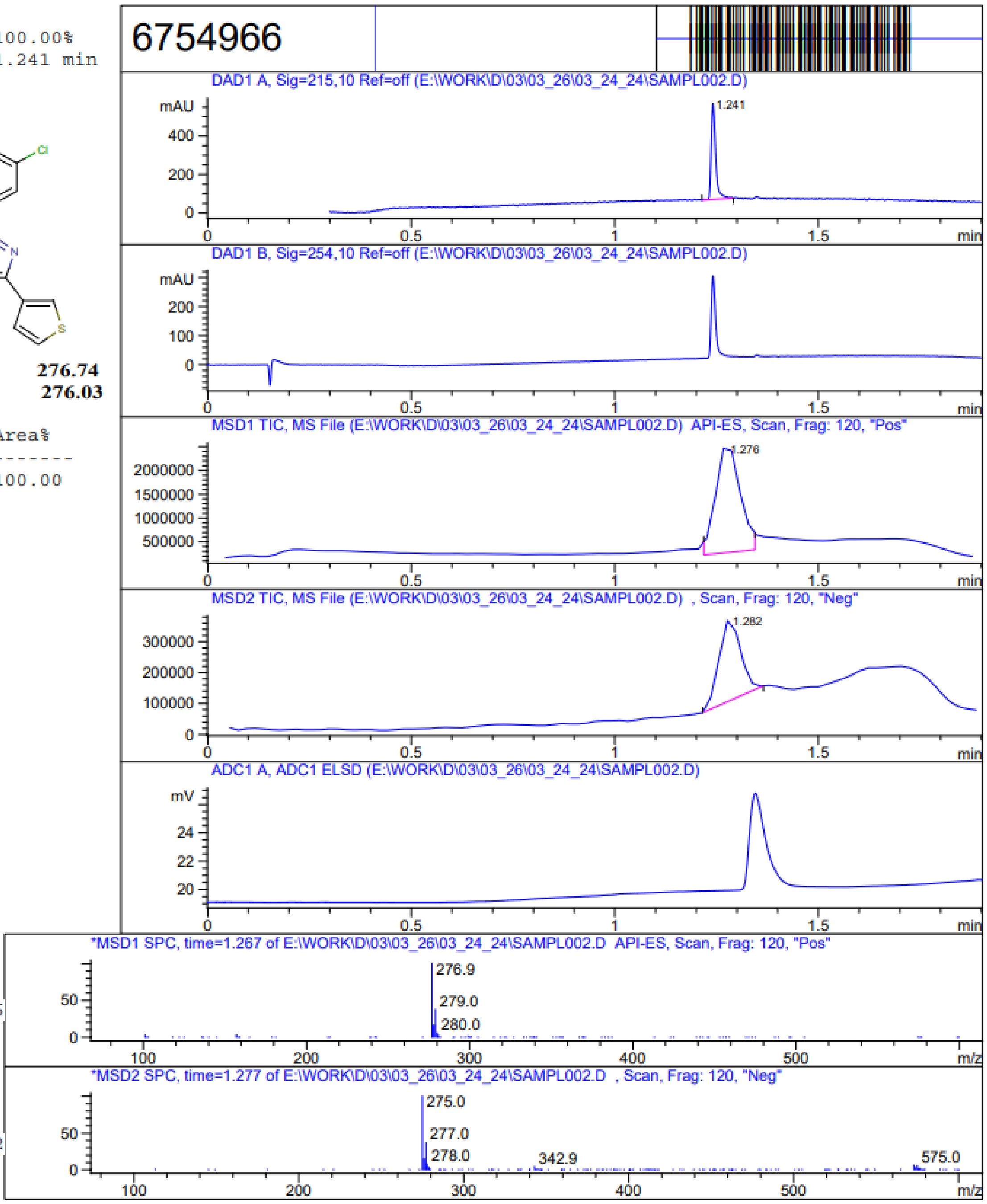

^{1}H NMR (500 MHz) spectrum of compound 2.36 (10 mM in DMSO-d6 at 298K).

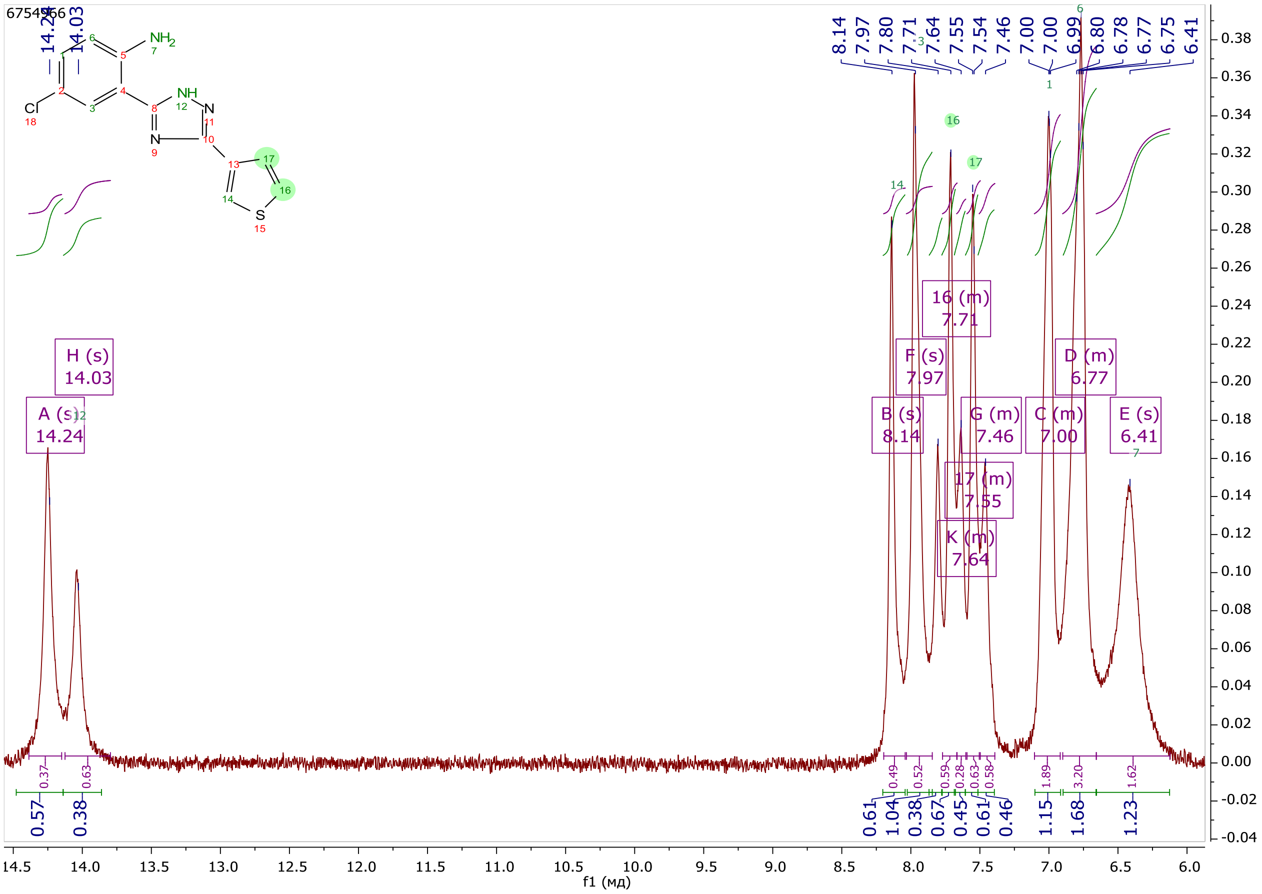

HPLC MS (methanol-water, APCI) data of compound 2.37

```
MaxPeak:   95.74%
Ret_Time: 1.297 min
```

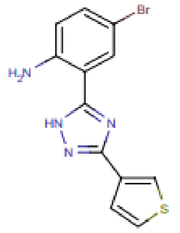

Mol Wt 321.2
Exact Mass 321.98

#	Time	Area%
1	1.270	4.26
2	1.297	95.74

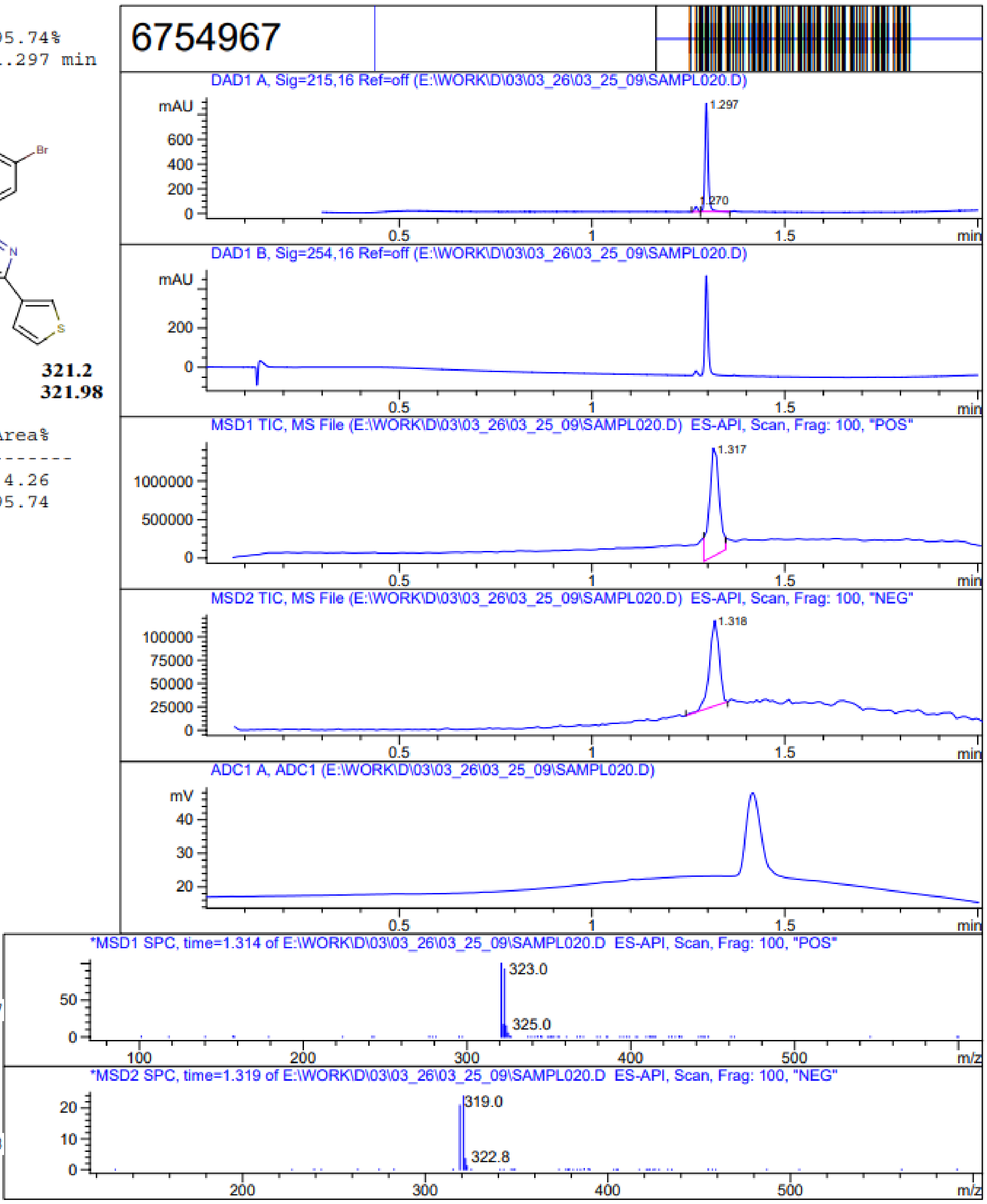

RT 1.317

RT 1.318

^{1}H NMR (500 MHz) spectrum of compound 2.37 (10 mM in DMSO-d6 at 298K).

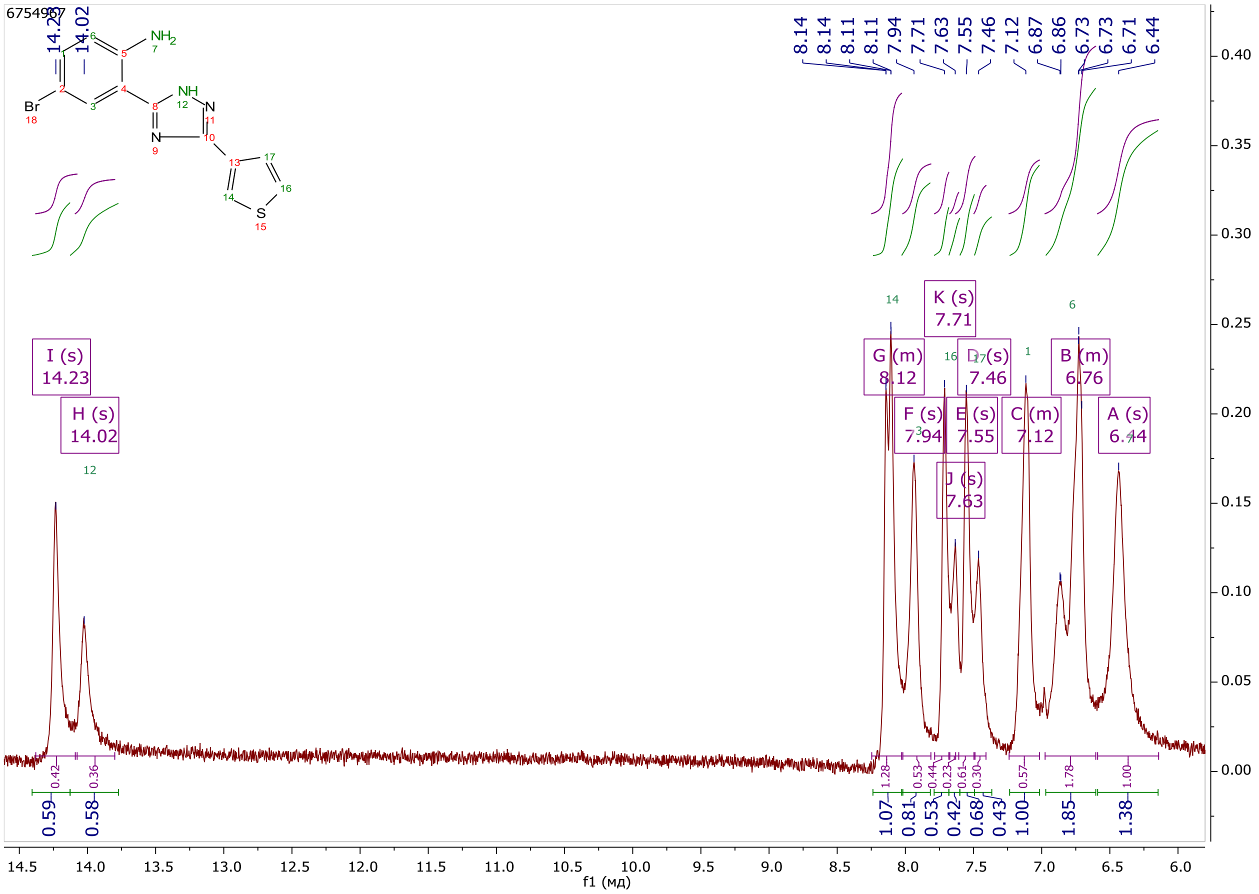

^{1}H NMR (500 MHz) spectrum of compound 2.38 (10 mM in DMSO-d6 at 298K).

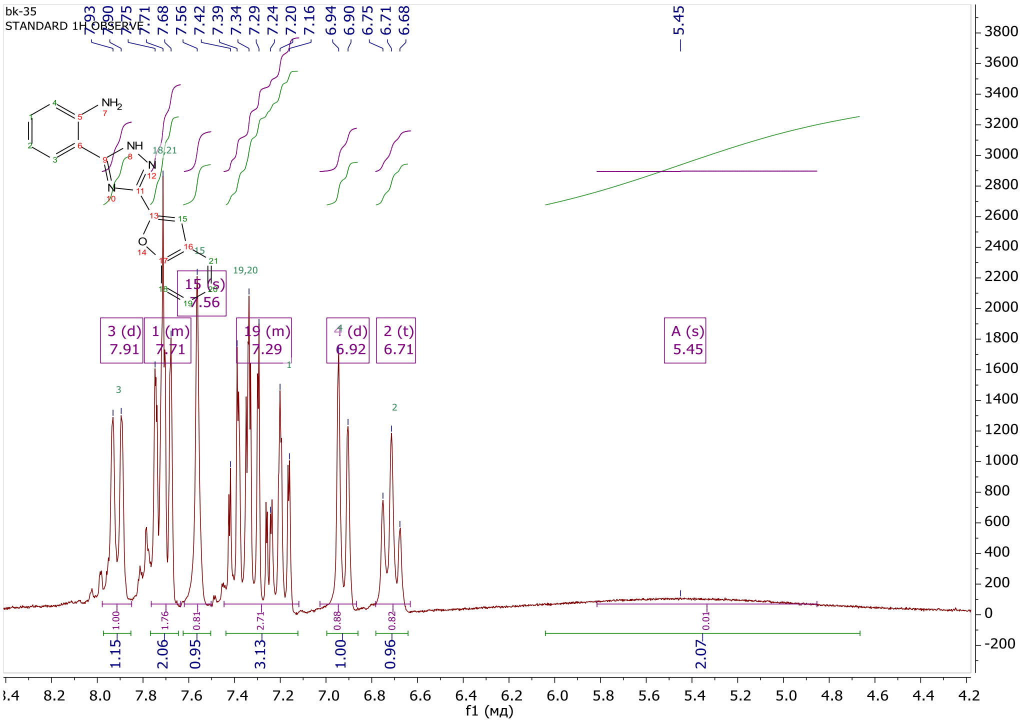

^{13}C NMR (125 MHz) spectrum of compound 2.38 (DMSO-d6 at 298K).

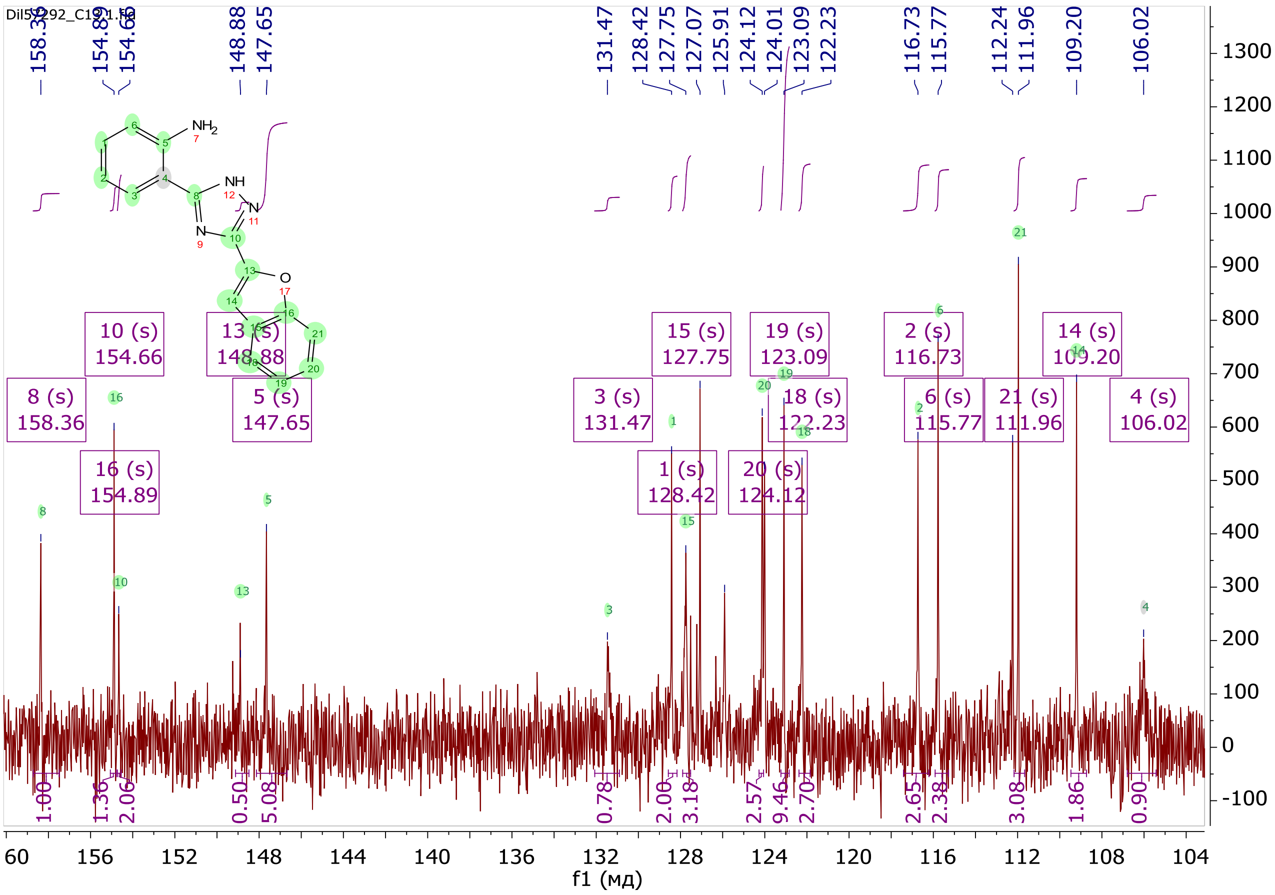

^{1}H NMR (500 MHz) spectrum of compound 2.39 (10 mM in DMSO-d6 at 298K).

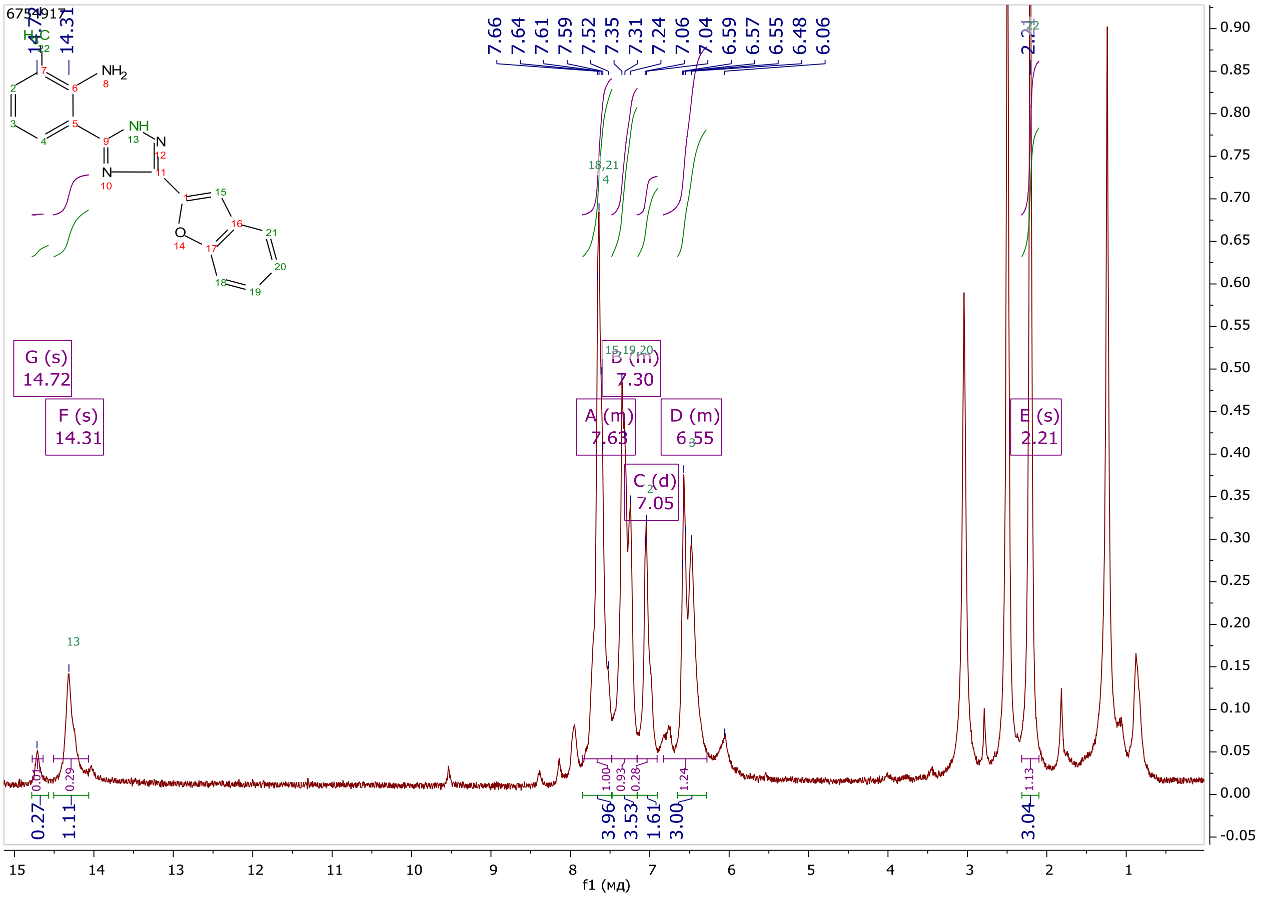

^{1}H NMR (500 MHz) spectrum of compound 2.40 (10 mM in DMSO-d6 at 298K).

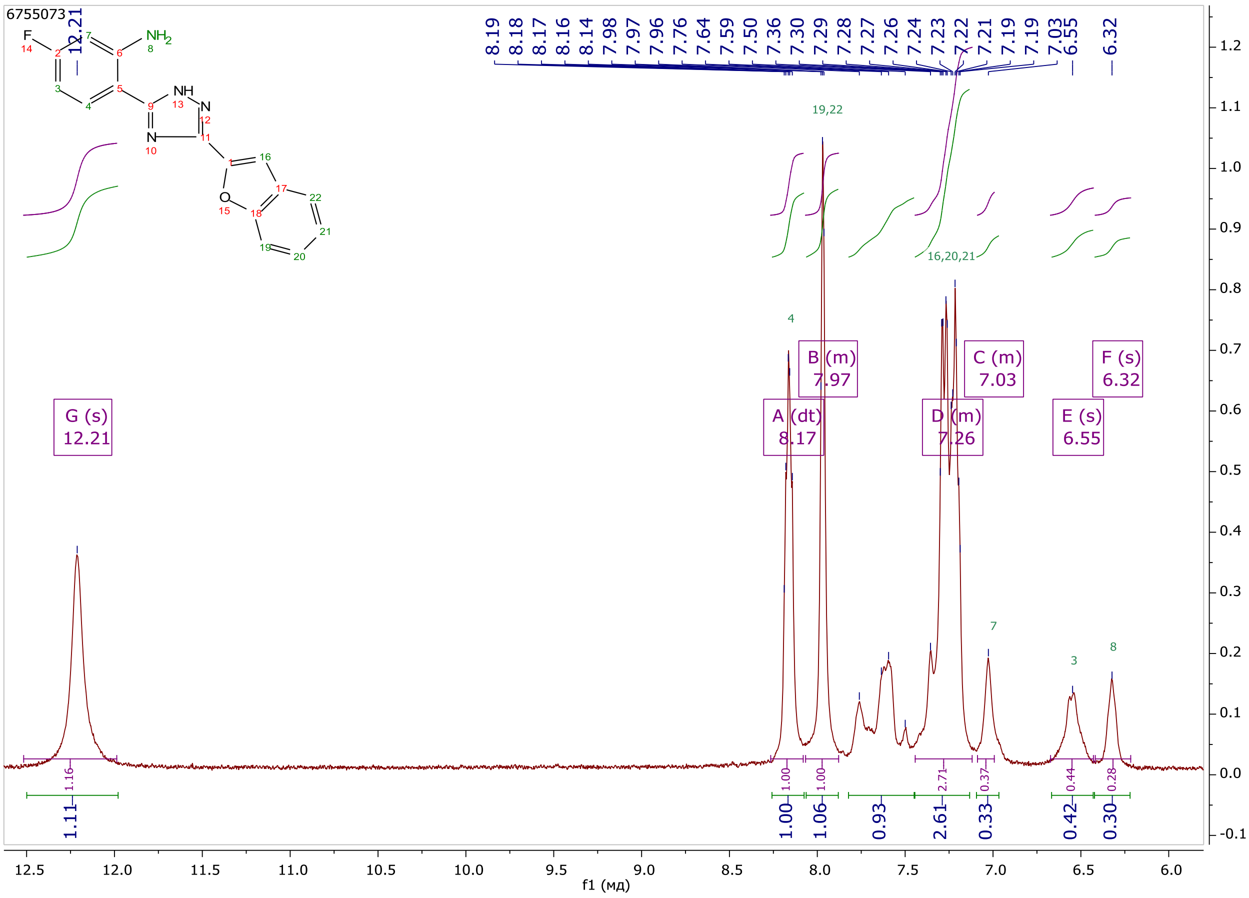

HPLC MS (methanol-water, APCI) data of compound 2.41

MaxPeak: 100.00%
Ret_Time: 1.367 min

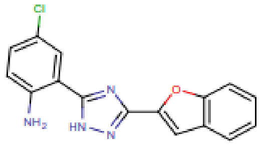

Mol Wt 310.74
Exact Mass 310.07

#	Time	Area%
1	1.367	100.00

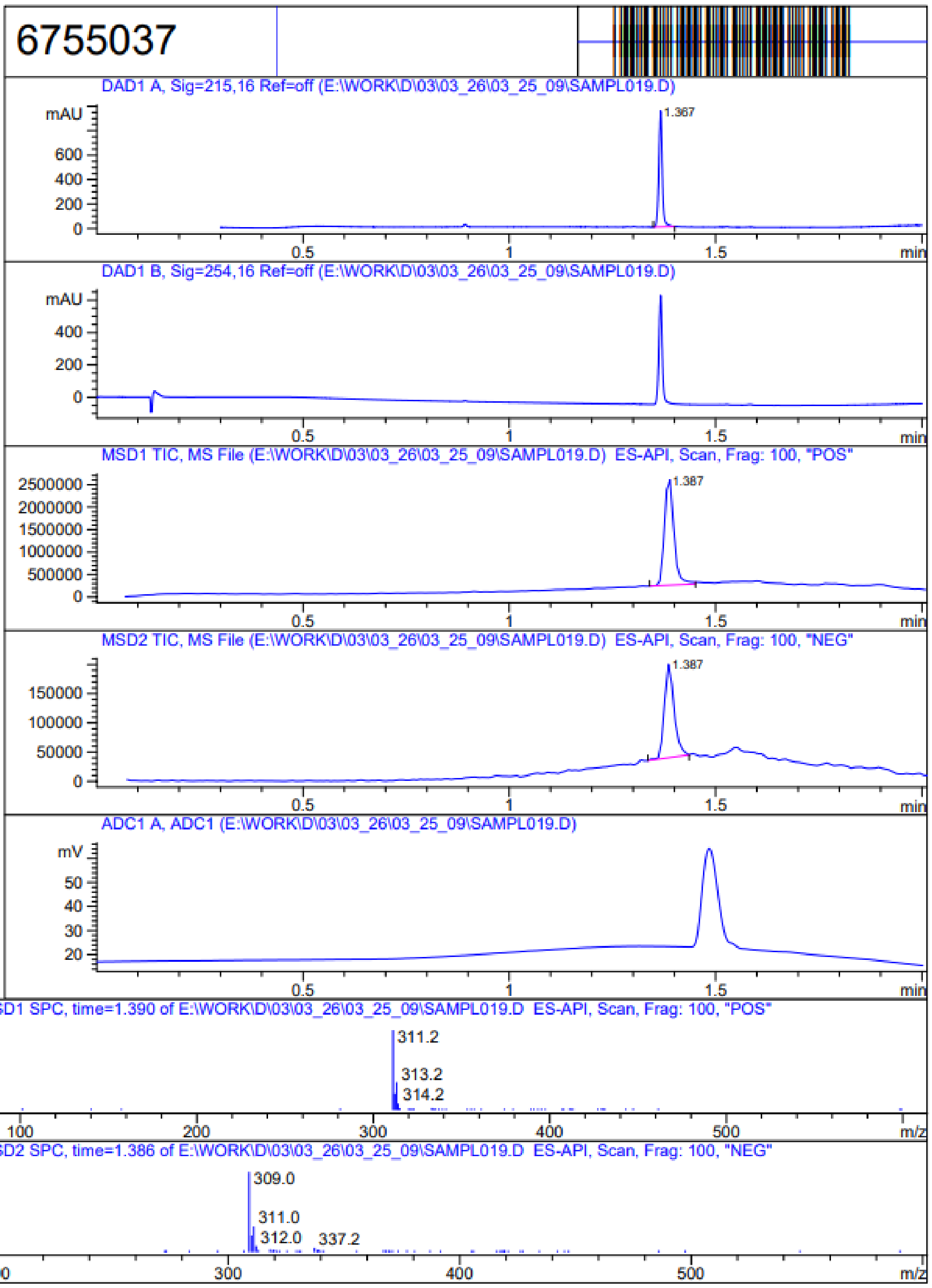

^{1}H NMR (500 MHz) spectrum of compound 2.41 (10 mM in DMSO-d6 at 298K).

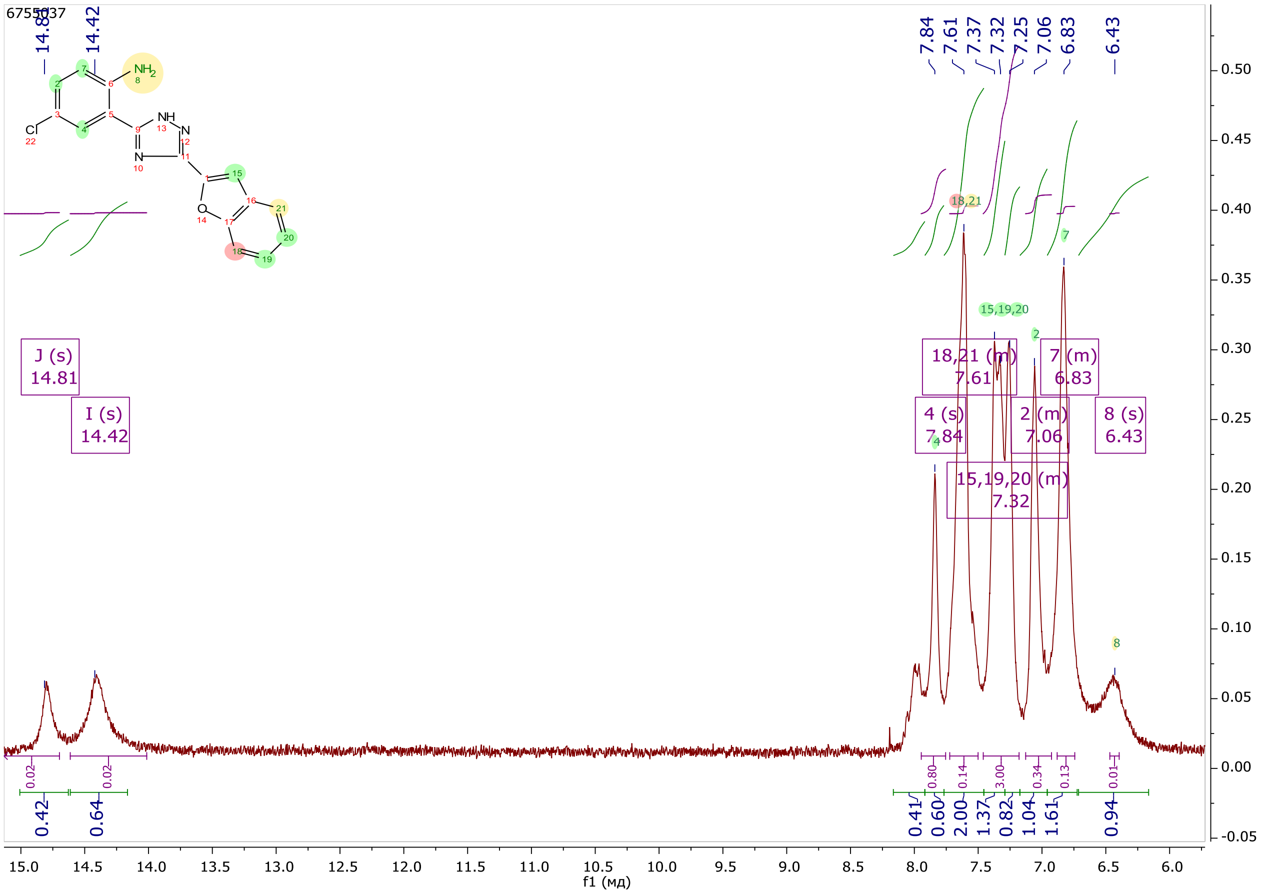

HPLC MS (methanol-water, APCI) data of compound 2.42

```
MaxPeak:   100.00%
Ret_Time:  1.374 min
```

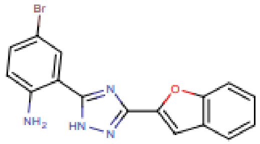

Mol Wt 355.19
Exact Mass 354.02

#	Time	Area%
1	1.374	100.00

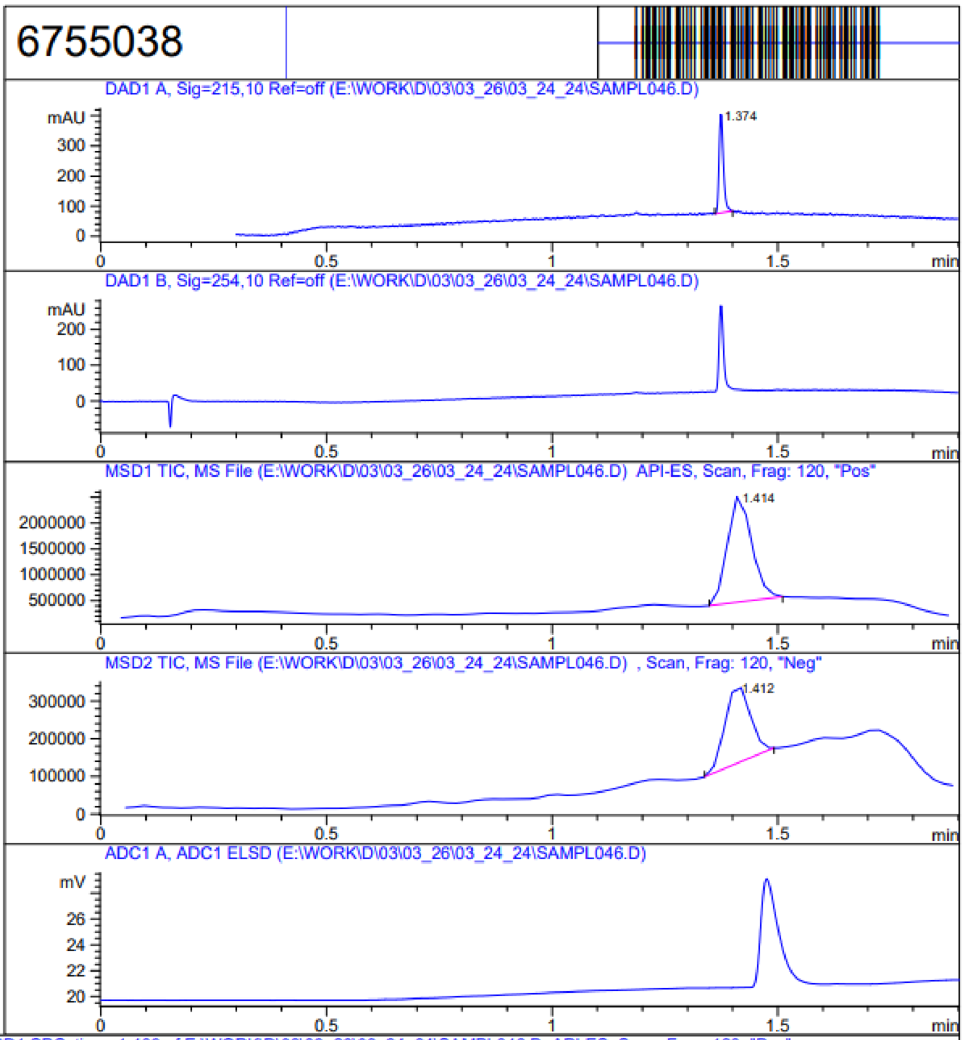

RT 1.414

RT 1.412

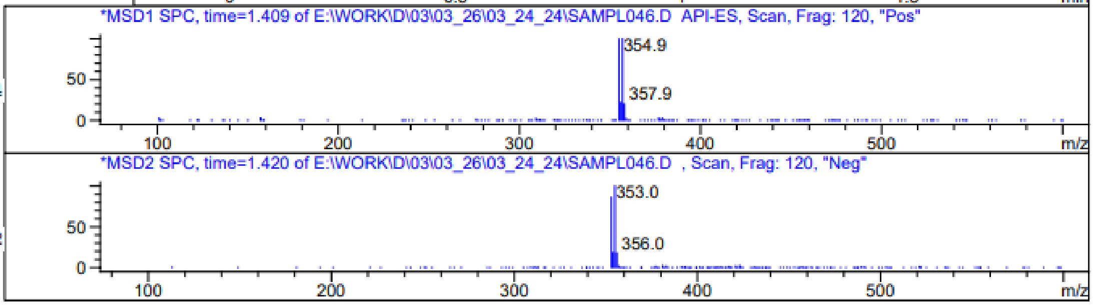

^{1}H NMR (500 MHz) spectrum of compound 2.42 (10 mM in DMSO-d6 at 298K).

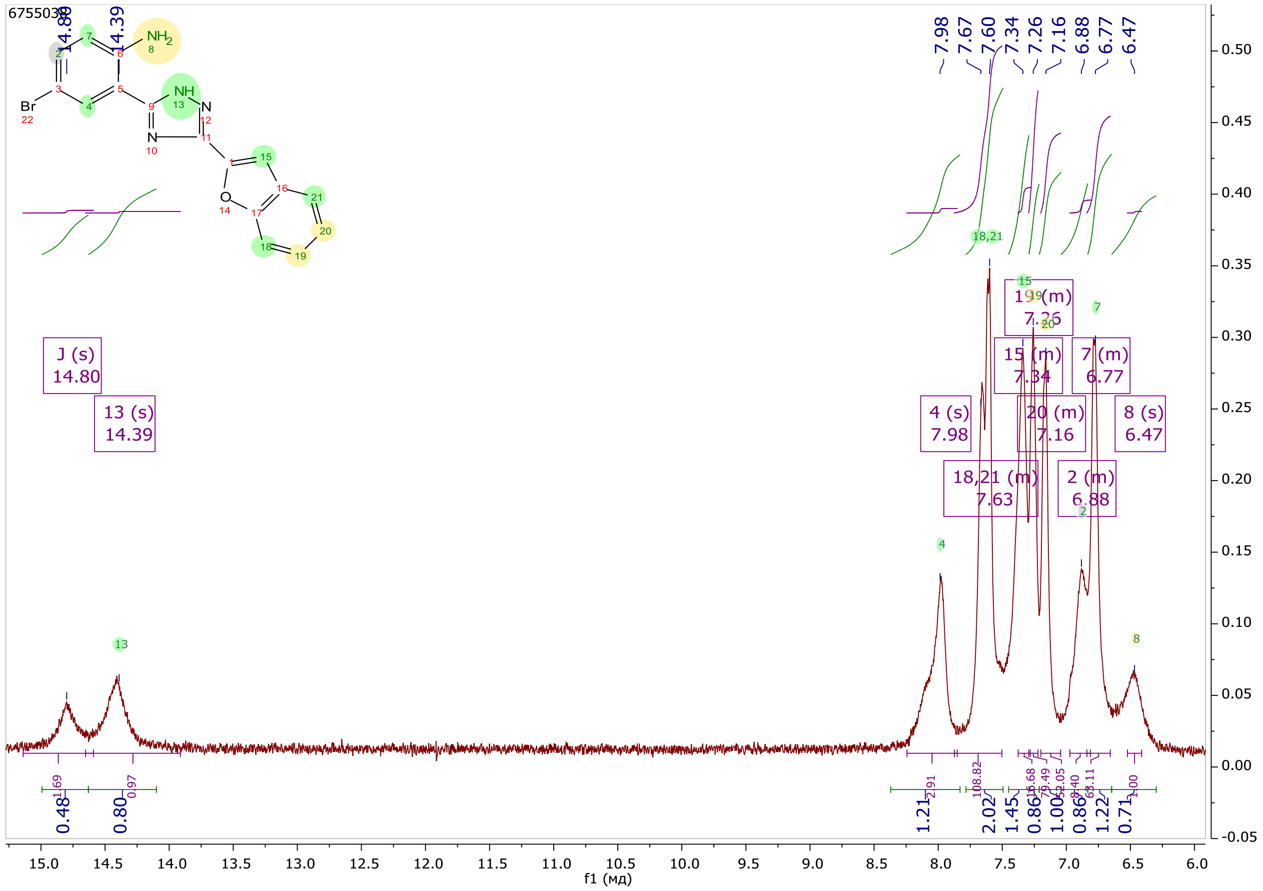

^{1}H NMR (500 MHz) spectrum of compound 2.45 (10 mM in DMSO-d6 at 298K).

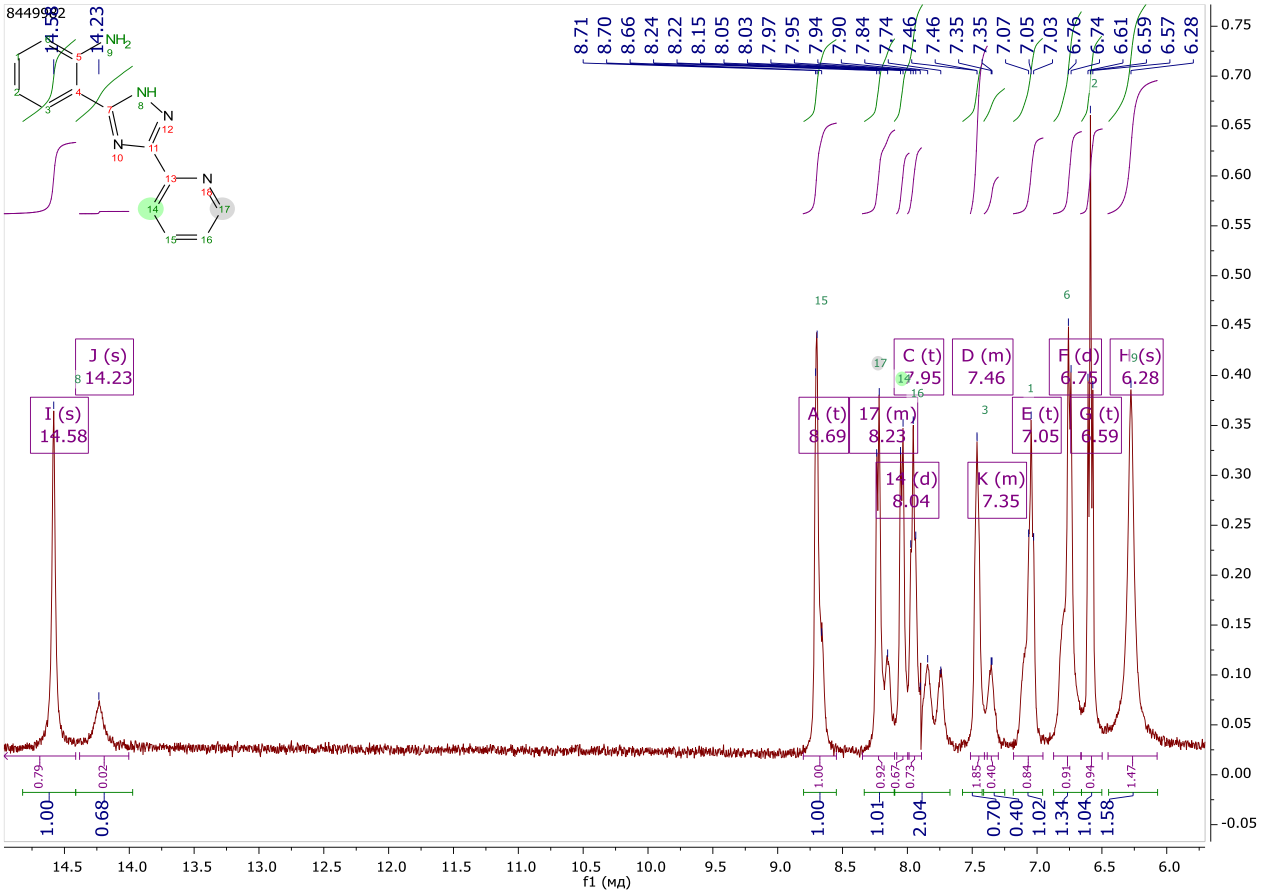

^{13}C NMR (125 MHz) spectrum of compound 2.45 (DMSO-d6 at 298K).

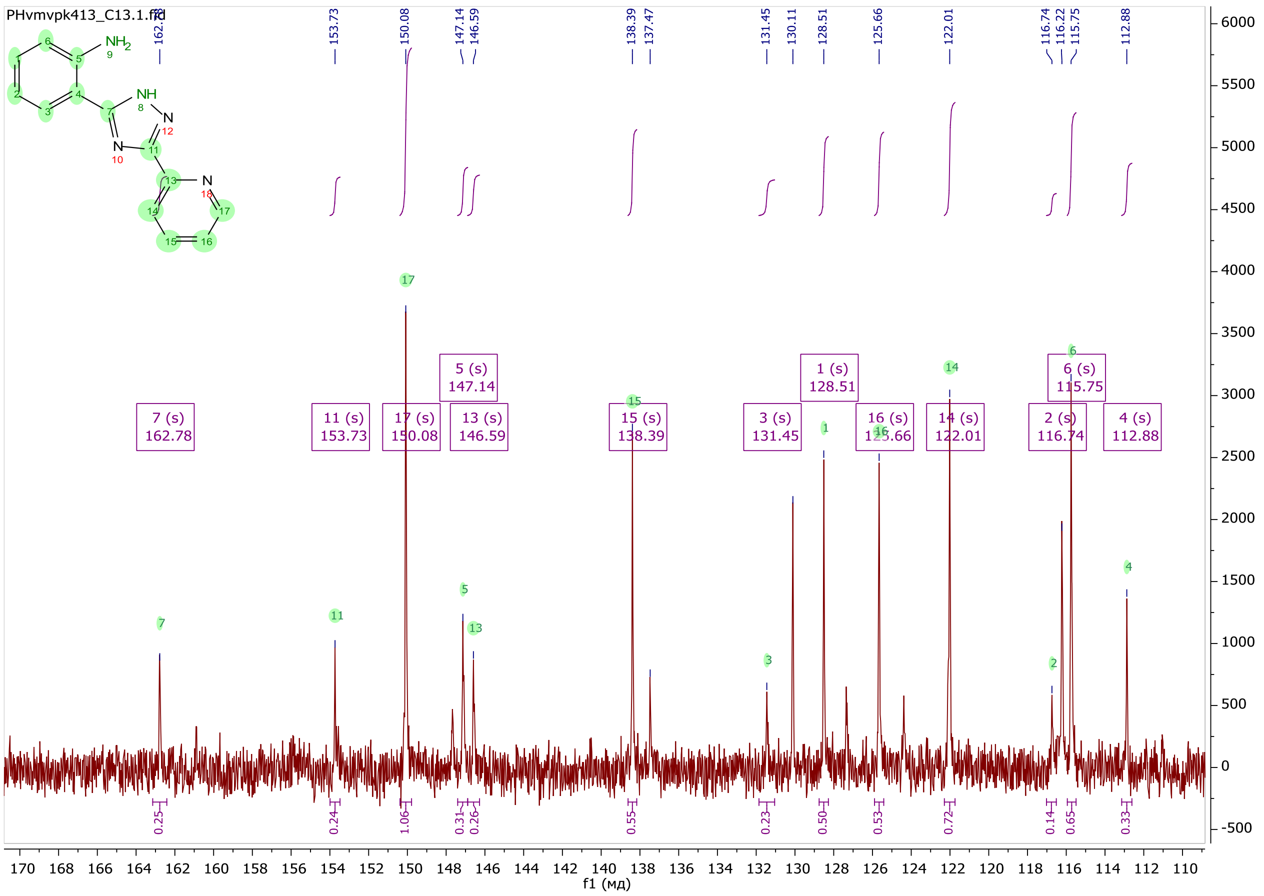

^{1}H NMR (500 MHz) spectrum of compound 2.46 (10 mM in DMSO-d6 at 298K).

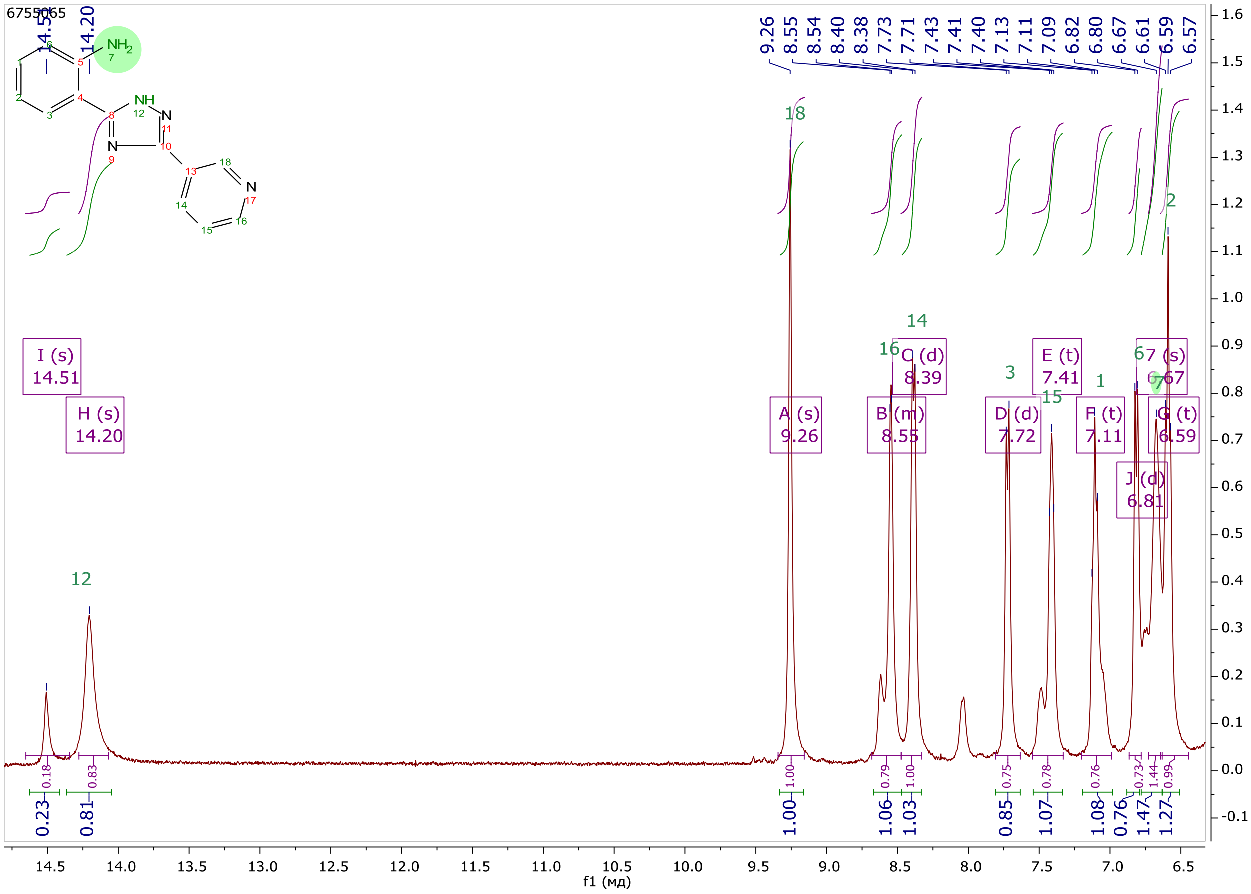

^{13}C NMR (125 MHz) spectrum of compound 2.46 (DMSO-d6 at 298K).

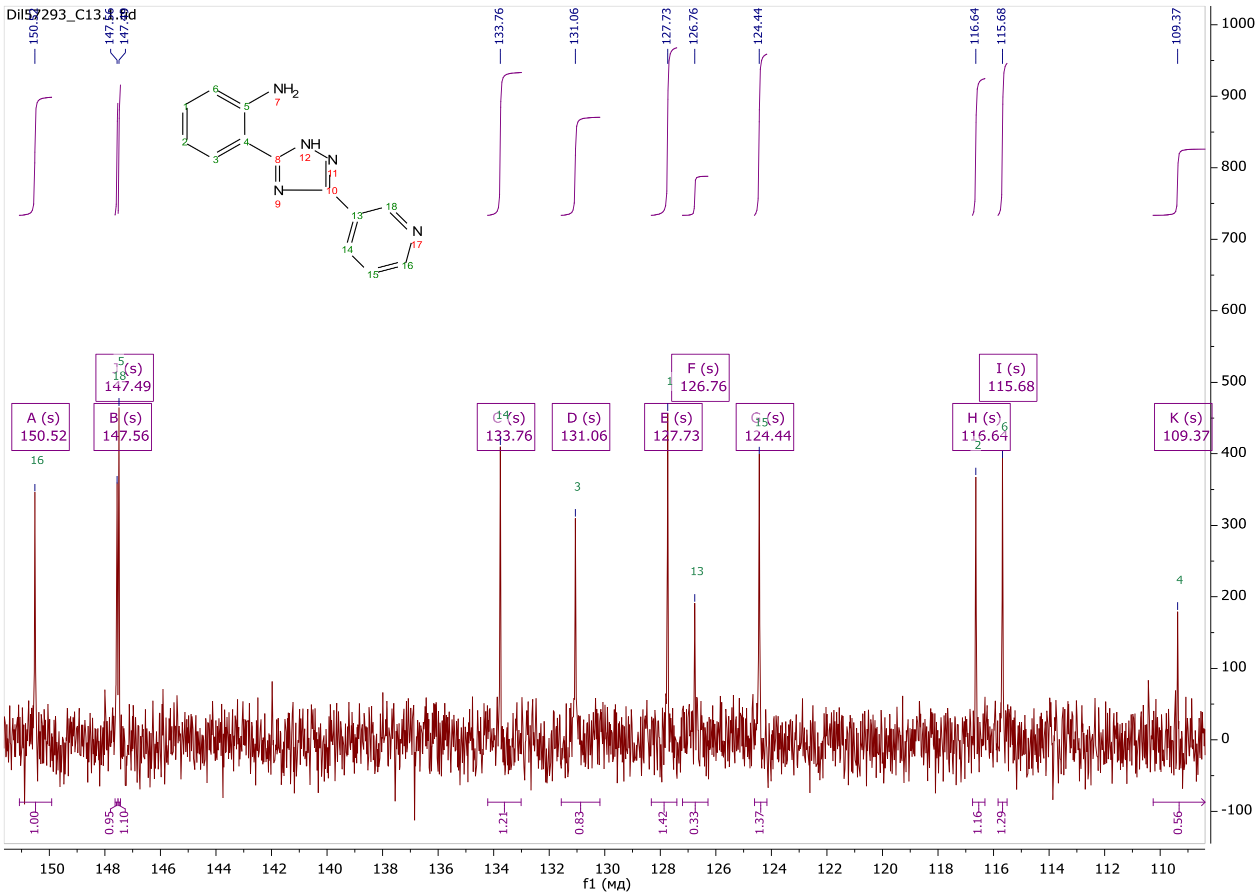

HPLC MS (methanol-water, APCI) data of compound 2.47

MaxPeak: 97.72%
Ret_Time: 0.751 min

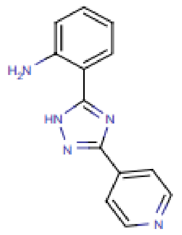

Mol Wt 237.26
Exact Mass 237.11

#	Time	Area%
1	0.751	97.72
2	0.787	2.28

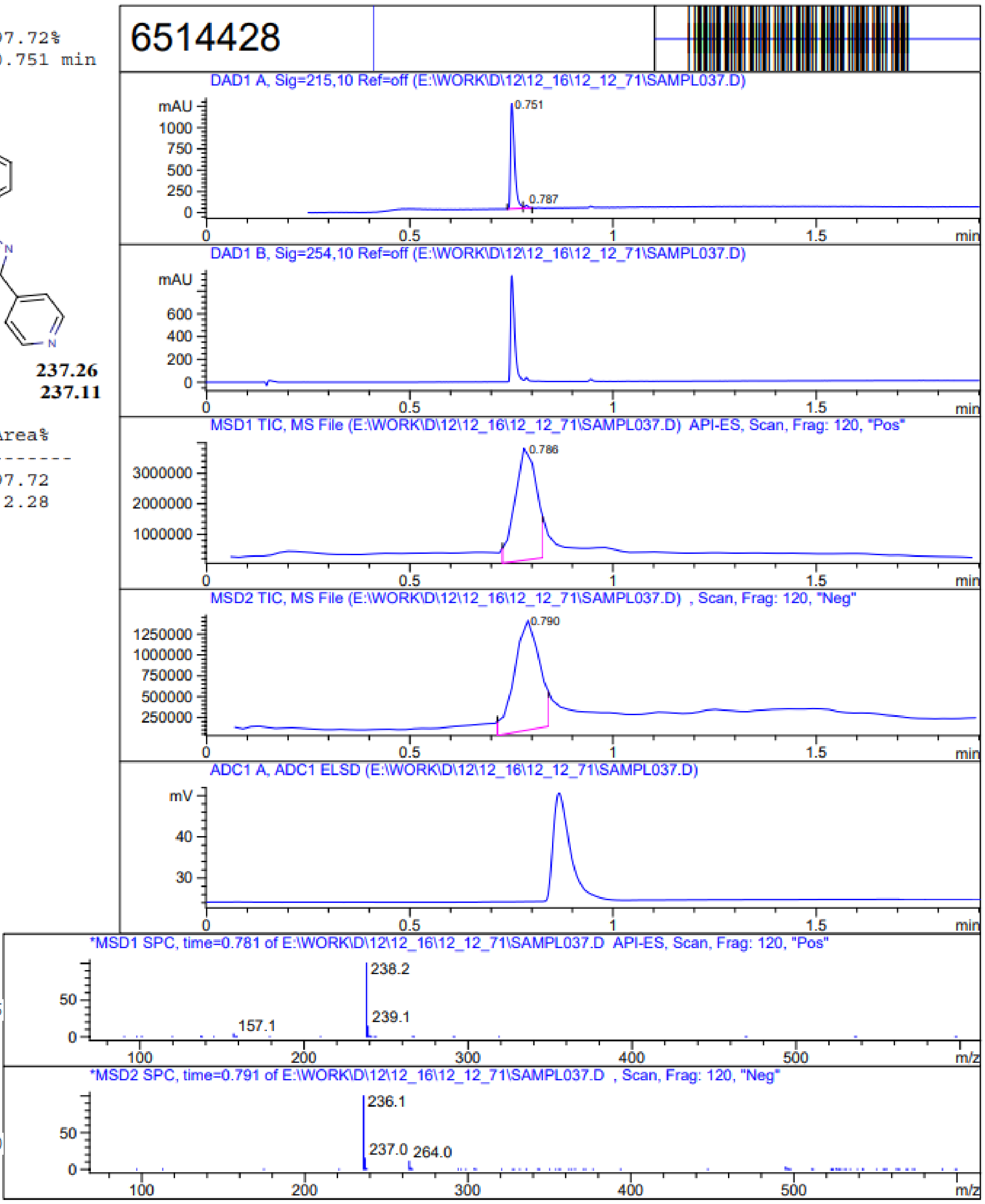

RT 0.786

RT 0.790

^{1}H NMR (500 MHz) spectrum of compound 2.47 (10 mM in DMSO-d6 at 298K).

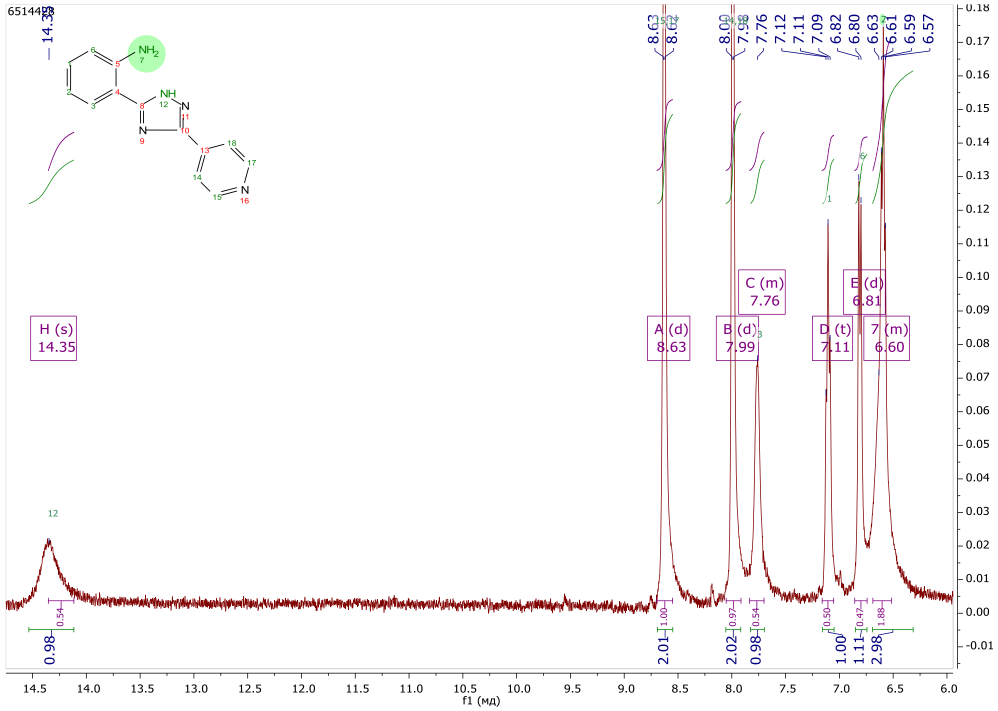

^{13}C NMR (125 MHz) spectrum of compound 2.47 (DMSO-d6 at 298K).

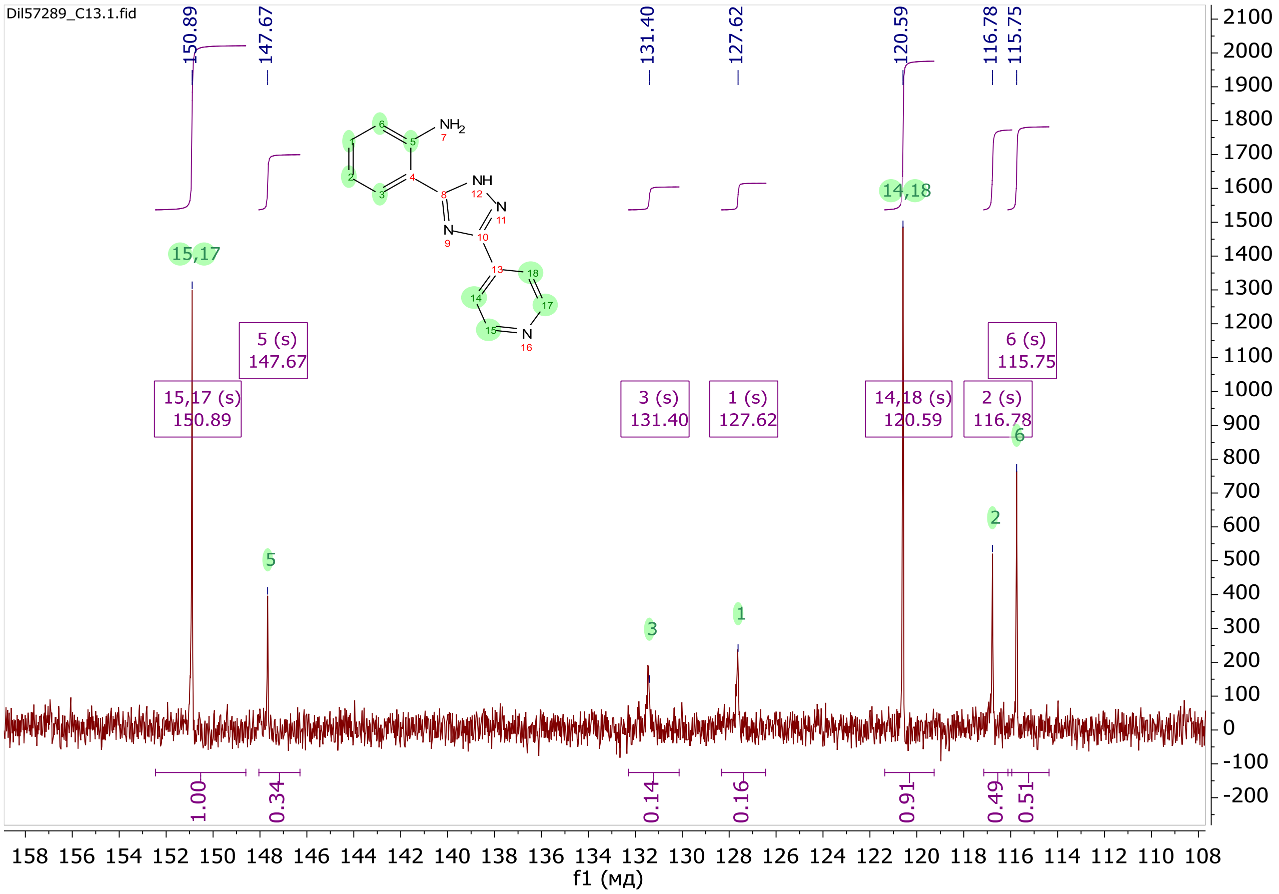

HPLC MS (methanol-water, APCI) data of compound 2.48

```
MaxPeak:   95.05%
Ret_Time:  0.944 min
```

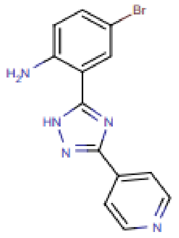

Mol Wt 316.16
Exact Mass 315.02

#	Time	Area%
1	0.944	95.05
2	1.359	4.95

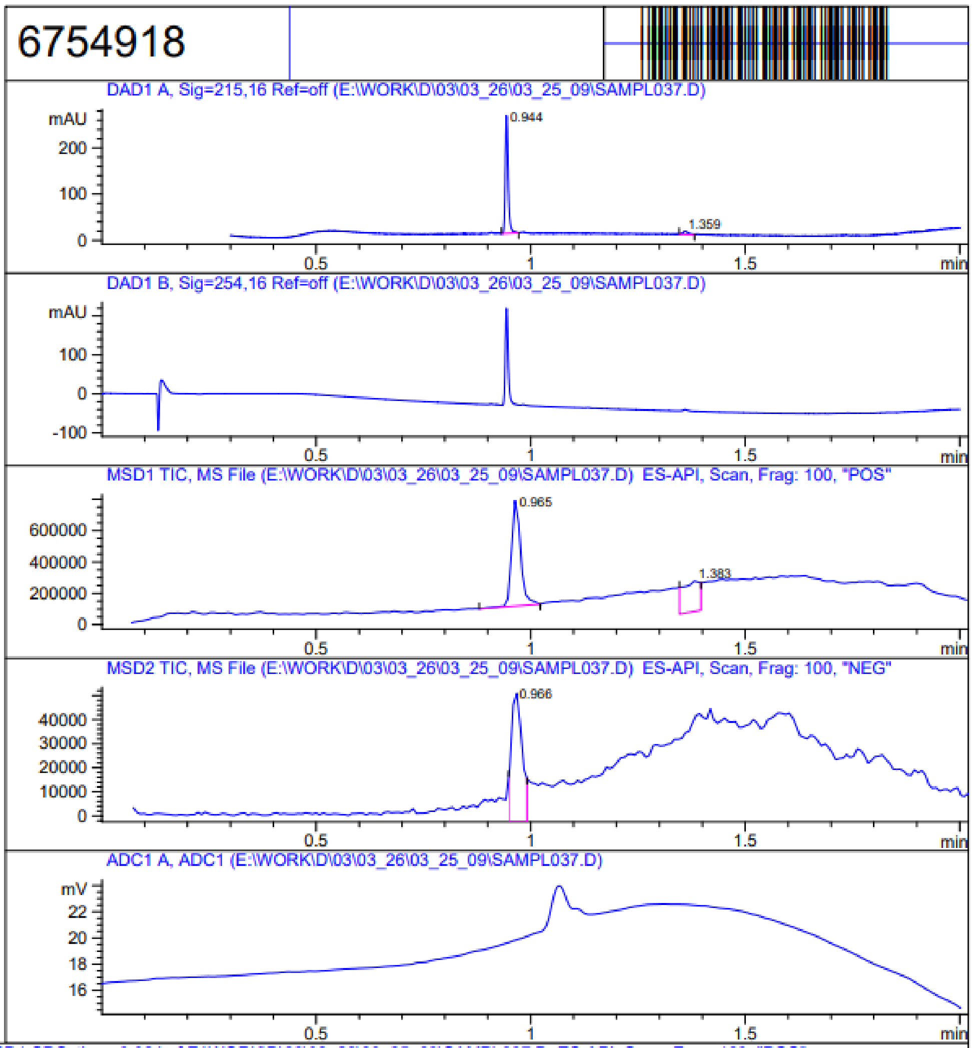

RT 0.965

RT 1.383

RT 0.966

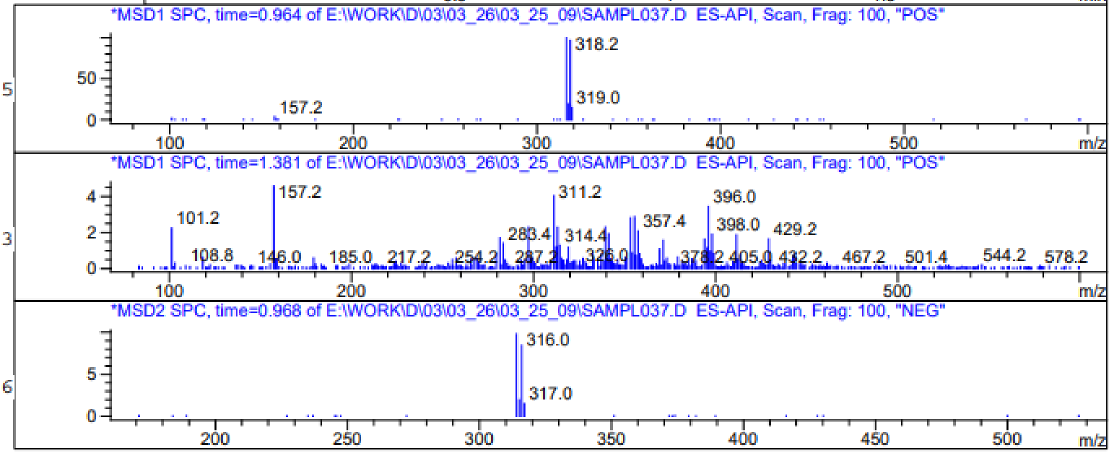

^{1}H NMR (500 MHz) spectrum of compound 2.48 (10 mM in DMSO-d6 at 298K).

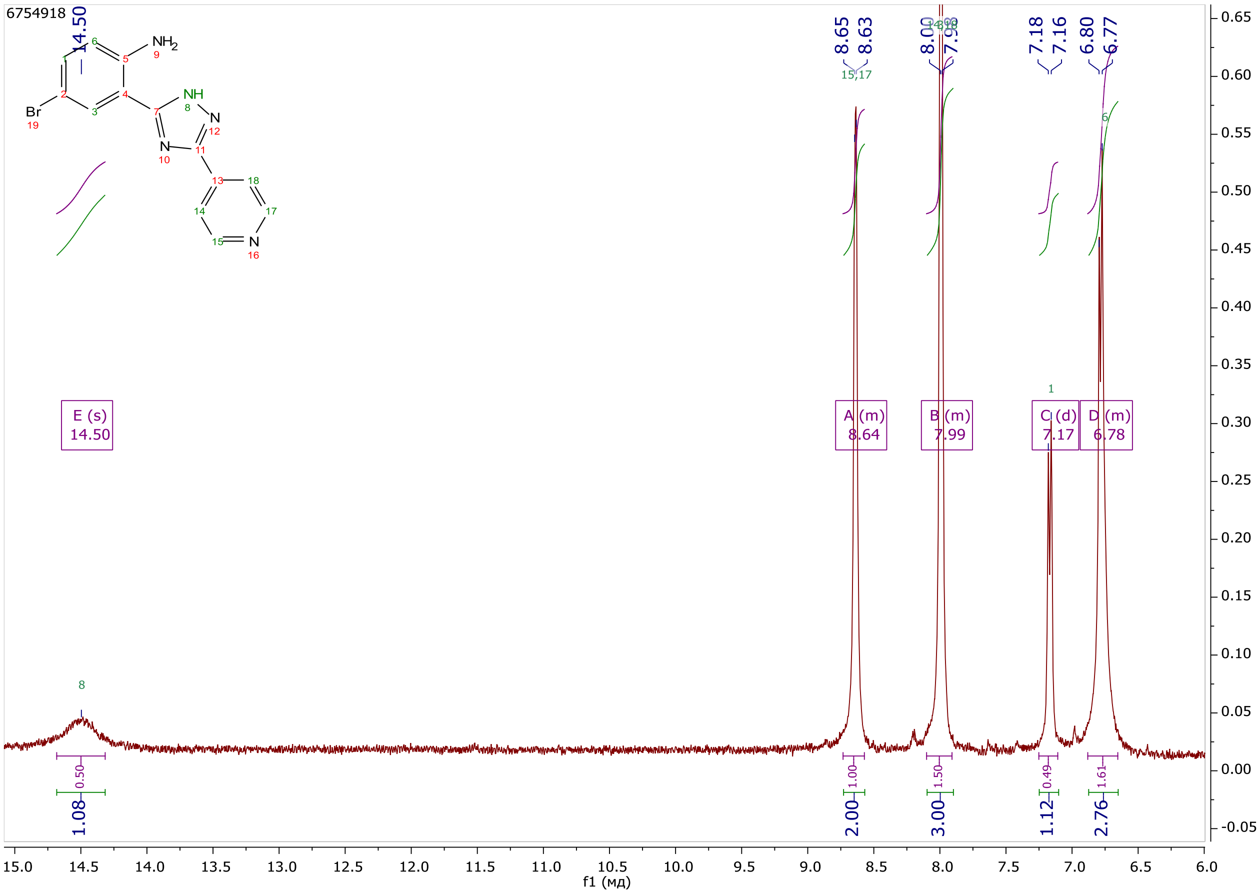

Supplement: Supplementary file 1 [file pharmaceuticals-18-00083-s001.zip › pharmaceuticals-3398371-supplementary.pdf]
